# The schizophrenia genetics knowledgebase: a comprehensive update of findings from candidate gene studies

**DOI:** 10.1038/s41398-019-0532-4

**Published:** 2019-08-27

**Authors:** Chenxing Liu, Tetsufumi Kanazawa, Ye Tian, Suriati Mohamed Saini, Serafino Mancuso, Md Shaki Mostaid, Atsushi Takahashi, Dai Zhang, Fuquan Zhang, Hao Yu, Hyoung Doo Shin, Hyun Sub Cheong, Masashi Ikeda, Michiaki Kubo, Nakao Iwata, Sung-Il Woo, Weihua Yue, Yoichiro Kamatani, Yongyong Shi, Zhiqiang Li, Ian Everall, Christos Pantelis, Chad Bousman

**Affiliations:** 1 Melbourne Neuropsychiatry Centre, Department of Psychiatry, University of Melbourne, Melbourne, VIC Australia; 2 Department of Human Genetics, Graduate School of Public Health, University of Pittsburgh, Pittsburgh, PA USA; 3 Department of Neuropsychiatry, Osaka Medical College, Osaka, Japan; 4 Florey Institute of Neuroscience and Mental Health, University of Melbourne, Melbourne, VIC Australia; 5 Department of Psychiatry, National University of Malaysia, Kuala Lumpur, Malaysia; 6 Department of Pharmacy, Brac University, Dhaka, Bangladesh; 7 Laboratory for Statistical Analysis, RIKEN Center for Integrative, Medical Sciences, Yokohama, Japan; 8 Laboratory for Omics Informatics, Omics Research Center, National Cerebral and Cardiovascular Center, Osaka, Japan; 9 Institute of Mental Health, Peking University Sixth Hospital, Beijing, China; 10 Key Laboratory of Mental Health, Ministry of Health and National Clinical Research Center for Mental Disorders (Peking University), Beijing, China; 11 Peking-Tsinghua Joint Center for Life Sciences/PKU-IDG/McGovern Institute for Brain Research, Peking University, Beijing, China; 12 Department of Psychiatry, The Affiliated Wuxi Mental Health Center of Nanjing Medical University, Wuxi, China; 13 Department of Psychiatry, Jining Medical University, Jining, China; 14 Department of Genetic Epidemiology, SNP Genetics, Inc., Seoul, Korea; 15 Department of Life Science, Sogang University, Seoul, Korea; 16 Department of Psychiatry, Fujita Health University School of Medicine, Toyoake, Japan; 17 RIKEN Center for Integrative Medical Sciences, Yokohama, Japan; 18 Soon Chun Hyang University Hospital, Seoul, Korea; 19 Center for Genomic Medicine, Kyoto University Graduate School of Medicine, Kyoto, Japan; 20 Bio-X Institutes, Key Laboratory for the Genetics of Developmental and Neuropsychiatric Disorders, Ministry of Education, Shanghai Jiao Tong University, Shanghai, China; 21 Institute of Psychiatry, Psychology, and Neuroscience, King’s College London, London, UK; 22 South London and Maudsley NHS Foundation Trust, London, UK; 23 Department of Electrical and Electronic Engineering, Centre for Neural Engineering (CfNE), University of Melbourne, Carlton South, VIC Australia; 24 NorthWestern Mental Health, Melbourne, VIC Australia; 25 Departments of Medical Genetics, Psychiatry, and Physiology & Pharmacology, University of Calgary, Calgary, AB Canada

**Keywords:** Genetics, Schizophrenia

## Abstract

Over 3000 candidate gene association studies have been performed to elucidate the genetic underpinnings of schizophrenia. However, a comprehensive evaluation of these studies’ findings has not been undertaken since the decommissioning of the schizophrenia gene (SzGene) database in 2011. As such, we systematically identified and carried out random-effects meta-analyses for all polymorphisms with four or more independent studies in schizophrenia along with a series of expanded meta-analyses incorporating published and unpublished genome-wide association (GWA) study data. Based on 550 meta-analyses, 11 SNPs in eight linkage disequilibrium (LD) independent loci showed Bonferroni-significant associations with schizophrenia. Expanded meta-analyses identified an additional 10 SNPs, for a total of 21 Bonferroni-significant SNPs in 14 LD-independent loci. Three of these loci (*MTHFR*, *DAOA*, *ARVCF*) had never been implicated by a schizophrenia GWA study. In sum, the present study has provided a comprehensive summary of the current schizophrenia genetics knowledgebase and has made available all the collected data as a resource for the research community.

## Introduction

Genome-wide association (GWA) studies have demonstrated that schizophrenia is a polygenic psychiatric disorder. The largest GWA study of schizophrenia to date has implicated more than 100 genetic loci associated with the disorder and future, even larger GWA studies are expected to increase the number of loci to >200^1^. Moreover, candidate gene studies continue to be conducted in schizophrenia, with an average of 100 published each year (Supplementary fig. S1). This unrelenting wave of schizophrenia genetic findings has made the most diligent readers among us struggle to follow the current evidence, leaving most of us unaware of findings from the vast majority of candidate gene studies.

In 2008, Allen et al. developed the SzGene database, a comprehensive catalogue of candidate gene studies in schizophrenia^2^. SzGene includes more than 1700 genetic association studies representing 1008 genes and 8788 polymorphisms. Since 2011 SzGene has not been updated and is now designated as a legacy database. As such, the thousands of candidate gene studies conducted in schizophrenia since 2011 have not been comprehensively captured and incorporated into this valuable schizophrenia knowledgebase. To remedy this issue and to provide an overview of candidate gene studies, we systematically identified all genetic association studies published in the field of schizophrenia between 2011–2017 and merged this information with the studies contained in the SzGene database.

## Methods

### Data collection from SzGene

Polymorphism data were collected from the SzGene database, encompassing general information of the study (PubMed ID, year of publication, first author name), discription of participants (diagnostic criteria, ancestry, sample size of case, control and trio), candidate gene name, genotyped SNP IDs and genotype distribution. The SzGene study also included family-based genetic studies, but the genotype distributions for these studies were not available in its database since these studies were not included in their meta-analyses. Thus, we gathered the counts of transmitted and non-transmitted alleles from these family studies. Furthermore, incorrect and incomplete information found in the SzGene database were revised after reviewing the original genetic study (see Supplementary Table S1 for a list of revised data).

### Search strategy

For the collection of genetic studies not assembled in SzGene database, a customized querying strategy (shown below) was developed based on the key words extracted from the abstracts of 1711 studies in the SzGene database.

(schizophrenia OR schizophrenic OR schizophrenics OR schizoaffective) AND (haplotypes OR haplotype OR allele OR alleles OR polymorphic OR polymorphism OR polymorphisms OR SNP OR SNPs) AND (“2010/01/01”[Date - Publication]: “2017/02/16”[Date - Publication])

The querying strategy was applied to PubMed electronic database to identify candidate gene studies. To further improve the coverage of studies, publication information of SNPs in dbSNP149 (Supplementary Table S2) was used as a second resource for querying genetic studies in schizophrenia.

### Literature review and data collection

We retrieved 3320 studies published between January 2010 and February 2017. Three reviewers (CL, SMS, YT) double-reviewed these studies, and disagreements were resolved through discussion with an independent reviewer (CB). Studies were processed for data collection if they were English-language articles with polymorphisms detected in schizophrenia patients (Supplementary Fig. S2). Information extracted from eligible studies included: PubMed ID, year of publication, authors, diagnostic criteria, ancestry of participants, sample size of cases, controls or trios, candidate gene name, genotyped SNP IDs and genotype/allele frequency data.

### Pre-processing

To systemically store and process data collected from published articles in a comparable and unified format, we conducted pre-processing steps to put electronic records in order. Supplementary Fig. S3 provides the workflow regarding the pre-processing steps, with detailed pre-processing information and outputs provided in Supplementary methods and Supplementary Tables S1, S3–S7.

### Inclusion/exclusion criteria

Studies were included in meta-analyses based on the following criteria: (1) the study was a case-control or family-based association study; (2) the study included genotype or allelic frequency data or sufficient summary statistics for meta-analysis and (3) the study was not an exon sequencing, whole-genome sequencing, or GWA study as raw data from these studies were not avaialble and the risk of overlap with the cases-control or family-based studies was assumed to be high.

For case-control studies to be included in the meta-analysis, one or more of the following data types was required: (1) genotype distributions of candidate SNPs in cases and controls; (2) allelic distributions of SNPs in cases and controls and (3) statistics related to an association test (p value, odds ratio and 95% confidence interval of odds ratio). When more than one of these data types were available, we prioritized genotyped distributions of SNPs. In family-based studies, counts of transmission versus non-transmissions of the reference allele in parents-offspring trios were used in the meta-analyses. We attempted to contact the corresponding authors to request data when the required data were not available or incomplete in the full-access article and supplements.

### Quality control

Minor allele frequency (MAF) information from the 1000 Genomes Project in African (AFR: pop_id = 16653 in dbSNP149), East Asian (pop_id = 16651), European (pop_id = 16652) and South Asian (pop_id = 16655) populations were downloaded from the dbSNP149 database (Supplementary Table S2). The risk and protective alleles of a SNP were switched if the reported allele frequency were considerably different from that in the corresponding 1000 genome population and the difference could not be attributed to known frequency discrepancies among subpopulations within the 1000 genome project data (see Supplementary Table S6). For the SNPs with complementary wild and variant alleles (that is, A–T or C–G transversion), meta-analysis results could be misleading because most of the published association studies did not provide DNA strand information for genotyped SNPs. To overcome this limitation caused by an ambiguous strand of A–T or C–G alleles, we manually checked the allele frequency of SNPs with complementary wild and variant alleles and aligned these SNPs to the forward strand based on the allele frequency obtained from the 1000 Genome Project Phase 3 (Supplementary Table S7). SNPs in which it was difficult to determine the forward/reverse strands according to allele frequency were excluded from the meta-analysis (Supplementary Table S7).

### Statistical analysis

Data from eligible studies were loaded into R (version 3.3.1) for statistical analysis. The meta-analysis was conducted using the “metafor” package and “meta” library in R. For all SNPs with four or more genetic studies, we computed an estimate of allelic effect (AE, referred to as treatment effect in ‘meta’ package) and standard error of allelic estimate (seAE), which are essential statistics for meta-analysis. Steps to obtain AE & seAE in case-control genetic studies were as follows^3^:

1 $${\it{OR}} = \frac{{{\it{C}}_a^{\left( 1 \right)}{\it{C}}_{\it{A}}^{\left( 0 \right)}}}{{{\it{C}}_A^{\left( 1 \right)}{\it{C}}_a^{\left( 0 \right)}}}$$

2 $${\it{AE}} = {\it{{\mathrm{log}}}}_{\it{e}}\left( {{\it{OR}}} \right)$$

3 $$\begin{array}{*{20}{l}} {{\mathrm{seAE}}} \hfill & = \hfill & {\sqrt {\frac{1}{{C_A^{\left( 1 \right)}}} + \frac{1}{{C_a^{\left( 1 \right)}}} + \frac{1}{{C_A^{\left( 0 \right)}}} + \frac{1}{{C_a^{\left( 0 \right)}}}} ,\left( {{\mathrm{if}}\,{\mathrm{allele/genotype}}\,{\mathrm{distribution}}\,{\mathrm{is}}\,{\mathrm{available}}} \right)} \hfill \\ {{\mathrm{seAE}}} \hfill & = \hfill & {\frac{{{\mathrm{AE}}}}{{ - 0.862 + \sqrt {0.743 - 2.404 \times {\it{log}}_{\it{e}}\left( p \right)} }}.\left( {{\mathrm{if}}\,{\mathrm{p}}\,{\mathrm{value}}\,{\mathrm{if}}\,{\mathrm{available}}} \right)} \hfill \\ {{\mathrm{seAE}}} \hfill & = \hfill & {{\it{CI}}_{{\it{up}}} - {\it{OR }}\left( {{\mathrm{if}}\,{\mathrm{confidence}}\,{\mathrm{interval}}\,{\mathrm{is}}\,{\mathrm{available}}} \right)} \hfill \end{array}$$

where *OR* is odds ratio of candidate SNP, *CI*_*up*_ is the upper bound of 95% confidence interval, and ${\it{C}}_{\it{A}}^{\left( 1 \right)}$, ${\it{C}}_{\it{a}}^{\left( 1 \right)}$, ${\it{C}}_{\it{A}}^{\left( 0 \right)}$, and ${\it{C}}_{\it{a}}^{\left( 0 \right)}$ were counts of A/a alleles in case and control groups, respectively.

For family-based stuides we adopted the approach for obtaining AE and seAE proposed by Kazeem and Farrall:

1 $${\mathrm{AE}} = {\it{log}}_{\it{e}}\left( {{\it{C}}_{\it{T}}/{\it{C}}_{\it{N}}} \right)$$

2 $${\mathrm{seAE}} = \frac{1}{{{\it{C}}_{\it{T}}}} + \frac{1}{{{\it{C}}_{\it{N}}}}$$

where *C*_*T*_ is the number of transmissions of the reference allele from parents to schizophrenia case, and *C*_*N*_ is the number of non-transmissions of the reference allele. The odds ratio and 95% confidence interval were obtained according to both the fix-effects and random-effects models. For random-effects models we obtained summary ORs and 95% CIs with the DerSimonian and Laird^4^, which utilizes weights that incorporate both the within-study and between-study variance. The meta-analysis pipeline was first applied to studies regardless of population and then repeated in Caucasian and Asian populations separately.

### Heterogeneity

We used Higgins’s *I*^2^ statistic to quantify between-study heterogeneity caused by differences in study design and ancestry. The *I*^2^ statistic was calculated as ${\it{I}}^2 = \left[ {{\it{Q}} - \left( {{\it{K}} - 1} \right)} \right] \times 100$, where Q is Cochran’s Q statistic and (K-1) is the degree of freedom. A large *I*^2^ value indicates significant heterogeneity among studies and the CI bounds calculated by the random-effects model are more reliable in the presence of high heterogeneity.

### Sensitivity analyses

To evaluate the impact of outlier and influential cases we employed an approach recommend by Viechtbauer and Cheung^5^. Studies were considered as outliers if the absolute value of studentized deleted residuals was greater than 1.96. To test the influence of each study, we conducted sensitivity tests applying a leave-one-out analysis approach, which excludes each study in turn. A study was considered an influential case if it led to considerable changes in the leave-one-out analysis, which can be determined by the DFFITS, DFBETAS and COVRATIO statistics. In addition to outlier and influential cases, we also performed sensitivity analyses, excluding studies that deviated from HWE at *p* < 0.05.

### Publication bias

Funnel plots analysis were performed to detect potential publication bias, and the regression test proposed by Harbord^6^ was used to quantify the asymmetry in the funnel plot. If publication bias was identified, the”trimfill” function in metafor with Viechtbauer’s recommended parameter^7^ was used to impute the missing studies in the funnel plot and to obtain the estimate of the effect size after adjustment for publication bias. According to the recommendations from Sterne et al.^8^, the test for publication bias was only applied to SNPs with 10 or more studies.

### Comparison with recent meta-analysis studies

To ensure completeness of our systematic literature search, we compared the studies identified by of our search to that of meta-analyses published between 1 January 2017, and 31 December 2017 using the following PubMed query:

(((schizophrenia) AND (meta OR meta-analysis)) AND (polymorphism OR polymorphisms OR SNP OR SNPs)) AND (“2017/01/01”[Date - Publication]: “2017/12/31”[Date - Publication])

### Collection of GWAS samples and expanded meta-analyses

To increase statistical power of our analyses we performed two expanded meta-analyses that combined our candidate gene study data with GWA study data obtained from the Psychiatric Genomics Consortium (PGC) schizophrenia GWA study (i.e. PGC2), four published Asian GWA studies, and an unpublished Chinese GWA study (see Supplementary methods for detailed descriptions). Candidate gene studies in Caucasians were excluded from the combined analyses due to the suspected high-degree of overlap between these samples and those included in the PGC2 dataset. We applied meta-analyses for SNPs that were nominally significant in the candidate-gene meta-analyses. The expanded meta-analyses combined GWA study data using the META software developed by Liu et al.^9^. The Z-statistic and *p*-value were estimated using the inverse-variance method based on a fixed-effects model, but for SNPs with pronounced heterogeneity (*I*^2^ > 75%), the Z-statistic was computed using a random-effect model^10^.

## Results

Our systematic search identified a total of 3320 publications examining 20,570 single nucleotide polymorphisms (SNPs) in more than 3414 genes. After applying inclusion and exclusion criteria and quality control procedures (see online methods), 1188 publications investigating 550 SNPs in 168 genes were eligible for meta-analysis (Supplementary Fig. S2), representing a doubling of the number of SNPs included in the last update of the SzGene database (Supplementary Table S8**)**. Comparison of our literature search strategy with 21 schizophrenia genetic meta-analyses published after 2017, showed our search strategy was robust and more comprehensive than the reference meta-analyses, with exception of four meta-analyses that included GWA studies, non-English articles, and/or studies published after our inclusion date (Supplementary Table S9).

The median number of studies and combined sample size (case and controls) for the meta-analyses performed were five (interquartile range (IQR) = 4–8) and 5542 (IQR = 3,198–8,709), respectively. Among the 550 SNPs subjected to meta-analyses, 82 SNPs were nominally associated with schizophrenia in Asians and/or Caucasians after adjusting for publication bias and sensitivity analyses (Supplementary Tables S10–S13, and Supplementary Data S2). Among the 29 SNPs that were significant (*p*-value < 0.05) in our meta-analysis and the PGC GWAS, the effect estimate (i.e. odds ratio) was in the same direction for 79% (*n* = 23) of them (Supplementary Table S10). Following Bonferroni correction (*p* < 9.09 × 10^−5^), 11 SNPs in eight linkage disequilibrium (LD) independent loci remained significant (Table 1). Five of these top eight LD-independent loci (*MTHFR*, *PDE4B, DAOA, ARVCF, TRAM1L1/NDST3*) were not located within risk loci identified in the largest schizophrenia GWA study produced by the PGC and three loci (*MTHFR*, *DAOA*, *ARVCF*) had never been implicated by a schizophrenia GWA study.

**Table 1:** Bonferroni associations in candidate gene meta-analyses

SNP	Gene	Chr	Position	Reference allele	Population	Cases vs controls (studies included)	*P*-value^a^	OR (95% CI)^b^	*I* ^2^	PGC (p)	PGC (odds ratio)
rs1801133	MTHFR	1	11856378	T	All populations	14242 vs 18541 (45)	2.61E-05^c^	1.12 (1.06–1.18)	57	0.55	1.0068
rs910694	PDE4B	1	66795976	C	All populations	1523 vs 1258 (4)	1.05E-05	0.77 (0.68–0.86)	2	0.48	0.99
rs1344706	ZNF804A	2	185778428	G	All populations	19724 vs 22368 (33)	1.72E-05^c^	0.92 (0.89–0.96)	31	1.27E-10	0.93
rs11098403	TRAM1L1/NDST3	4	118646907	G	Asian	1861 vs 2081 (4)	1.45E-10	0.65 (0.58–0.75)	0	3.52E-05	1.05
rs6932590	PRSS16/POM121L2	6	27248931	C	All populations	8351 vs 15485 (15)	3.05E-07^c^	0.86 (0.81–0.91)	0	1.02E-20	0.89
rs13211507	PGBD1	6	28257377	C	Caucasian	4914 vs 12522 (13)	1.47E-06	0.76 (0.68–0.85)	7	6.28E-26	0.81
rs2071287	NOTCH4	6	32170433	A	Asian	6608 vs 12918 (6)	4.38E-05	0.89 (0.85–0.94)	14	N.A	N.A
rs3131296	NOTCH4	6	32172993	A	All populations	6112 vs 13633 (15)	2.22E-07^c^	0.79 (0.73–0.87)	0	N.A	N.A
rs1006737	CACNA1C	12	2345295	A	All populations	14337 vs 15969 (9)	5.61E-07^c^	1.18 (1.1–1.25)	0	1.09E-16	1.1
rs778293	DAOA/-	13	106169199	G	Asian	2899 vs 3218 (4)	1.60E-05	1.18 (1.09–1.27)	0	0.53	0.99
rs165815	ARVCF	22	19959473	C	All populations	2982 vs 3253 (6)	6.36E-07^c^	1.64 (1.35–1.99)	0	0.04	0.97

The table presents Bonferroni-significant polymorphisms in Asians and/or Caucasians after adjusting for publication bias and sensitivity analyses

^a^The p value and odds ratio were calculated based on random-effect model

^b^Odds ratio was calculated under allelic model corresponding to the reference allele

^c^We present the results (*p*-value and odds ratio) in sensitivity analysis for the polymorphism

To further explore the 82 nominally associated SNPs identified in our candidate gene meta-analyses, we conducted two sets of expanded meta-analyses that combined candidate gene study data with schizophrenia GWA study data. In both sets of expanded meta-analyses, 61 SNPs with actual or imputed genotypes from the 82 SNPs found to be nominally associated with schizophrenia in our candidate-gene meta-analyses were examined (Supplementary Table S10).

In the first set of expanded meta-analyses, we focused on Asian studies only. Combined data from five Asian schizophrenia GWA studies (14,433 cases and 26,797 controls, see Supplementary methods) and applicable Asian candidate-gene studies revealed two Bonferroni-significant LD-independent loci (*CNNM2, MPC2*) not identified in our candidate gene only meta-analyses (Supplementary Table S14, and Supplementary Data S3). The *CNNM2* gene had not previoulsy been implicated in an Asian GWA study of schizophrenia, although among Caucasians in the PGC schizophrenia GWA study this gene was located within one of the 108 loci associated with schizophrenia^1^. Whereas the locus represented by *MPC2*, had not been implicated previously by the PGC schizophrenia GWA study but was indentified as a genome-wide significant (*p* = 1.00 × 10^−8^) locus in a previous Asian GWA study^11^.

**Fig. 1:**
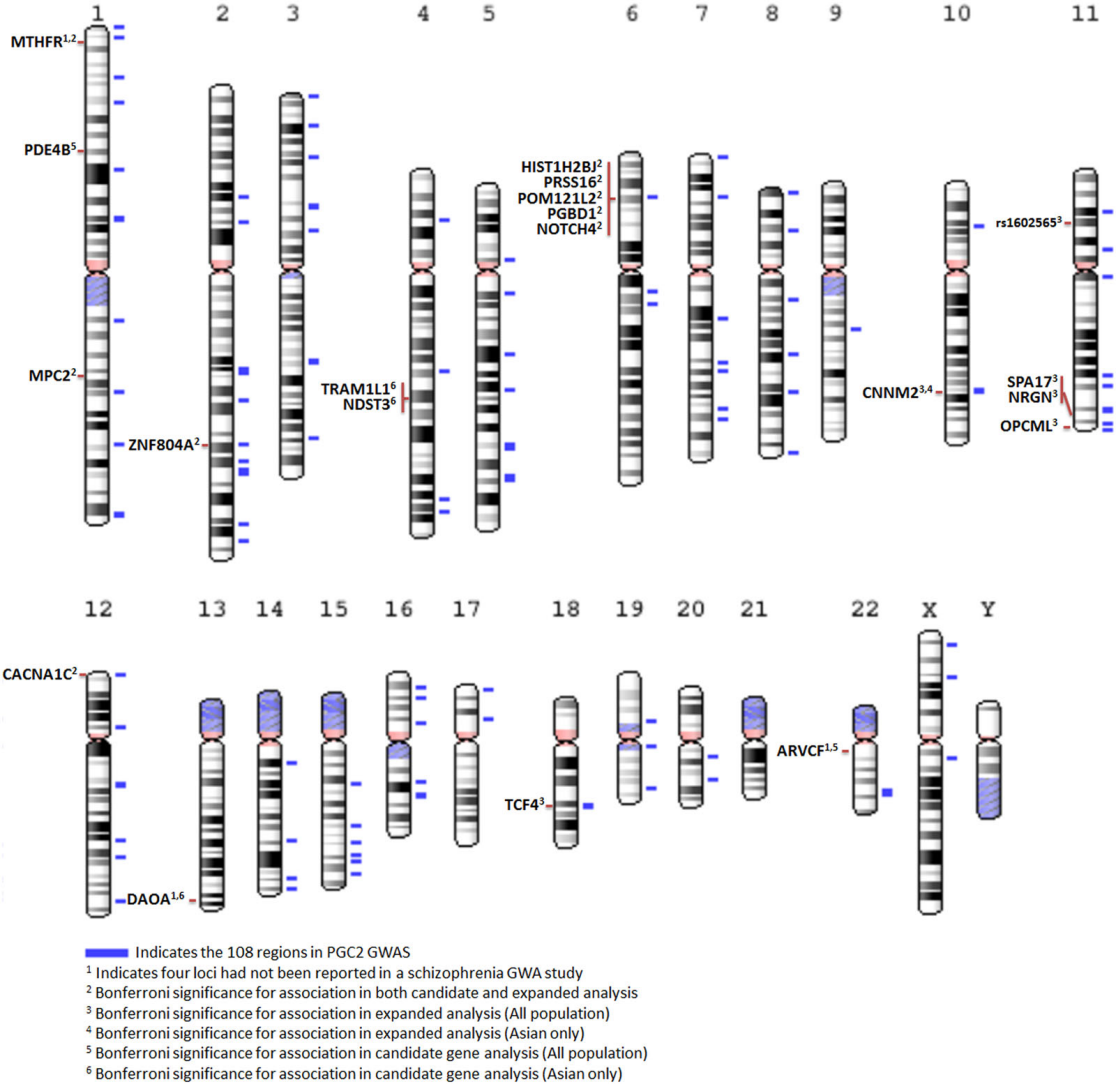
Chromosome ideogram presents significant loci after Bonferroni correction

In the second set of expanded meta-analyses, Caucasian data from the PGC schizophrenia GWA study (34,241 cases and 45,604 control)^1^ was combined with the Asian candidate gene and GWA study data. Caucasian candidate gene study data indentified in our systematic search were not included because of high suspected overlaps in participants between these studies and the PGC GWA study. Meta-analyses of these combined data showed four additional Bonferroni-significant LD-independent loci *(SPA17*/*NRGN, OPCML, TCF4*, chr11 intergnetic region). Two of these loci, represented by *OPCML* and an intergenic region (rs1602565) in chromosome 11, did not sit within one of the 108 schizophrenia risk loci identified by the PGC GWA study (Supplementary Table S15, and Supplementary Data S4). Although, a GWA study conducted by O’Donnovan and collegues (2008) reported nominal associations between schizophrenia and both these loci (*OPCML*: *p* = 5.63 × 10^−4^; rs1602565: *p* = 2.99 × 10^−6^)^12^. Altogether, our meta-analyses (candidate gene only and expanded with GWA studies) identified 14 Bonferroni LD-independent loci (Fig. 1, Supplementary Table S16).

## Discussions

Our study represents the first comprehensive update of the schizophrenia knowledgebase in nearly a decade. We identified 21 Bonferroni-significant SNPs in 14 LD-independent loci associated with schizophrenia susceptibility. Three (*MTHFR*, *DAOA*, *ARVCF*) of these loci had not been reported in a schizophrenia GWA study within the Asian or Caucasian populations. However, the association result of MTHFR, DAOA and ARVCF didn’t achieve the GWAS-significant threshold (*p* < 10E-08), more association analyses in expended population are needed before further functional study.

Our findings support the notion that genetic risk loci for schizophrenia can be shared across or be specific to particular populations. *CNNM2* is an example of a shared locus in Caucasian and Asian populations. The PGC schizophrenia GWA study implicated *CNNM2* among Caucasians and our current findings echoed this association in Asians. Conversely, *MPC2* is an example of a population specific locus. The PGC schizophrenia GWA study did not identify *MPC2* as a significant locus but in our Asian only meta-analyses we found a robust association between this gene and schizophrenia.

In our candidate gene meta-analyses, 11 SNPs were identified after Bonferroni correction. However, three of them (rs165815 in ARVCF, rs910694 in PDE4B and rs11098403 in the intergenic region between TRAM1L1 and NDST3) should be interpreted with caution because of their insufficient sample sizes and uncharacteristically large odds ratios. Moreover, neither of these SNPs was replicated in our second-round meta-analyses combined with GWAS results. The other seven genes/loci (PDE4B, ZNF804A, PRSS16/POM121L2, PGBD1, NOTCH4 and CACNA1C) were reported by previous GWAS, suggesting most of the candidate gene studies that identified these SNPs were motivated by prior GWAS findings. In this sense, candidate gene studies didn’t provide much new insight into the pathology of schizophrenia in addition to GWAS, which was consistent with the viewpoint summarized by Johnson et al. and Farrell et al^13,14^. Previous reviews have suggested that biological-based candidate genes were not more likely to be associated with schizophrenia compared with non-candidate genes. Our systematic review further revealed that an increasing number of association studies have been GWAS-motivated rather than the conventional biological-based candidate gene studies. Thus, candidate gene studies appear to have been conducted more for the purpose of replicating GWAS results, rather than providing new insights on the genetic mechanism of schizophrenia.

The results of our meta-analyses are not without notable limitations. First, our quality control procedures attempted to identify studies that had obvious overlapping samples. However, we cannot guarantee duplicate samples were not included and as such estimated pooled effect sizes could be negatively or positively biased. Second, several loci identified in the candidate gene meta-analyses had larger than would be expected pooled odds ratios (OR < 0.8, OR > 1.2) due to the limited sample sizes available. In fact, three (*PDE4B, TRAM1L1/NDST3, ARVCF*) of the eight Bonferroni-significant loci identified in the candidate gene meta-analyses did not remain significant in the expanded meta-analyses, warranting cautious interpretation and further validation of these loci. Third, the exclusion of non-English articles and limited success at obtaining access to unpublished data from authors of otherwise eligible studies might have biased our results. Fourth, we did not employ analytical approaches commonly used in single SNP-based meta-analyses to estimate pooled effects under various genetic models (e.g. dominant, recessive) or among haplotypes, nor did we employ meta-regression techniques to adjust for potential confounding factors. These decisions were based on the data available to us for the majority of studies we identified and the difficulty of obtaining the necessary data from authors to adequately perform these additional analyses. Fifth, population stratification is a major bias that can affect genetic association results and is rarely accounted for in candidate gene association studies. Finally, the random-effects model is typically viewed as a conservative approach to meta-analyses; however, in the context of funnel plot asymmetry (i.e. effect estimates are related to standard errors) random-effects models can attenuate the influence of large studies and inflate the influence of small studies.

In sum, we have provided the first comprehensive update of the schizophrenia genetics knowledgebase since the decommissioning of the SZGene database in 2011^2^. Although candidate gene studies were an essential contributor to schizophrenia genetics a decade ago, our study findings suggest that these studies have had a modest contribution to the understanding of schizophrenia since the emergence of GWAS technology. Despite these findings, traditional candidate gene studies will likely continue but we anticipate that these studies will be used more frequently to follow-up GWAS implicated genetic variants within deeply phenotyped individuals from which greater insights on the clinical and functional relevance of these variants may be gained.

## Supplementary information

Supplementary methods

Figure S1

Figure S2

Figure S3

Supplementary Data S2

Supplementary Data S3

Supplementary Data S4

Table S1

Table S2

Table S3

Table S4

Table S5

Table S6

Table S7

Table S8

Table S9

Table S10

Table S11

Table S12

Table S13

Table S14

Table S15

Table S16

Supplementary Dataset

## Data Availability

Data used for meta-analyses are provided in the Supplementary Data S1 and the R script used for meta-analyses is available upon request.

## References

[1] Working Group of the Psychiatric Genomics Consortium. (2014). Biological insights from 108 schizophrenia-associated genetic loci. Nature.

[2] Allen NC (2008). Systematic meta-analyses and field synopsis of genetic association studies in schizophrenia: the SzGene database. Nat. Genet..

[3] Altman, D. G. & Bland, J. M. How to obtain the confidence interval from a P value. *BMJ* **343**, d2090 (2011). 10.1136/bmj.d2090 21824904

[4] DerSimonian R, Laird N (1986). Meta-analysis in clinical trials. Control. Clin. trials.

[5] Viechtbauer W, Cheung MW-L (2010). Outlier and influence diagnostics for meta-analysis. Res. Synth. methods.

[6] Harbord RM, Egger M, Sterne JAC (2006). A modified test for small-study effects in meta-analyses of controlled trials with binary endpoints. Stat. Med..

[7] Wolfgang Viechtbauer. Conducting Meta Analyses in R with the metafor Package. *J. Stat. Softw*. **36,** 1–48 (2010).

[8] Sterne JAC (2011). Recommendations for examining and interpreting funnel plot asymmetry in meta-analyses of randomised controlled trials. BMJ.

[9] Liu JZ (2010). Meta-analysis and imputation refines the association of 15q25 with smoking quantity. Nat. Genet..

[10] Winkler TW (2014). Quality control and conduct of genome-wide association meta-analyses. Nat. Protoc..

[11] Shi Y (2011). Common variants on 8p12 and 1q24.2 confer risk of schizophrenia. Nat. Genet..

[12] O’Donovan MC (2008). Identification of loci associated with schizophrenia by genome-wide association and follow-up. Nat. Genet..

[13] Johnson EC (2017). No evidence that schizophrenia candidate genes are more associated with schizophrenia than noncandidate genes. Biol. Psychiatry.

[14] Farrell MS (2015). Evaluating historical candidate genes for schizophrenia. Mol. psychiatry.

