# Supplementary material for: The schizophrenia genetics knowledgebase: a comprehensive update of findings from candidate gene studies
Source: Transl Psychiatry. 2019 Aug 27;9:205. doi: 10.1038/s41398-019-0532-4 (PMC6711957; doi:10.1038/s41398-019-0532-4)
Supplement: Supplementary file 1 — Supplementary methods [file 41398_2019_532_MOESM1_ESM.pdf]

SUPPLEMENTARY METHODS

The schizophrenia genetics knowledgebase: A comprehensive update of findings from candidate gene studies

Table of Contents

Pre-processing steps 2

Collection of GWAS samples 3

Supplementary Figures

Figure S1. Progress of candidate gene study over years 7

Figure S2. Workflow of systematic review 8

Figure S3. Workflow of pre-processing steps 9

References 10

Supplementary methods

Pre-processing steps

Diagnosis: Schizophrenia patients were categorized as "narrow-SZ" and "broad-SZ," where the definition of "narrow-SZ" encompasses diagnoses of schizophrenia and schizoaffective patients, and the "broad-SZ" includes disorders within the ICD category of "Schizophrenia (F20)" or DSM category of "Schizophrenia and other psychotic disorders. Only genotype/allelic data collected form studies using a narrow-SZ definition were used in our meta-analyses, which is consistent with the criteria used in the SzGene meta-anayses. Although genetic studies of broad-SZ patients were reviewed and kept in our electronic database, they were excluded from meta-analysis if more than 10% patients were diagnosed as broad-SZ and the raw data of narrow-SZ patients was not provided separately.

Diagnostic Criteria: DSM-IV and ICD-10 criteria have been utilized in the majority of genetic studies in schizophrenia published after 2010, while Research Diagnostic Criteria (RDC), Chinese classification of mental diseases (CCMD), DSM-III and other assessments were adopted in ~11.8% of the studies. In our database, studies with patients diagnosed according to more than one criterion have been labelled as "Mix criteria", and studies with patients diagnosed by CCMD or unclassified criteria have been labelled as "Other criteria".

Polymorphism: As naming conventions of polymorphisms change over time, different names or aliases might be used in studies for the same polymorphism. We converted the name of SNP to the dbSNP identifier (rs ID) using the annotated information from the NCBI dbSNP149

database (**Supplementary Table S3**). For human polymorphisms of which the dbSNP identifier is not available, we designated a unified name based on its name in the SzGene database or the most common name among genetic studies. Only biallelic SNPs with dbSNP identifiers (rs ID) were included in the current meta-analyses.

Ethnicity/Race: For each study, the ethnicity/race of participants were categorized into four subgroups: Caucasian, Asian, African, and other/mixed. Studies reporting recruitment of participants from United States of America, Canada, Australia and other multiracial countries that did not specify ethnicity/race of participants were recorded as “other/mixed”. Participants from countries having clear racial and ethnic groups were assigned to the corresponding category based on the population information and the recruiting region provided in the studies (**Supplementary Table S4**).

Studies with overlapping samples: Publications with suspected overlap in samples were identified by scanning the summary statistics and publication information of the studies. For each pair of studies with duplicate participants that examined the same SNP, the study with the smaller sample size was removed from the meta-analysis (**Supplementary Table S5**).

Collection of GWAS samples

PGC2 GWAS: We obtained the summary results of PGC2 GWA study from the PGC website (see URLs). It consisted of 49 case-control studies (34,241 cases vs. 45,604 control) and three family-designed studies (1,235 trios). There were no overlapping samples between the three Asian GWA studies included in the PGC and our five Asian GWA studies from China, Japan,

and Korea. Any SNPs with an imputation INFO score of $\geqslant$ 0.6 after quality control were included in our combined analysis. For more details, please see the study by Schizophrenia Working Group of the Psychiatric Genomics Consortium[1].

Chinese GWAS set 1: The Chinese GWA study was conducted by the Peking University Sixth Hospital, combining four independent GWA study datasets recruited from multiple hospitals in China. Schizophrenia patients and health controls of Han Chinese ancestry were recruited from multiple hospitals in China. All patients met DSM-IV diagnosis for schizophrenia according to at least two experienced psychiatrists. Written informed consent was obtained from all participants, and the study was approved by the Institutional Ethical Committee of each hospital. After quality control, the dataset consisted of 4,384 cases and 5,770 controls. The summary statistics of 82 SNPs was extracted from the dataset after the imputation of untyped markers. Details of genotyping platforms, quality controls, and imputation approaches can be found elsewhere[2].

Chinese GWAS set 2: The second Chinese GWA study was conducted by the Bio-X Institutes of Shanghai Jiao Tong University, with 7,699 cases and 18,327 controls Chinese individuals genotyped by Affymetrix or Illumina Arrays[3]. Diagnoses of patients were performed by two independent psychiatrists in accordance with DSM-IV diagnostic criteria for schizophrenia. The same quality control and imputation processes were applied to several GWAS samples enrolled in the study. The study was reviewed and approved by the Ethics Committee of Human Genetic Resources at the Bio-X Institutes of Shanghai Jiao Tong University. The

association results of SNPs were extracted from this combined GWAS set.

Chinese GWAS set 3: The GWA study dataset consisted of 350 cases and 350 controls genotyped by the Illumina PsychArray platform. Participants were recruited from Shanxi and Jiangsu Province in China, and the study was approved by the Ethics Committee at The Affiliated Wuxi Mental Health Center, Nanjing Medical University. Diagnoses of patients were performed by two independent psychiatrists according to DSM-IV criteria for schizophrenia. For the quality control of GWAS data, we adhered to the procedures of previous study[4].

Japanese GWAS set: The Japanese GWAS study initially included 2,131 cases genotyped by the Illumina Human Omni Express Exome v1.0 chip, with 1940 cases remaining after quality control[5]. The comparison group was 7,408 non-psychiatric controls recruited for the BioBank Japan Project. We then performed genotype imputation using the Asian subset (Japanese in Tokyo (JPT), Han Chinese in Beijing (CHB), and Southern Han Chinese (CHS) populations) from the 1000 Genomes Project Phase I dataset as reference.

Korean GWAS set: The Korean GWA study in schizophrenia included 350 cases and 700 controls from five hospitals in Korea. The Korean set was genotyped using the Illumina HumanOmni1 Array. Diagnoses of patients were performed by trained psychiatrist following the DSM-IV criteria of schizophrenia. For further details on this study see Ref. [6]. Genotype imputation with the 1000 Genomes Project Phase 3 reference panel was conducted using the Michigan Imputation Server. The study samples were phased using SHAPEIT (v2.r790)

followed by imputation using minimac3 (v2.0.1).

Supplementary Figures

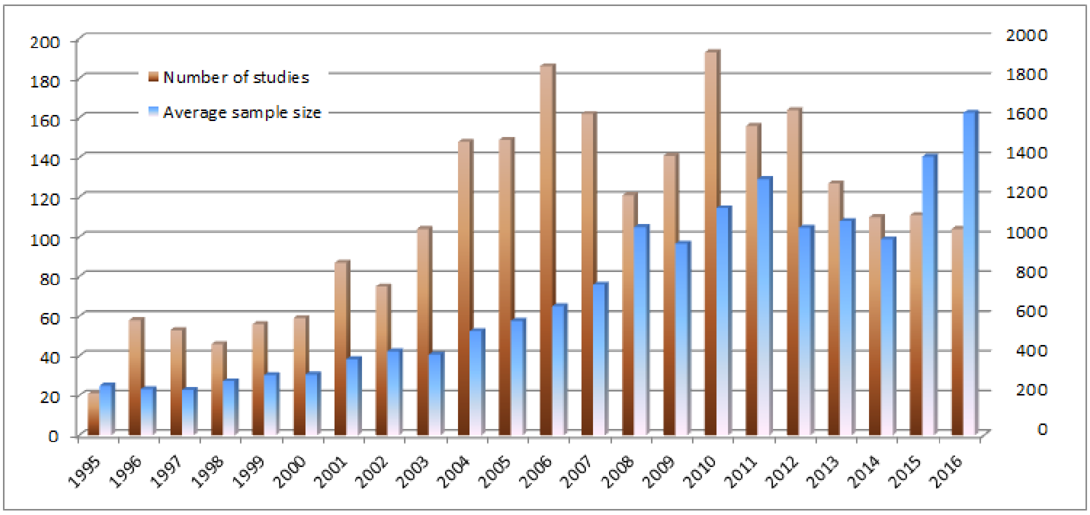

Supplementary Figure S1. Progress of candidate gene study over years

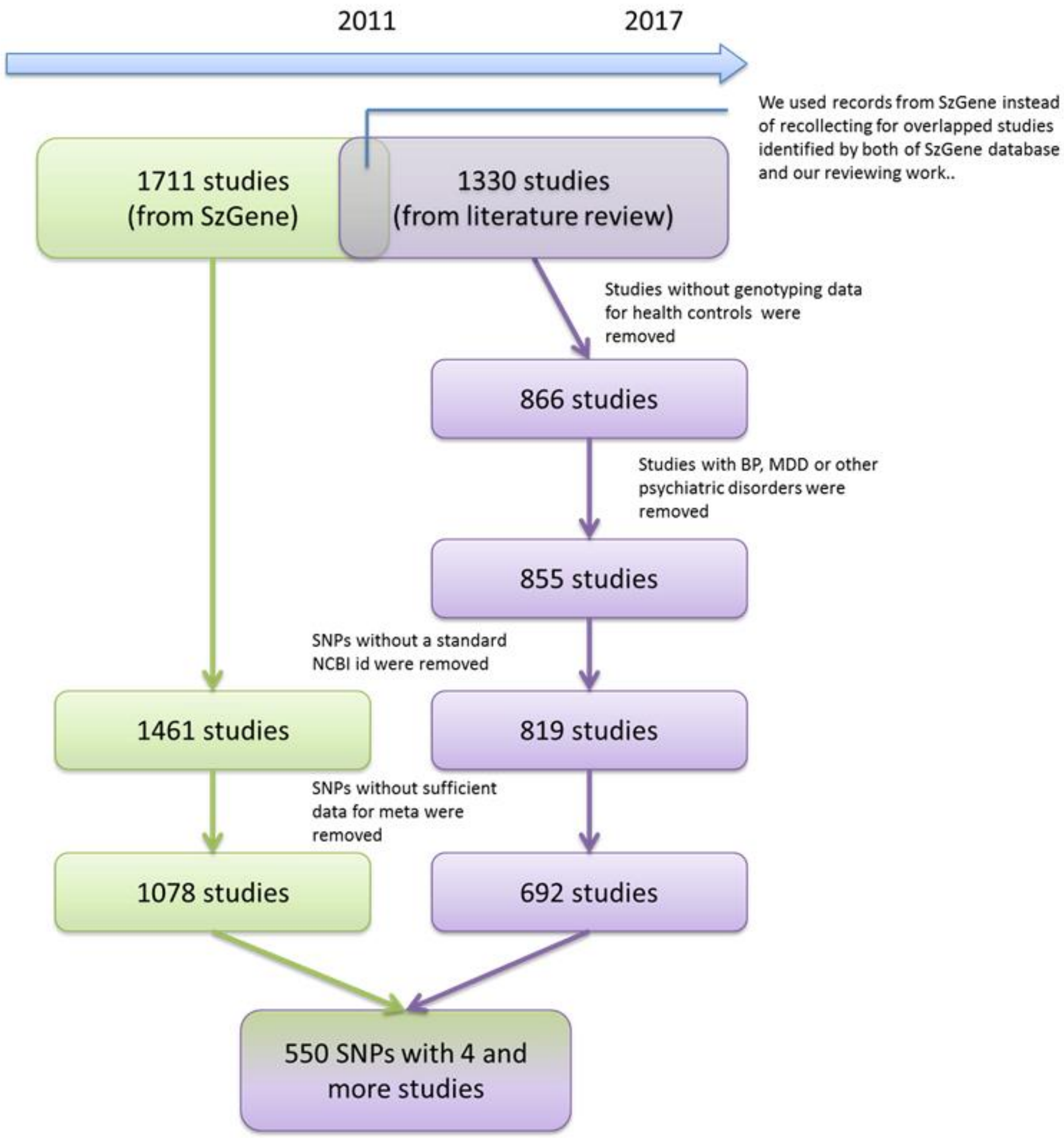

Supplementary Figure S2. Workflow of systematic review

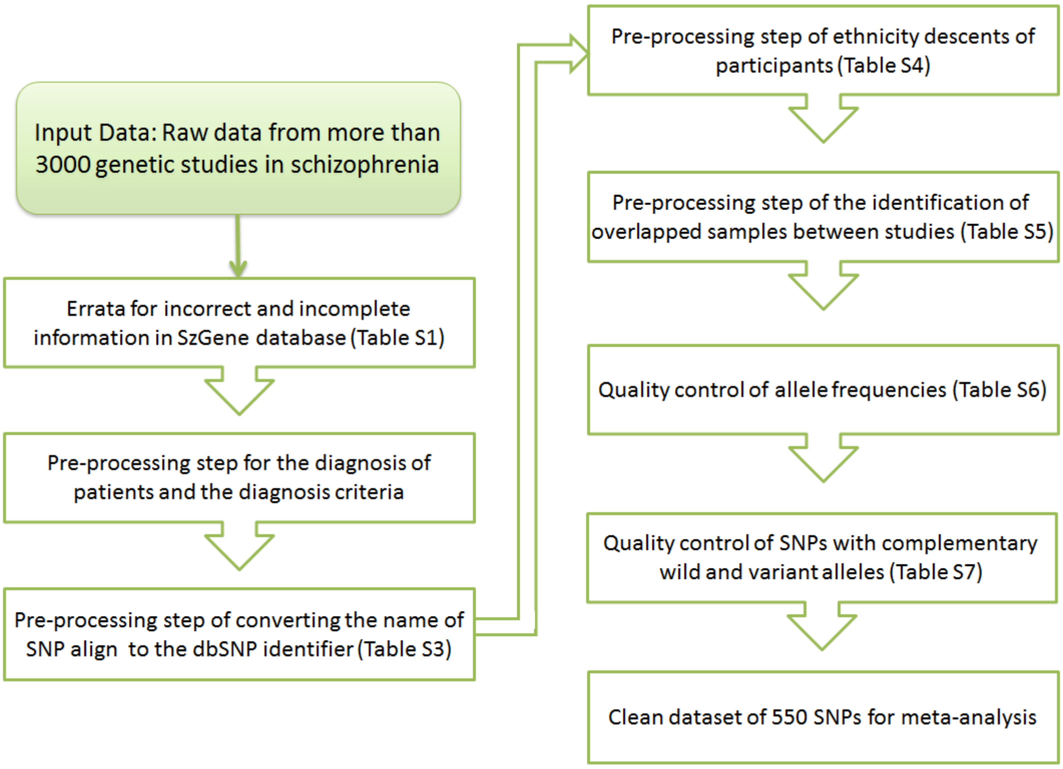

Supplementary Figure S3. Workflow of pre-processing steps

References

1. Working Group of the Psychiatric Genomics Consortium. Biological insights from 108 schizophrenia-associated genetic loci. *Nature*. 2014;511(7510):421-427.
2. Yu H, Yan H, Li J, et al. Common variants on 2p16.1, 6p22.1 and 10q24.32 are associated with schizophrenia in Han Chinese population. *Molecular psychiatry*. 2017;22(7):954-960.
3. Li Z, Chen J, Yu H, et al. Genome-wide association analysis identifies 30 new susceptibility loci for schizophrenia. *Nature genetics*. 2017;49(11):1576-1583.
4. Anderson CA, Pettersson FH, Clarke GM, Cardon LR, Morris AP, Zondervan KT. Data quality control in genetic case-control association studies. *Nature protocols*. 2010;5(9):1564-1573.
5. Ikeda M, Takahashi A, Kamatani Y, et al. Genome-Wide Association Study Detected Novel Susceptibility Genes for Schizophrenia and Shared Trans-Populations/Diseases Genetic Effect. *Schizophrenia bulletin*. 2018.
6. Kim LH, Park BL, Cheong HS, et al. Genome-wide association study with the risk of schizophrenia in a Korean population. *American journal of medical genetics. Part B, Neuropsychiatric genetics : the official publication of the International Society of Psychiatric Genetics*. 2016;171B(2):257-265.
