# Supplementary figures and images for: The schizophrenia genetics knowledgebase: a comprehensive update of findings from candidate gene studies
Source: Transl Psychiatry. 2019 Aug 27;9:205. doi: 10.1038/s41398-019-0532-4 (PMC6711957; doi:10.1038/s41398-019-0532-4)

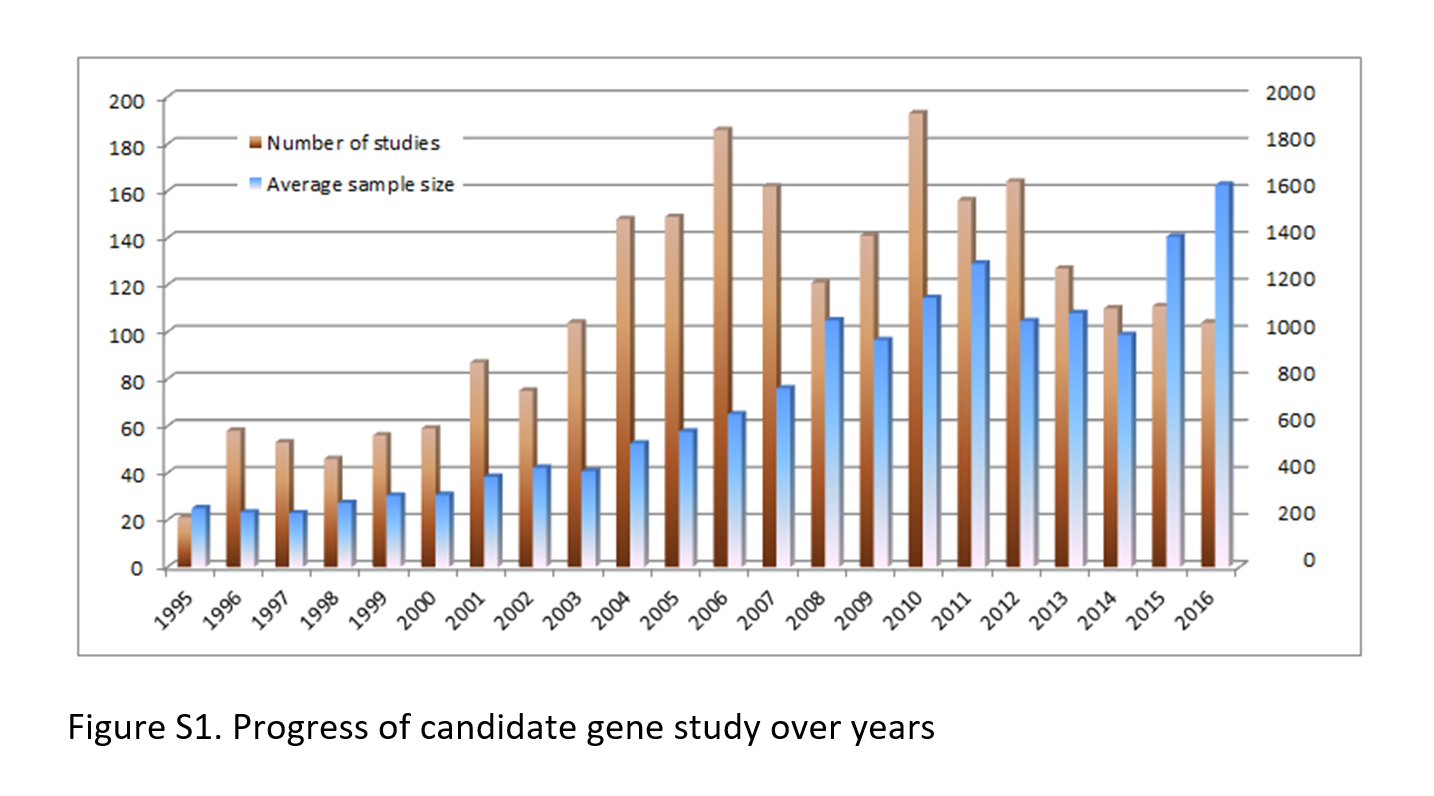

Supplement: Supplementary file 2 — Figure S1 [file 41398_2019_532_MOESM2_ESM.png]

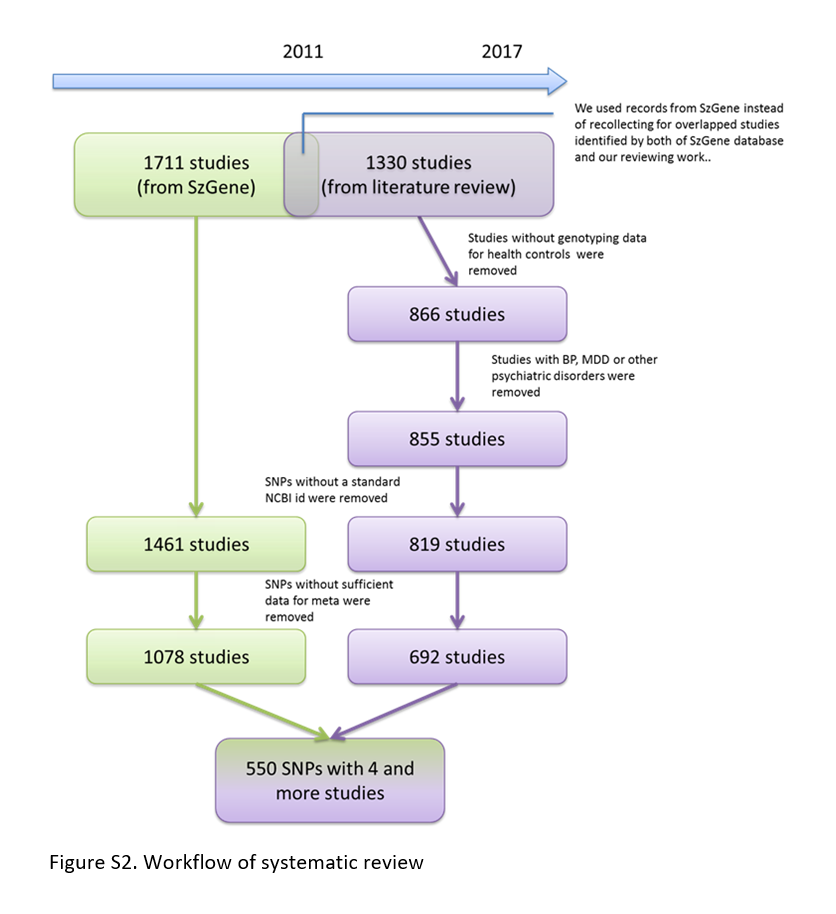

Supplement: Supplementary file 3 — Figure S2 [file 41398_2019_532_MOESM3_ESM.png]

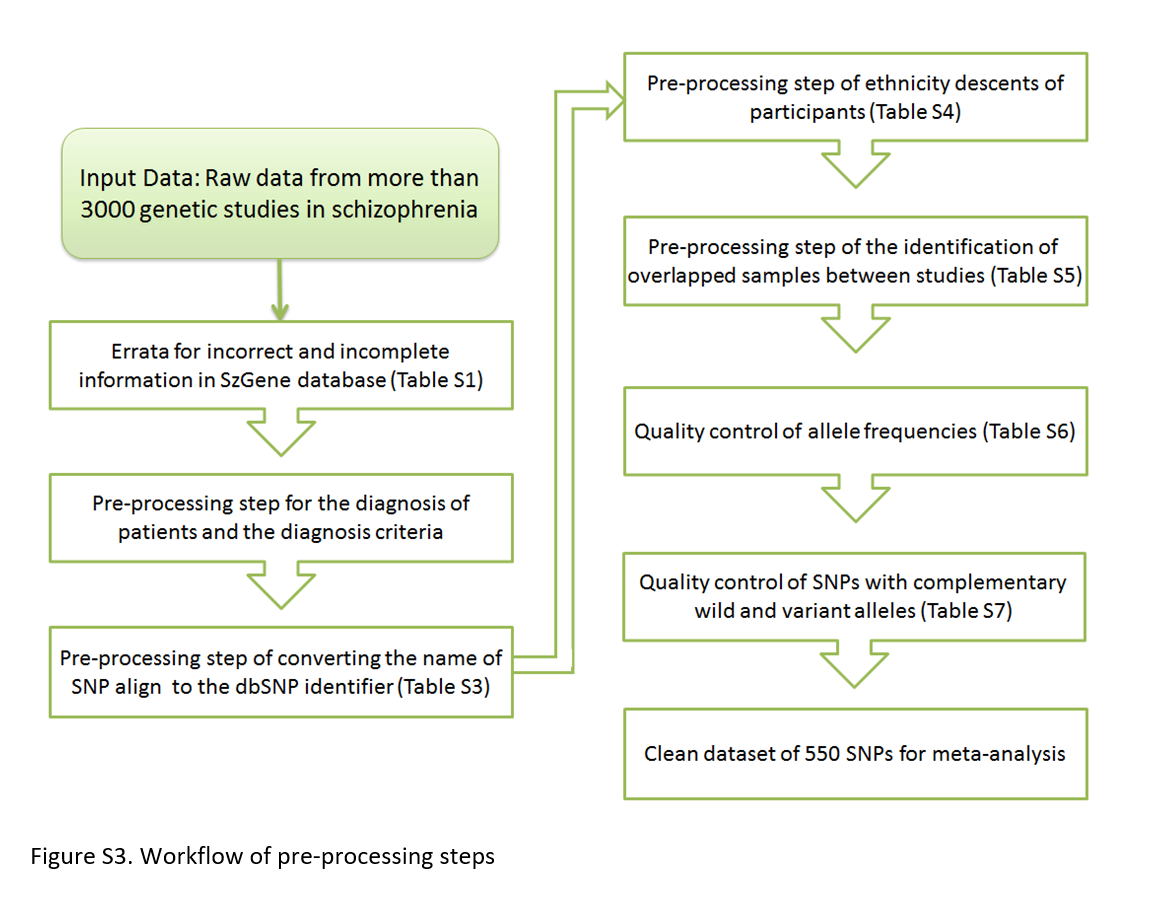

Supplement: Supplementary file 4 — Figure S3 [file 41398_2019_532_MOESM4_ESM.png]
