# Supplementary material for: The schizophrenia genetics knowledgebase: a comprehensive update of findings from candidate gene studies
Source: Transl Psychiatry. 2019 Aug 27;9:205. doi: 10.1038/s41398-019-0532-4 (PMC6711957; doi:10.1038/s41398-019-0532-4)
Supplement: Supplementary file 5 — Supplementary Data S2 [file 41398_2019_532_MOESM5_ESM.pdf]

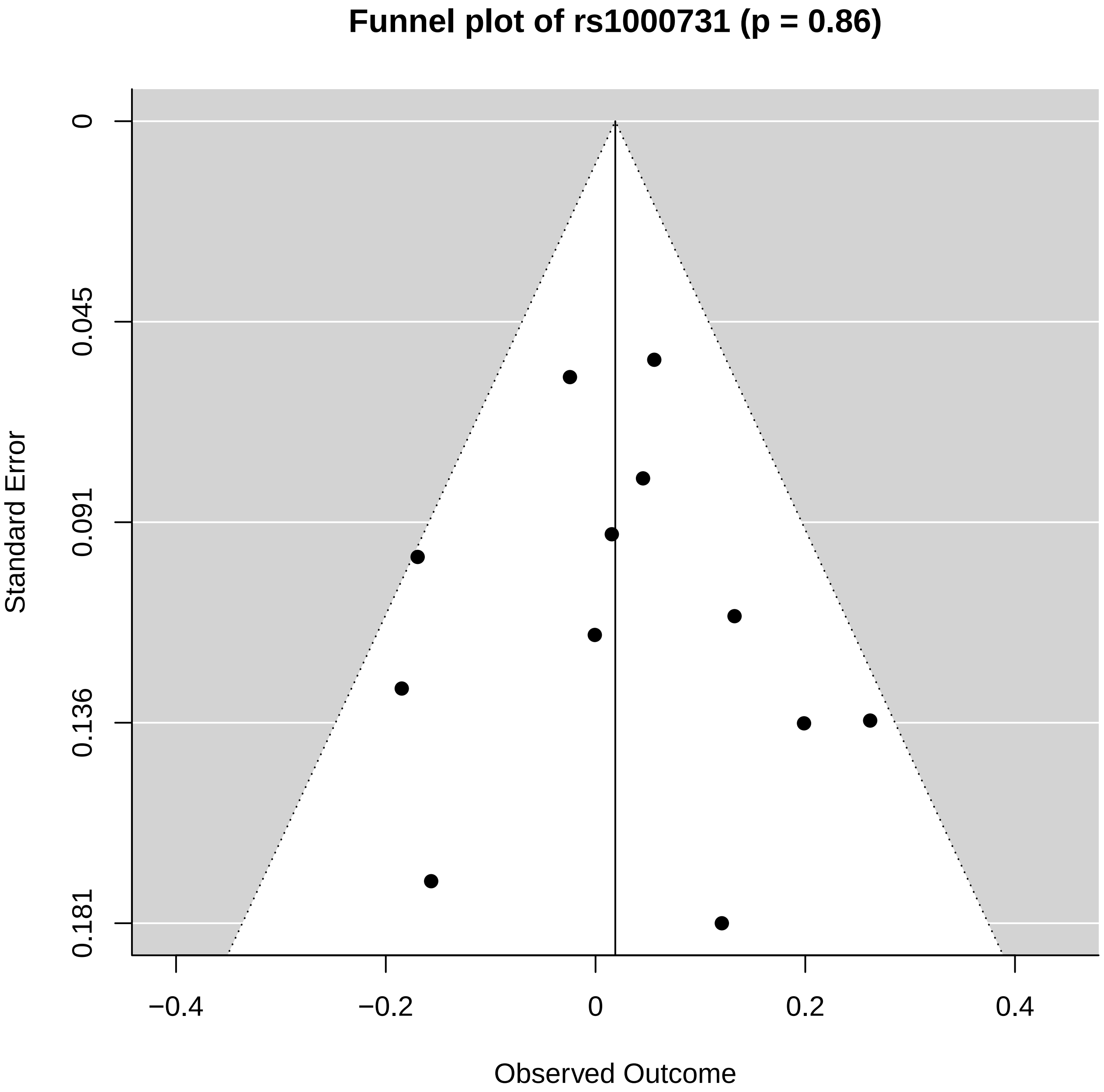
Funnel plot of rs1000731 (p = 0.86)
Standard Error
0
0.045
0.091
0.136
0.181
−0.4
−0.2
0
0.2
0.4
Observed Outcome

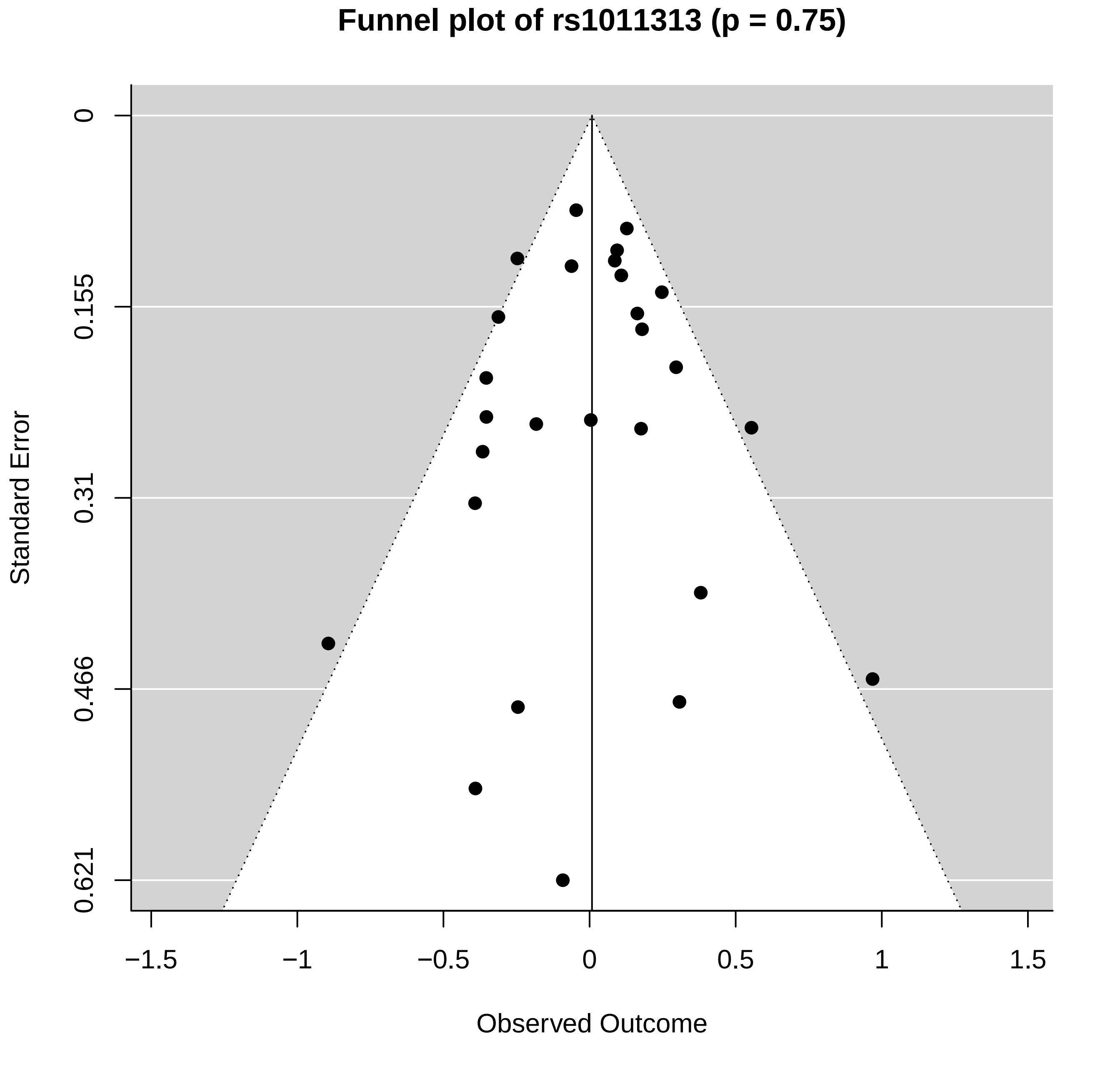
Funnel plot of rs1011313 (p = 0.75)
Standard Error
0
0.155
0.31
0.466
0.621
−1.5
−1
−0.5
0
0.5
1
1.5
Observed Outcome

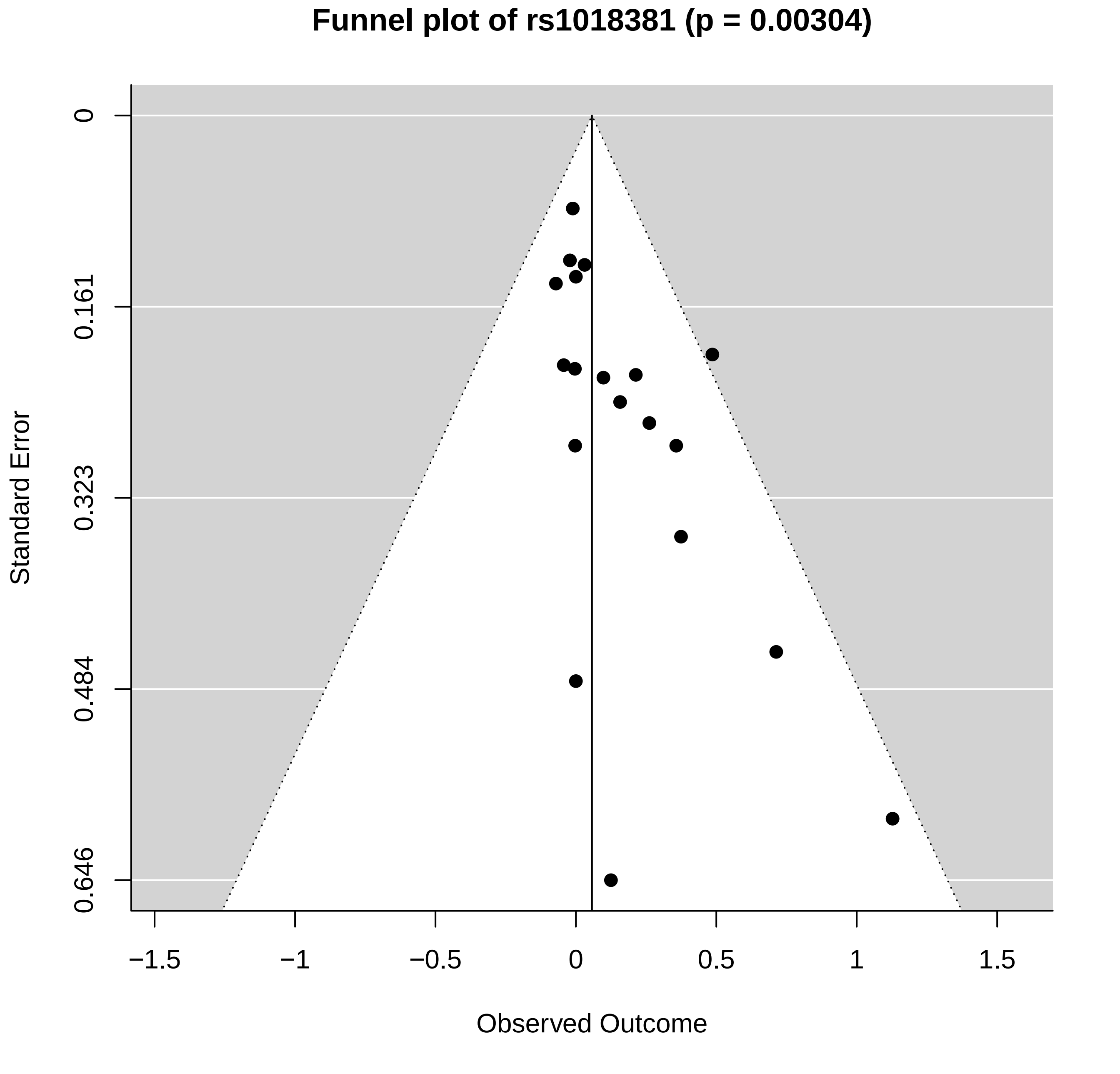

Funnel plot of rs1018381 (p = 0.00304)
Standard Error
0
0.161
0.323
0.484
0.646
−1.5
−1
−0.5
0
0.5
1
1.5
Observed Outcome

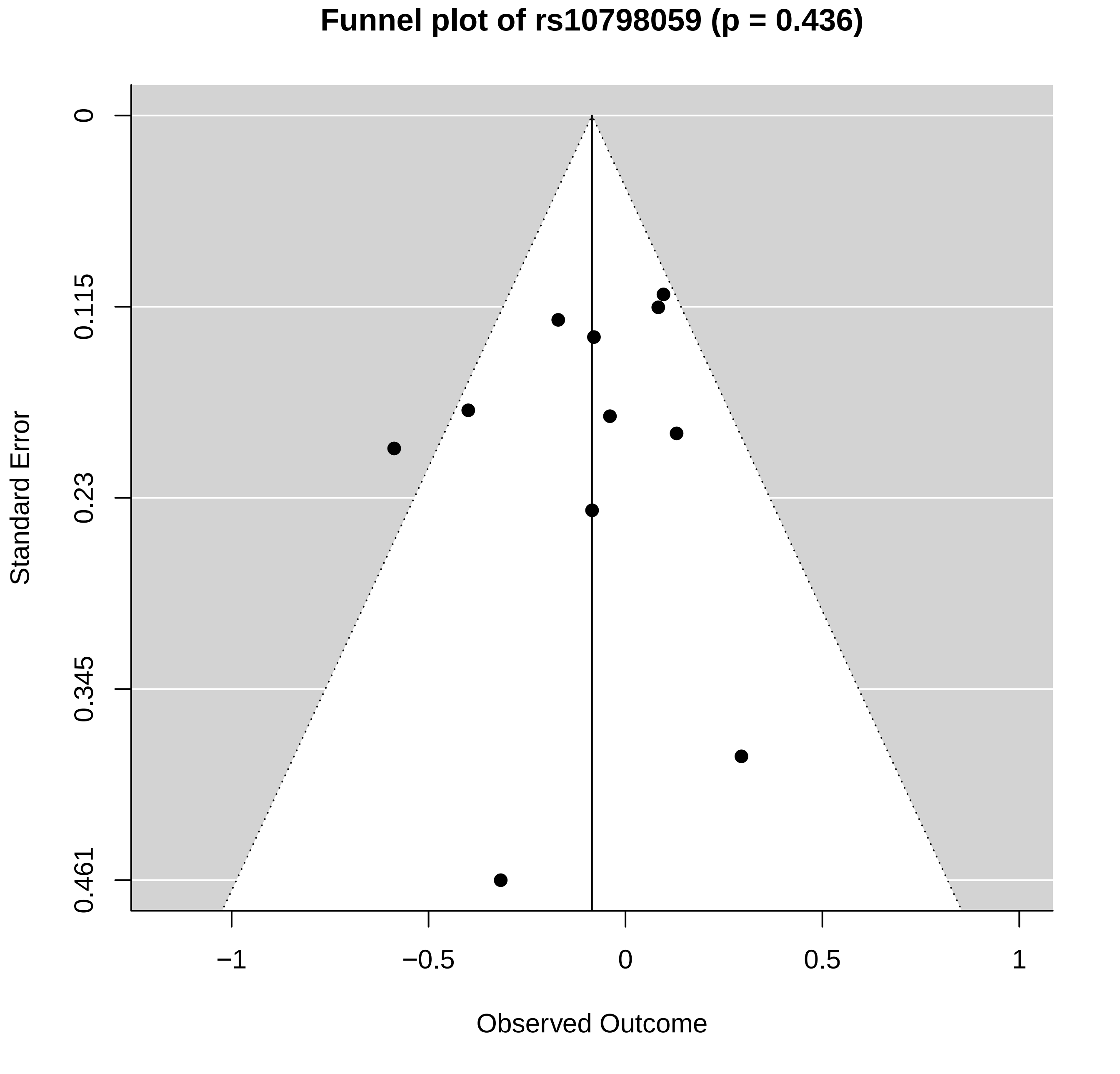

Funnel plot of rs10798059 (p = 0.436)
Standard Error
0
0.115
0.23
0.345
0.461
−1
−0.5
0
0.5
1
Observed Outcome

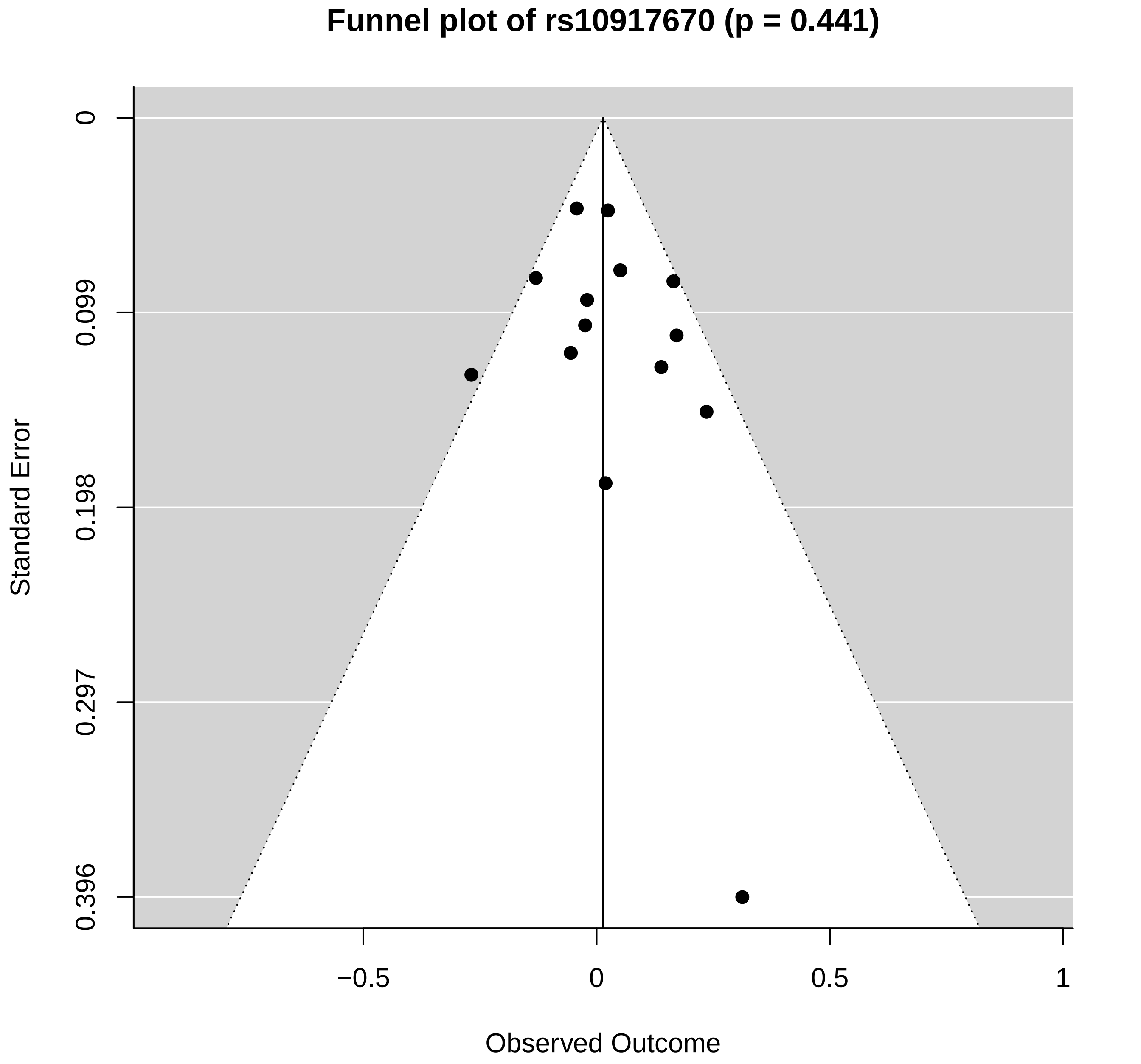
Funnel plot of rs10917670 (p = 0.441)
Standard Error
0
0.099
0.198
0.297
0.396
−0.5
0
0.5
1
Observed Outcome

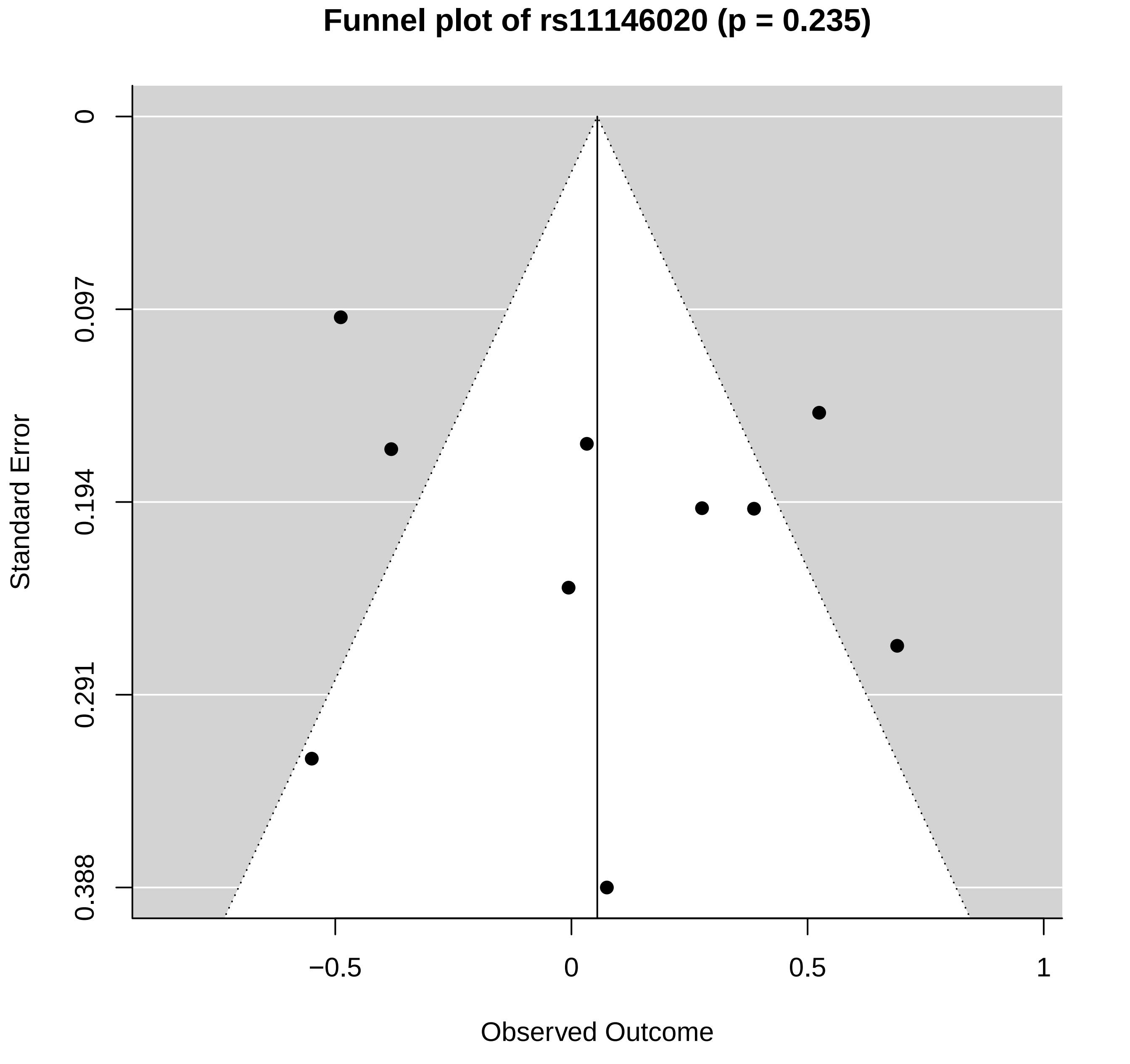
Funnel plot of rs11146020 (p = 0.235)
Standard Error
0
0.097
0.194
0.291
0.388
−0.5
0
0.5
1
Observed Outcome

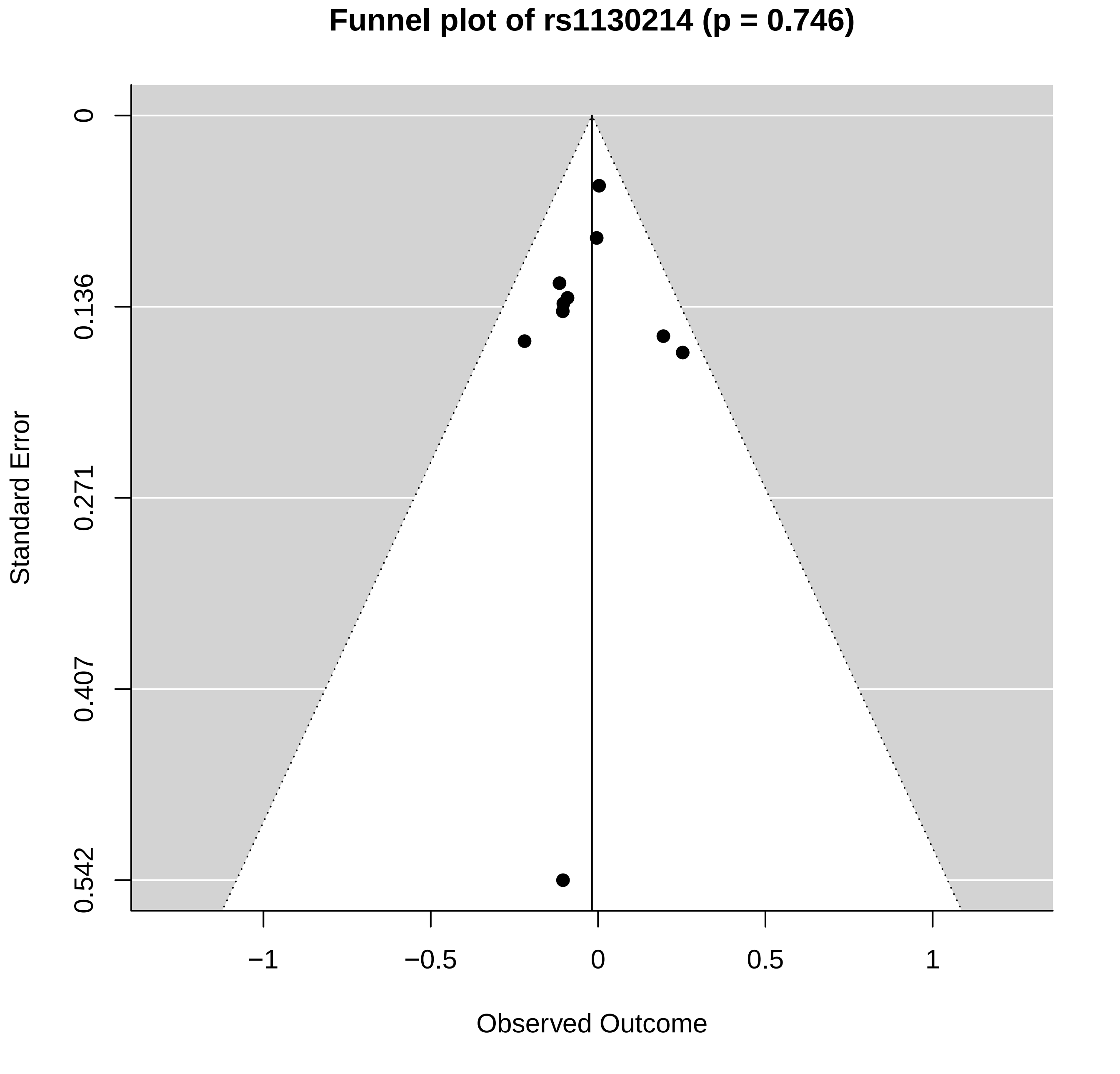

Funnel plot of rs1130214 (p = 0.746)
Standard Error
0
0.136
0.271
0.407
0.542
−1
−0.5
0
0.5
1
Observed Outcome

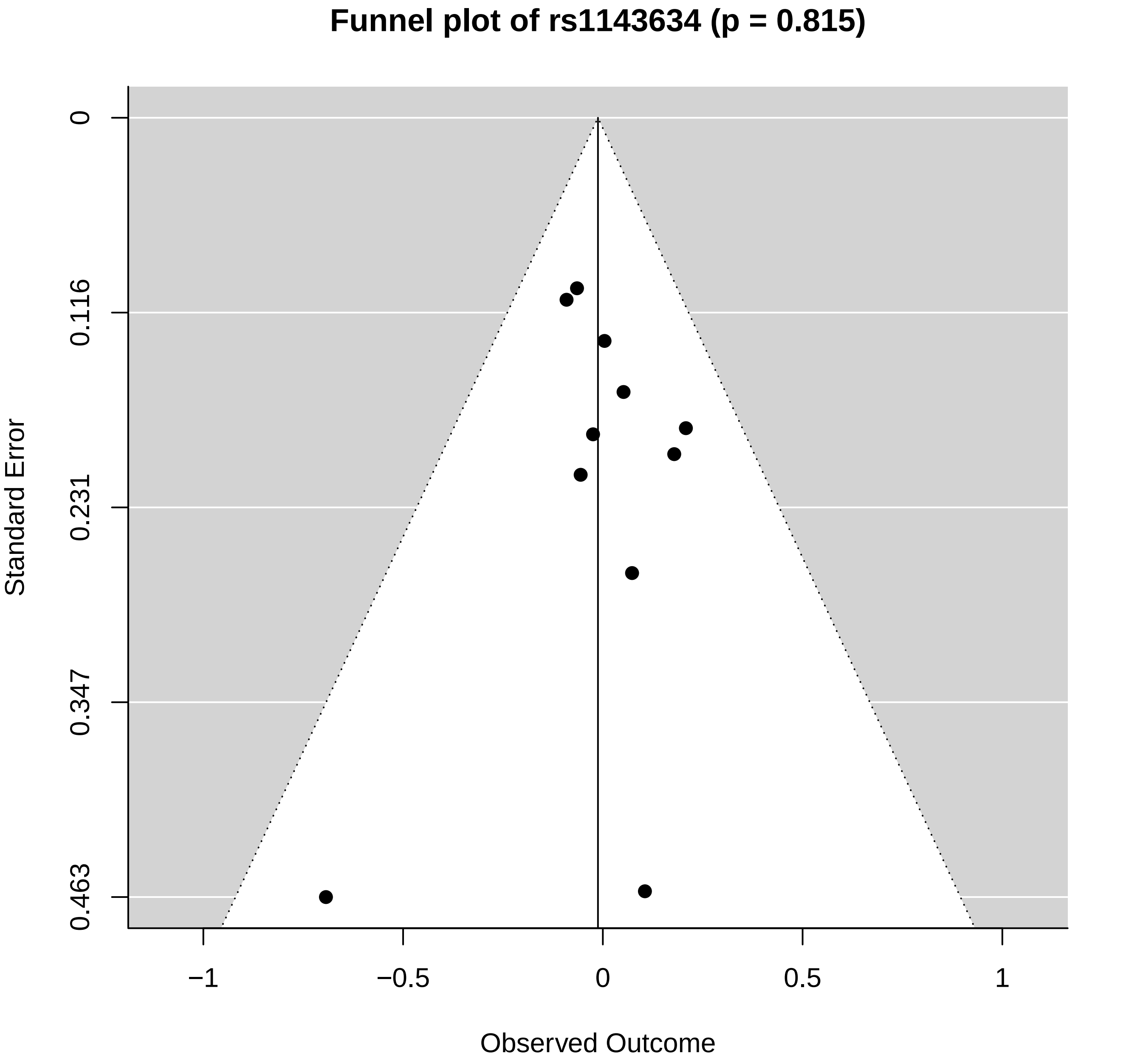

Funnel plot of rs1143634 (p = 0.815)
Standard Error
0
0.116
0.231
0.347
0.463
−1
−0.5
0
0.5
1
Observed Outcome

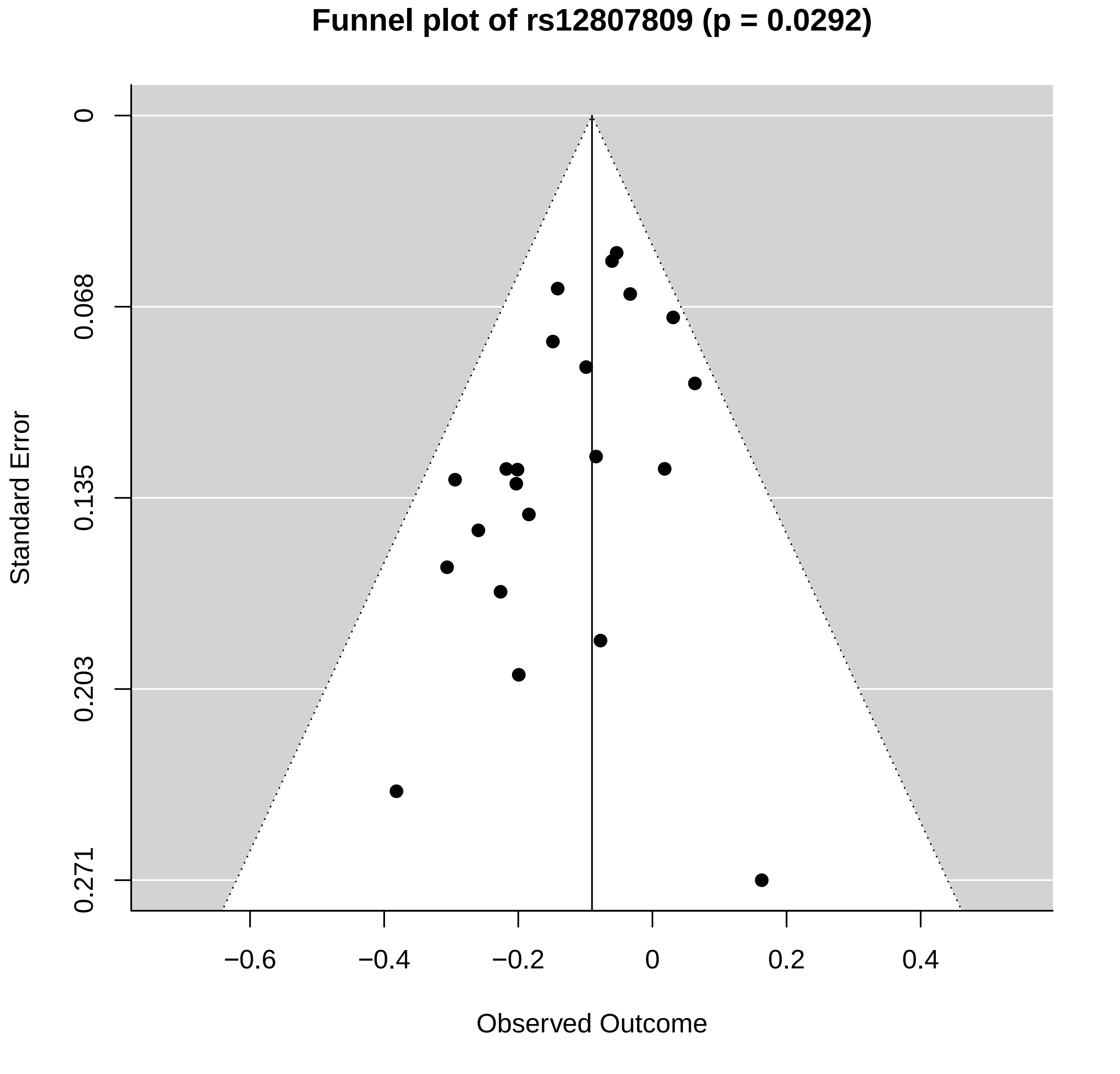

Funnel plot of rs12807809 (p = 0.0292)
Standard Error
0
0.068
0.135
0.203
0.271
−0.6
−0.4
−0.2
0
0.2
0.4
Observed Outcome

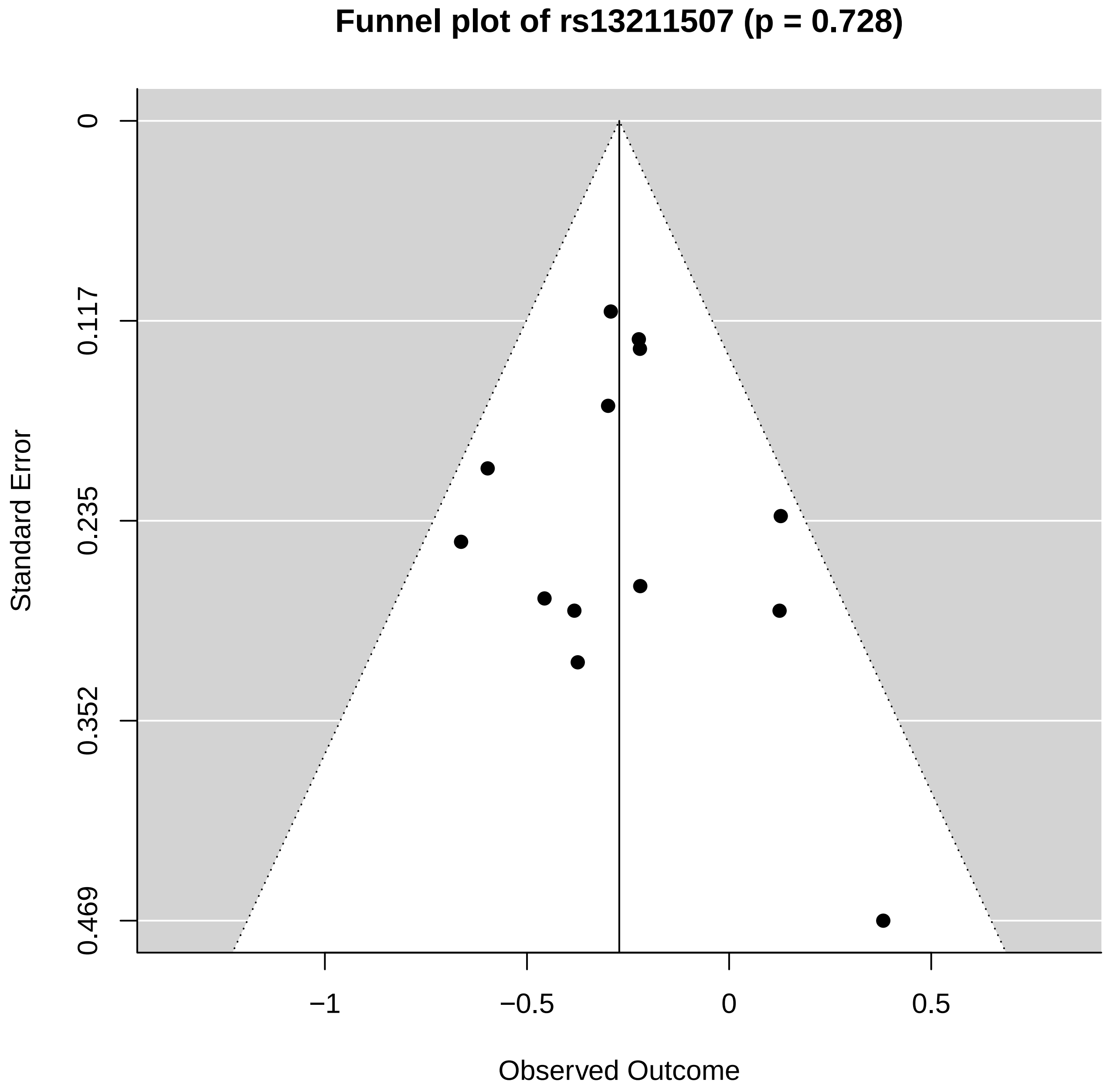
Funnel plot of rs13211507 (p = 0.728)
Standard Error
0
0.117
0.235
0.352
0.469
−1
−0.5
0
0.5
Observed Outcome

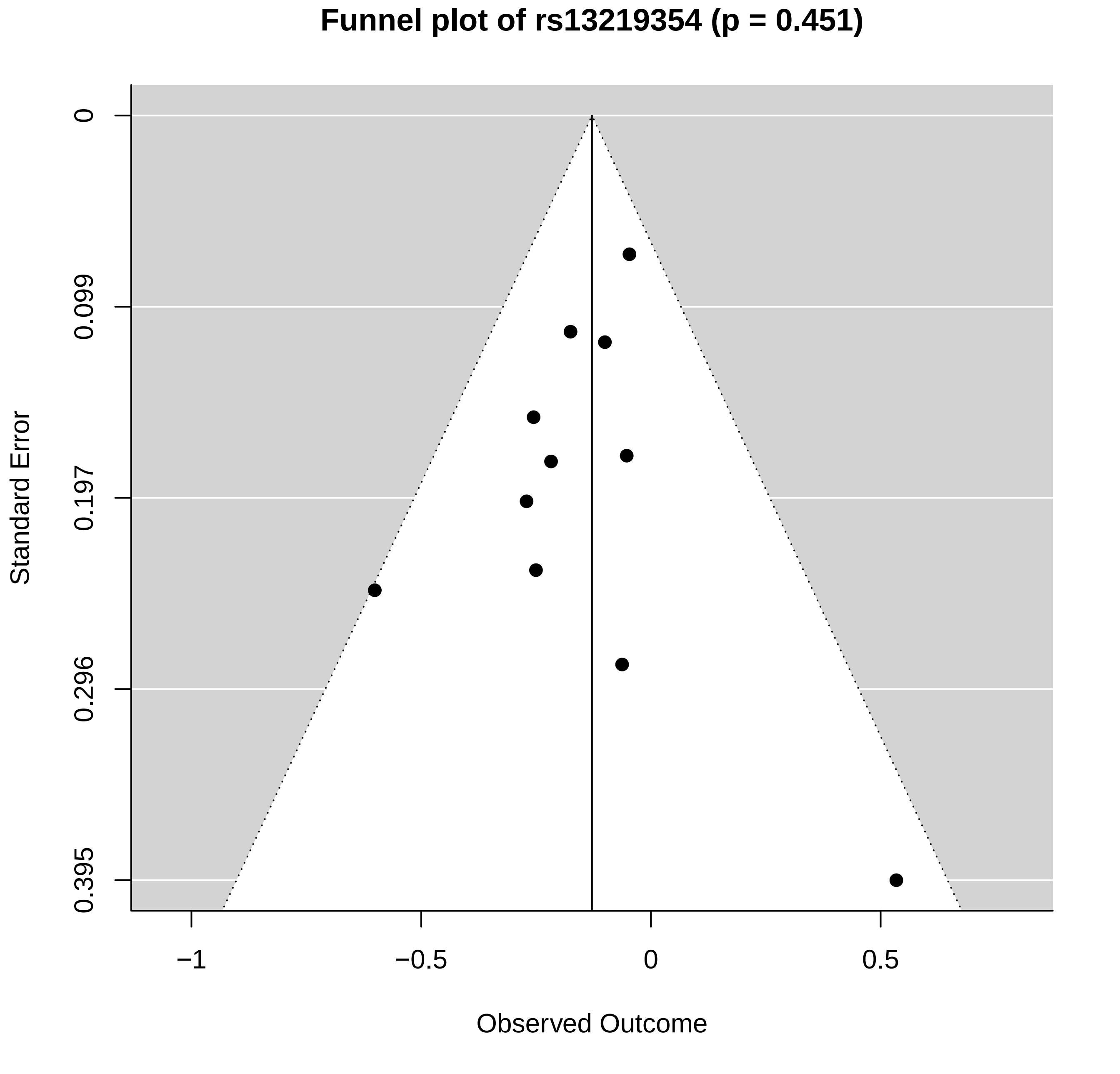
Funnel plot of rs13219354 (p = 0.451)
Standard Error
0
0.099
0.197
0.296
0.395
−1
−0.5
0
0.5
Observed Outcome

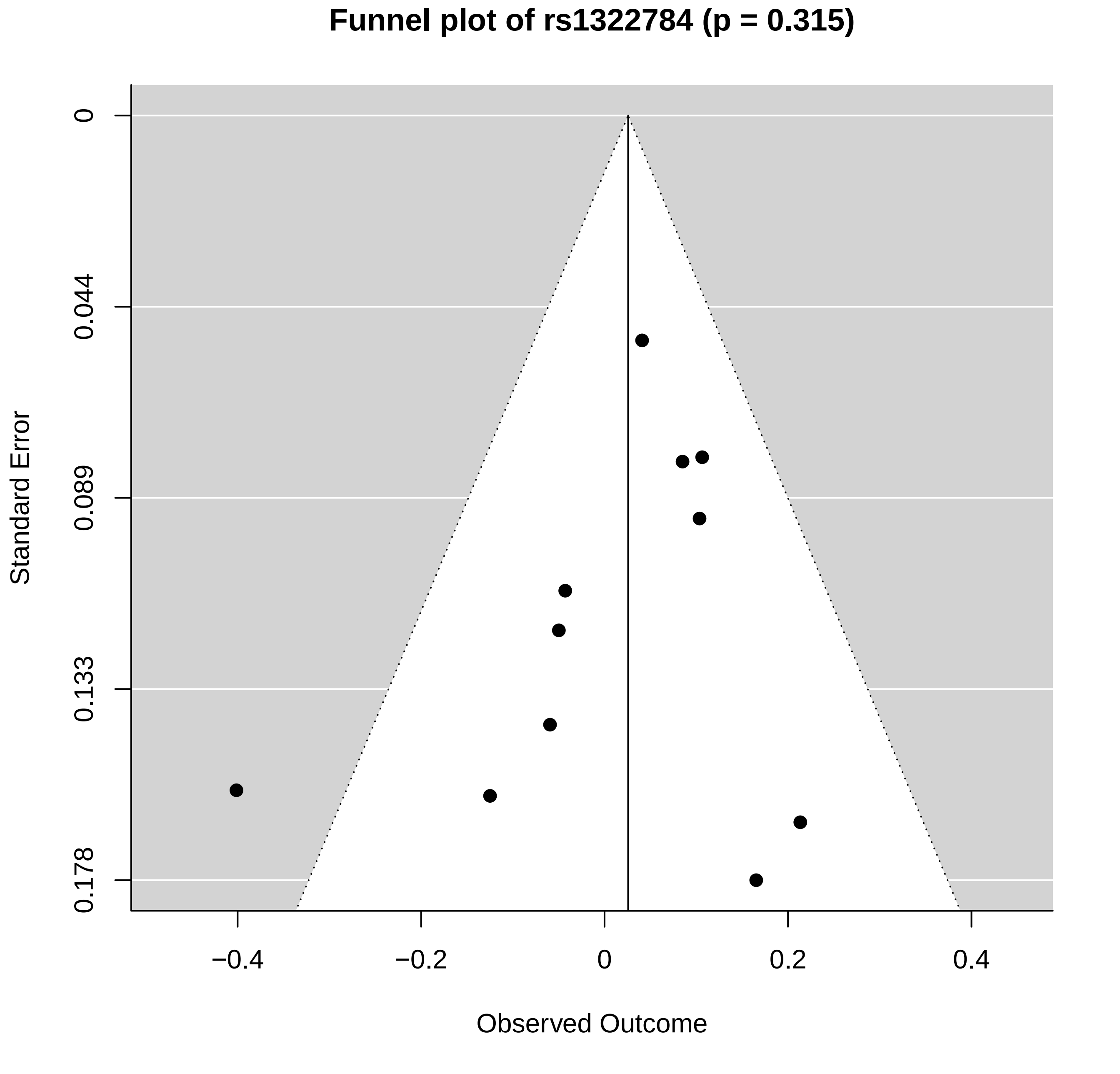

Funnel plot of rs1322784 (p = 0.315)
Standard Error
0
0.044
0.089
0.133
0.178
−0.4
−0.2
0
0.2
0.4
Observed Outcome

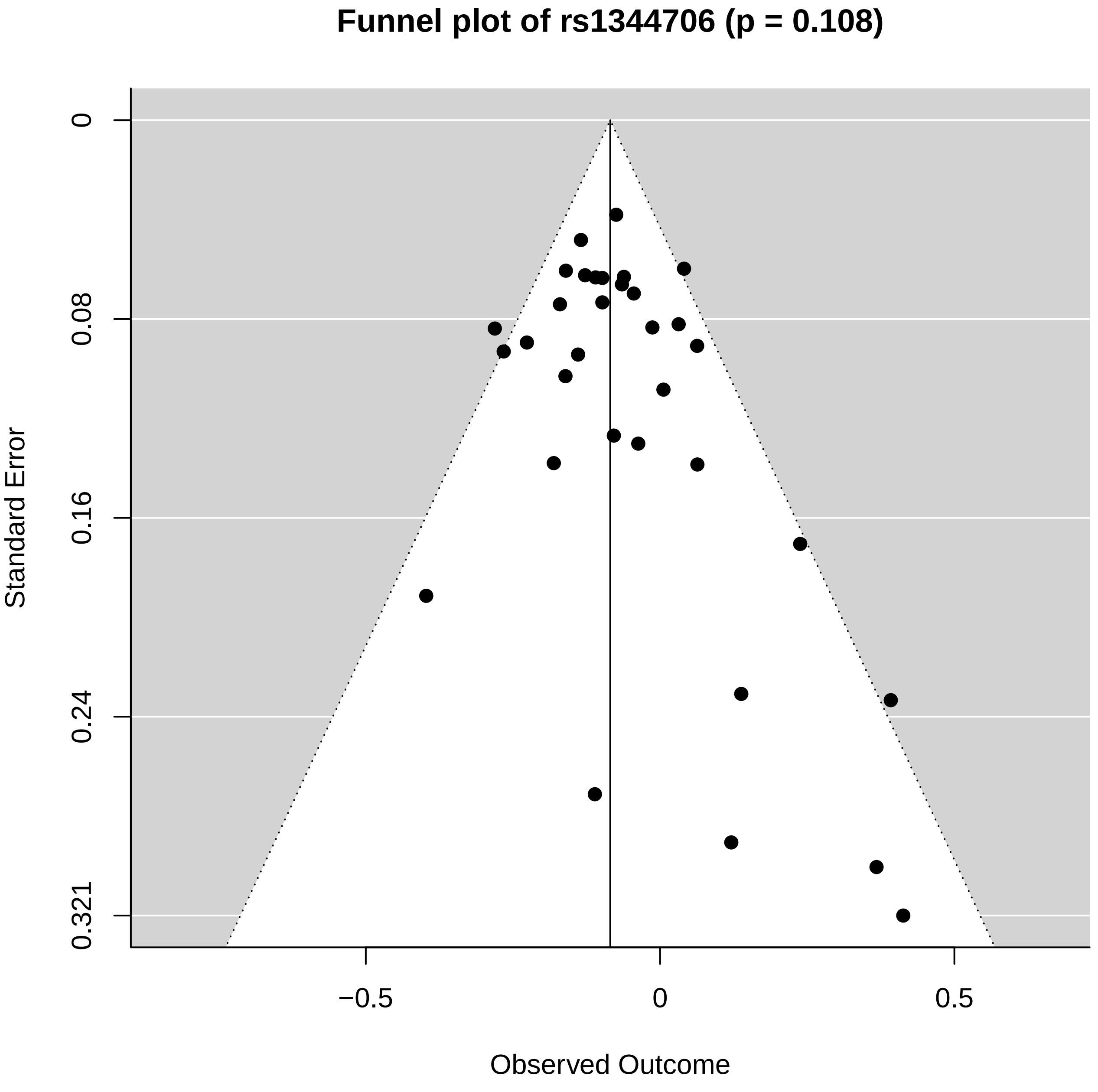
Funnel plot of rs1344706 (p = 0.108)
Standard Error
0
0.08
0.16
0.24
0.321
−0.5
0
0.5
Observed Outcome

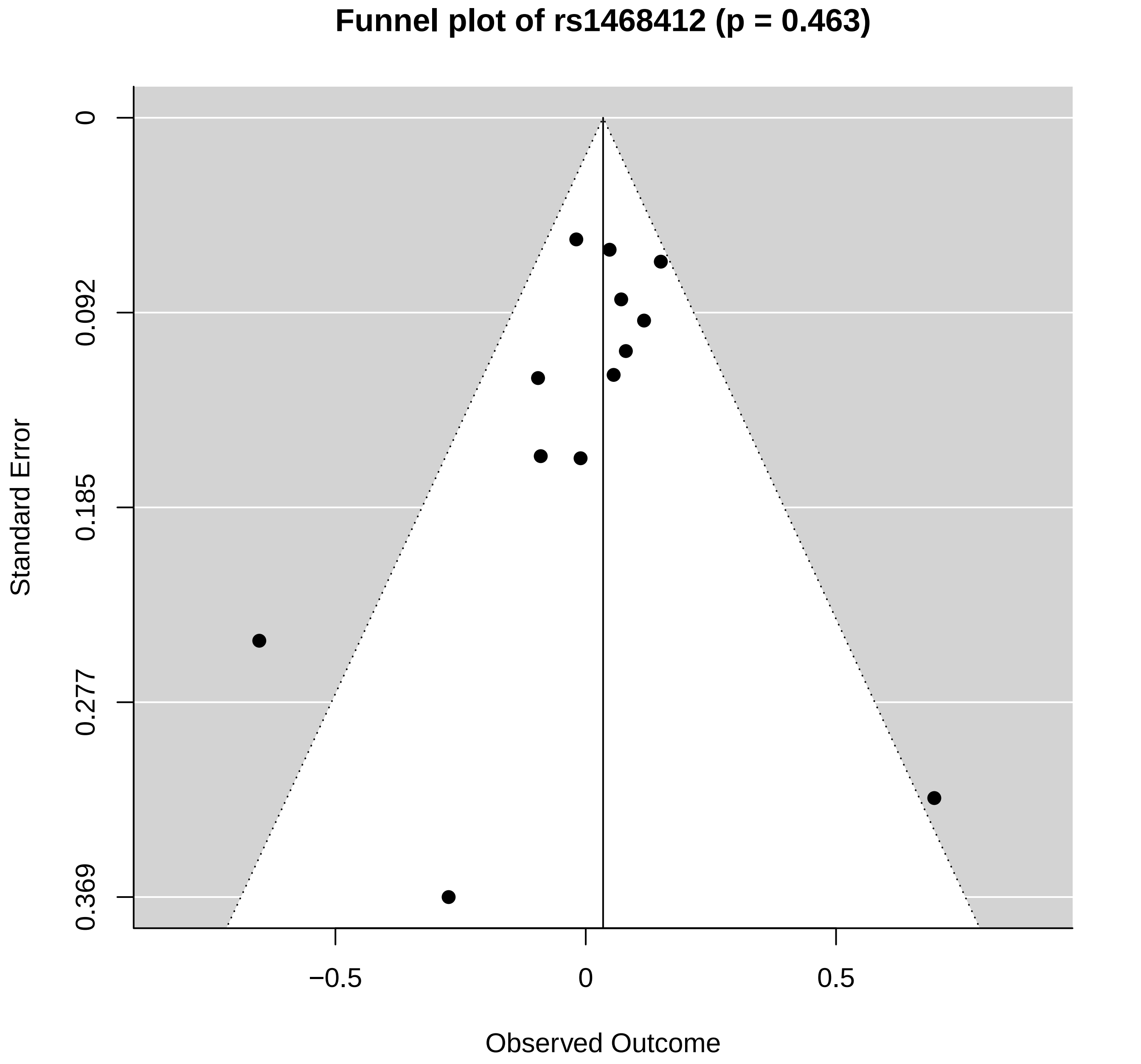

Funnel plot of rs1468412 (p = 0.463)
Standard Error
0
0.092
0.185
0.277
0.369
−0.5
0
0.5
Observed Outcome

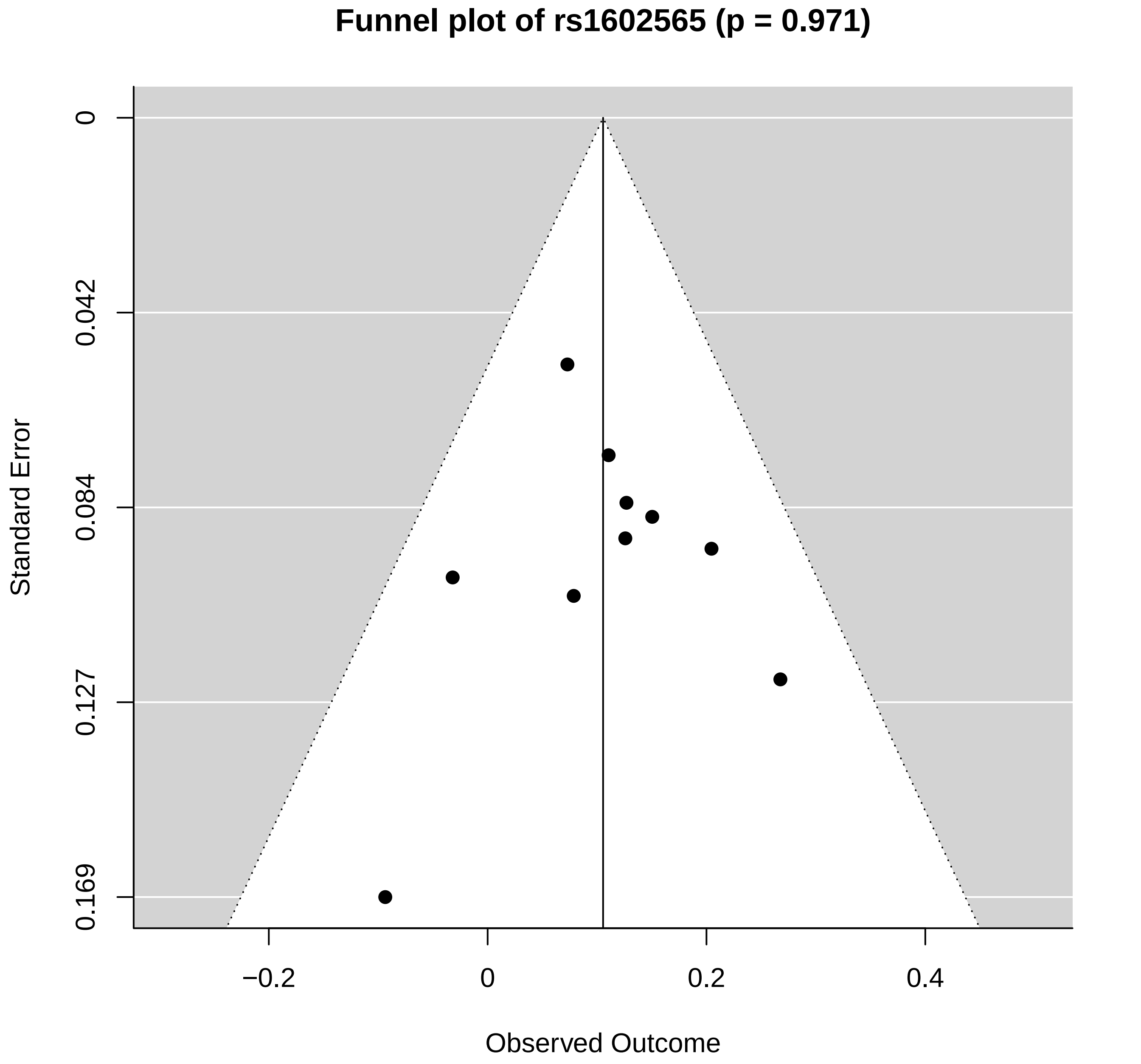
Funnel plot of rs1602565 (p = 0.971)
Standard Error
0
0.042
0.084
0.127
0.169
−0.2
0
0.2
0.4
Observed Outcome

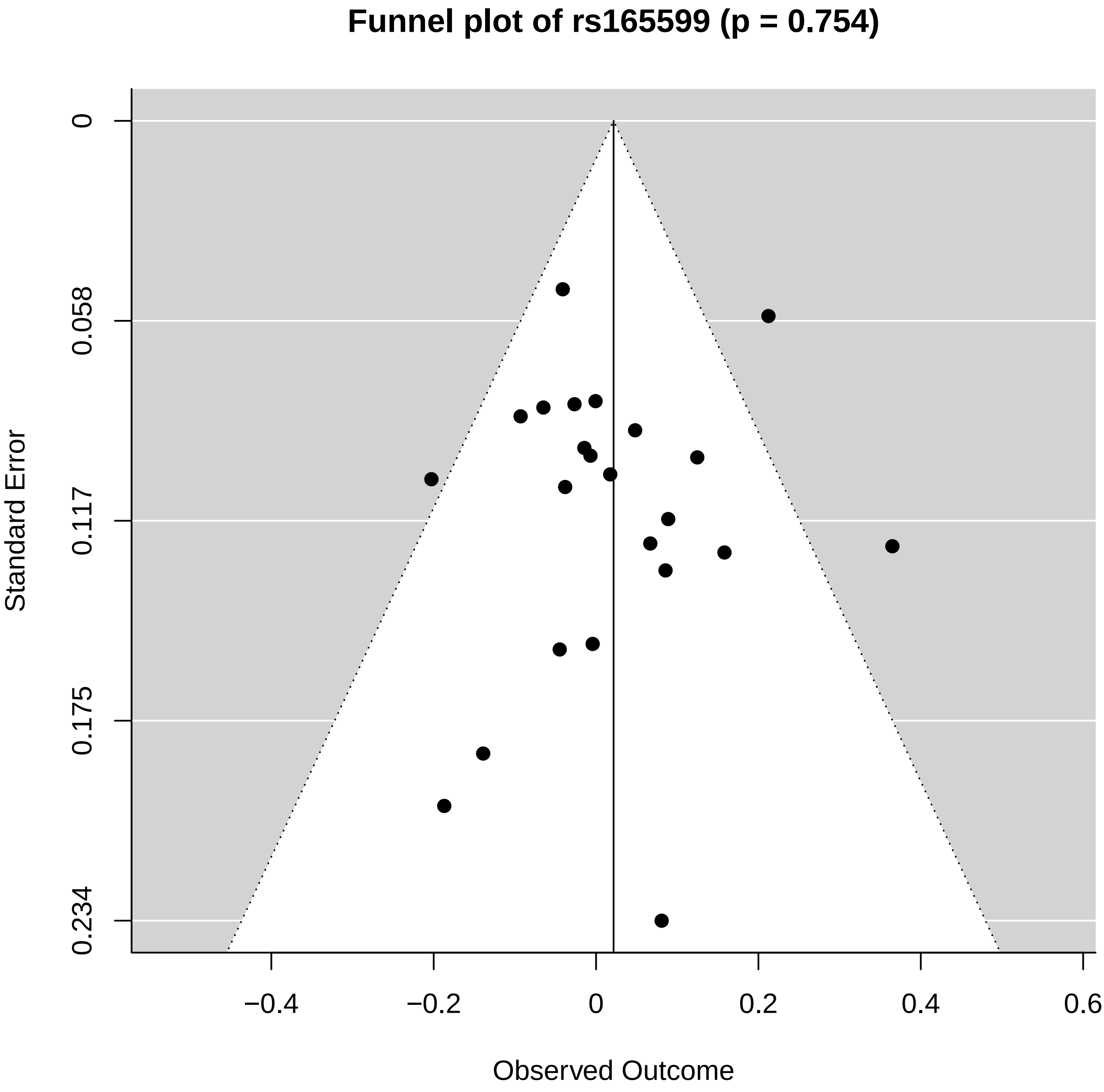
Funnel plot of rs165599 (p = 0.754)
Standard Error
0
0.058
0.117
0.175
0.234
−0.4
−0.2
0
0.2
0.4
0.6
Observed Outcome

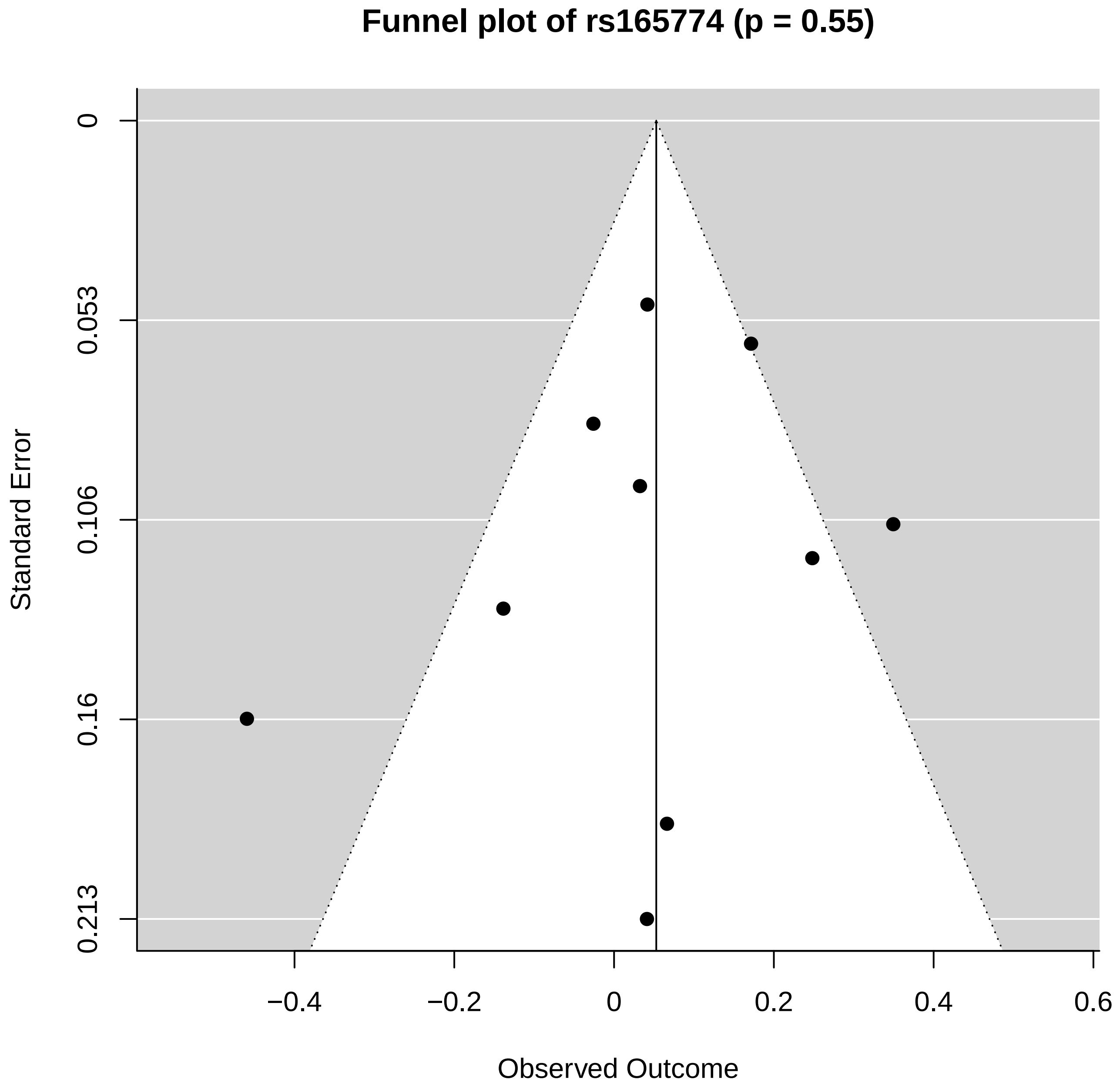
Funnel plot of rs165774 (p = 0.55)
Standard Error
0
0.053
0.106
0.16
0.213
−0.4
−0.2
0
0.2
0.4
0.6
Observed Outcome

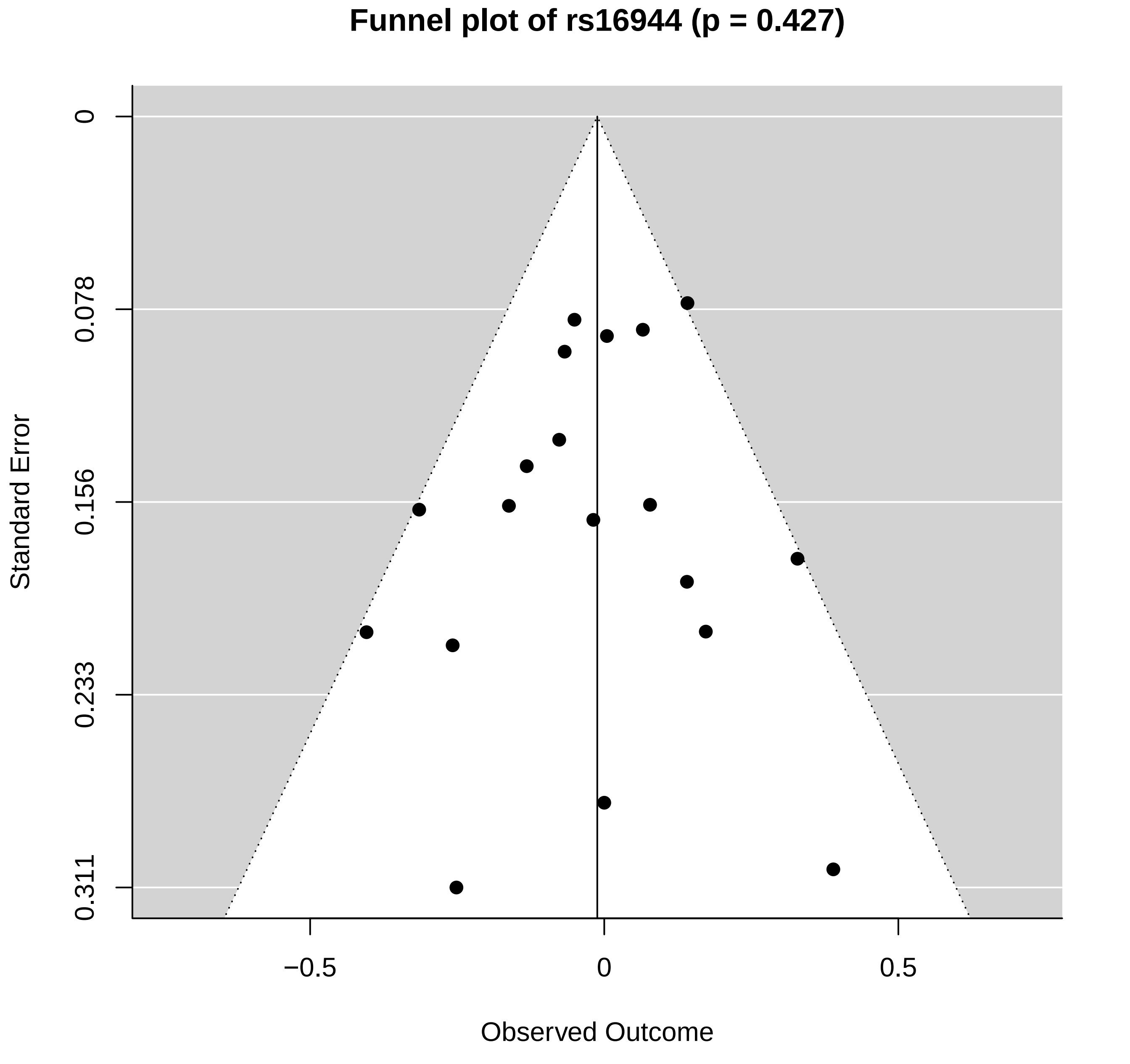

Funnel plot of rs16944 (p = 0.427)
Standard Error
0
0.078
0.156
0.233
0.311
−0.5
0
0.5
Observed Outcome

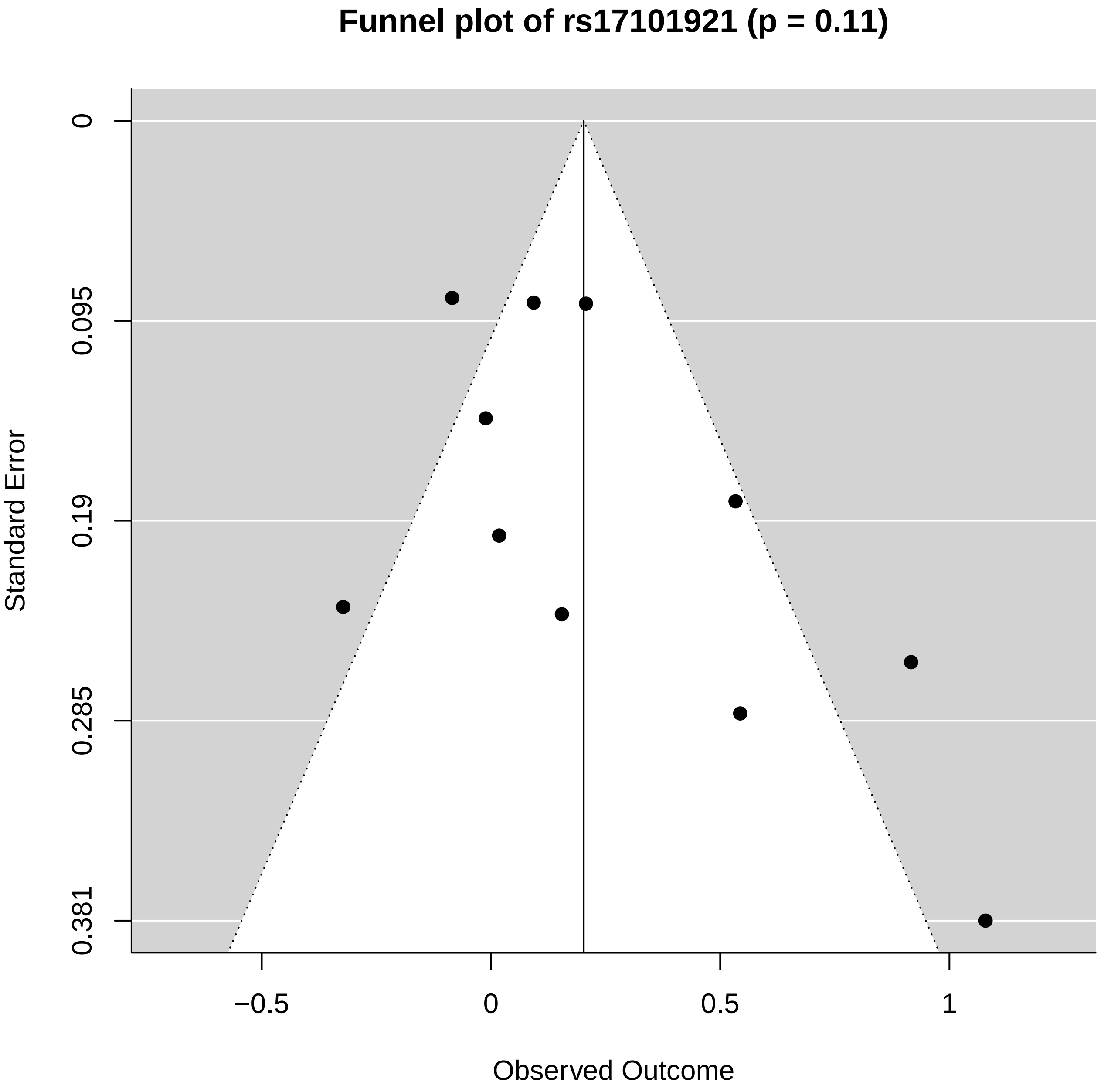
Funnel plot of rs17101921 (p = 0.11)
Standard Error
0
0.095
0.19
0.285
0.381
−0.5
0
0.5
1
Observed Outcome

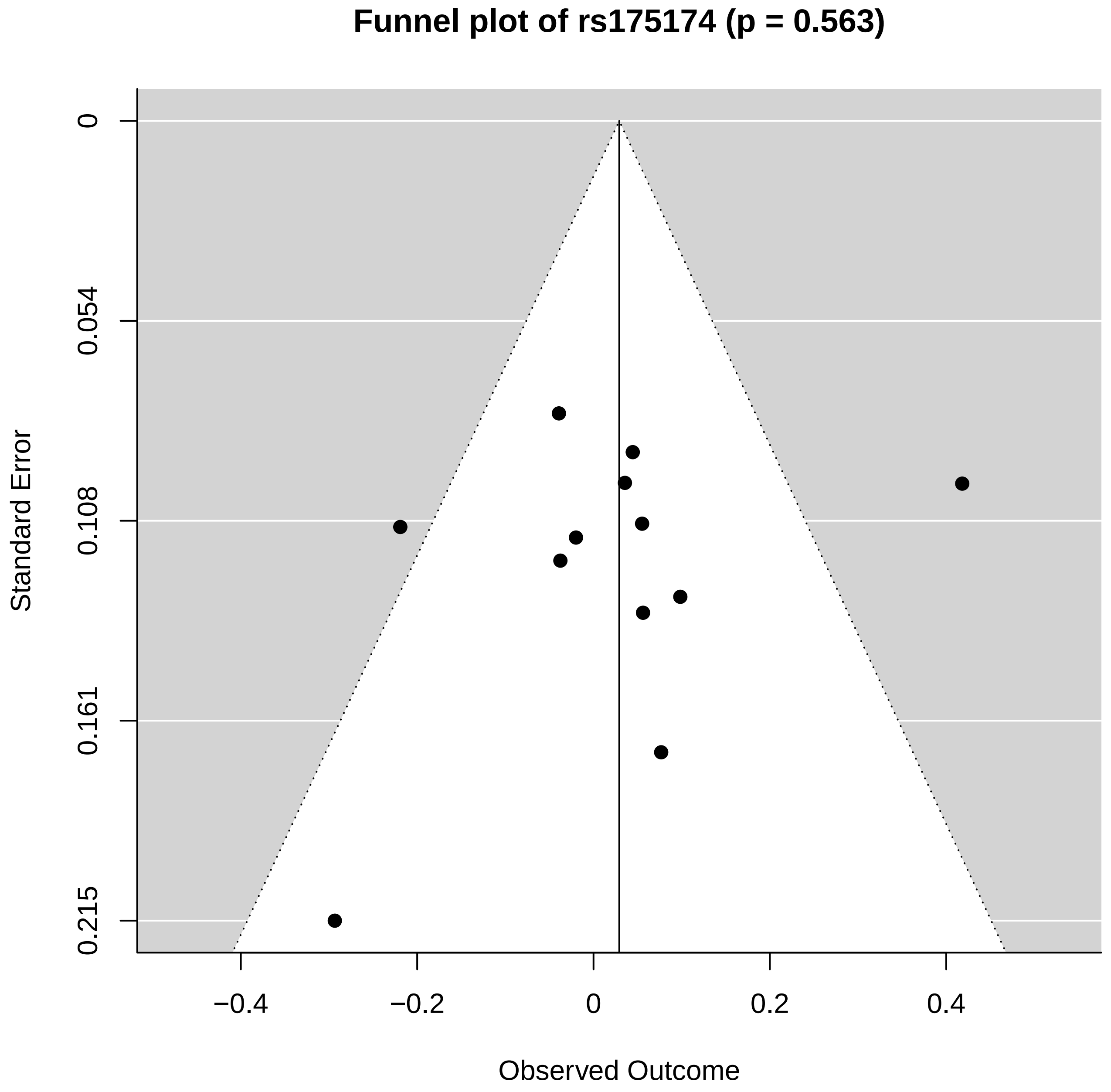

Funnel plot of rs175174 (p = 0.563)
Standard Error
0
0.054
0.108
0.161
0.215
−0.4
−0.2
0
0.2
0.4
Observed Outcome

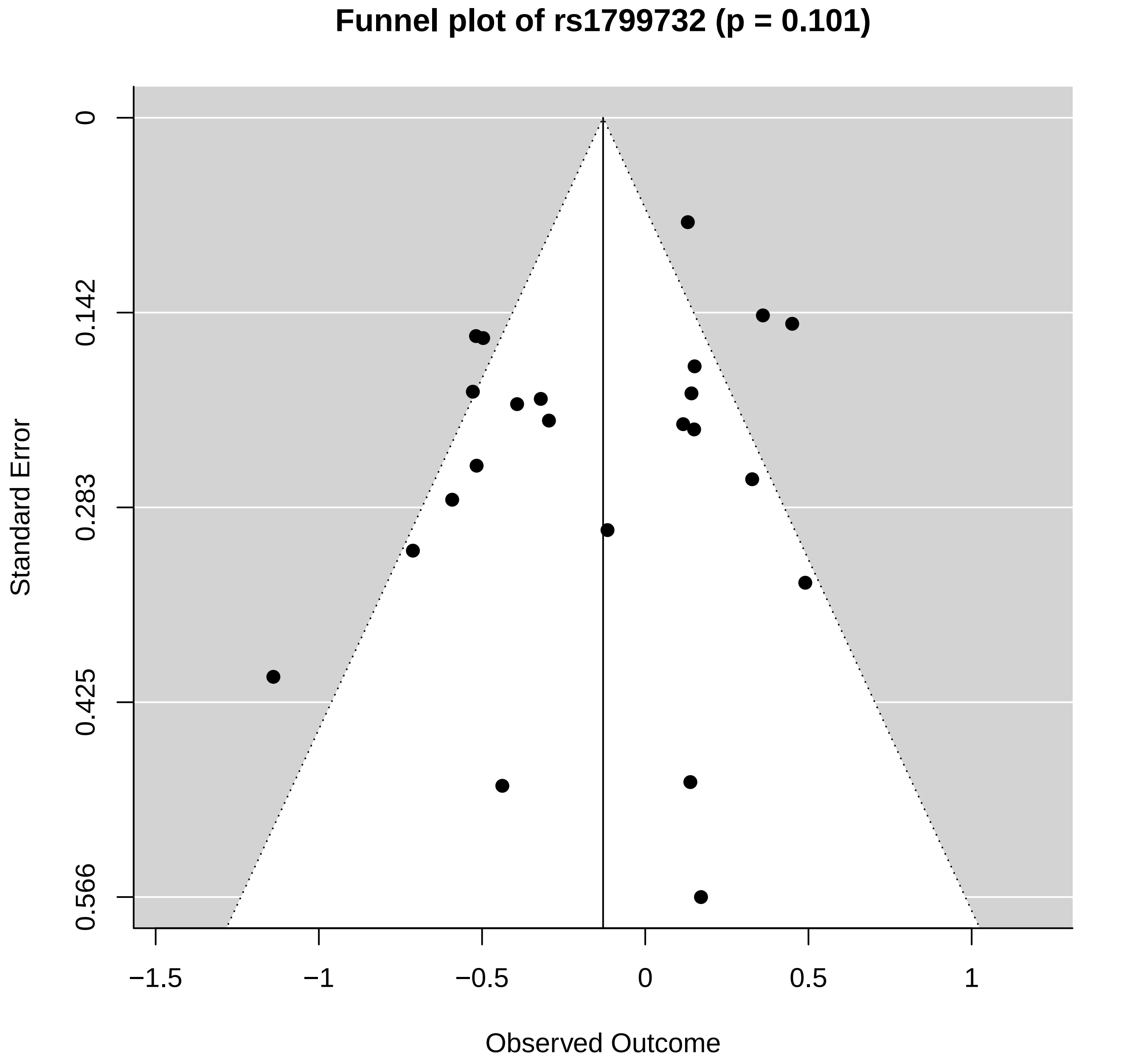
Funnel plot of rs1799732 (p = 0.101)
Standard Error
0
0.142
0.283
0.425
0.566
−1.5
−1
−0.5
0
0.5
1
Observed Outcome

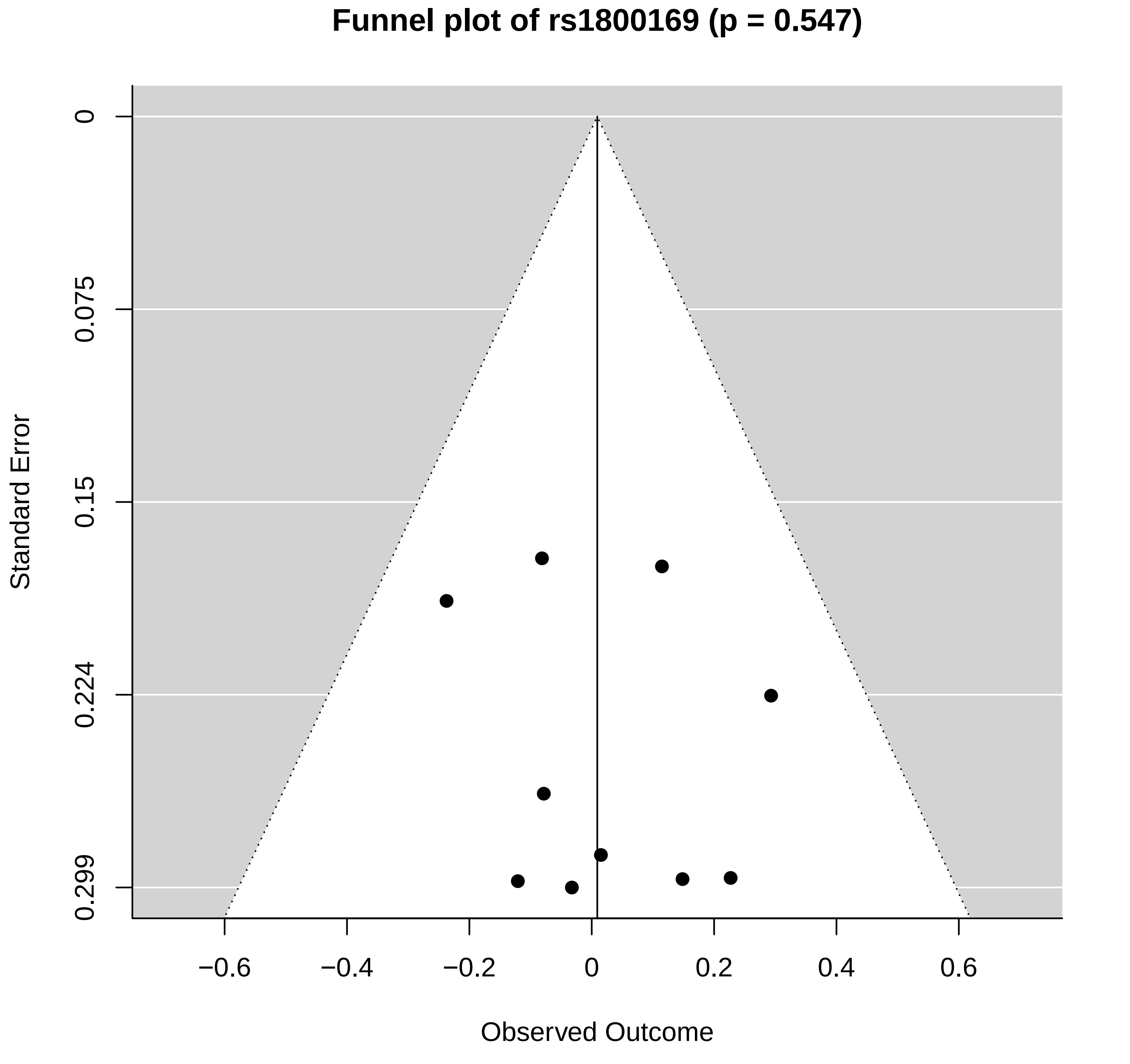

Funnel plot of rs1800169 (p = 0.547)
Standard Error
0
0.075
0.15
0.224
0.299
Observed Outcome
−0.6
−0.4
−0.2
0
0.2
0.4
0.6

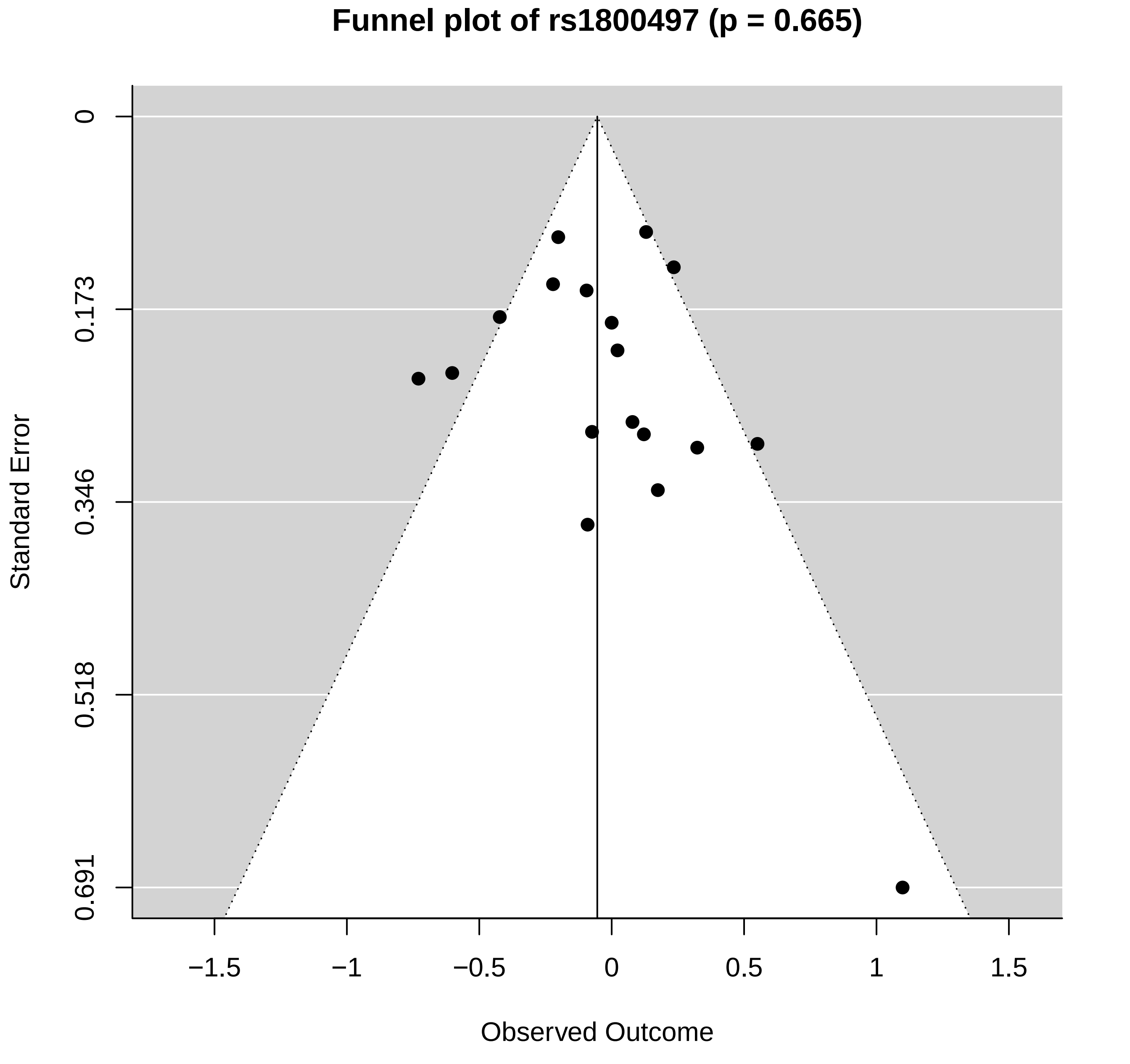
Funnel plot of rs1800497 (p = 0.665)
Standard Error
0
0.173
0.346
0.518
0.691
−1.5
−1
−0.5
0
0.5
1
1.5
Observed Outcome

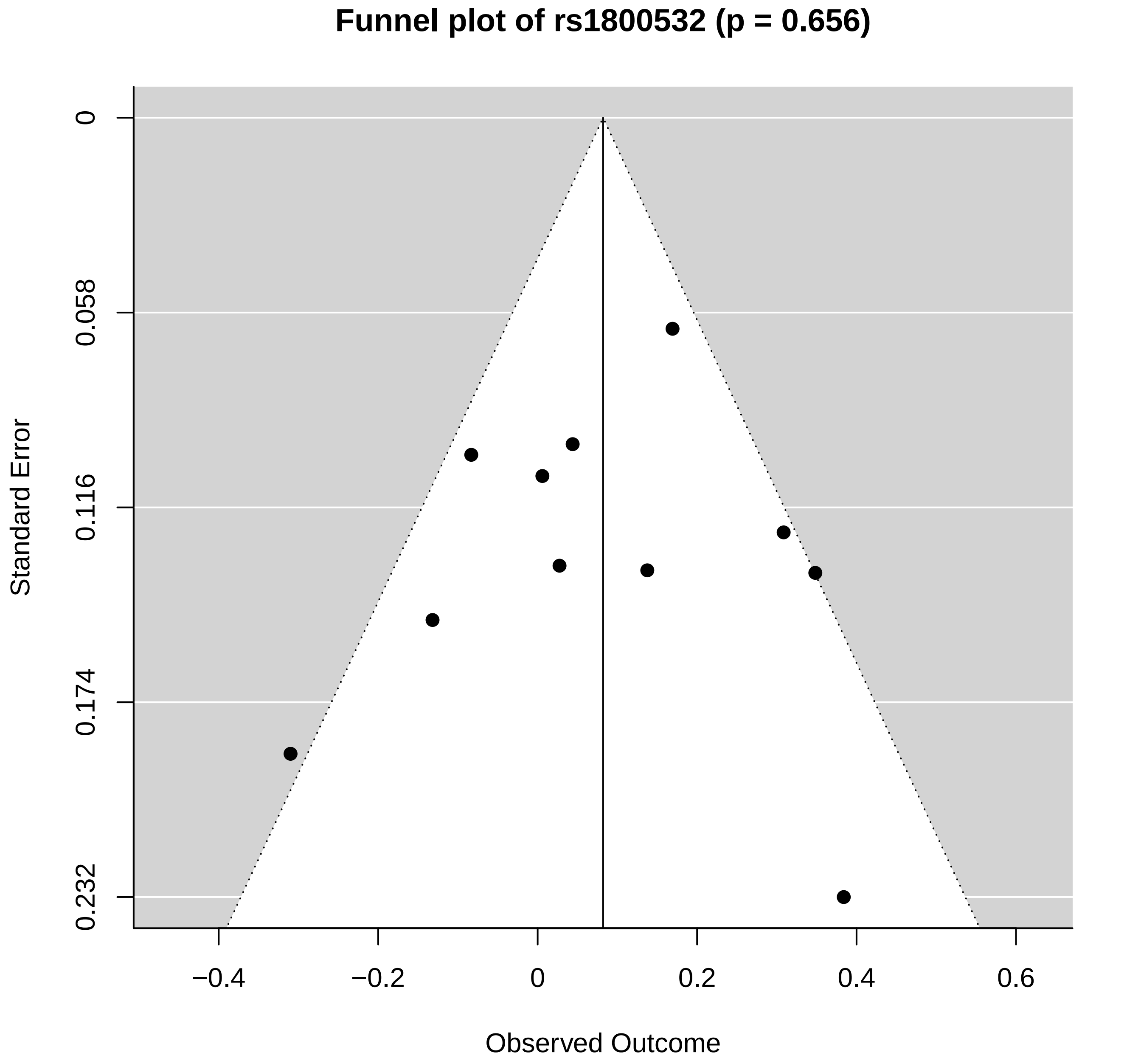

Funnel plot of rs1800532 (p = 0.656)
Standard Error
0
0.058
0.116
0.174
0.232
−0.4
−0.2
0
0.2
0.4
0.6
Observed Outcome

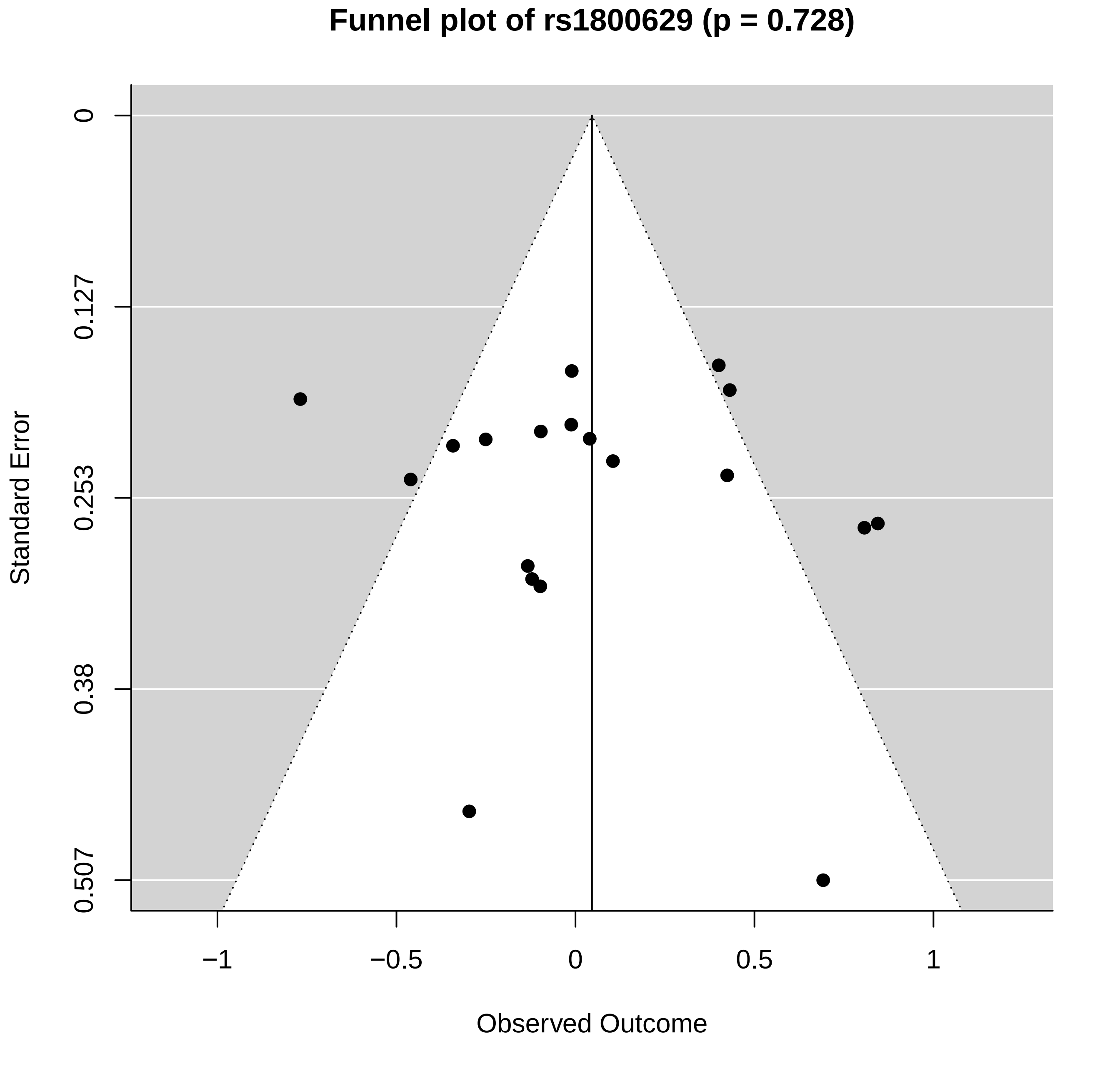
Funnel plot of rs1800629 (p = 0.728)
Standard Error
0
0.127
0.253
0.38
0.507
−1
−0.5
0
0.5
1
Observed Outcome

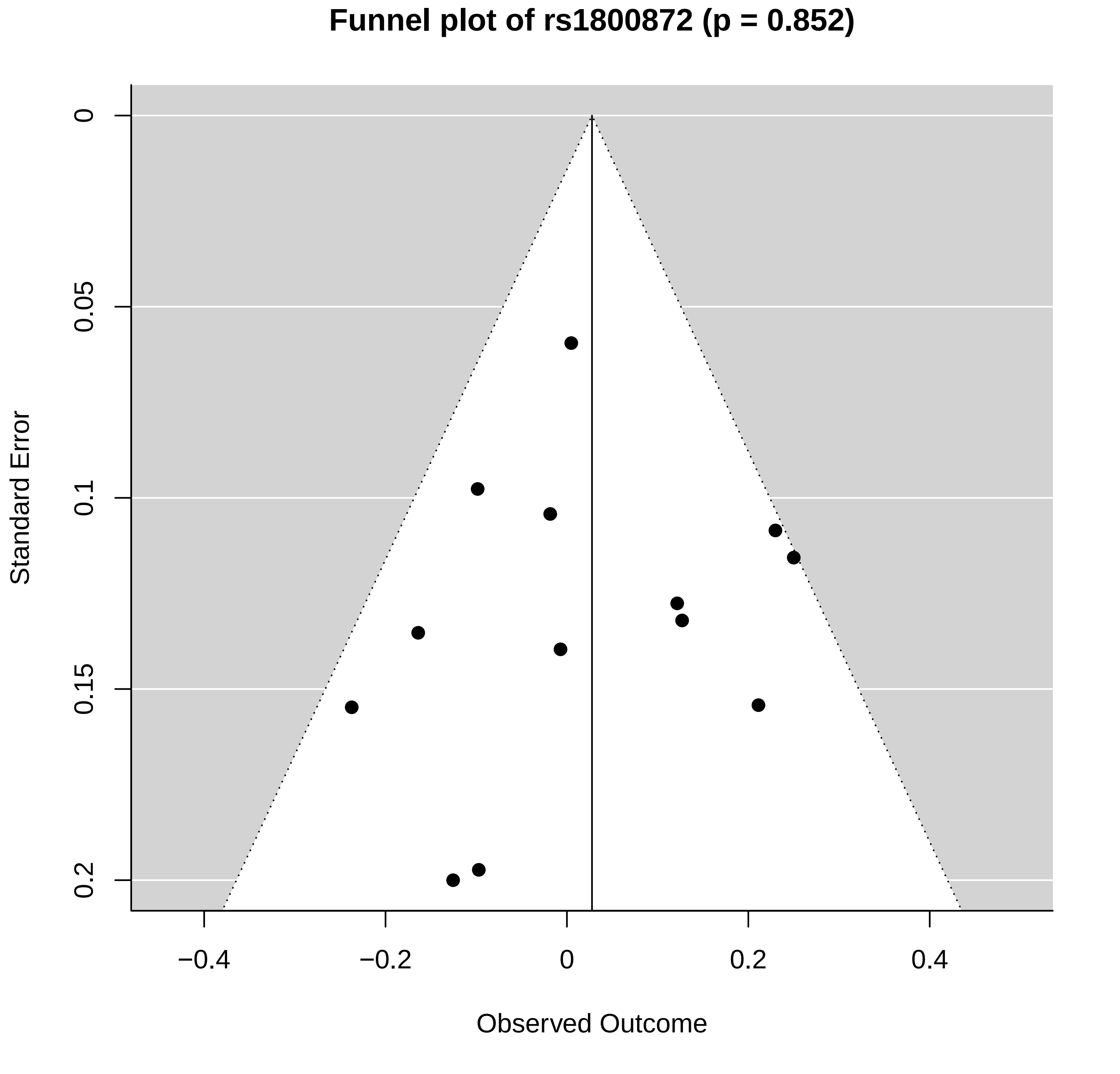

Funnel plot of rs1800872 (p = 0.852)
Standard Error
0
0.05
0.1
0.15
0.2
−0.4
−0.2
0
0.2
0.4
Observed Outcome

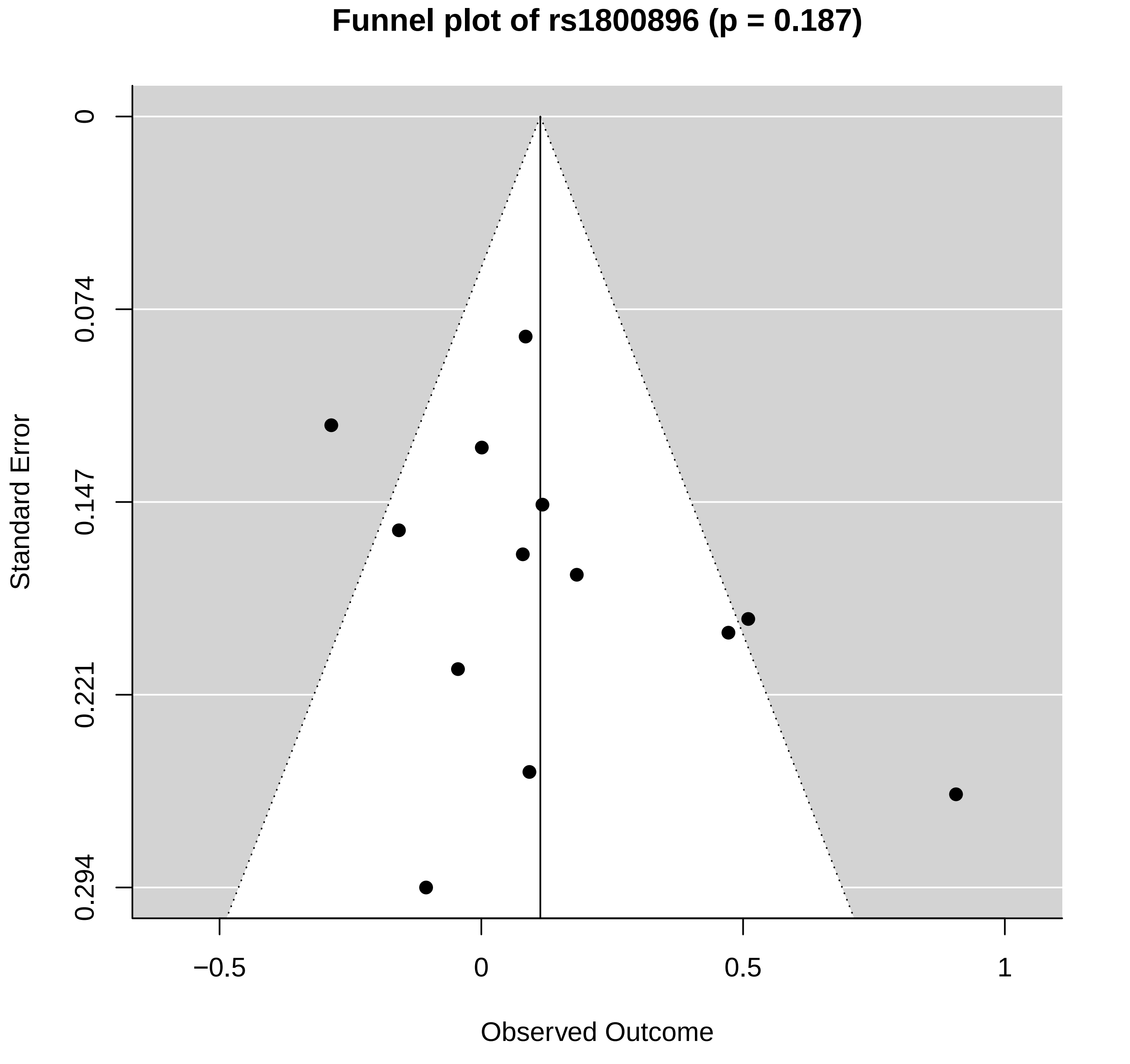

Funnel plot of rs1800896 (p = 0.187)
Standard Error
0
0.074
0.147
0.221
0.294
Observed Outcome
−0.5
0
0.5
1

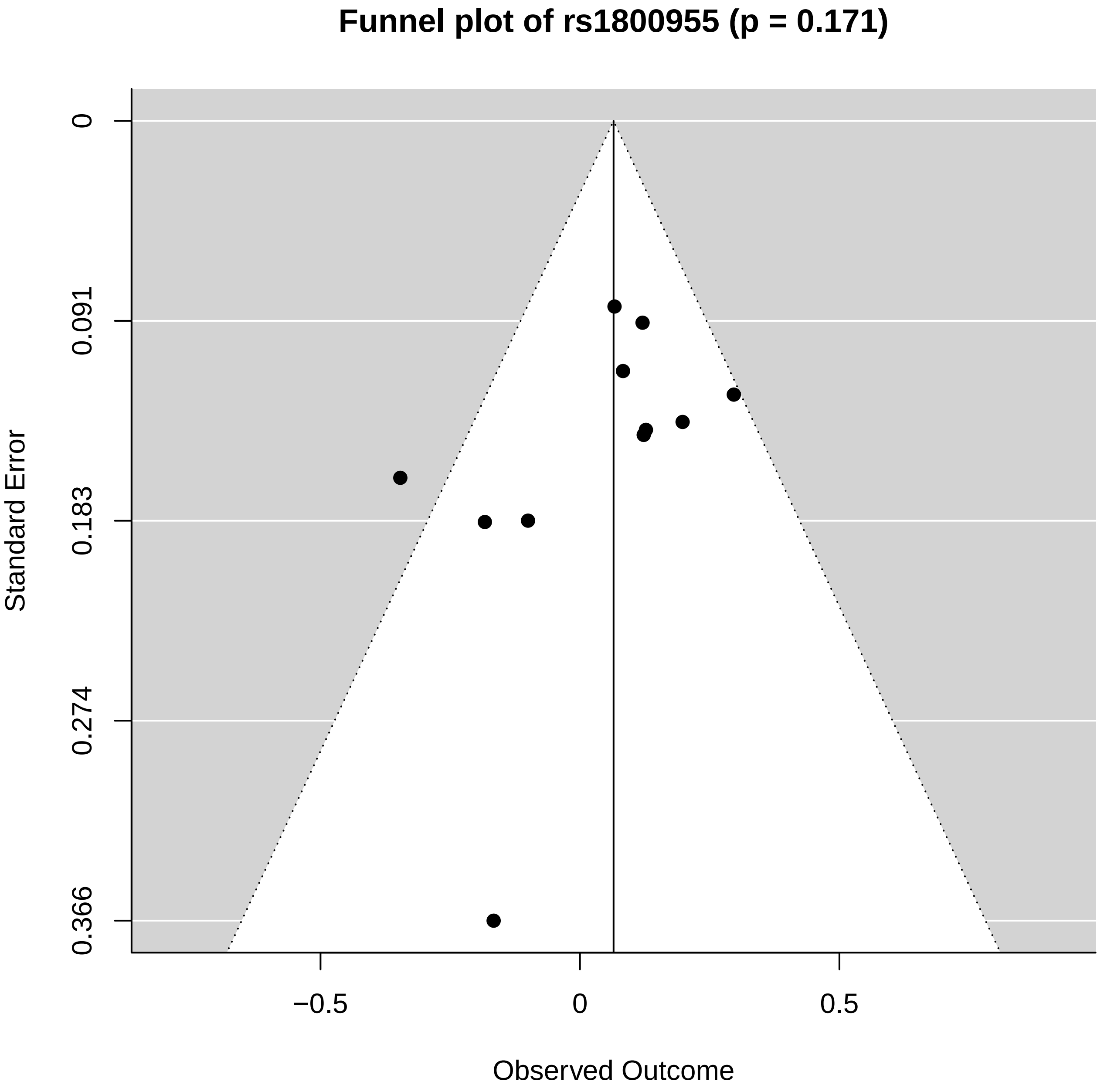
Funnel plot of rs1800955 (p = 0.171)
Standard Error
0
0.091
0.183
0.274
0.366
−0.5
0
0.5
Observed Outcome

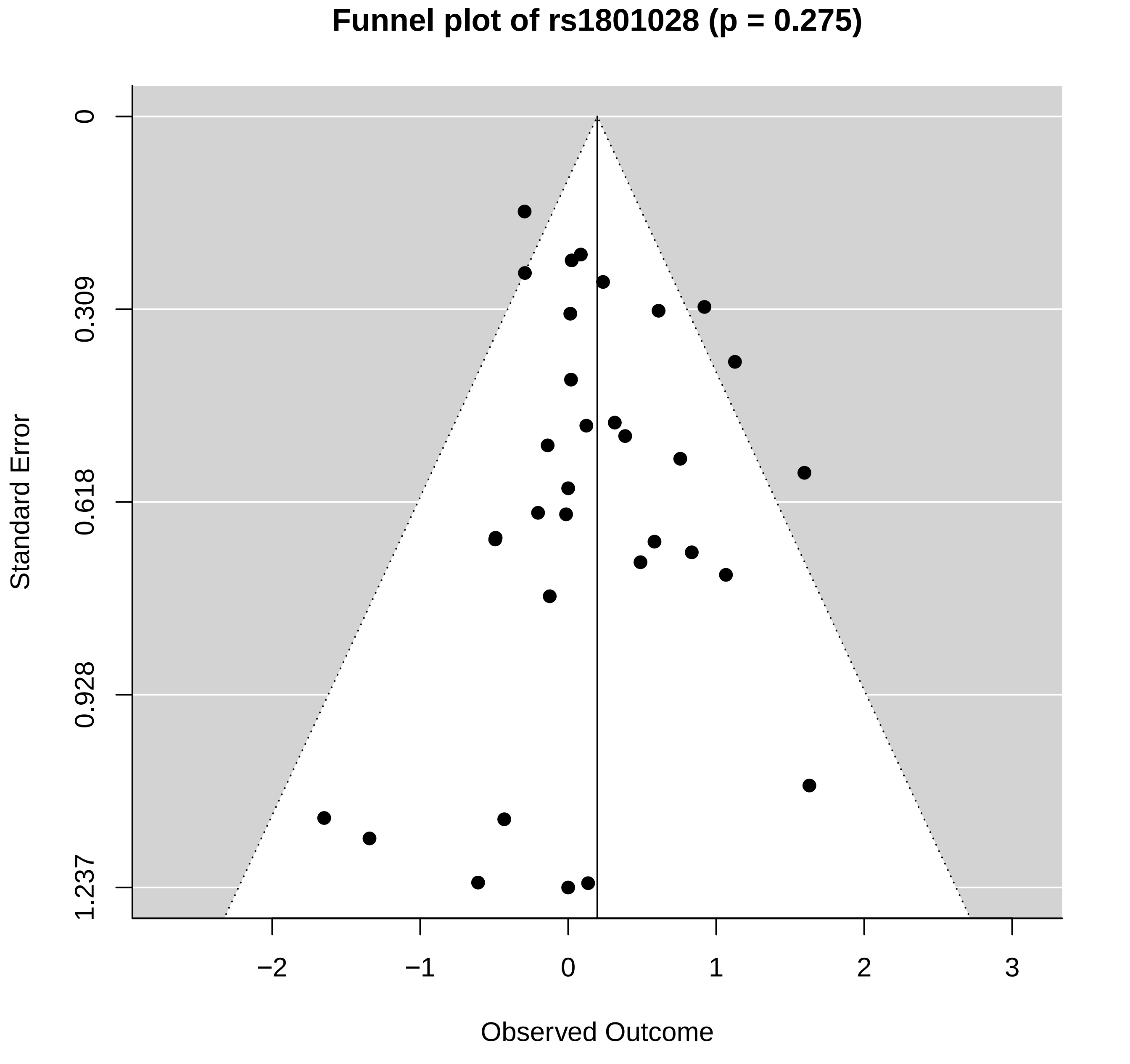
Funnel plot of rs1801028 (p = 0.275)
Standard Error
0
0.309
0.618
0.928
1.237
−2
−1
0
1
2
3
Observed Outcome

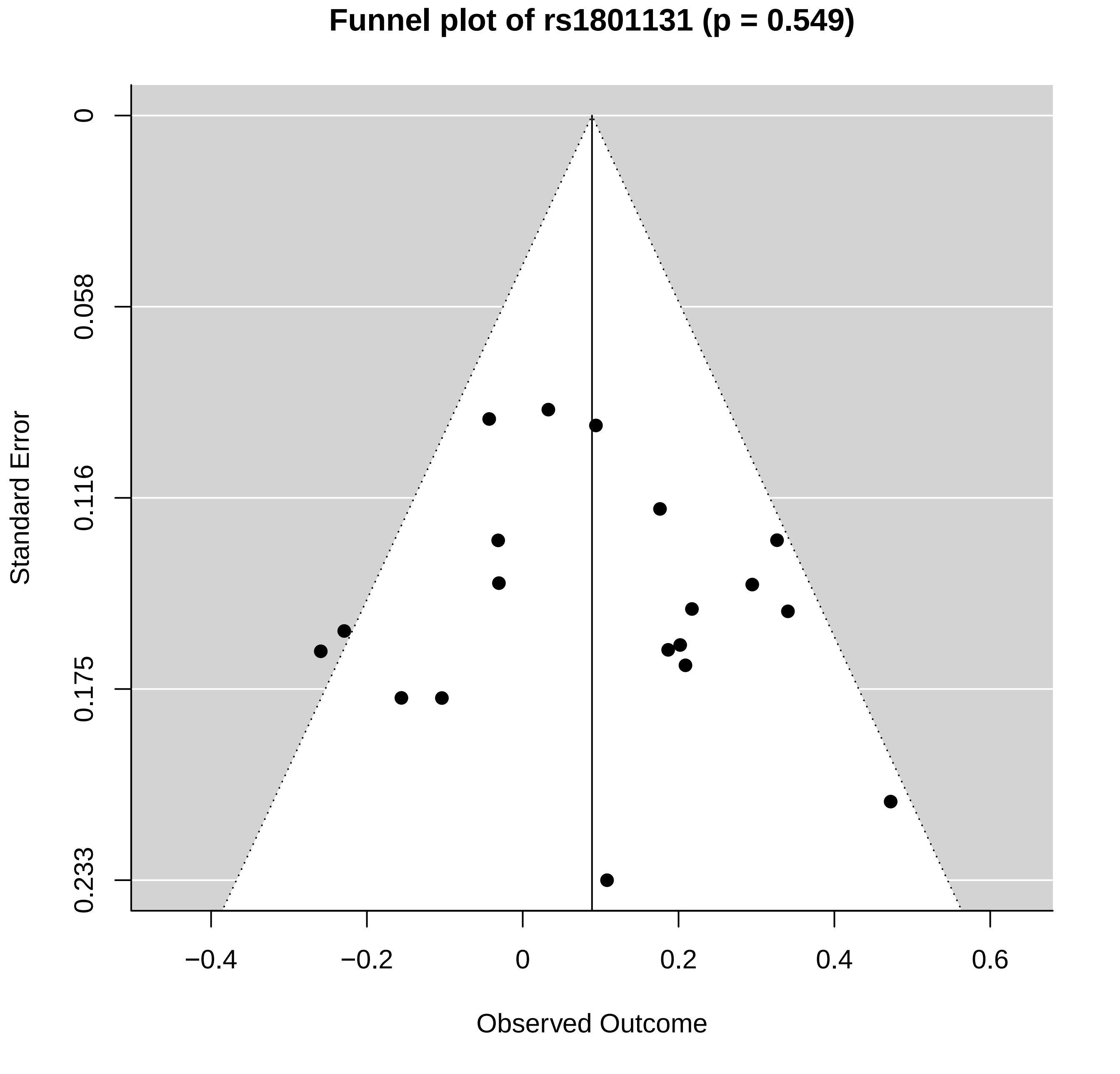

Funnel plot of rs1801131 (p = 0.549)
Standard Error
0
0.058
0.116
0.175
0.233
−0.4
−0.2
0
0.2
0.4
0.6
Observed Outcome

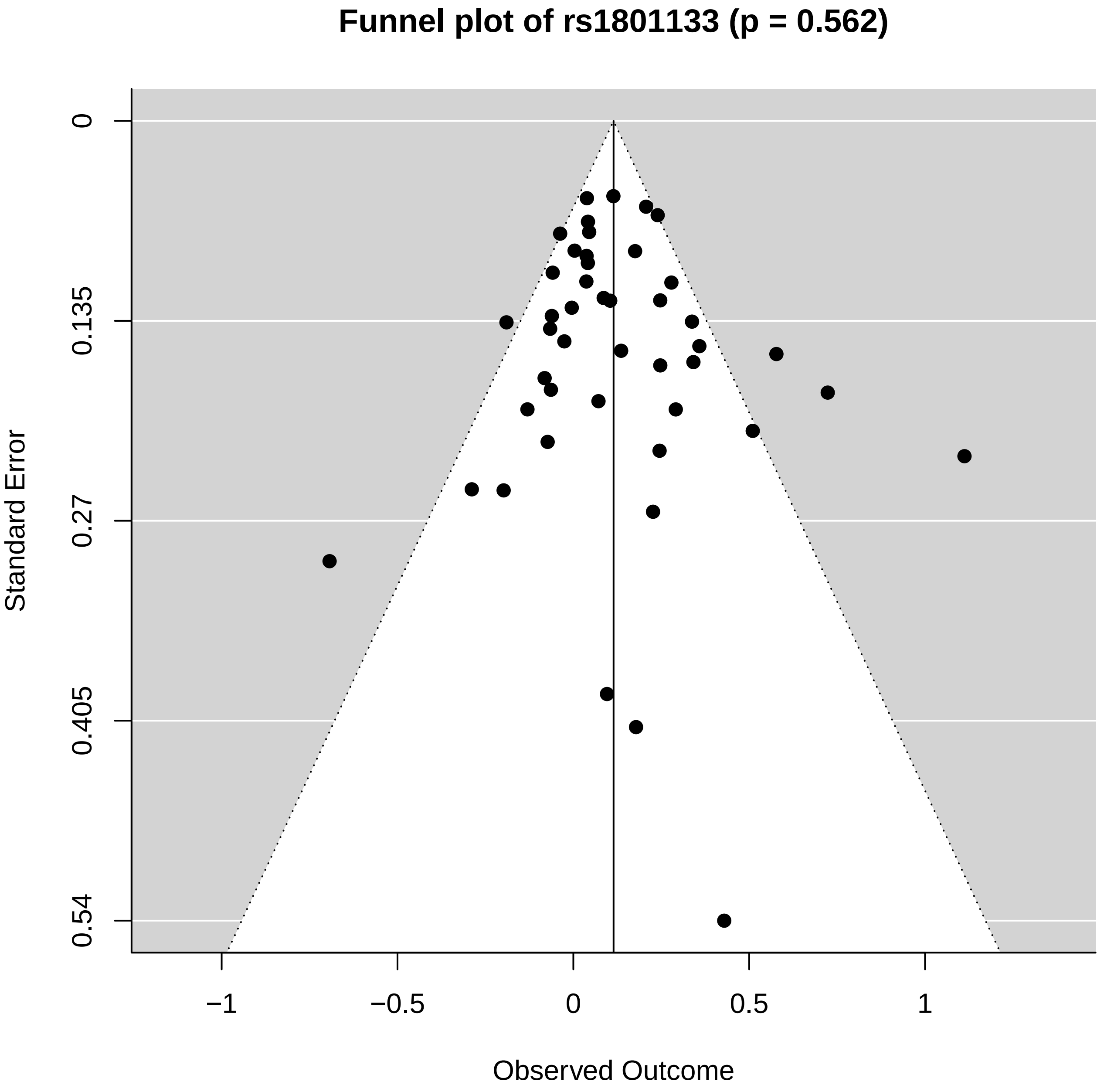

Funnel plot of rs1801133 (p = 0.562)
Standard Error
0
0.135
0.27
0.405
0.54
−1
−0.5
0
0.5
1
Observed Outcome

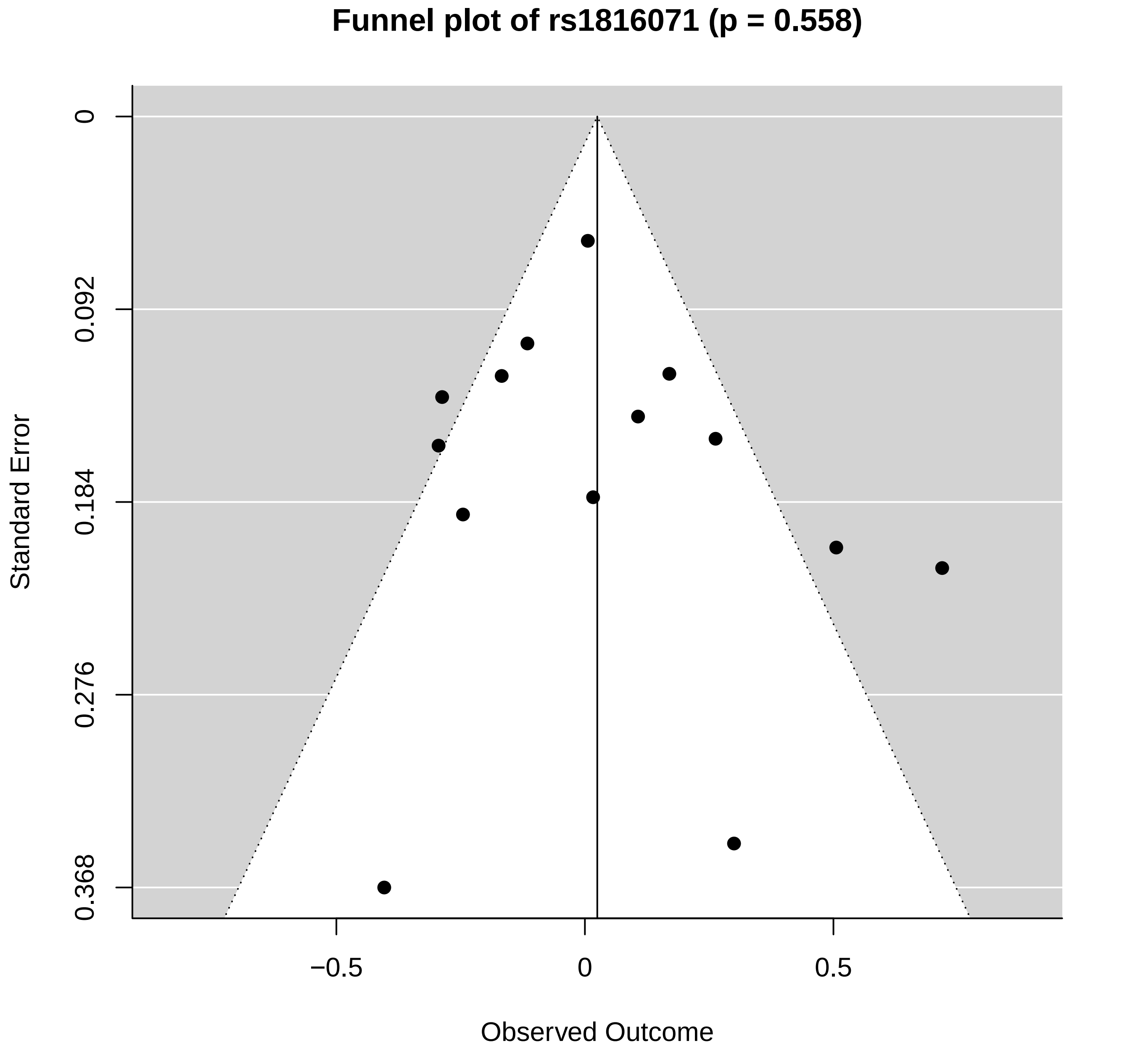
Funnel plot of rs1816071 (p = 0.558)
Standard Error
0
0.092
0.184
0.276
0.368
−0.5
0
0.5
Observed Outcome

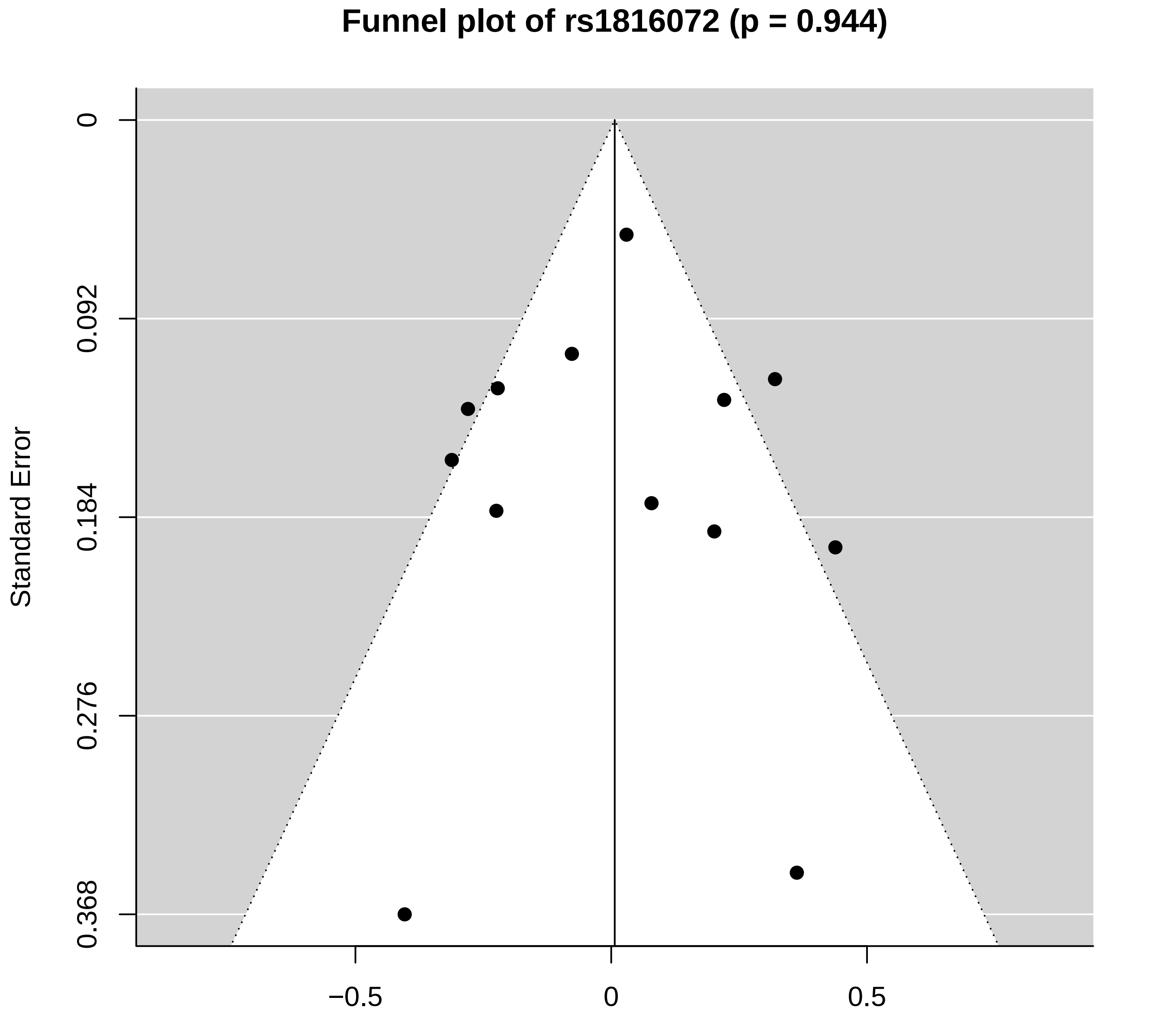

Funnel plot of rs1816072 (p = 0.944)
Standard Error
0
0.092
0.184
0.276
0.368
−0.5
0
0.5
Observed Outcome

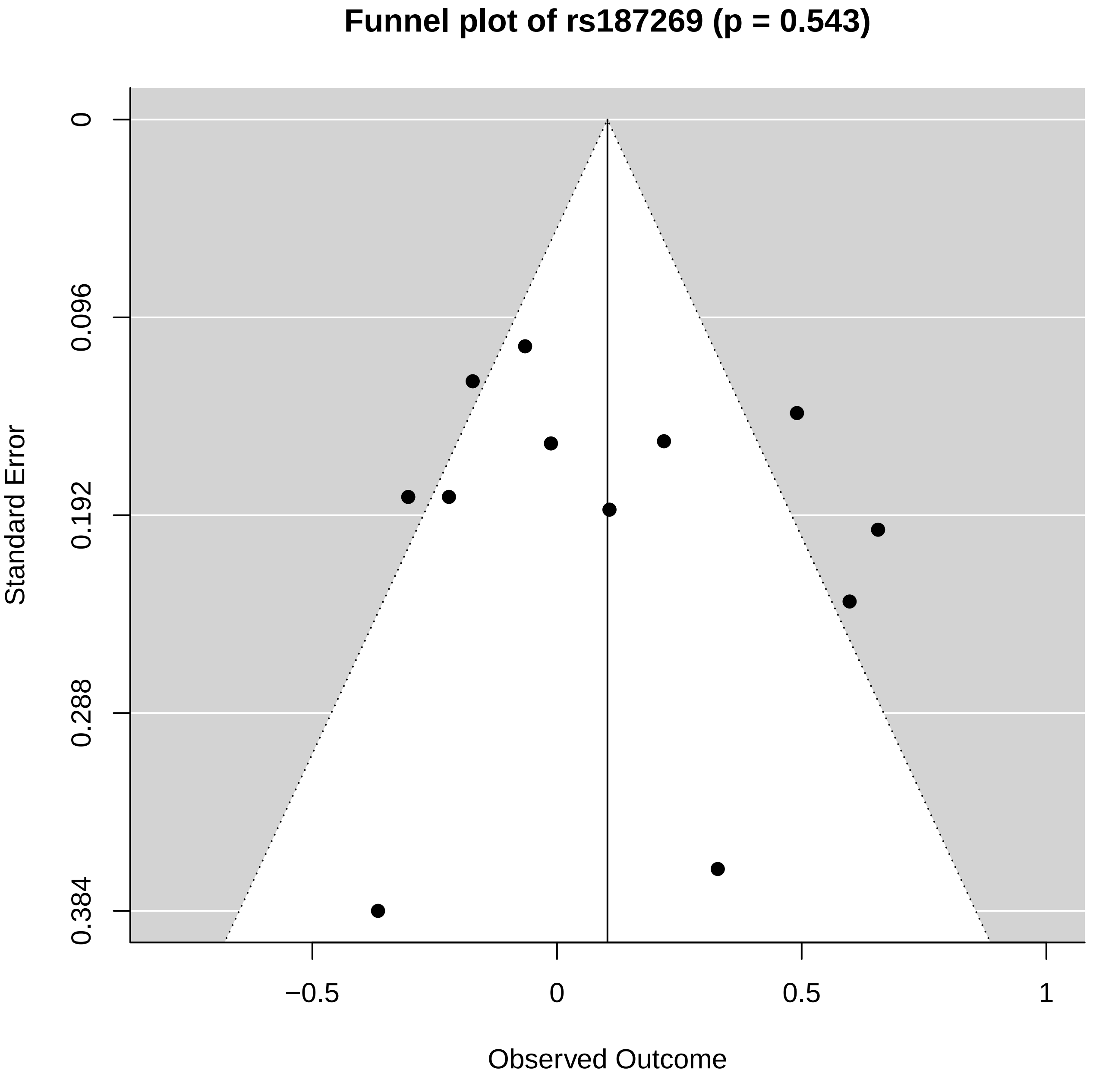

Funnel plot of rs187269 (p = 0.543)
0
0.096
0.192
0.288
0.384
Standard Error
−0.5
0
0.5
1
Observed Outcome

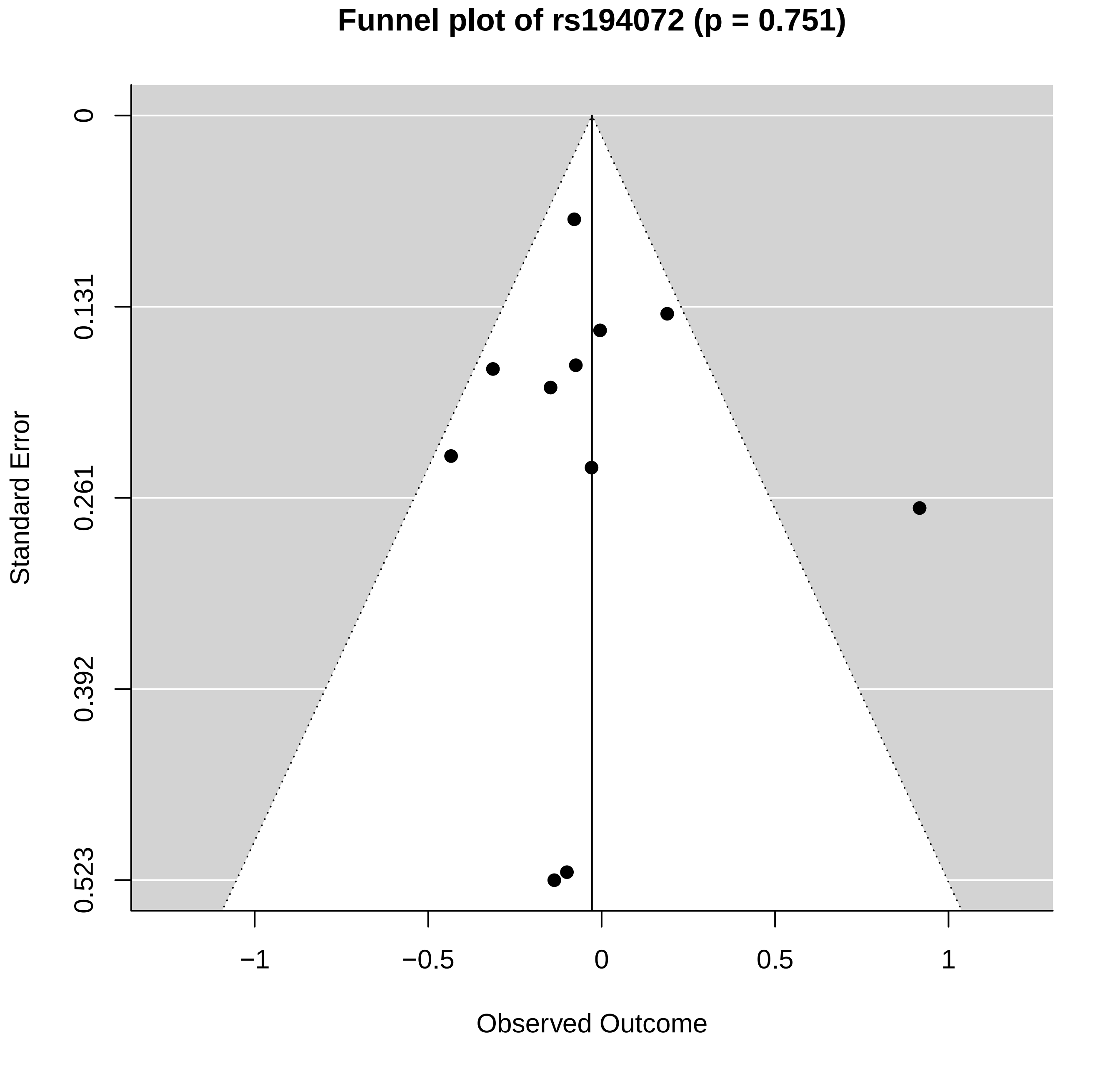

Funnel plot of rs194072 (p = 0.751)
0
0.131
0.261
0.392
0.523
Standard Error
−1
−0.5
0
0.5
1
Observed Outcome

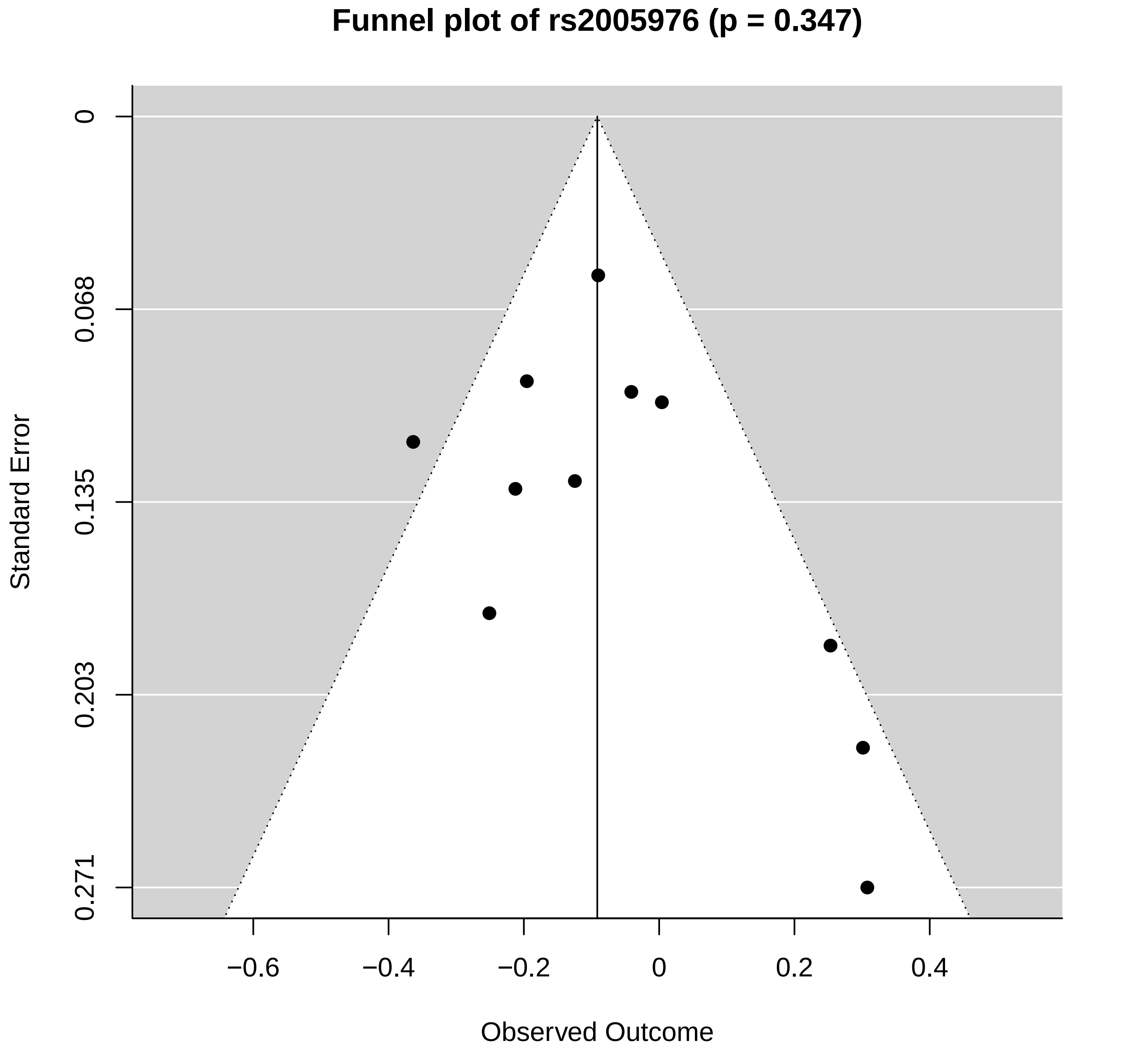

Funnel plot of rs2005976 (p = 0.347)
Standard Error
0
0.068
0.135
0.203
0.271
−0.6
−0.4
−0.2
0
0.2
0.4
Observed Outcome

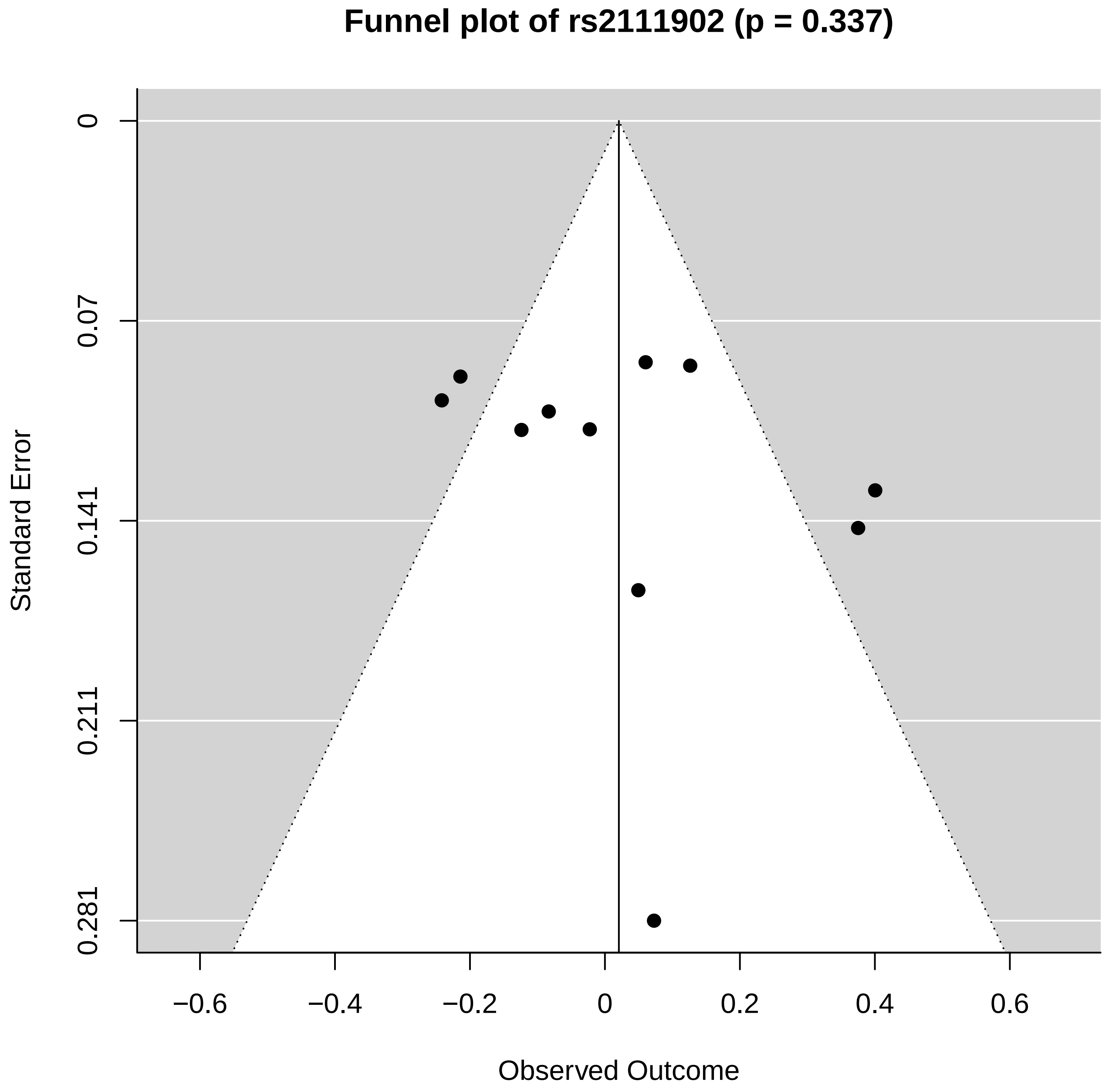
Funnel plot of rs2111902 (p = 0.337)
Standard Error
0
0.07
0.141
0.211
0.281
−0.6
−0.4
−0.2
0
0.2
0.4
0.6
Observed Outcome

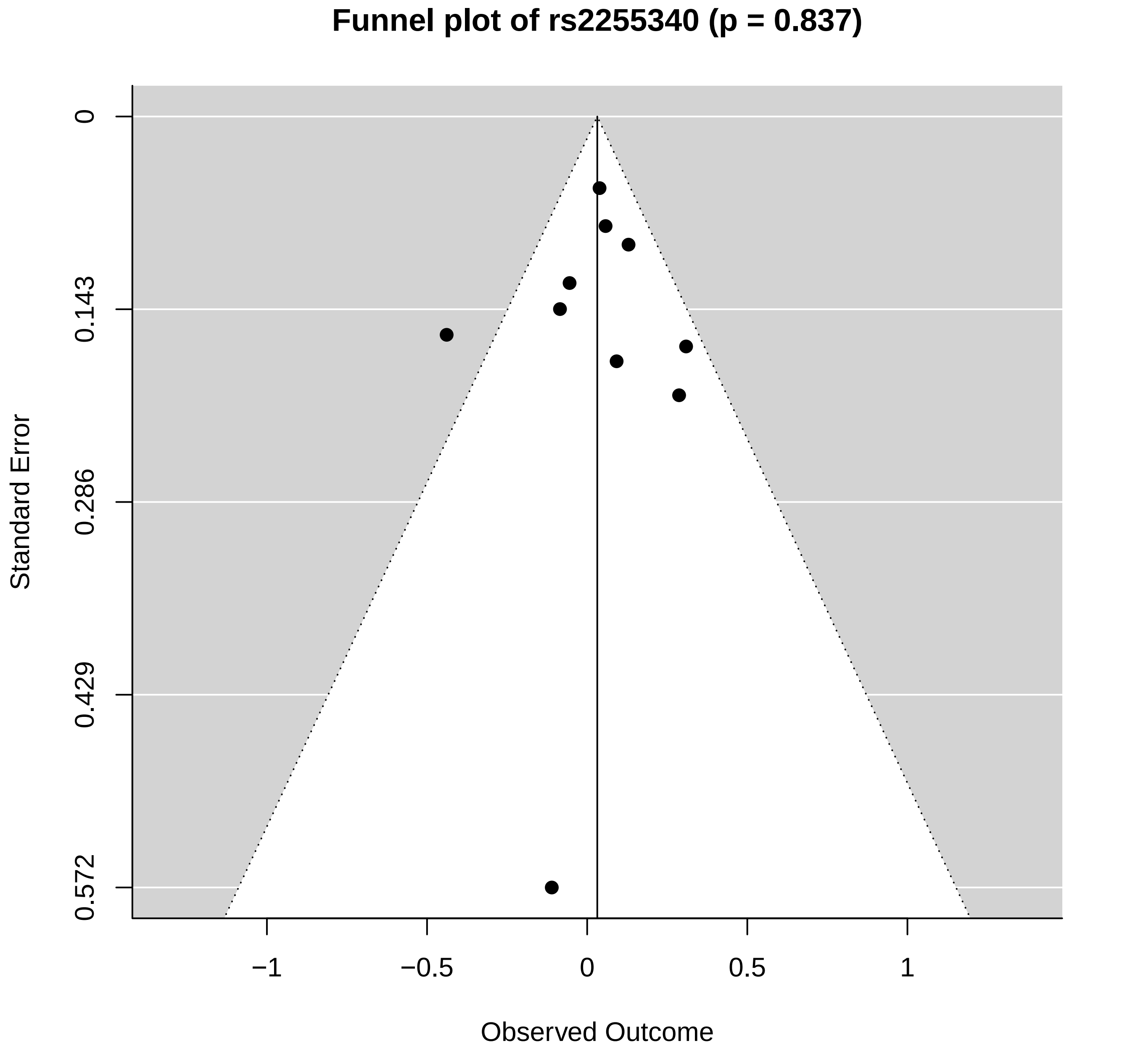
Funnel plot of rs2255340 (p = 0.837)
Standard Error
0
0.143
0.286
0.429
0.572
−1
−0.5
0
0.5
1
Observed Outcome

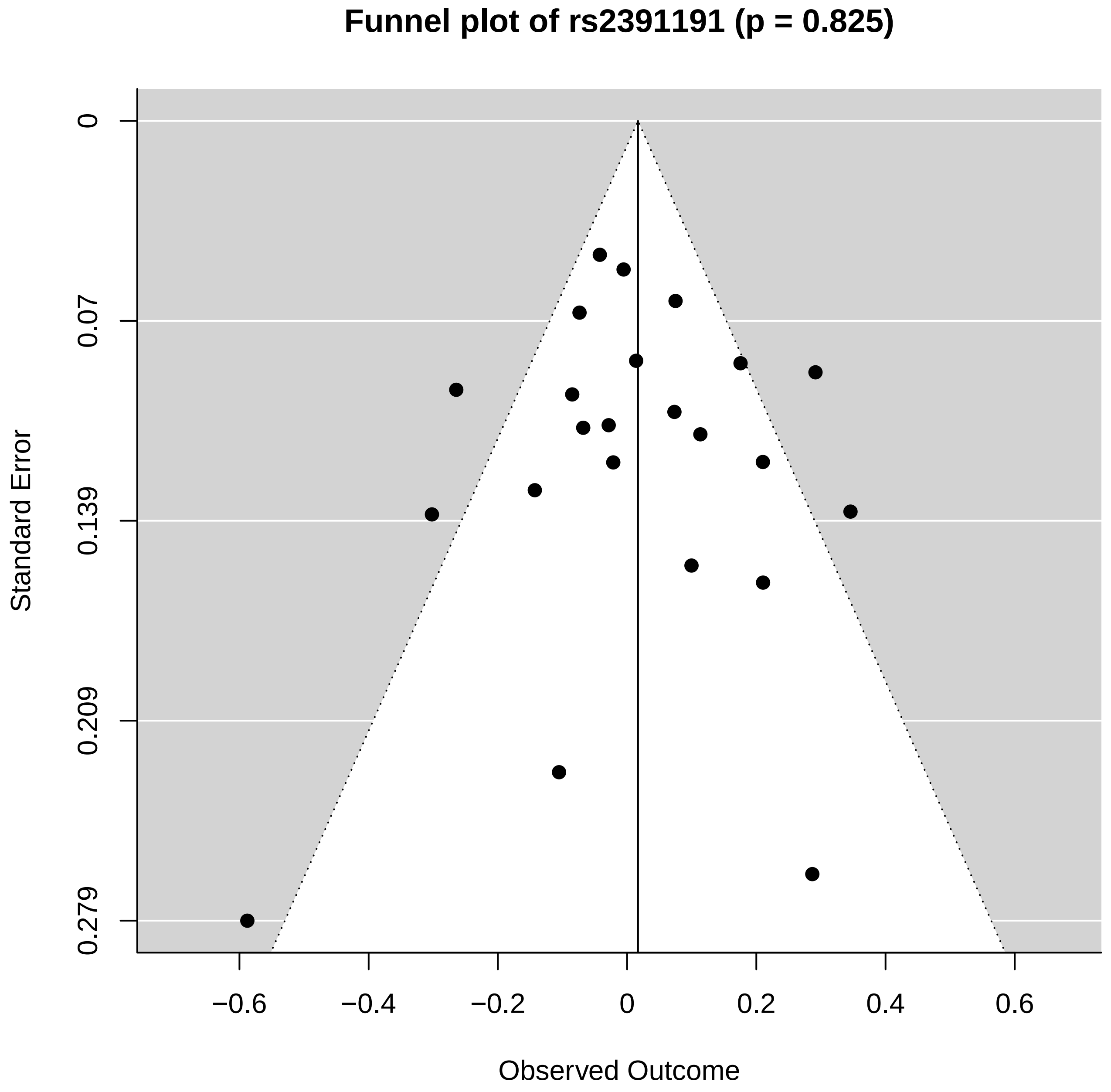

Funnel plot of rs2391191 (p = 0.825)
Standard Error
0
0.07
0.139
0.209
0.279
−0.6
−0.4
−0.2
0
0.2
0.4
0.6
Observed Outcome

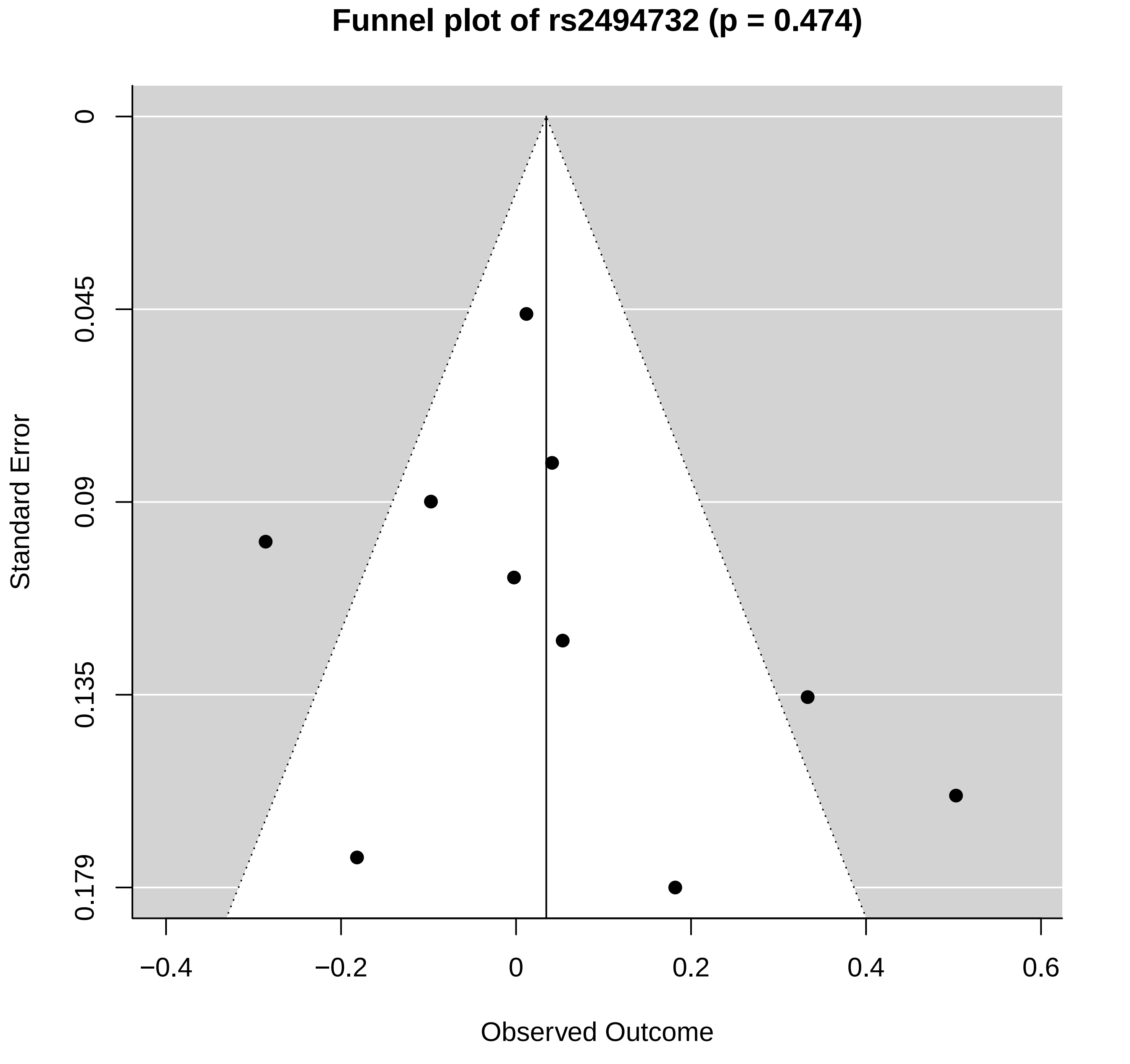
Funnel plot of rs2494732 (p = 0.474)
Standard Error
0
0.045
0.09
0.135
0.179
−0.4
−0.2
0
0.2
0.4
0.6
Observed Outcome

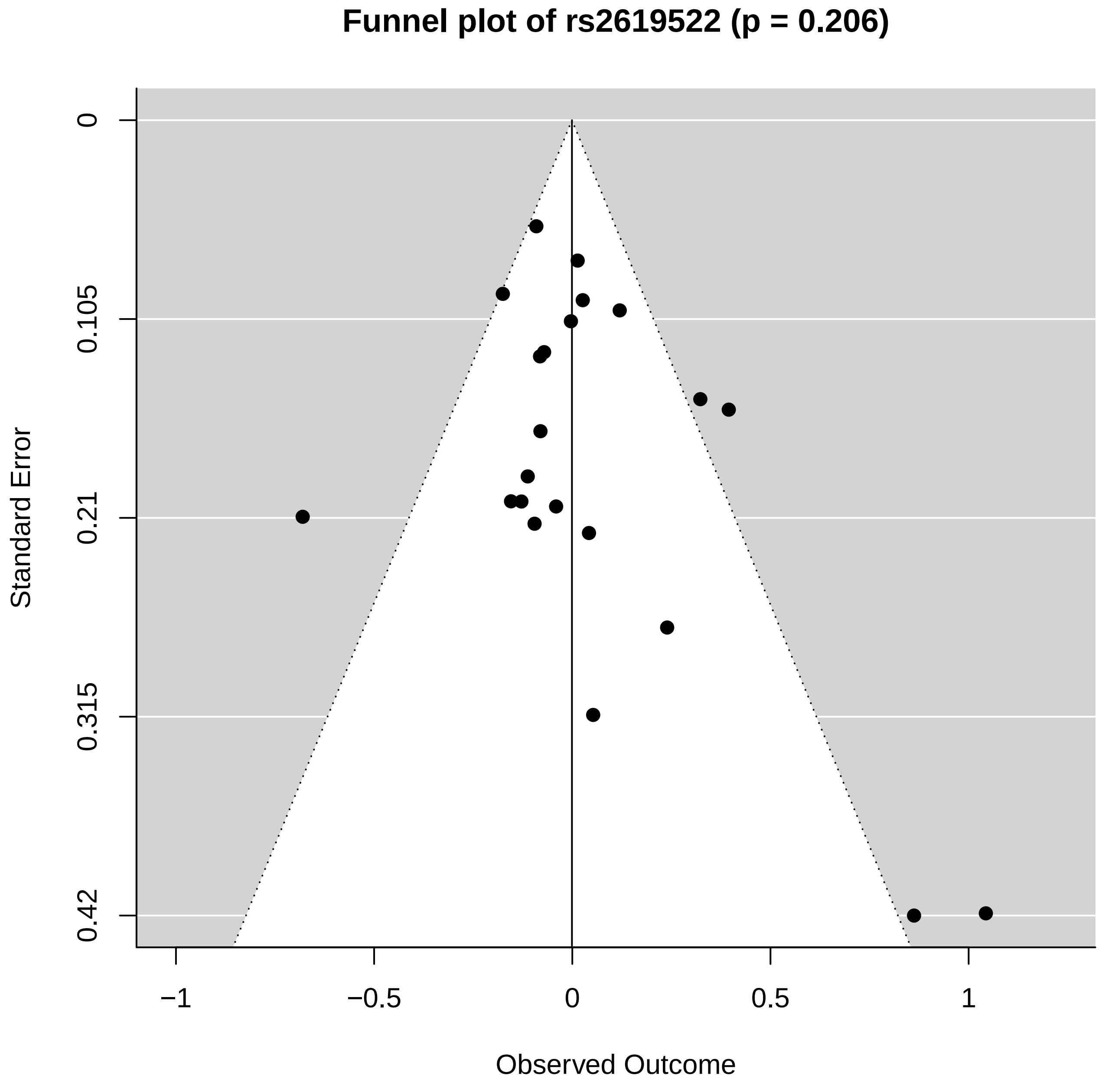

Funnel plot of rs2619522 (p = 0.206)
Standard Error
0
0.105
0.21
0.315
0.42
−1
−0.5
0
0.5
1
Observed Outcome

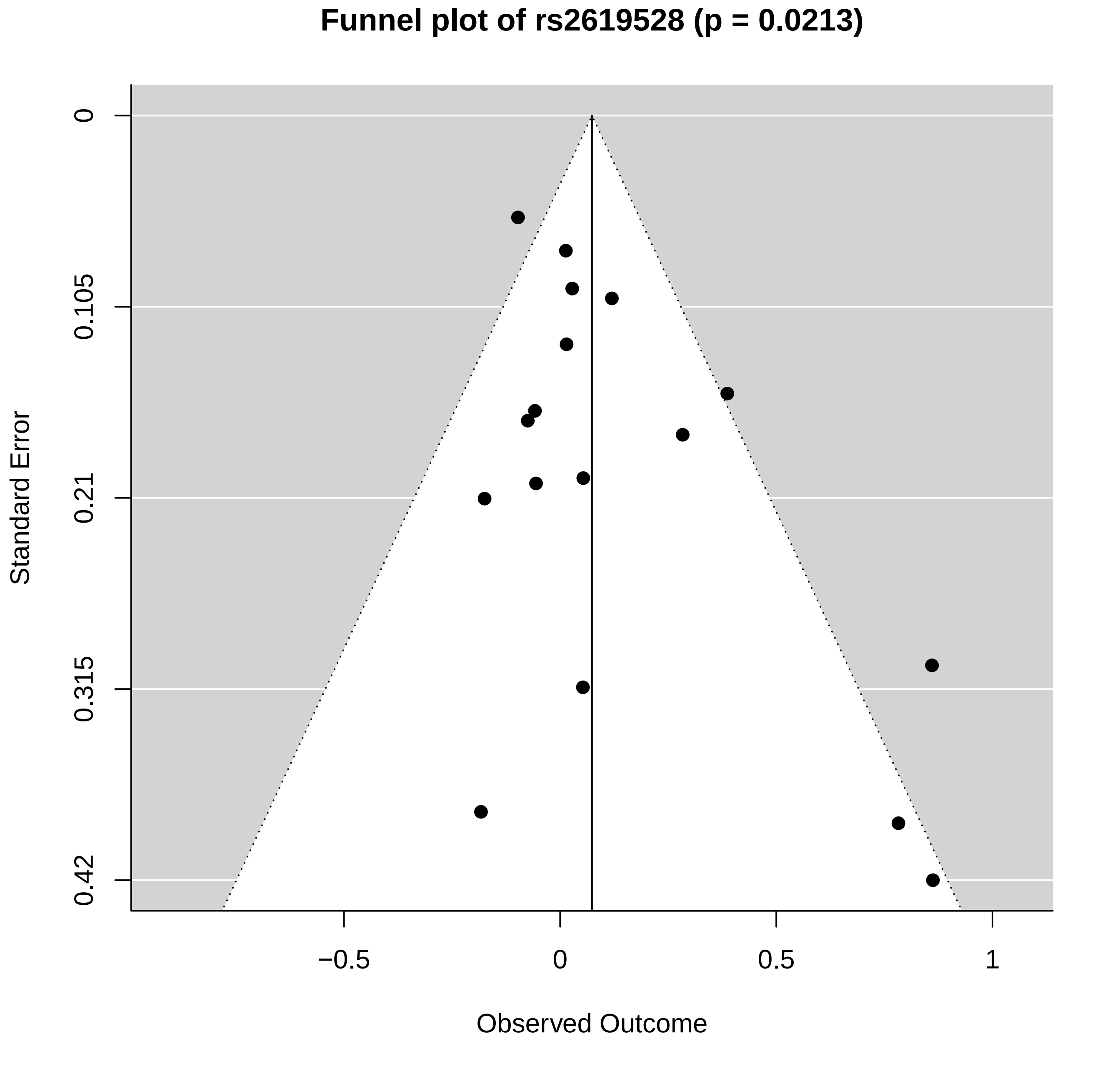

Funnel plot of rs2619528 (p = 0.0213)
Standard Error
0
0.105
0.21
0.315
0.42
−0.5
0
0.5
1
Observed Outcome

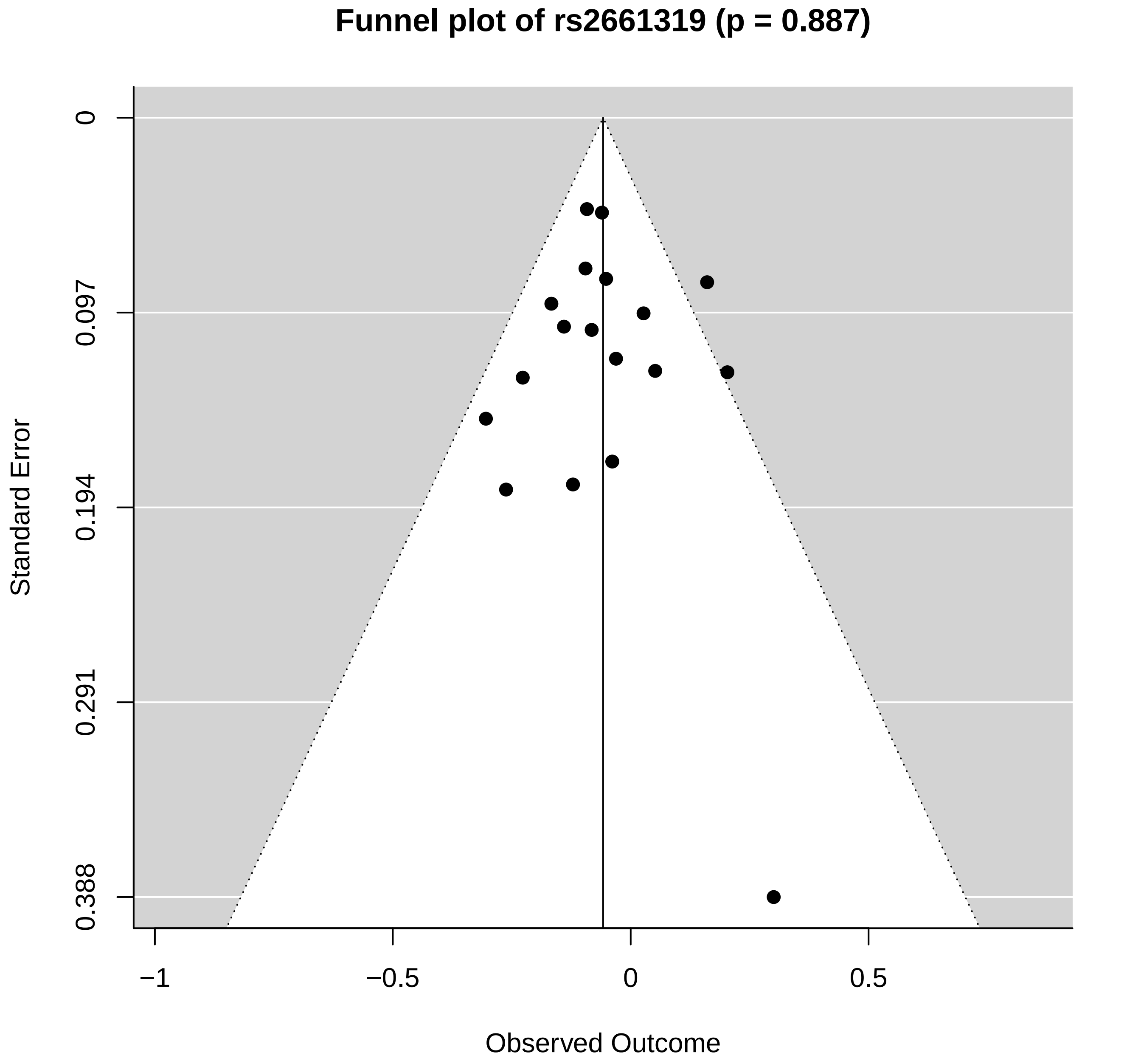
Funnel plot of rs2661319 (p = 0.887)
Standard Error
0
0.097
0.194
0.291
0.388
−1
−0.5
0
0.5
Observed Outcome

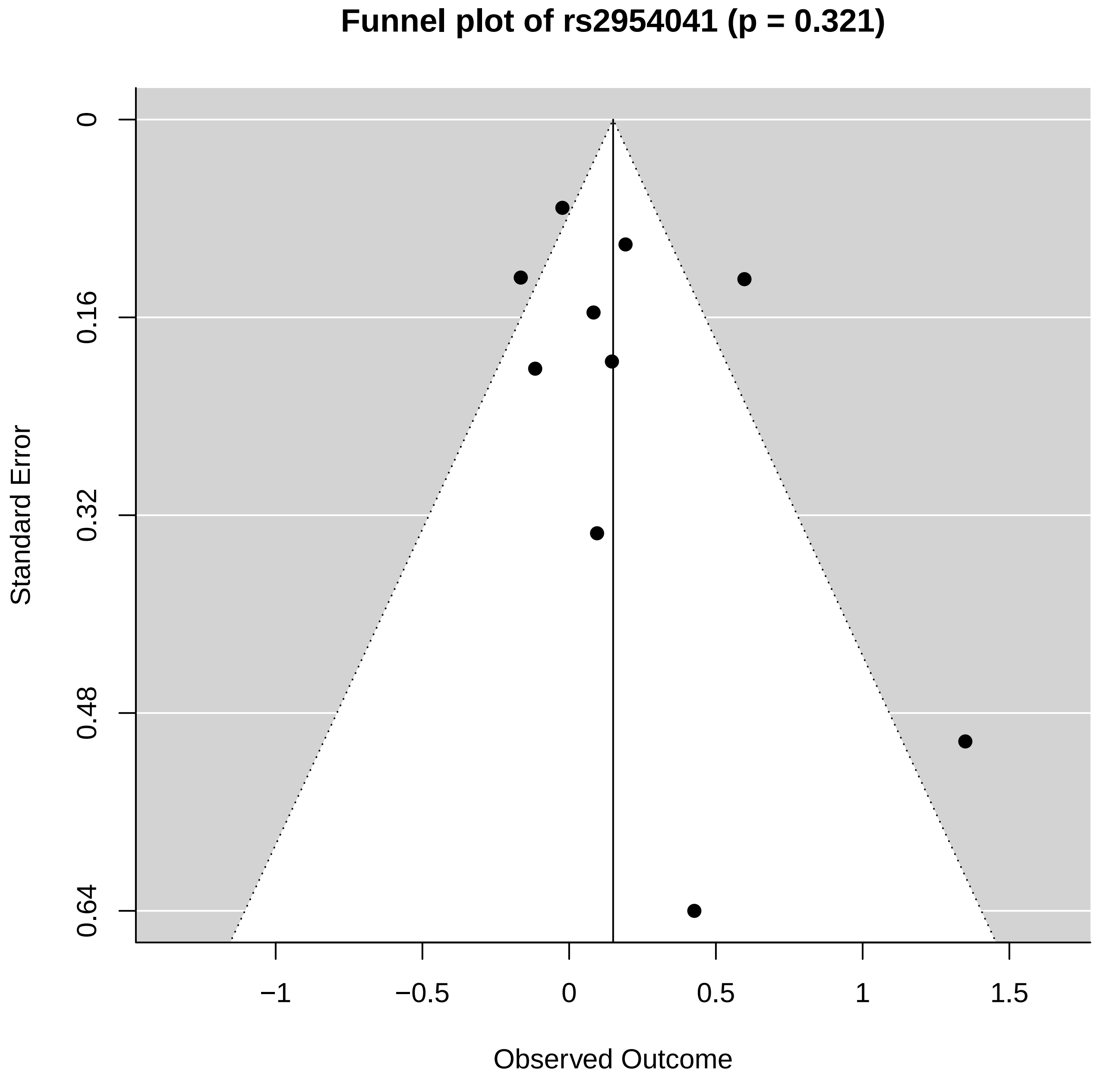

Funnel plot of rs2954041 (p = 0.321)
Standard Error
0
0.16
0.32
0.48
0.64
−1
−0.5
0
0.5
1
1.5
Observed Outcome

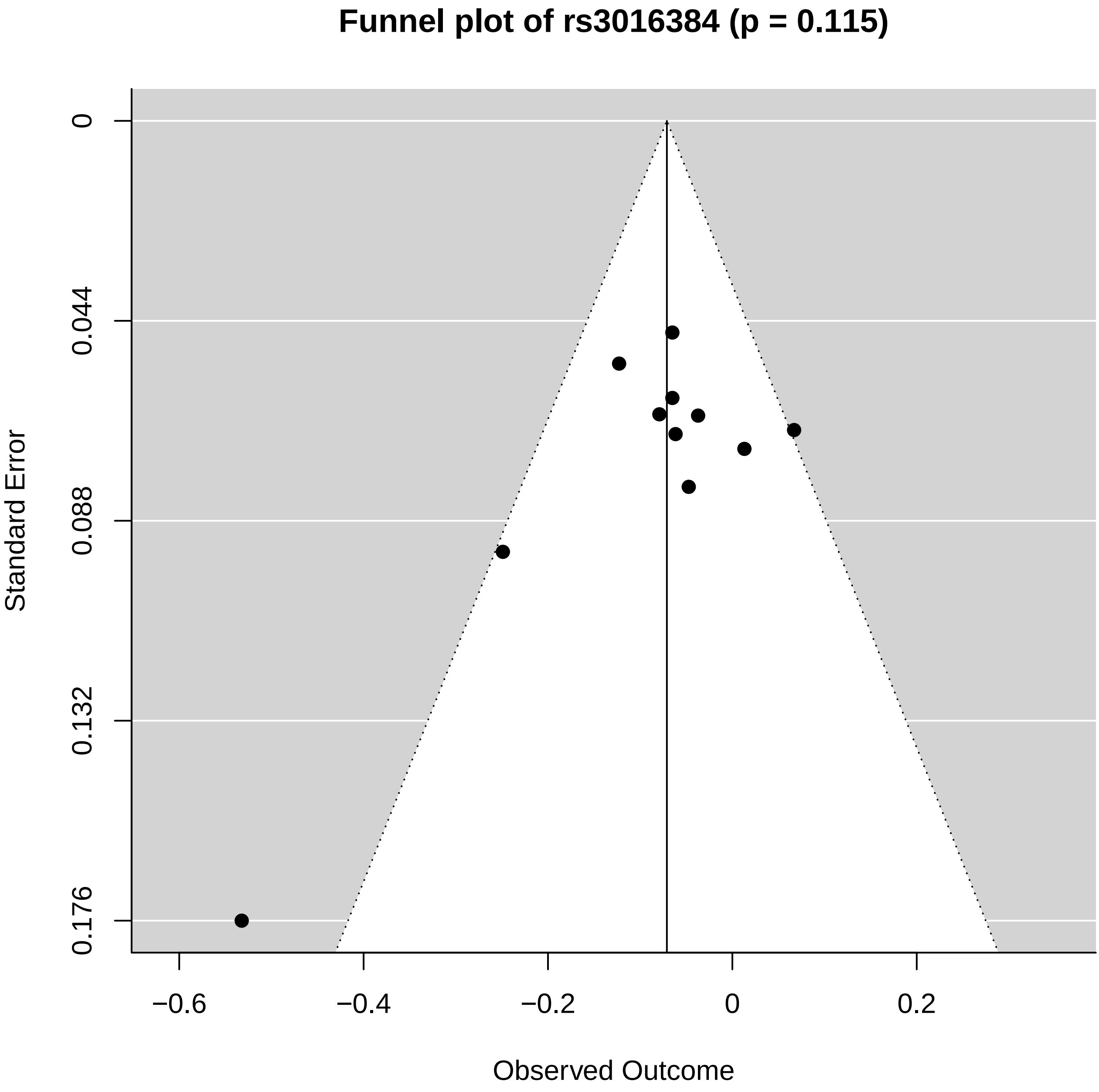
Funnel plot of rs3016384 (p = 0.115)
0
0.044
0.088
0.132
0.176
Standard Error
−0.6
−0.4
−0.2
0
0.2
Observed Outcome

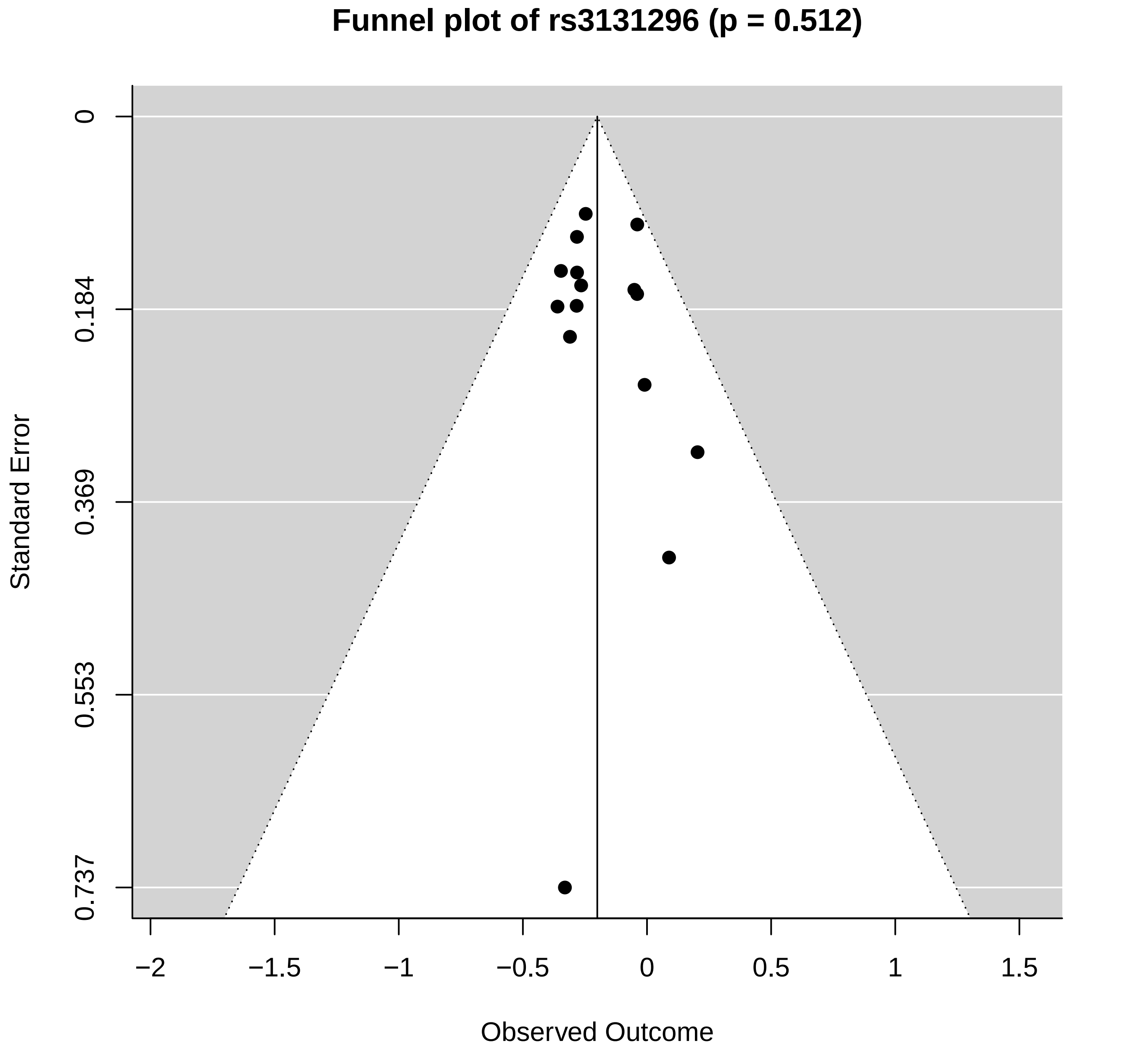

Funnel plot of rs3131296 (p = 0.512)
Standard Error
0
0.184
0.369
0.553
0.737
−2
−1.5
−1
−0.5
0
0.5
1
1.5
Observed Outcome

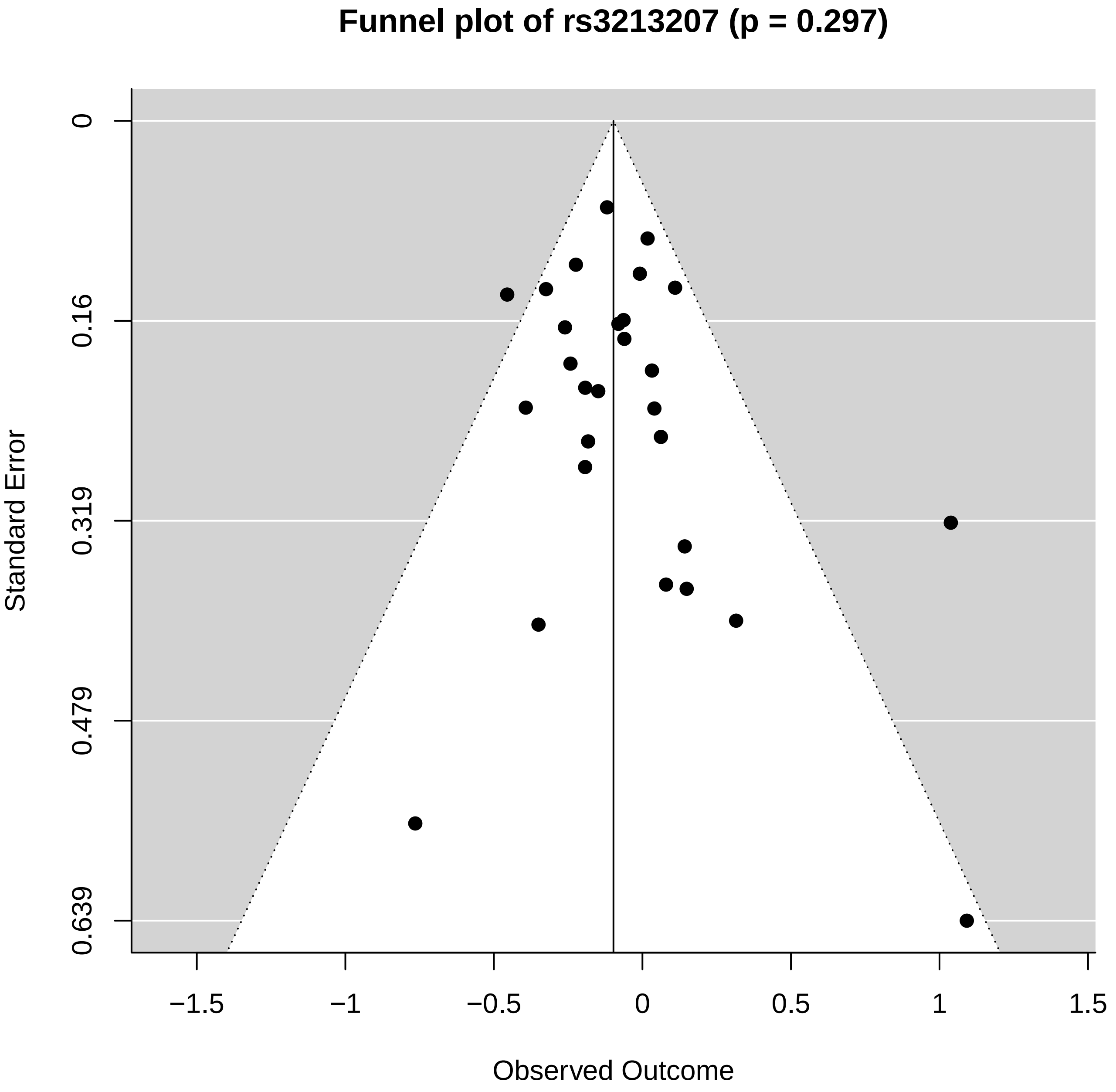

Funnel plot of rs3213207 (p = 0.297)
Standard Error
0
0.16
0.319
0.479
0.639
−1.5
−1
−0.5
0
0.5
1
1.5
Observed Outcome

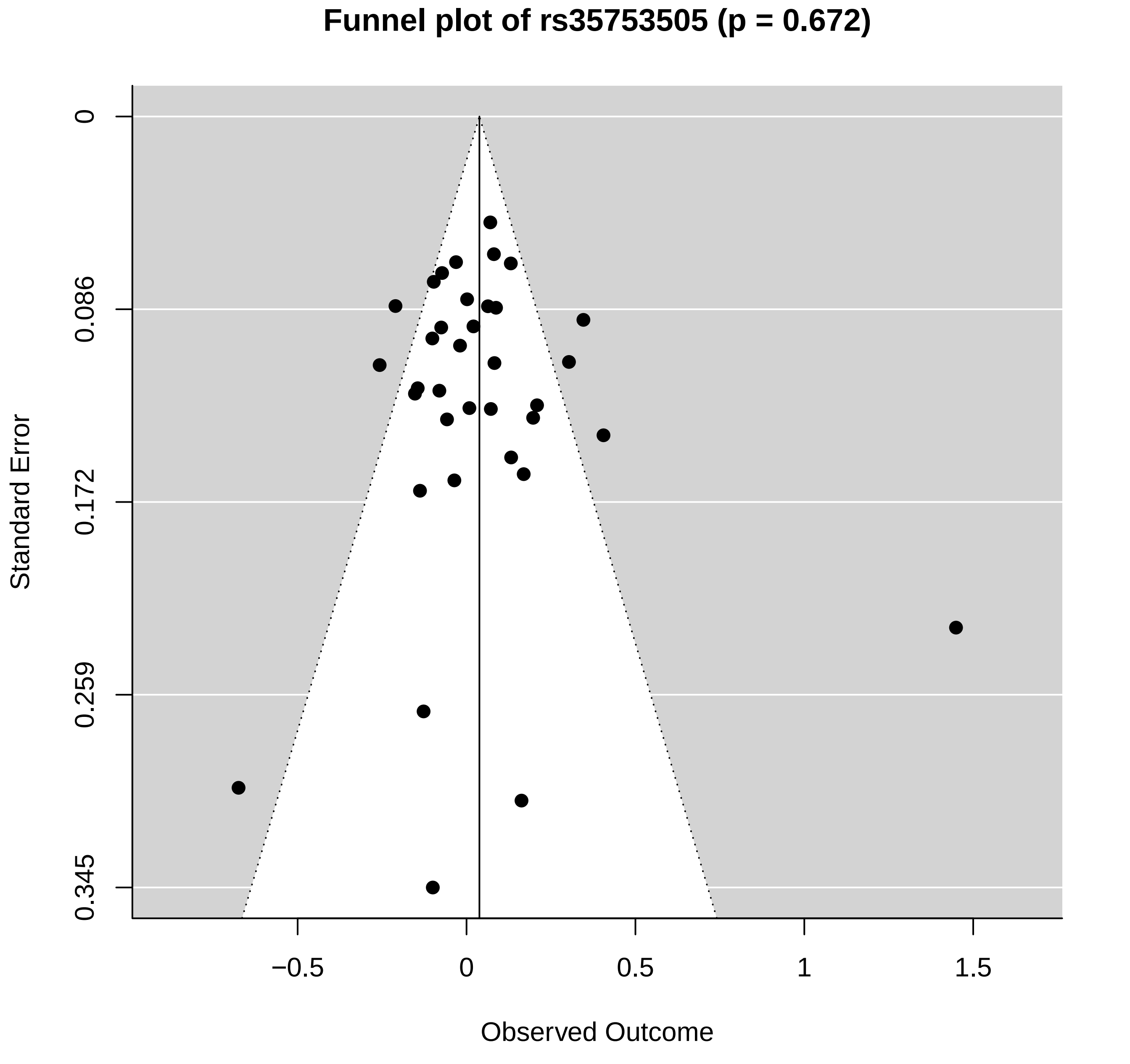

Funnel plot of rs35753505 (p = 0.672)
0
0.086
0.172
0.259
0.345
Standard Error
−0.5
0
0.5
1
1.5
Observed Outcome

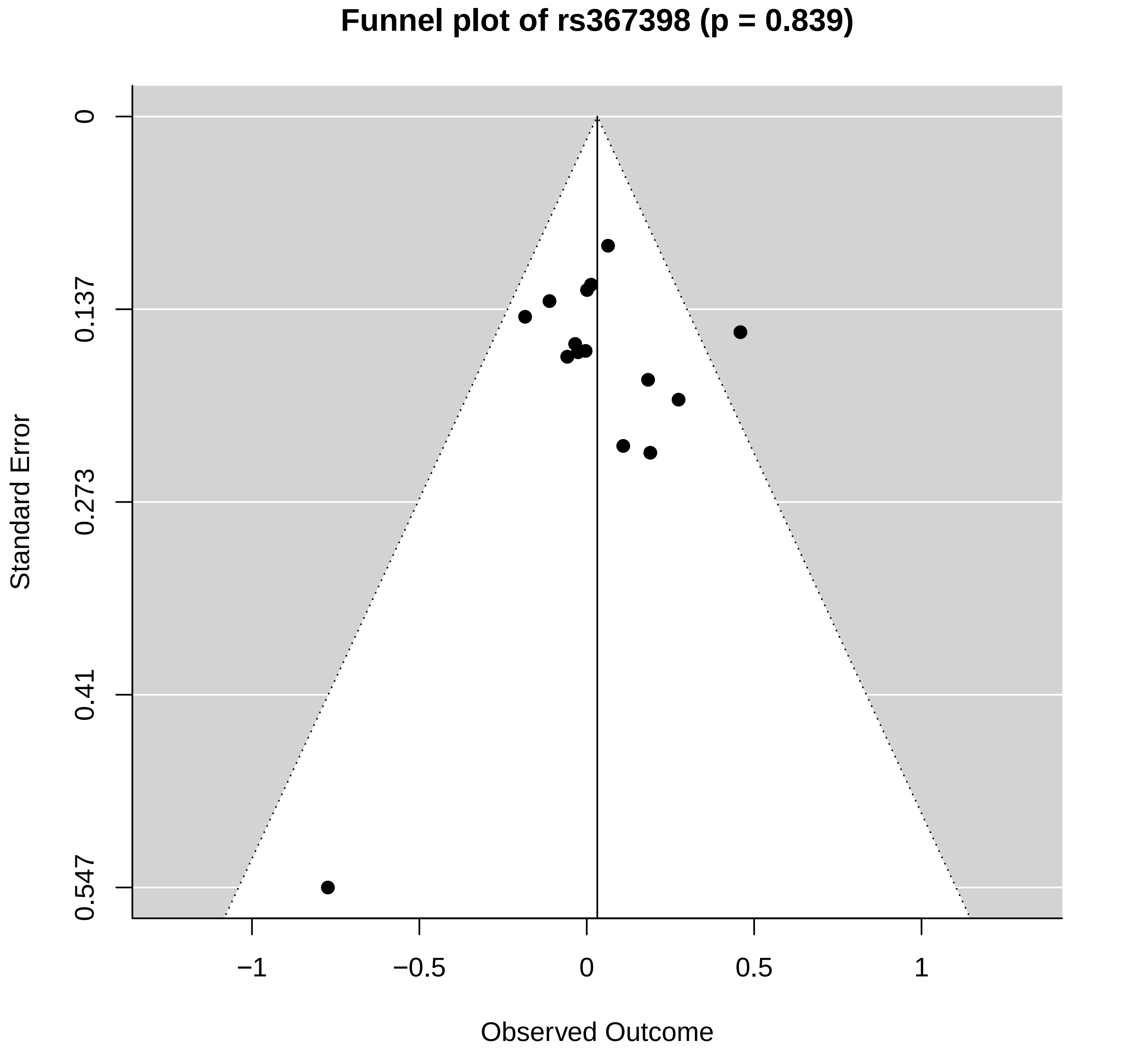
Funnel plot of rs367398 (p = 0.839)
Standard Error
0
0.137
0.273
0.41
0.547
−1
−0.5
0
0.5
1
Observed Outcome

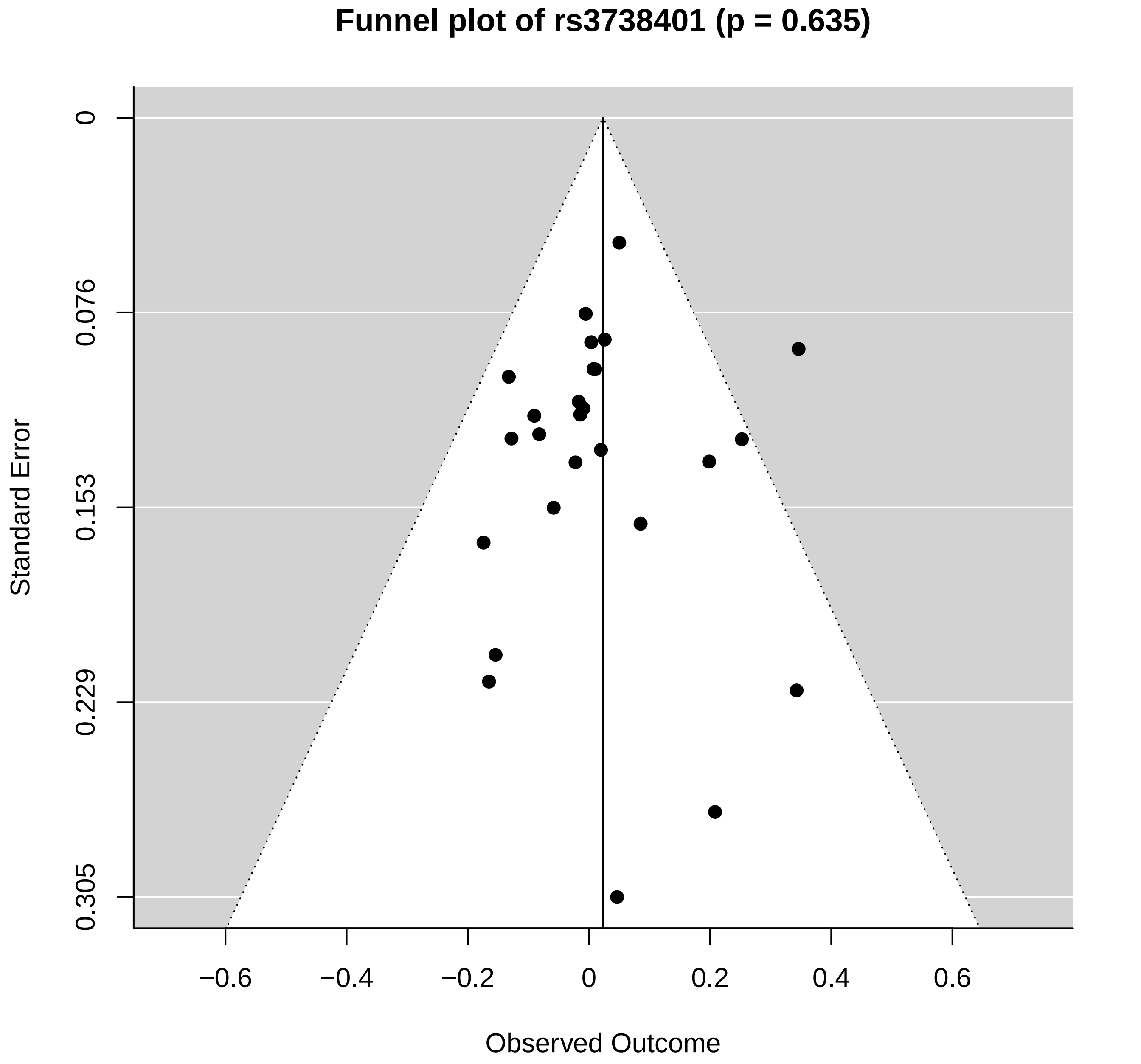
Funnel plot of rs3738401 (p = 0.635)
Standard Error
0
0.076
0.153
0.229
0.305
−0.6
−0.4
−0.2
0
0.2
0.4
0.6
Observed Outcome

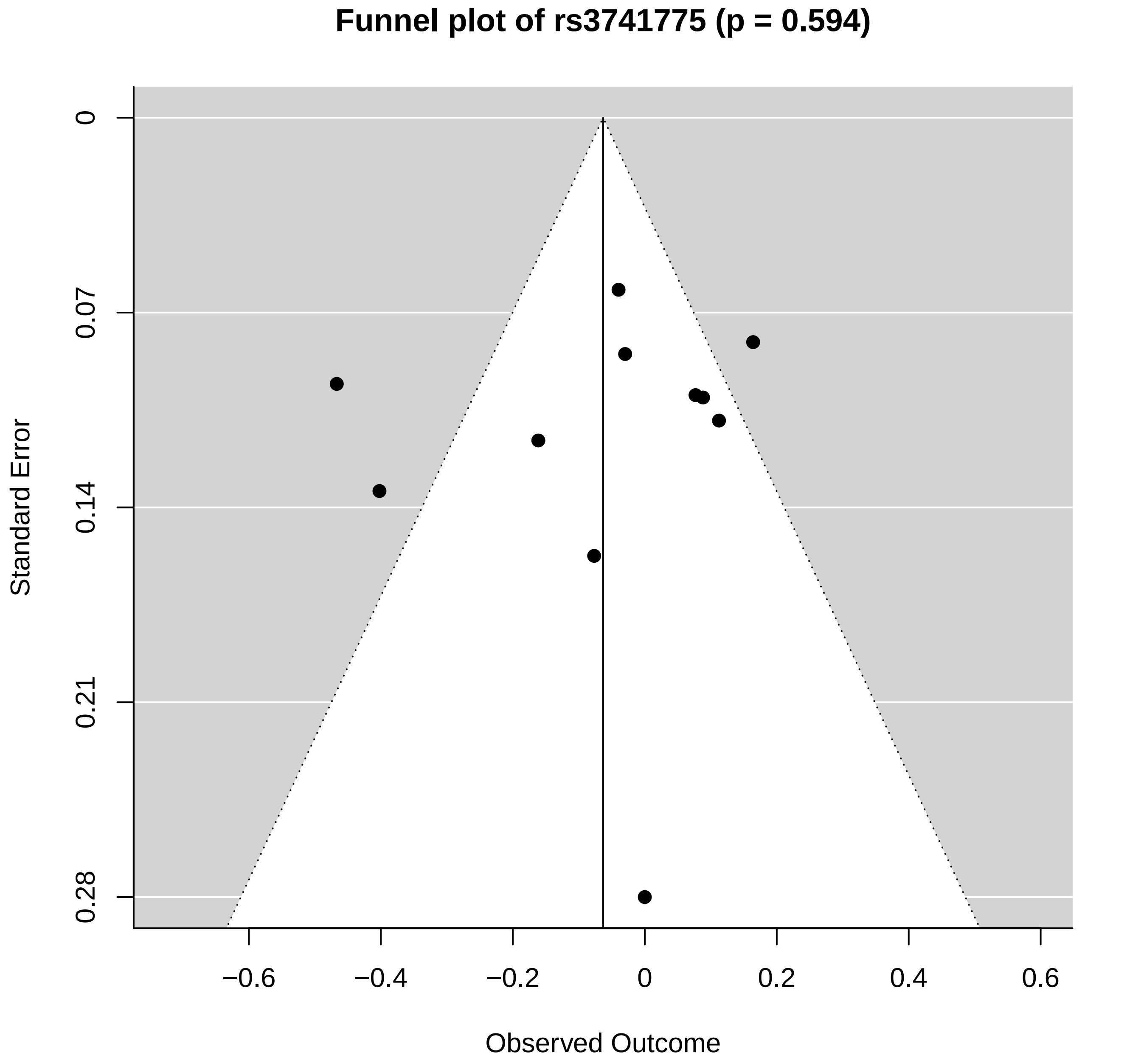

Funnel plot of rs3741775 (p = 0.594)
Standard Error
0
0.07
0.14
0.21
0.28
−0.6
−0.4
−0.2
0
0.2
0.4
0.6
Observed Outcome

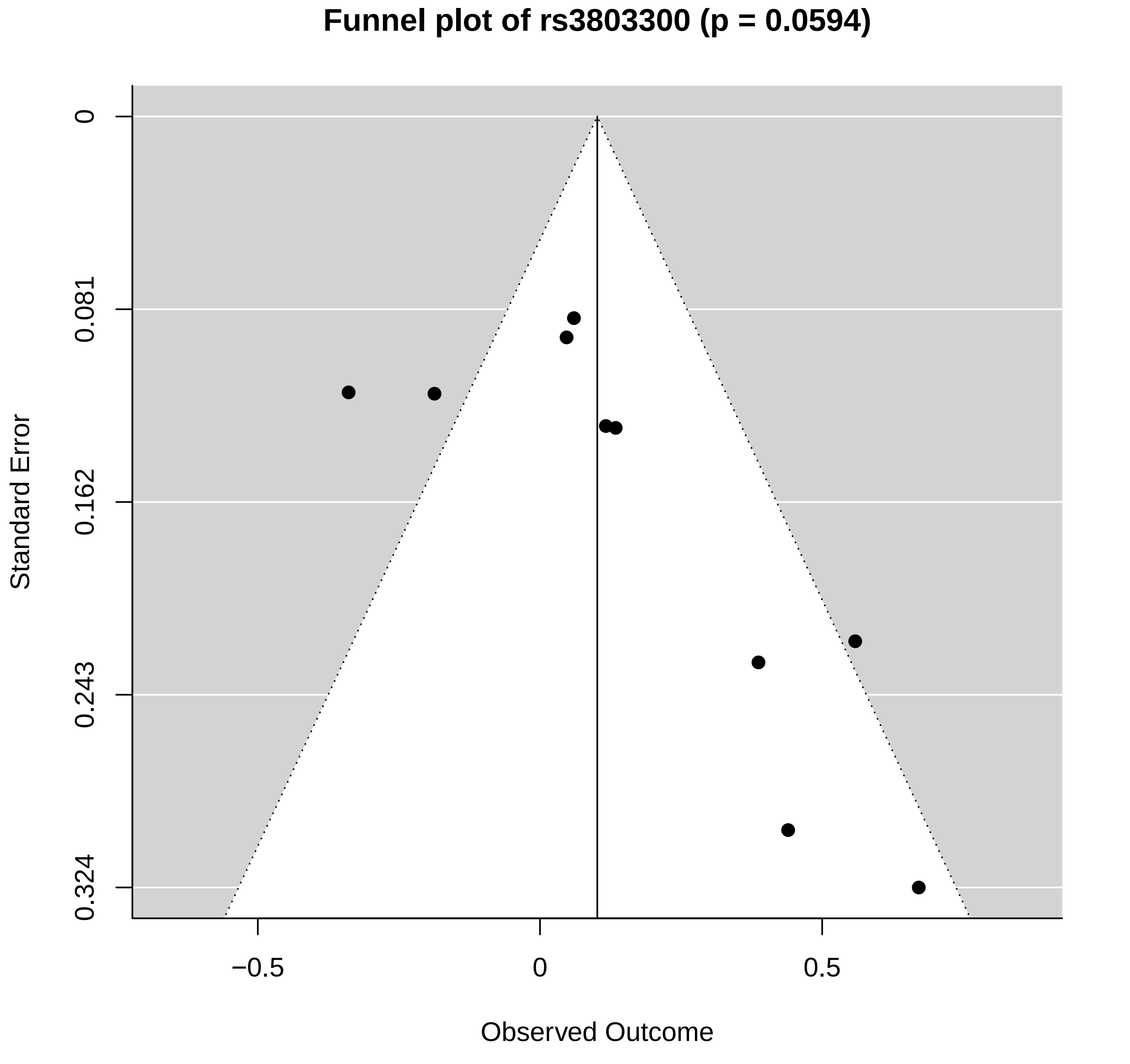

Funnel plot of rs3803300 (p = 0.0594)
Standard Error
0
0.081
0.162
0.243
0.324
−0.5
0
0.5
Observed Outcome

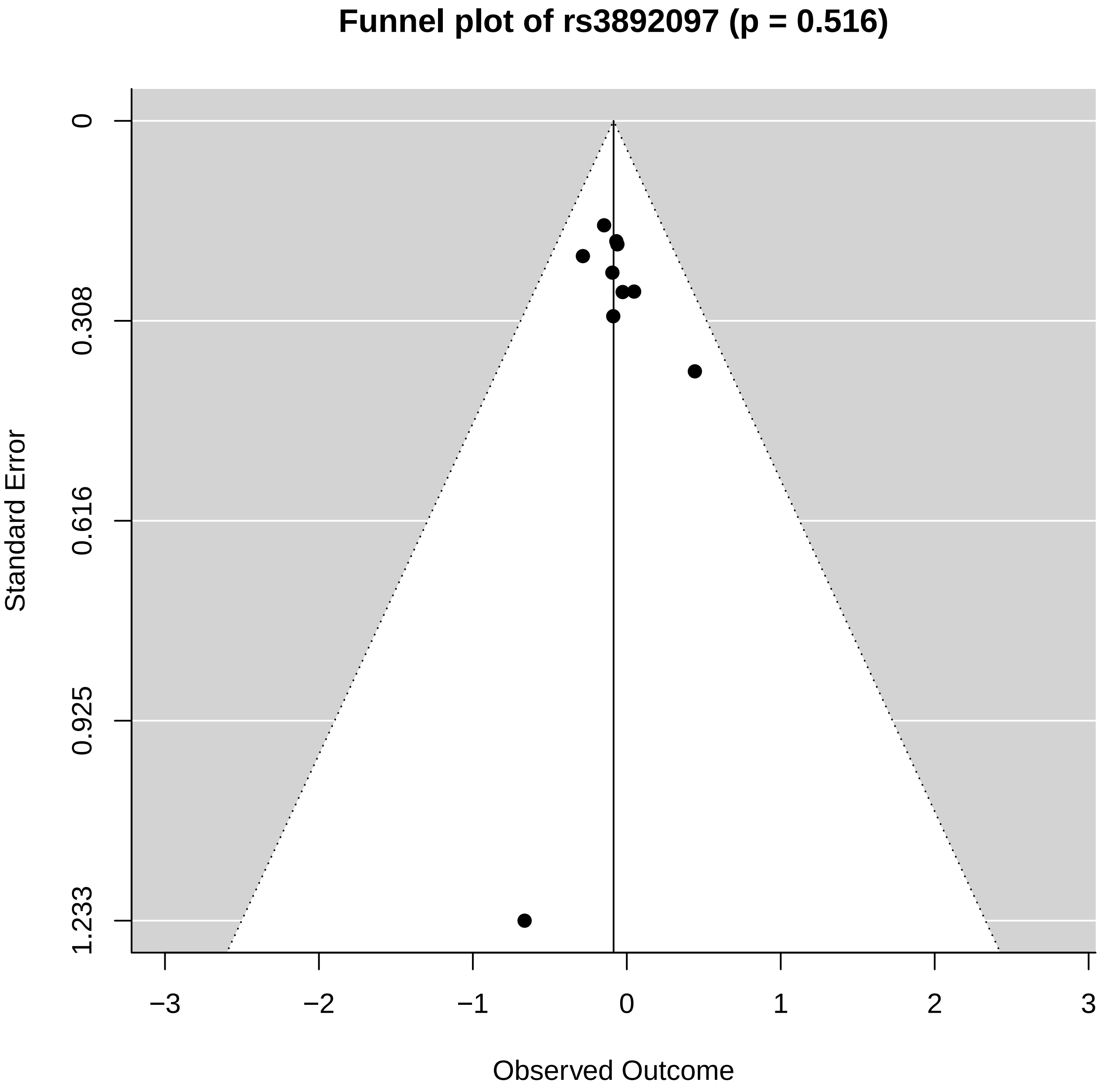
Funnel plot of rs3892097 (p = 0.516)
Standard Error
0
0.308
0.616
0.925
1.233
−3
−2
−1
0
1
2
3
Observed Outcome

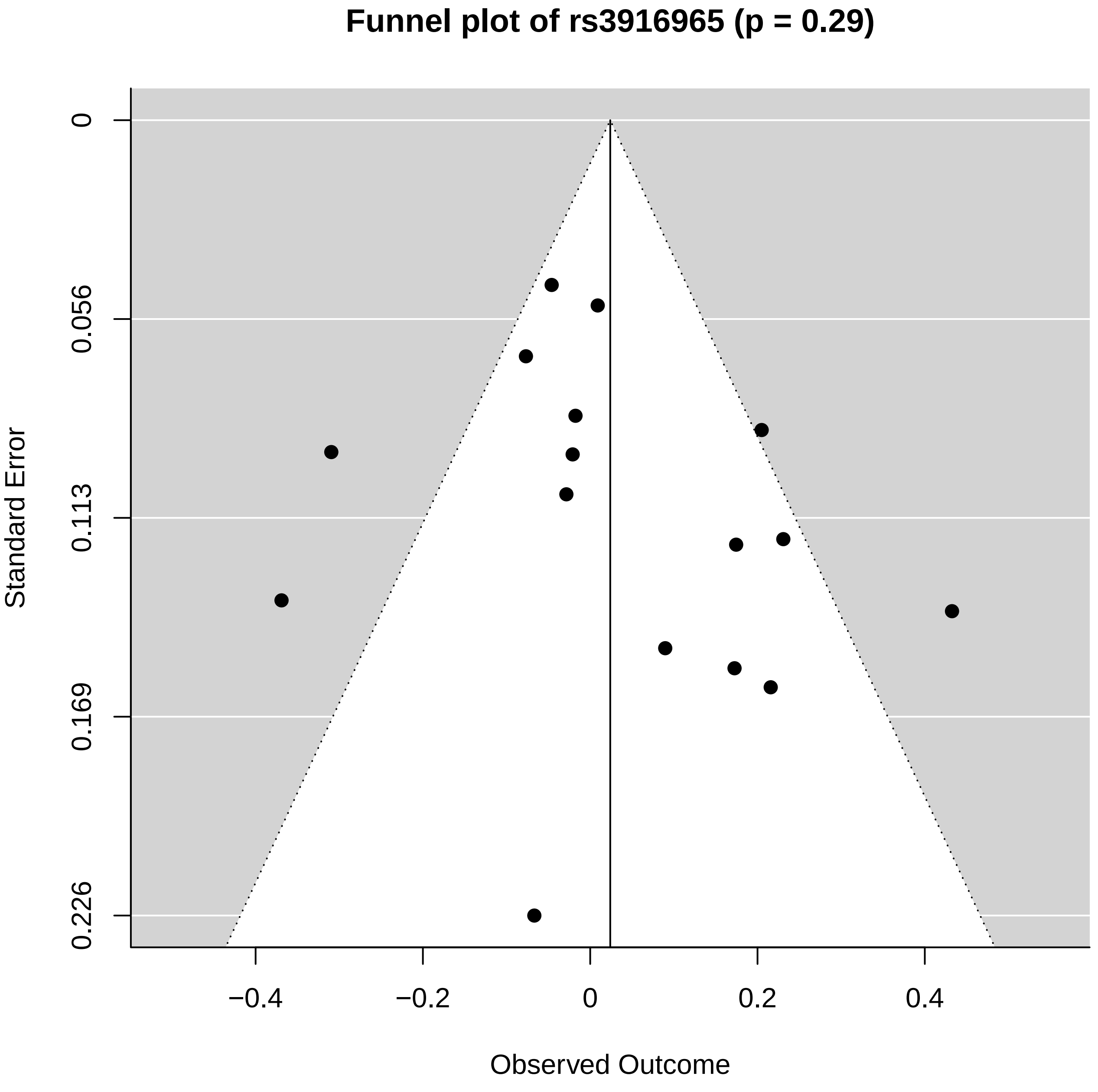

Funnel plot of rs3916965 (p = 0.29)
Standard Error
0
0.056
0.113
0.169
0.226
−0.4
−0.2
0
0.2
0.4
Observed Outcome

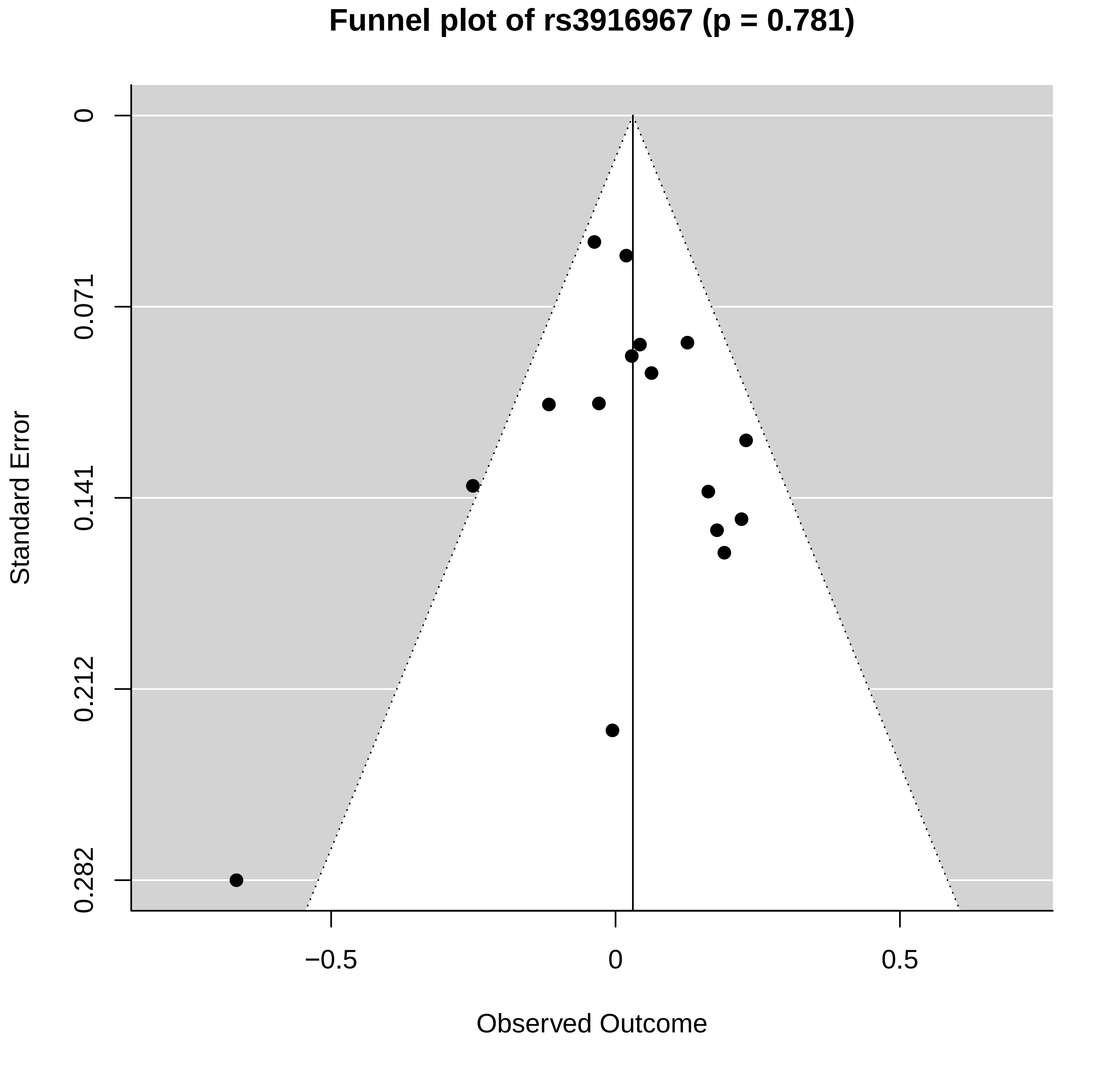
Funnel plot of rs3916967 (p = 0.781)
Standard Error
0
0.071
0.141
0.212
0.282
−0.5
0
0.5
Observed Outcome

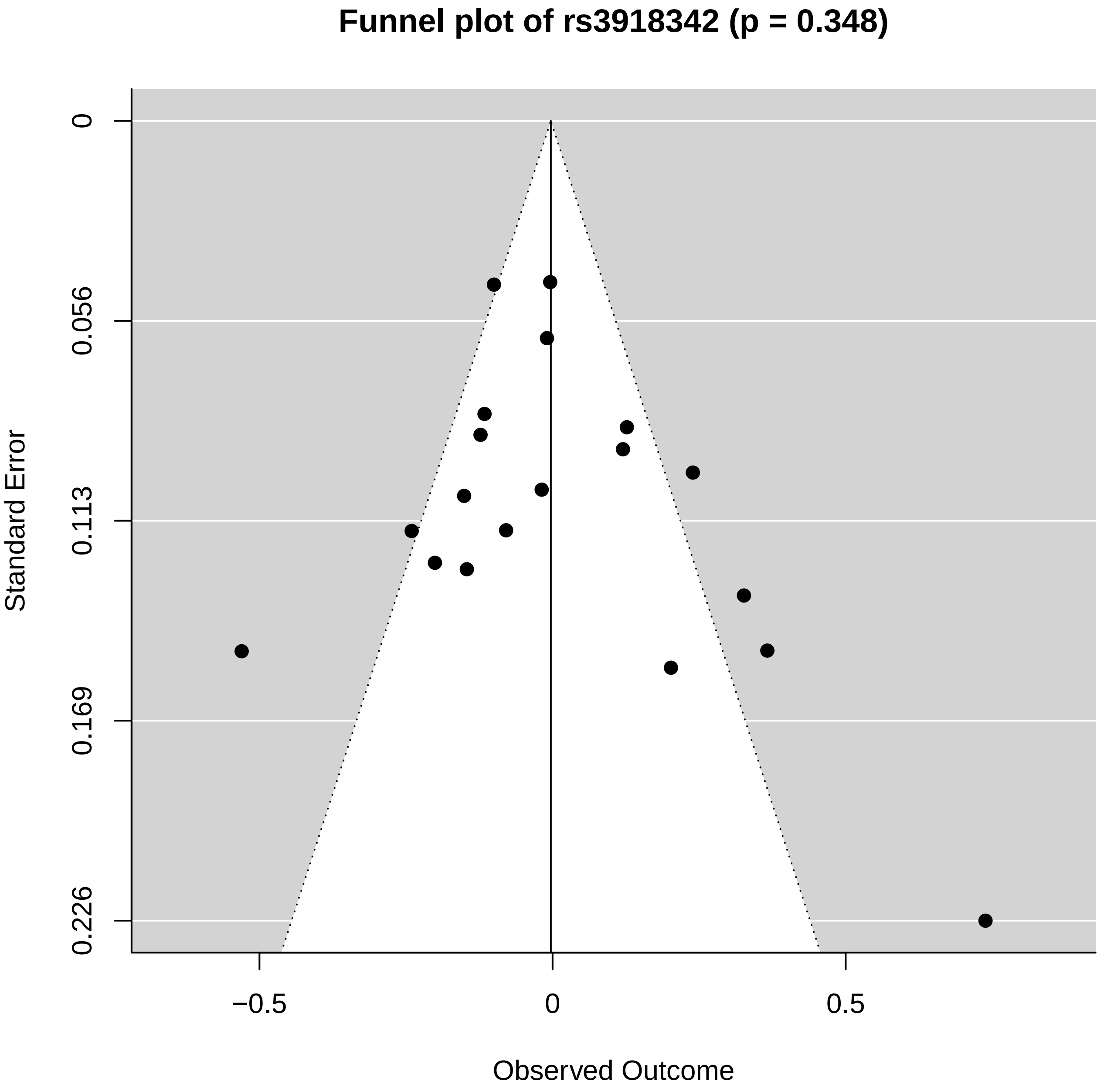

Funnel plot of rs3918342 (p = 0.348)
0
0.056
0.113
0.169
0.226
Standard Error
−0.5
0
0.5
Observed Outcome

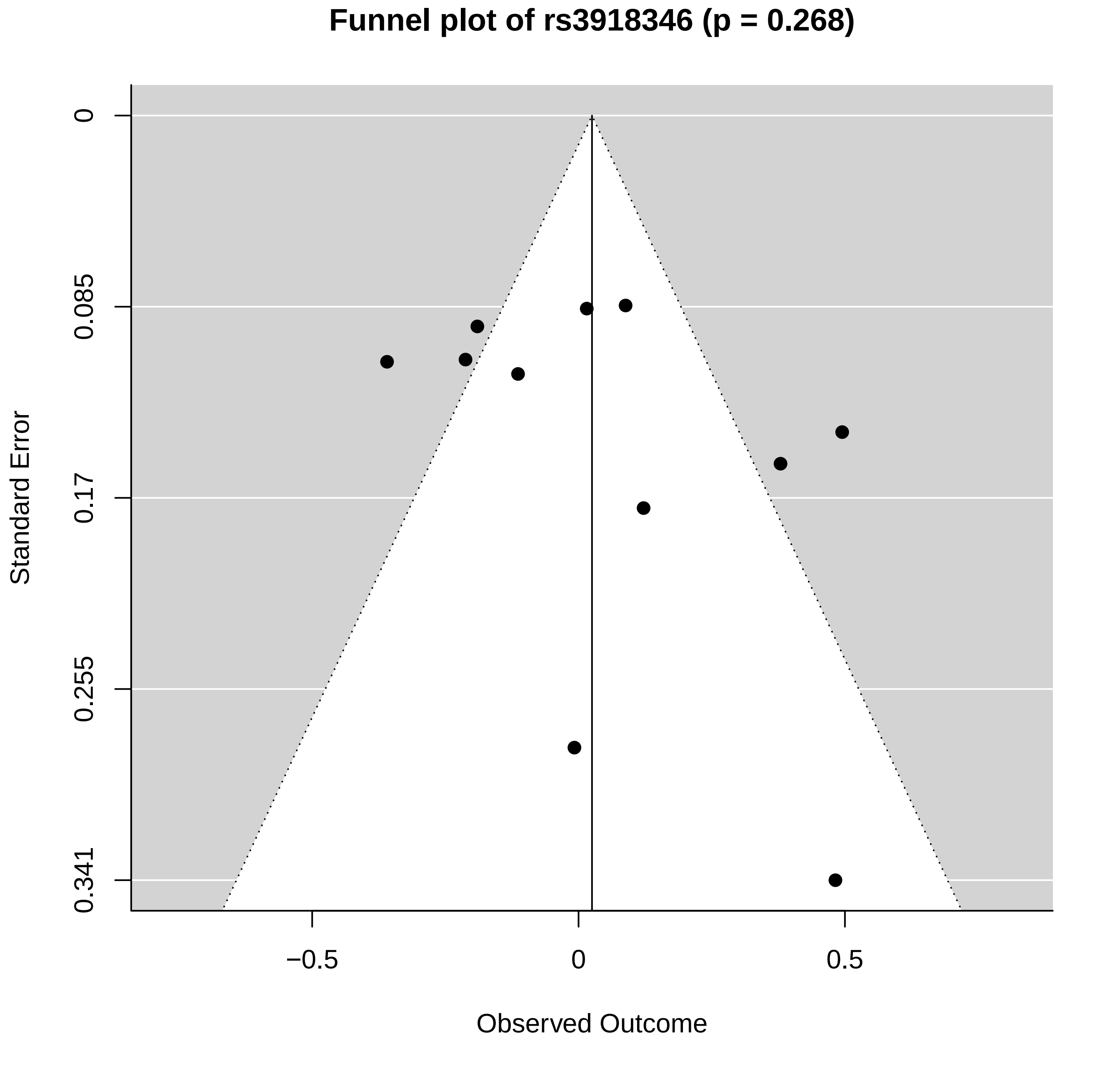
Funnel plot of rs3918346 (p = 0.268)
Standard Error
0
0.085
0.17
0.255
0.341
−0.5
0
0.5
Observed Outcome

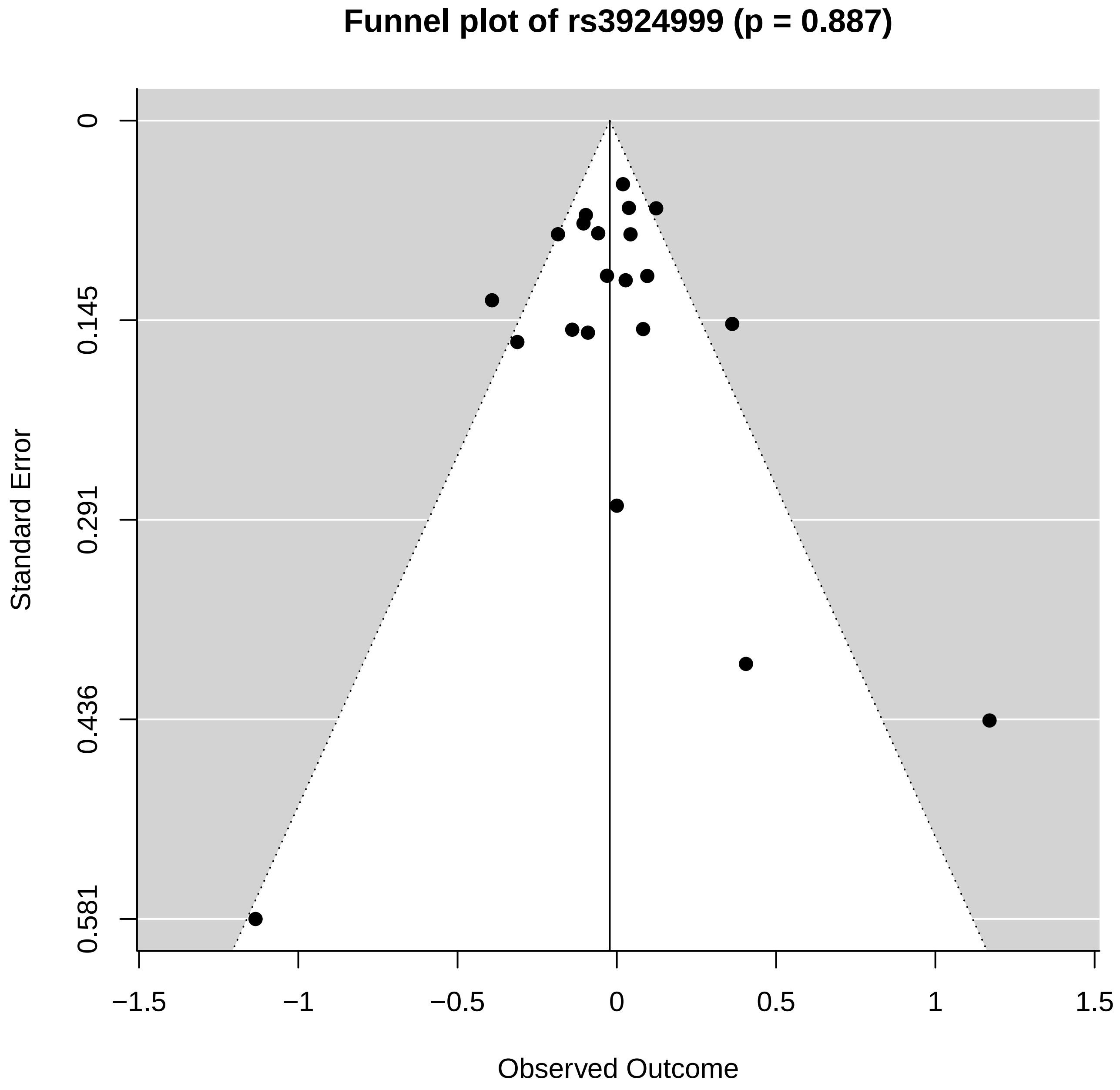

Funnel plot of rs3924999 (p = 0.887)
Standard Error
0
0.145
0.291
0.436
0.581
−1.5
−1
−0.5
0
0.5
1
1.5
Observed Outcome

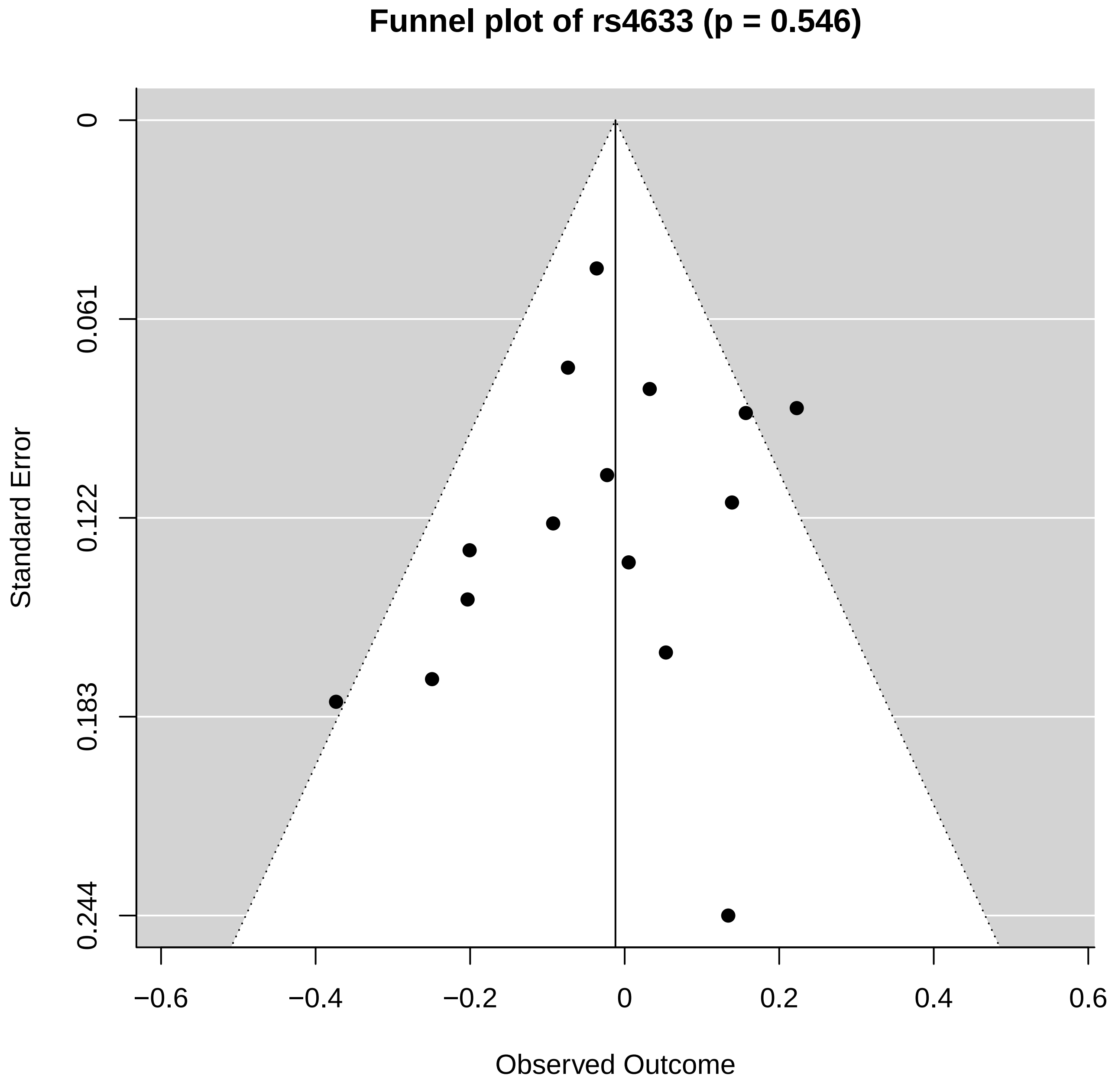
Funnel plot of rs4633 (p = 0.546)
Standard Error
0
0.061
0.122
0.183
0.244
−0.6
−0.4
−0.2
0
0.2
0.4
0.6
Observed Outcome

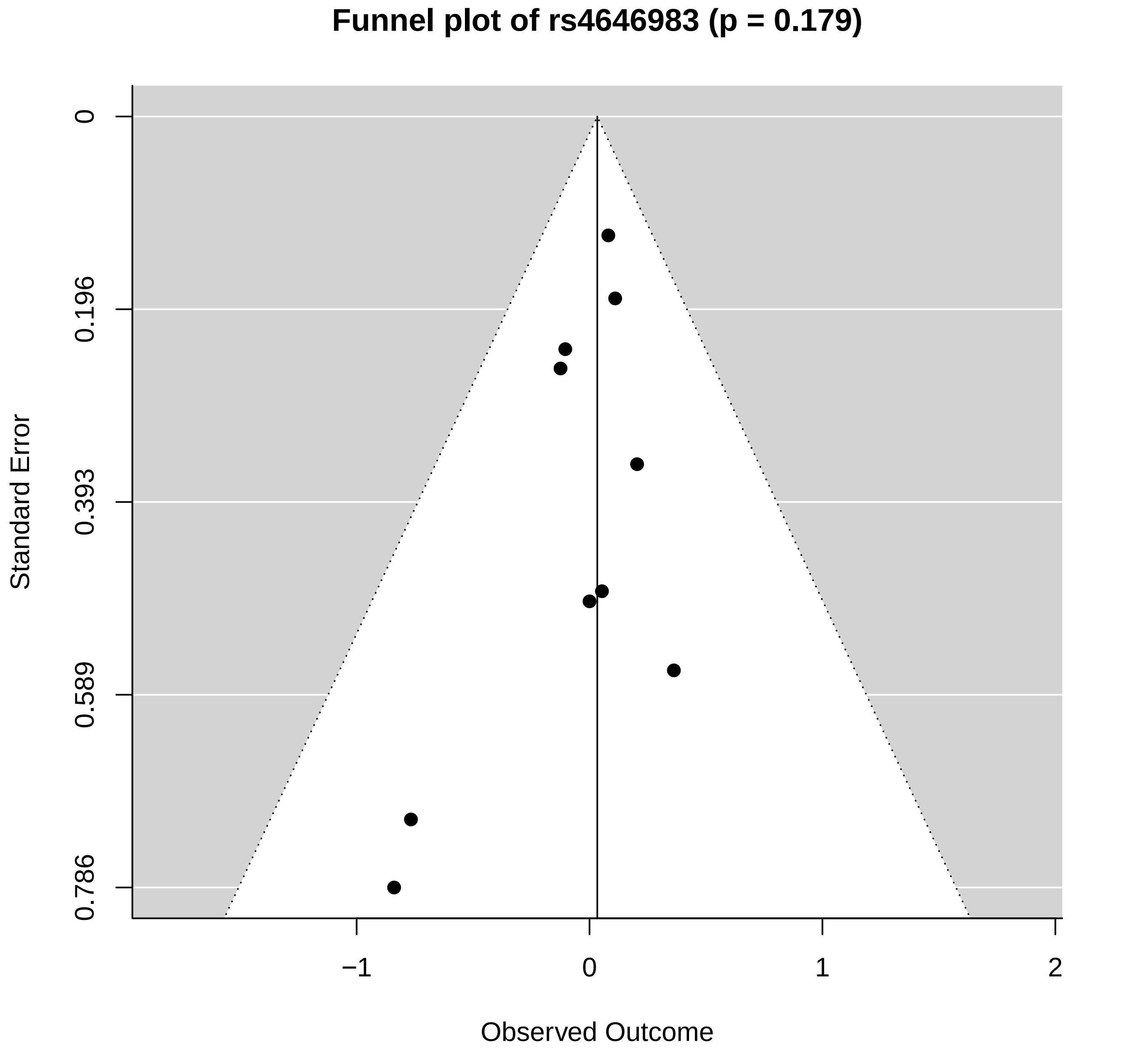

Funnel plot of rs4646983 (p = 0.179)
Standard Error
0
0.196
0.393
0.589
0.786
−1
0
1
2
Observed Outcome

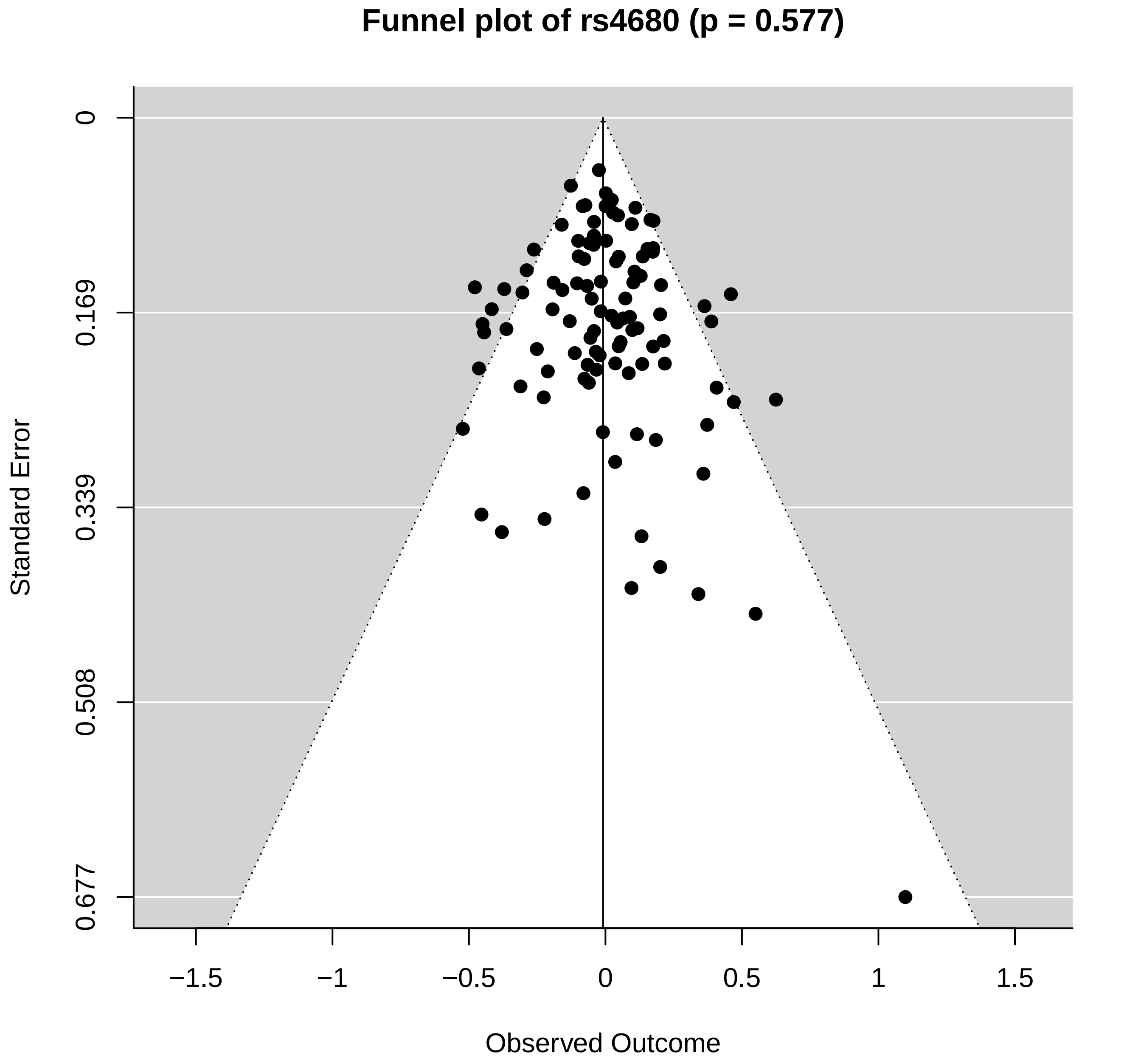
Funnel plot of rs4680 (p = 0.577)
Standard Error
0
0.169
0.339
0.508
0.677
−1.5
−1
−0.5
0
0.5
1
1.5
Observed Outcome

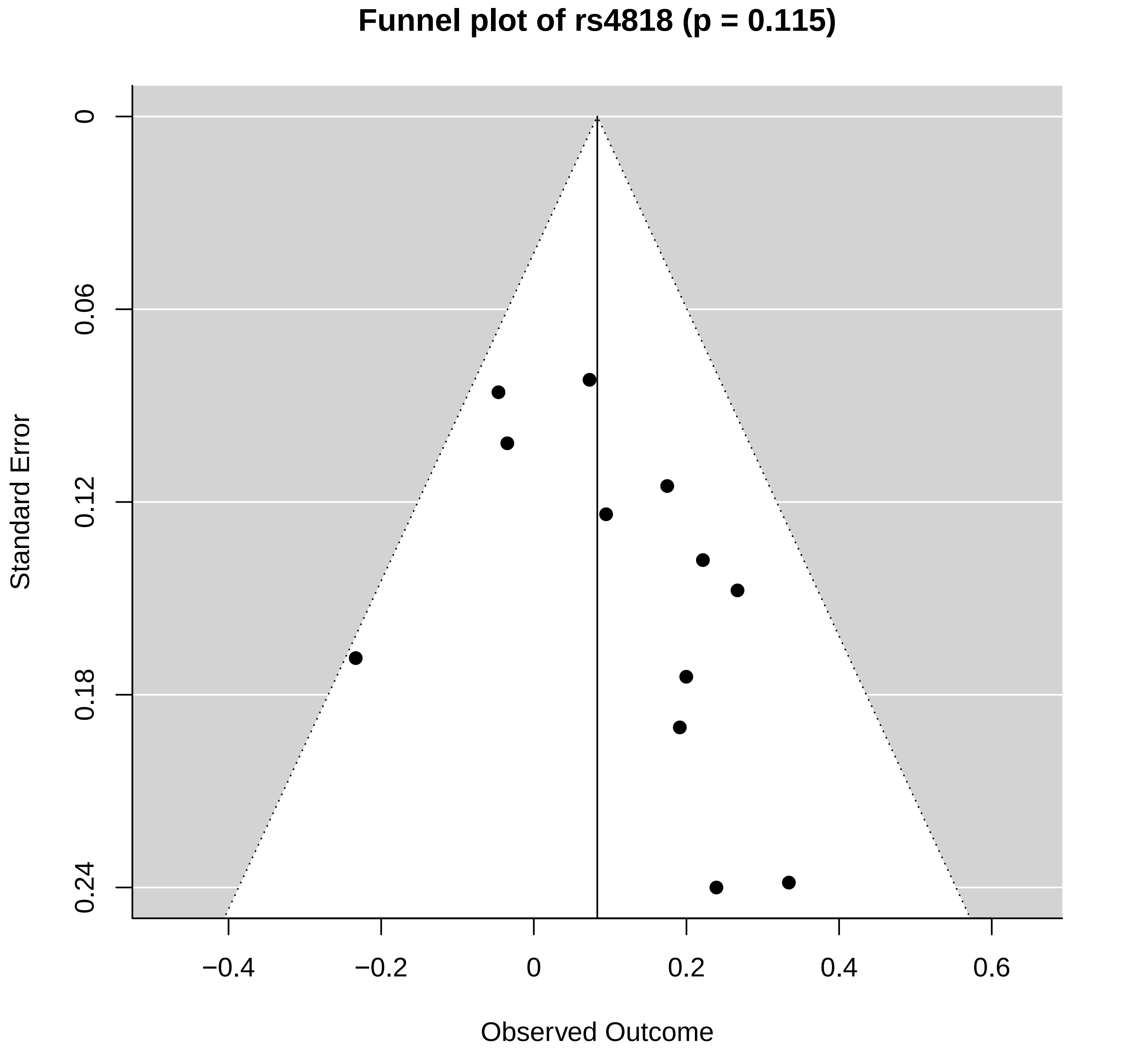
Funnel plot of rs4818 (p = 0.115)
Standard Error
0
0.06
0.12
0.18
0.24
−0.4
−0.2
0
0.2
0.4
0.6
Observed Outcome

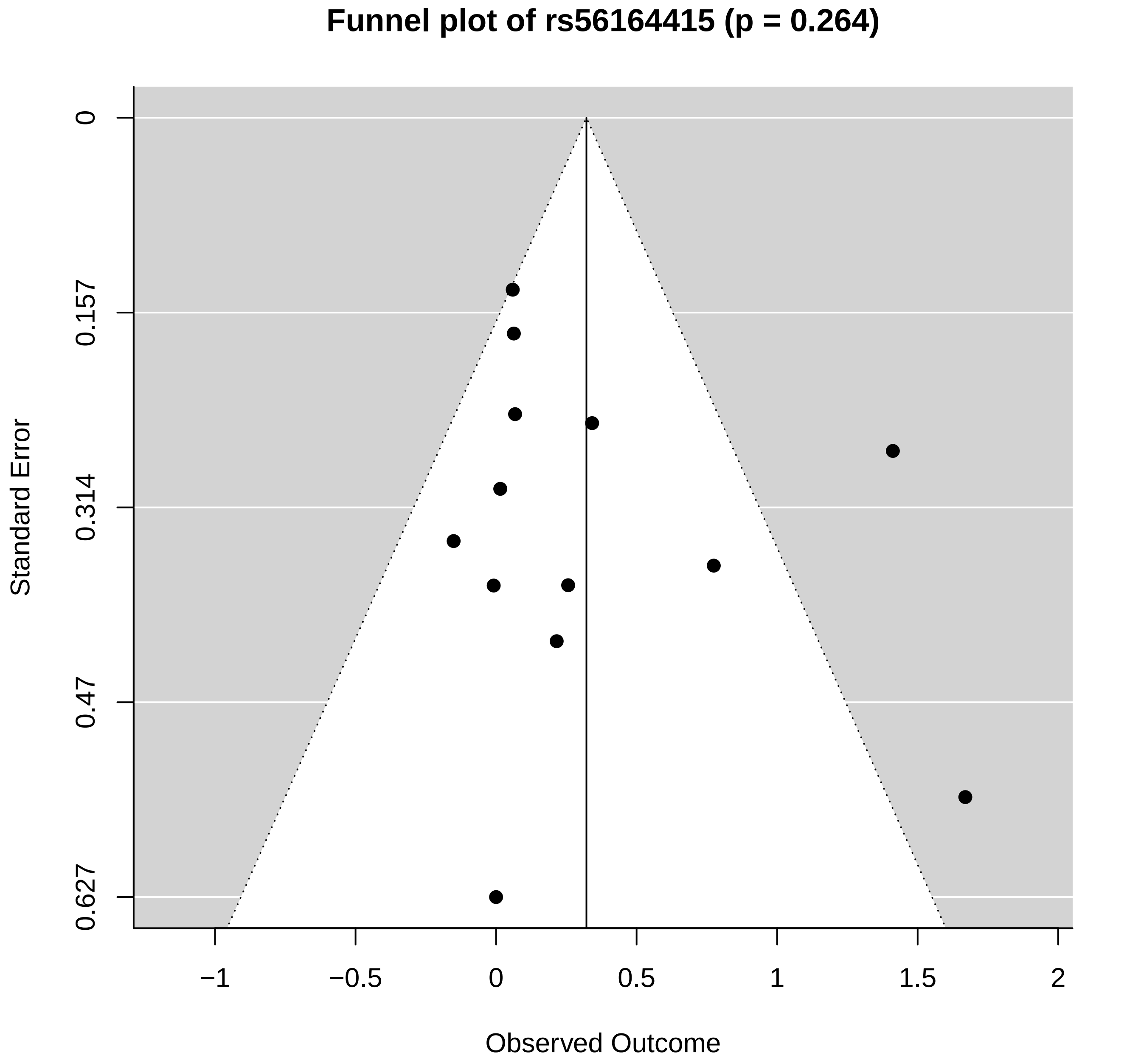
Funnel plot of rs56164415 (p = 0.264)
Standard Error
0
0.157
0.314
0.47
0.627
−1
−0.5
0
0.5
1
1.5
2
Observed Outcome

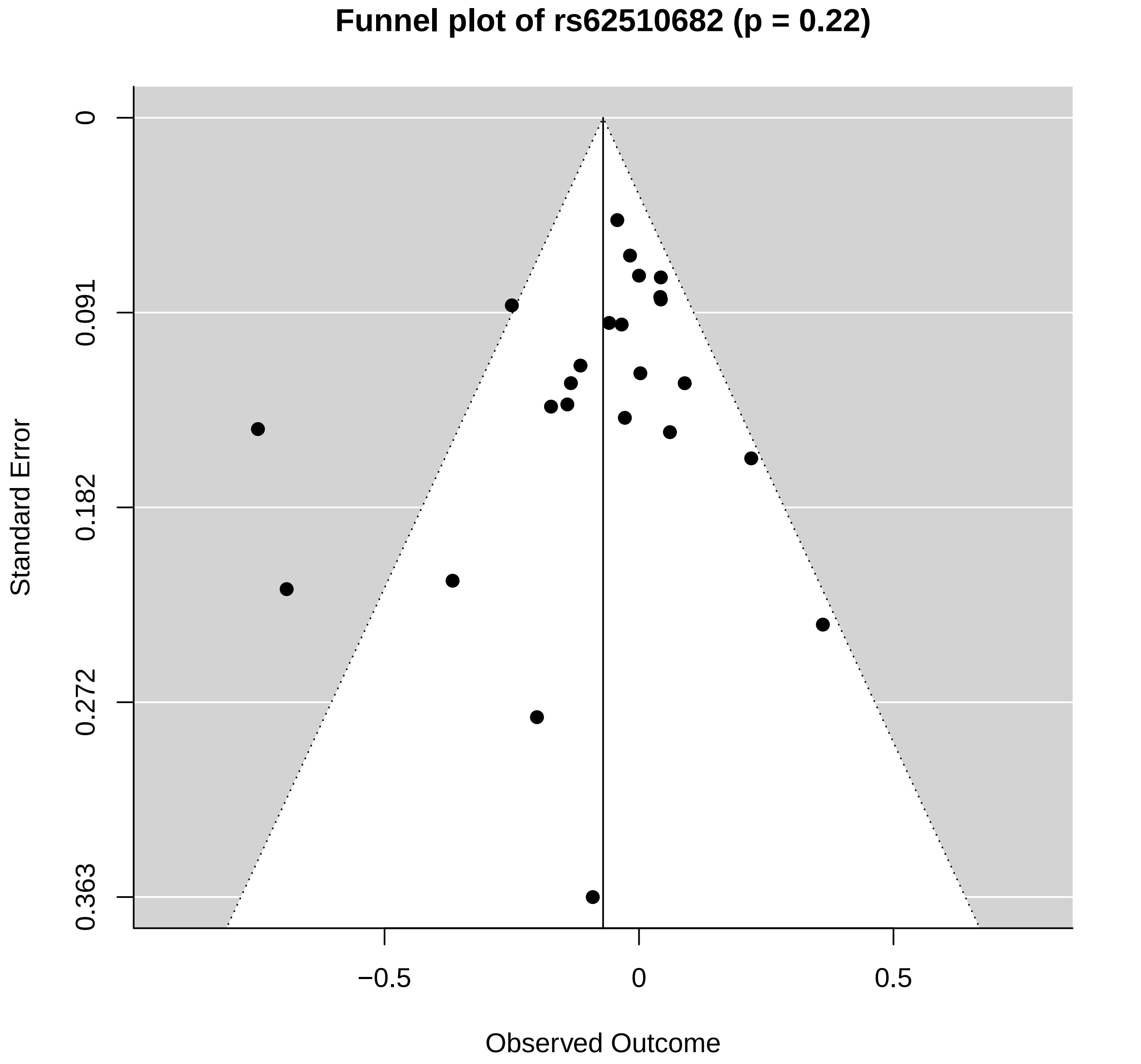

Funnel plot of rs62510682 (p = 0.22)
Standard Error
0
0.091
0.182
0.272
0.363
−0.5
0
0.5
Observed Outcome

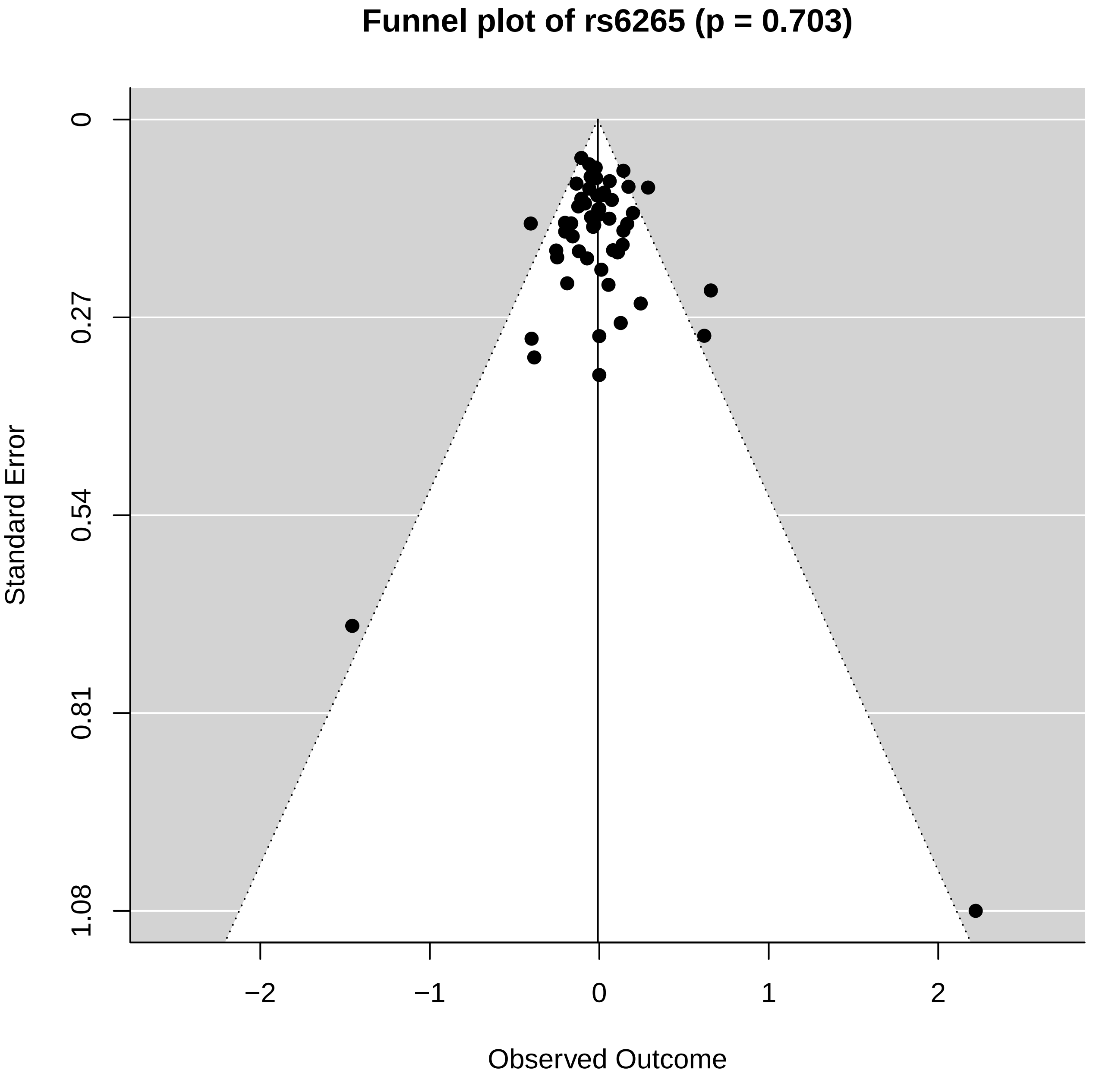

Funnel plot of rs6265 (p = 0.703)
Standard Error
0
0.27
0.54
0.81
1.08
−2
−1
0
1
2
Observed Outcome

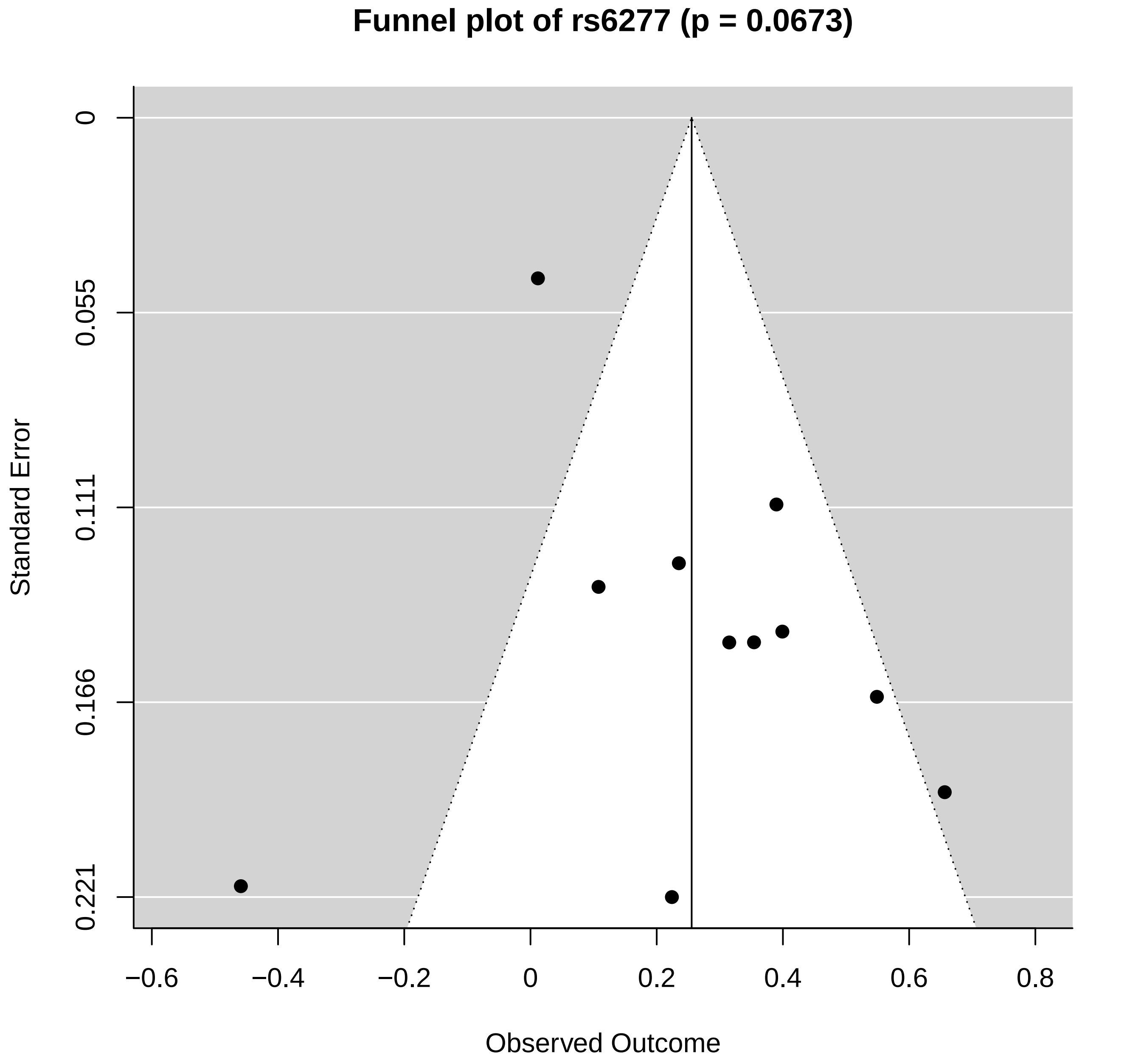

Funnel plot of rs6277 (p = 0.0673)
Standard Error
0
0.055
0.111
0.166
0.221
−0.6
−0.4
−0.2
0
0.2
0.4
0.6
0.8
Observed Outcome

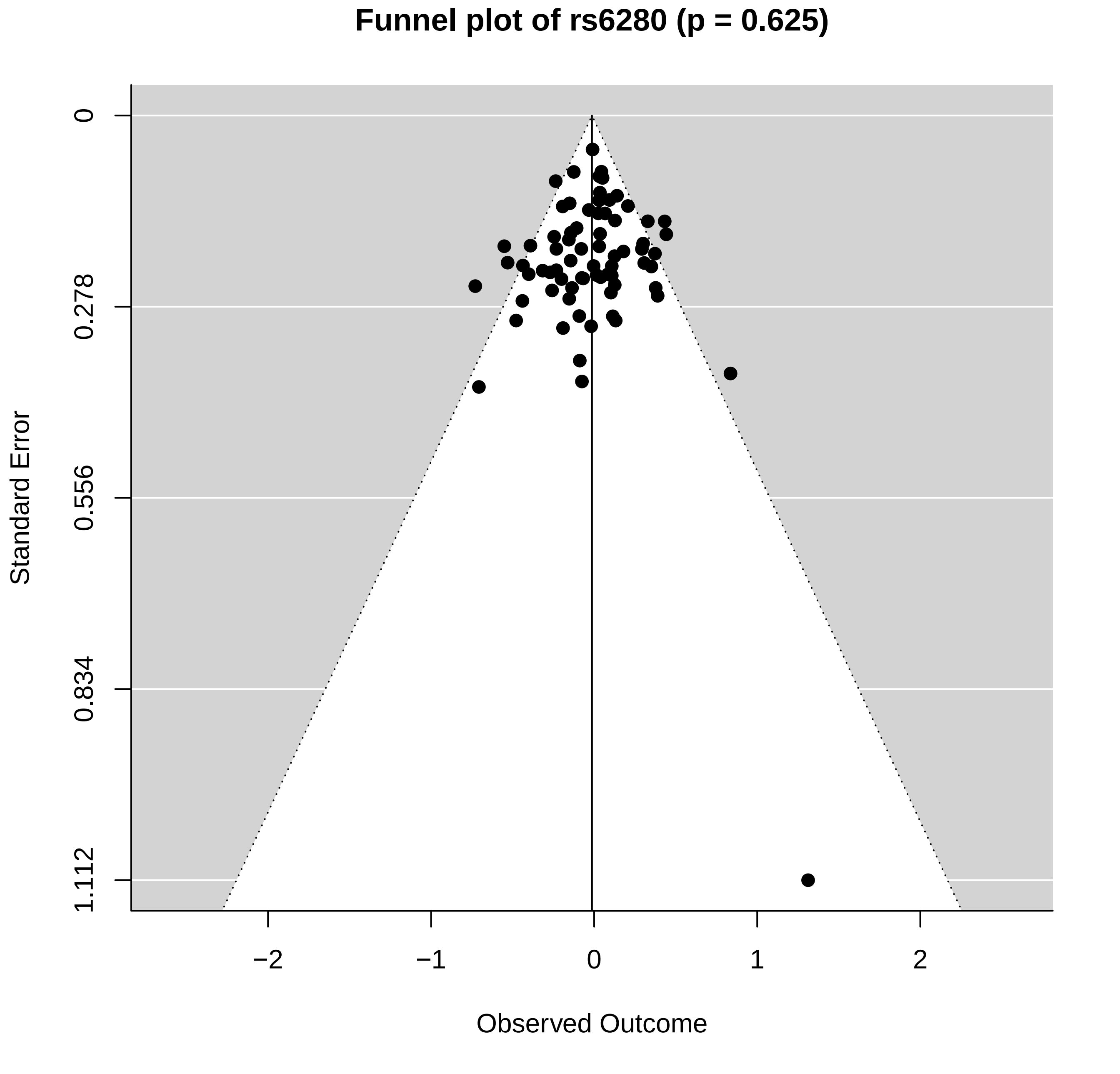

Funnel plot of rs6280 (p = 0.625)
Standard Error
0
0.278
0.556
0.834
1.112
−2
−1
0
1
2
Observed Outcome

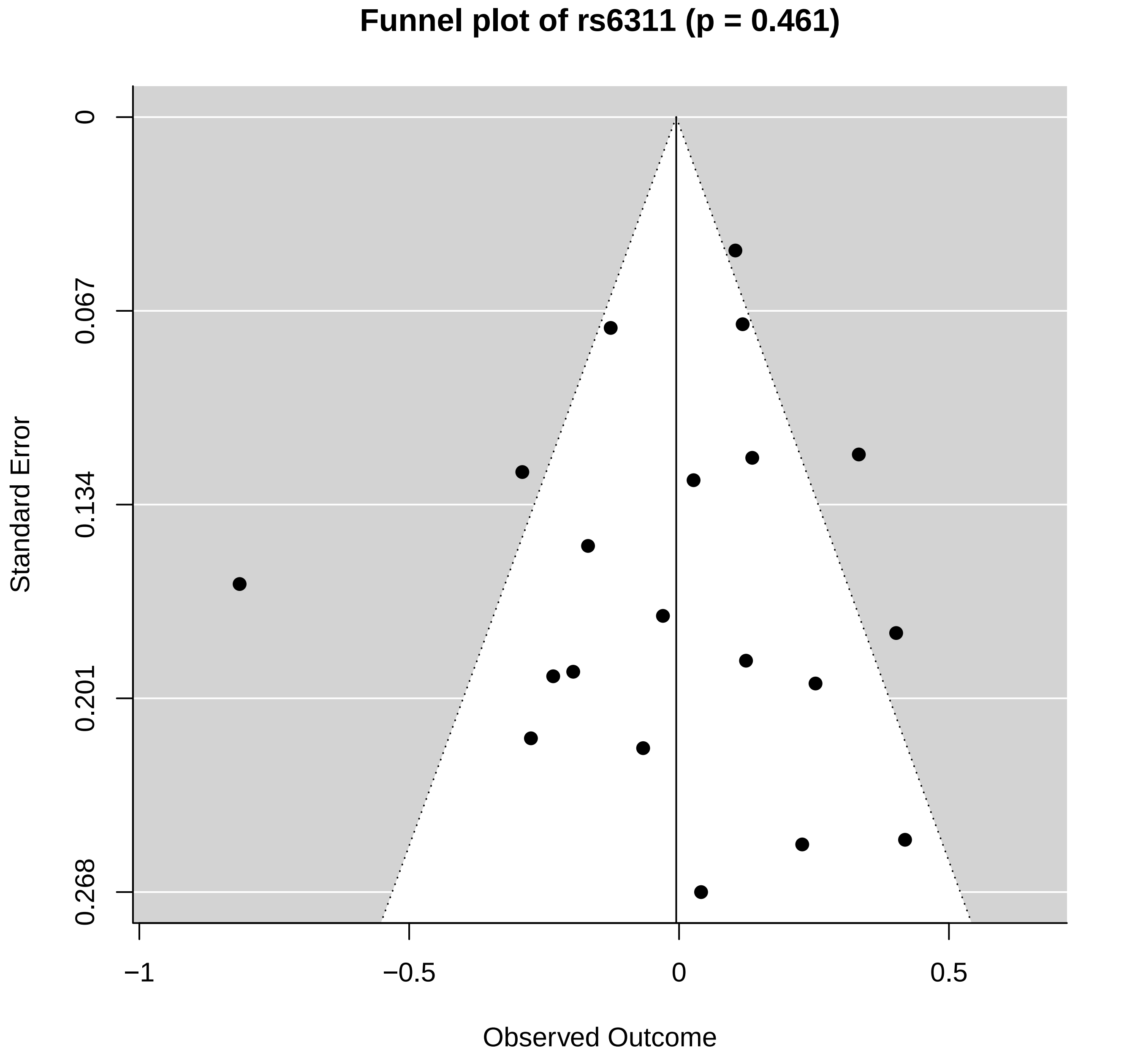
Funnel plot of rs6311 (p = 0.461)
Standard Error
0
0.067
0.134
0.201
0.268
−1
−0.5
0
0.5
Observed Outcome

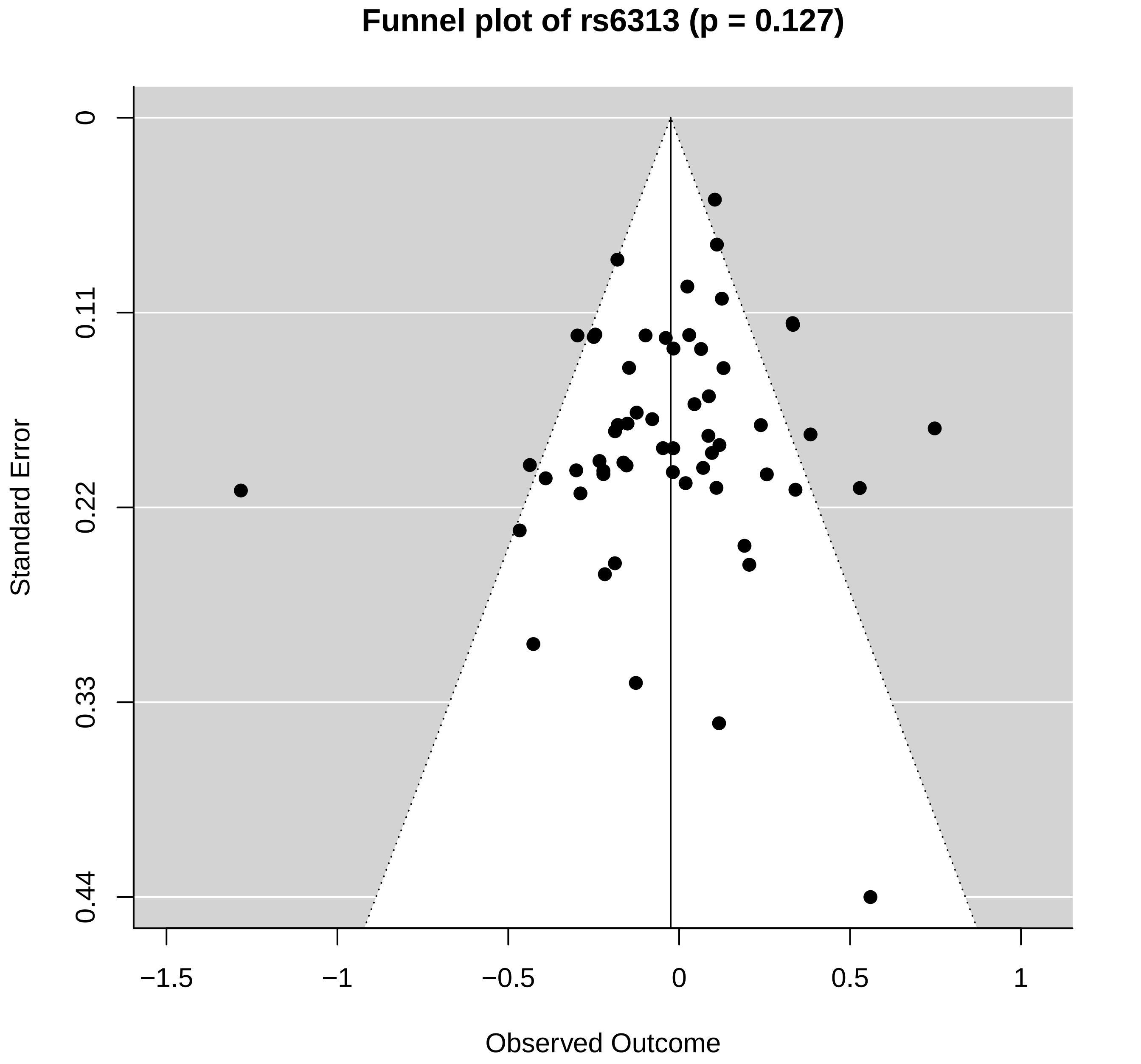

Funnel plot of rs6313 (p = 0.127)
Standard Error
0
0.11
0.22
0.33
0.44
−1.5
−1
−0.5
0
0.5
1
Observed Outcome

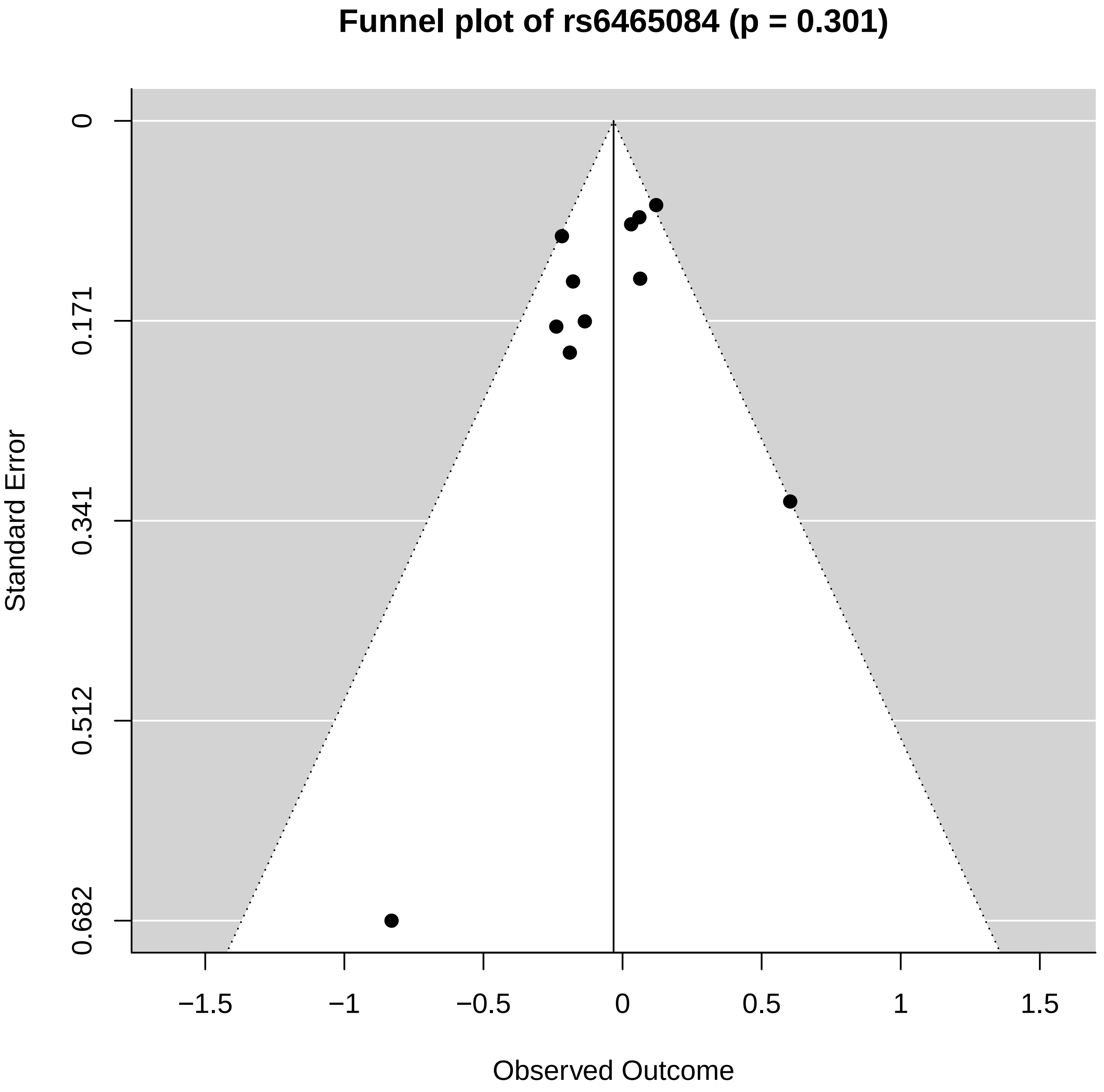
Funnel plot of rs6465084 (p = 0.301)
Standard Error
0
0.171
0.341
0.512
0.682
Observed Outcome
−1.5
−1
−0.5
0
0.5
1
1.5

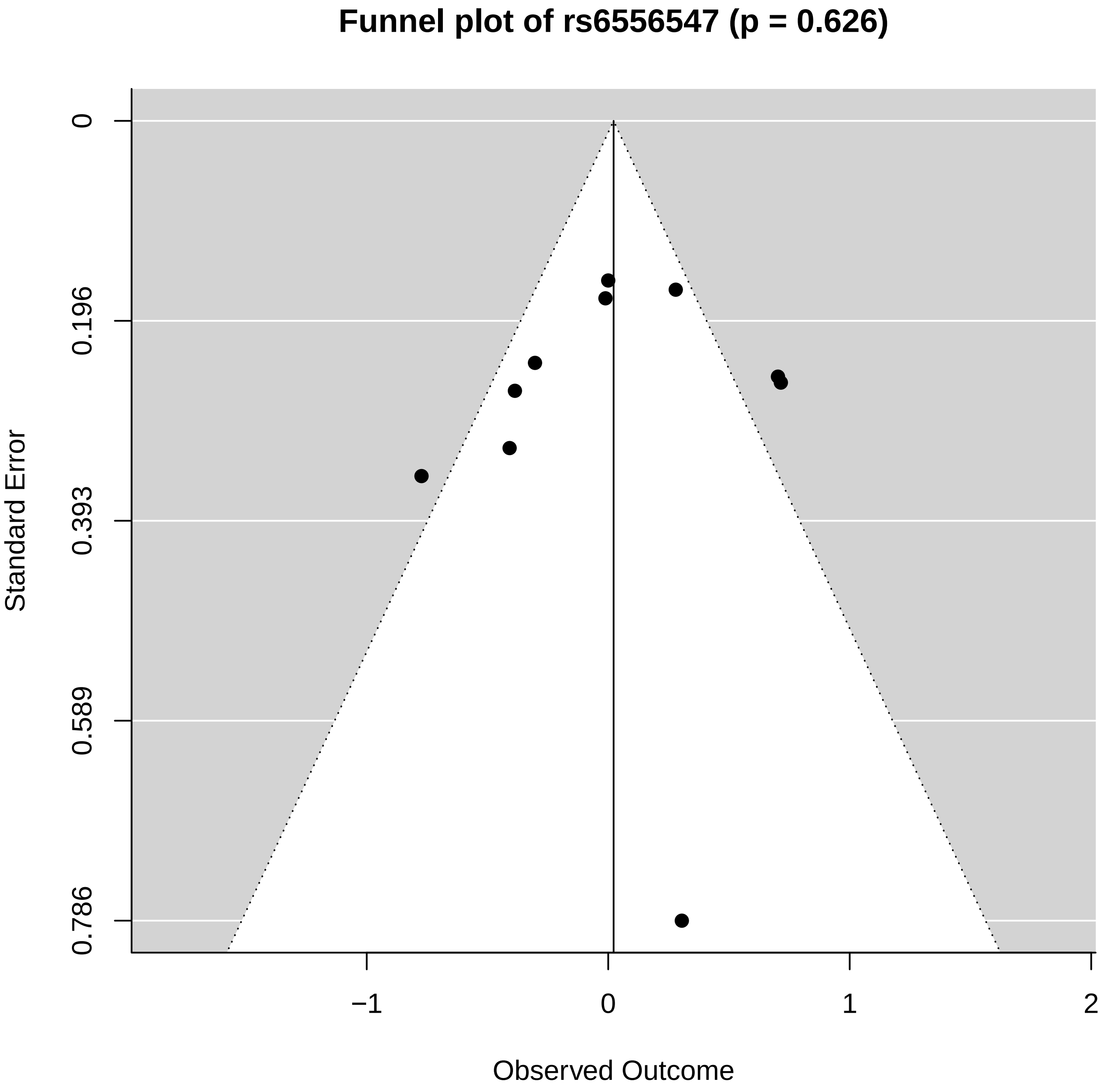

Funnel plot of rs6556547 (p = 0.626)
Standard Error
0
0.196
0.393
0.589
0.786
−1
0
1
2
Observed Outcome

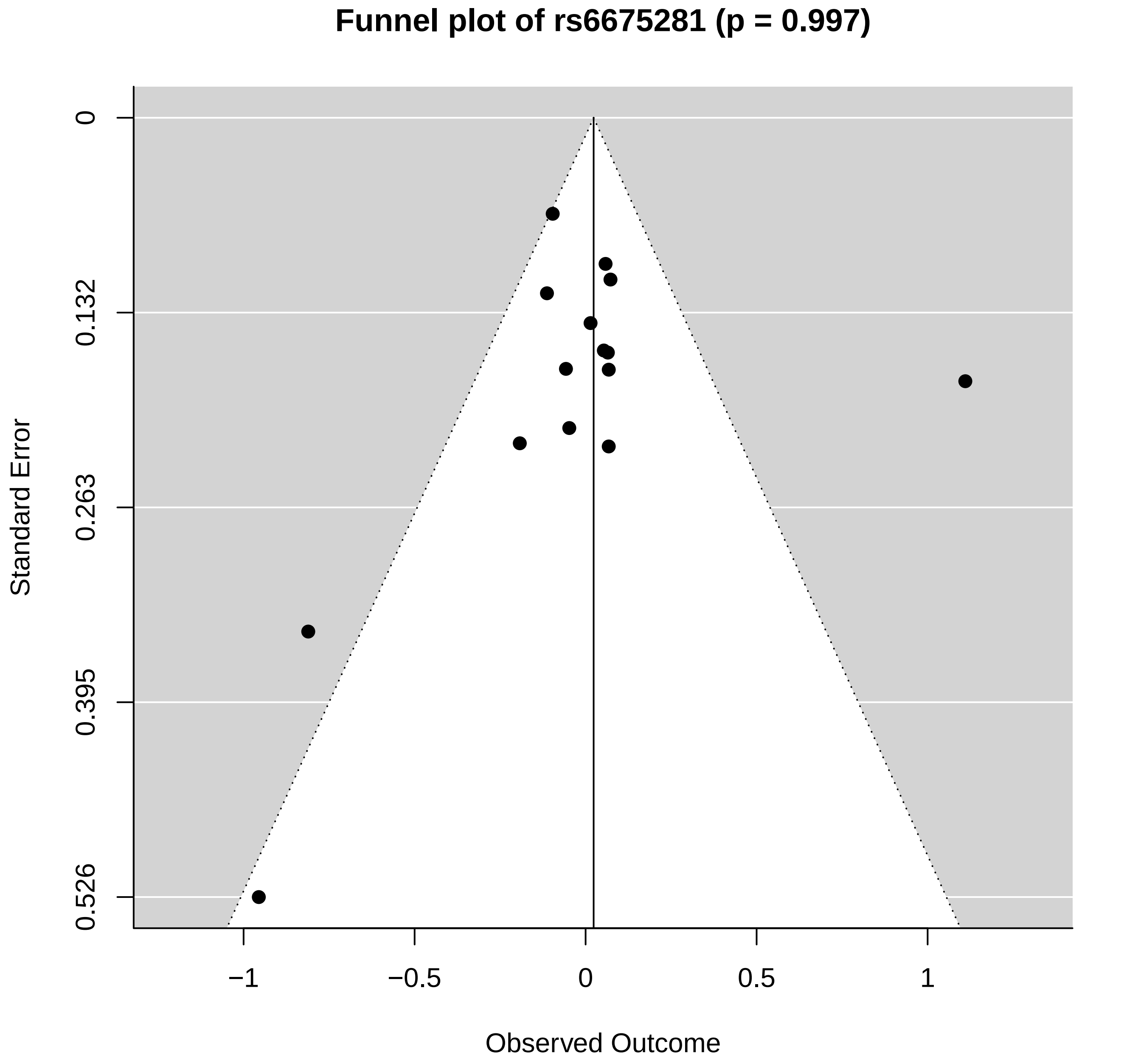

Funnel plot of rs6675281 (p = 0.997)
Standard Error
0
0.132
0.263
0.395
0.526
Observed Outcome
−1
−0.5
0
0.5
1

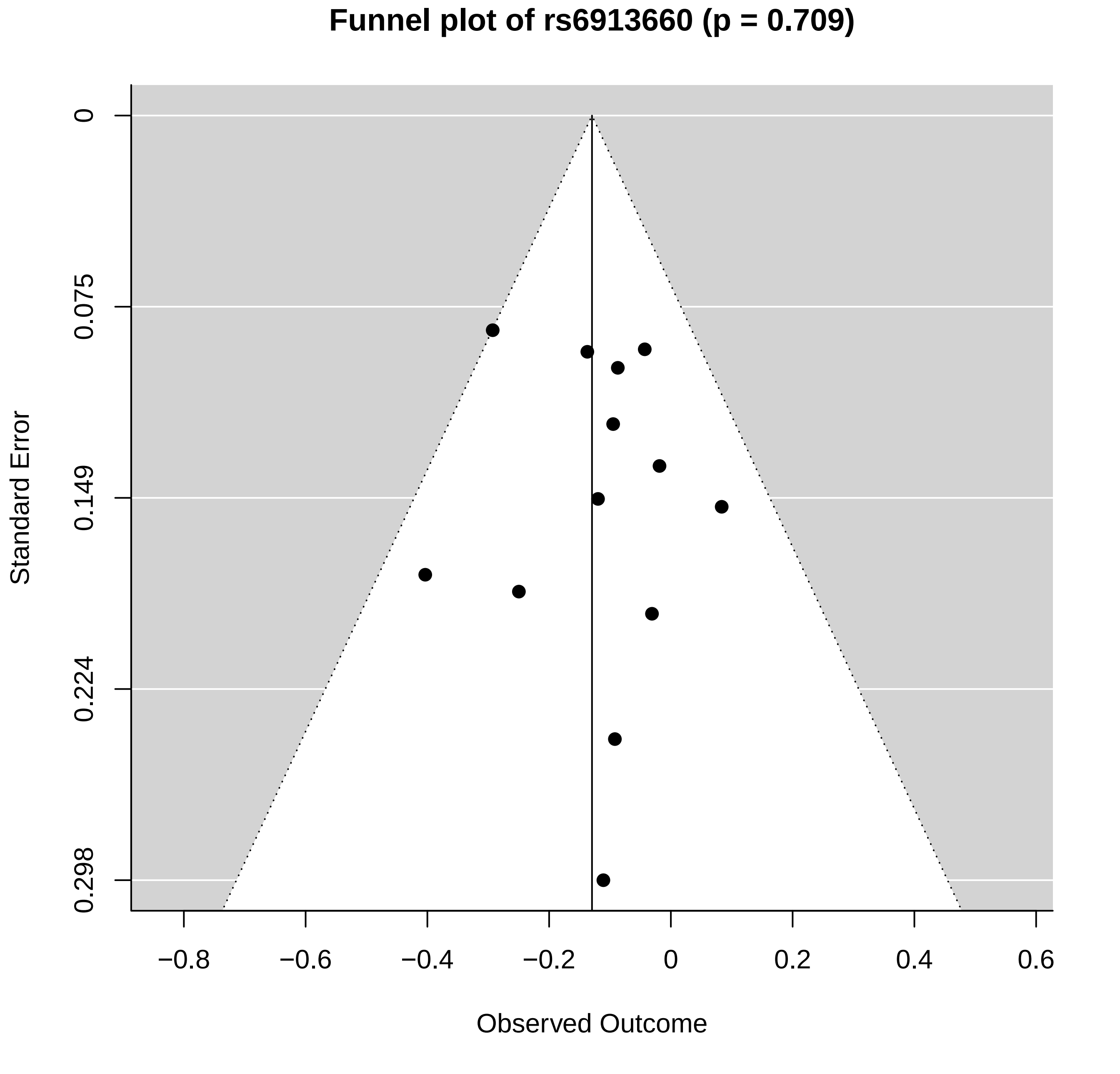
Funnel plot of rs6913660 (p = 0.709)
Standard Error
0
0.075
0.149
0.224
0.298
−0.8
−0.6
−0.4
−0.2
0
0.2
0.4
0.6
Observed Outcome

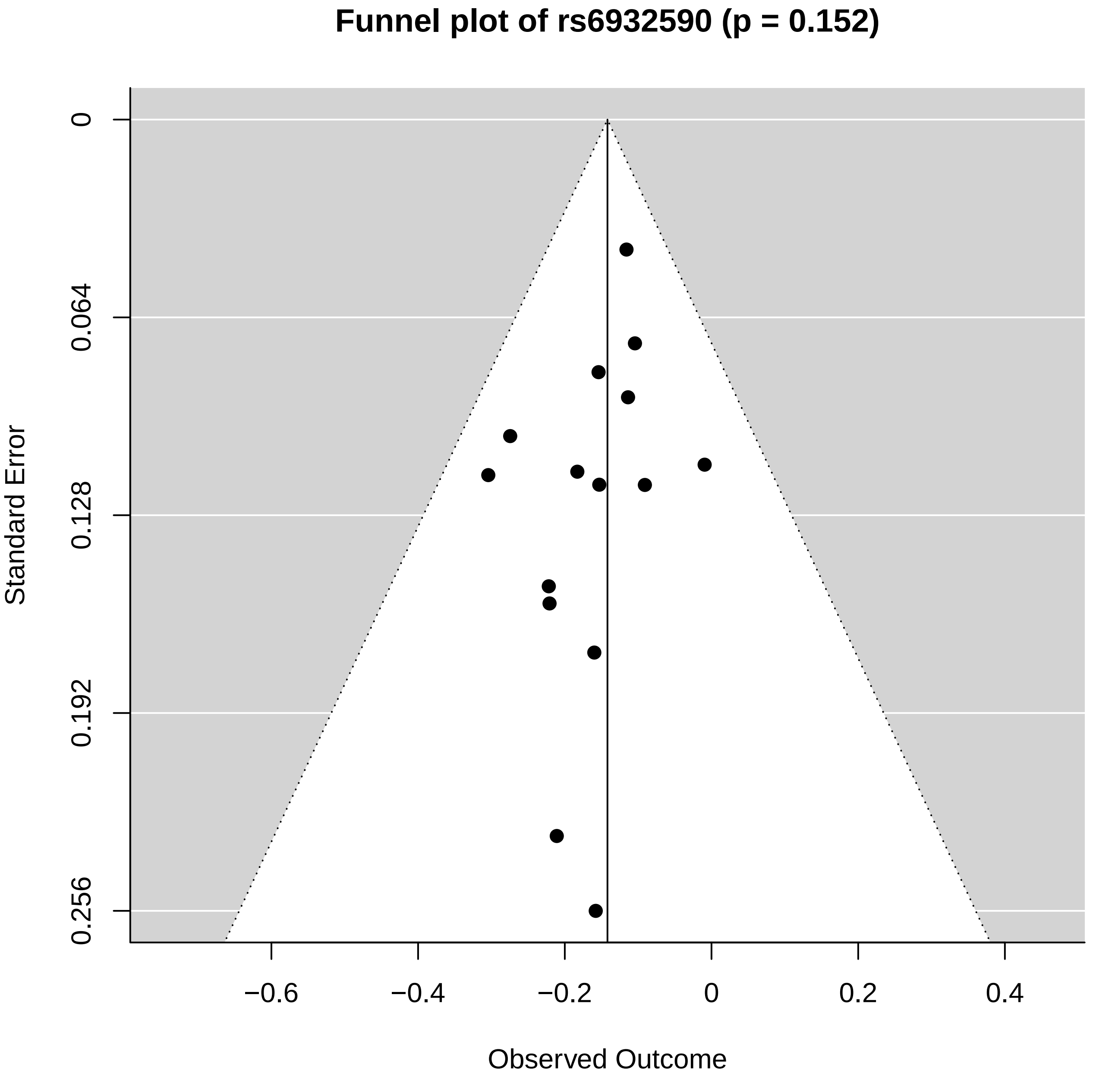
Funnel plot of rs6932590 (p = 0.152)
Standard Error
0
0.064
0.128
0.192
0.256
−0.6
−0.4
−0.2
0
0.2
0.4
Observed Outcome

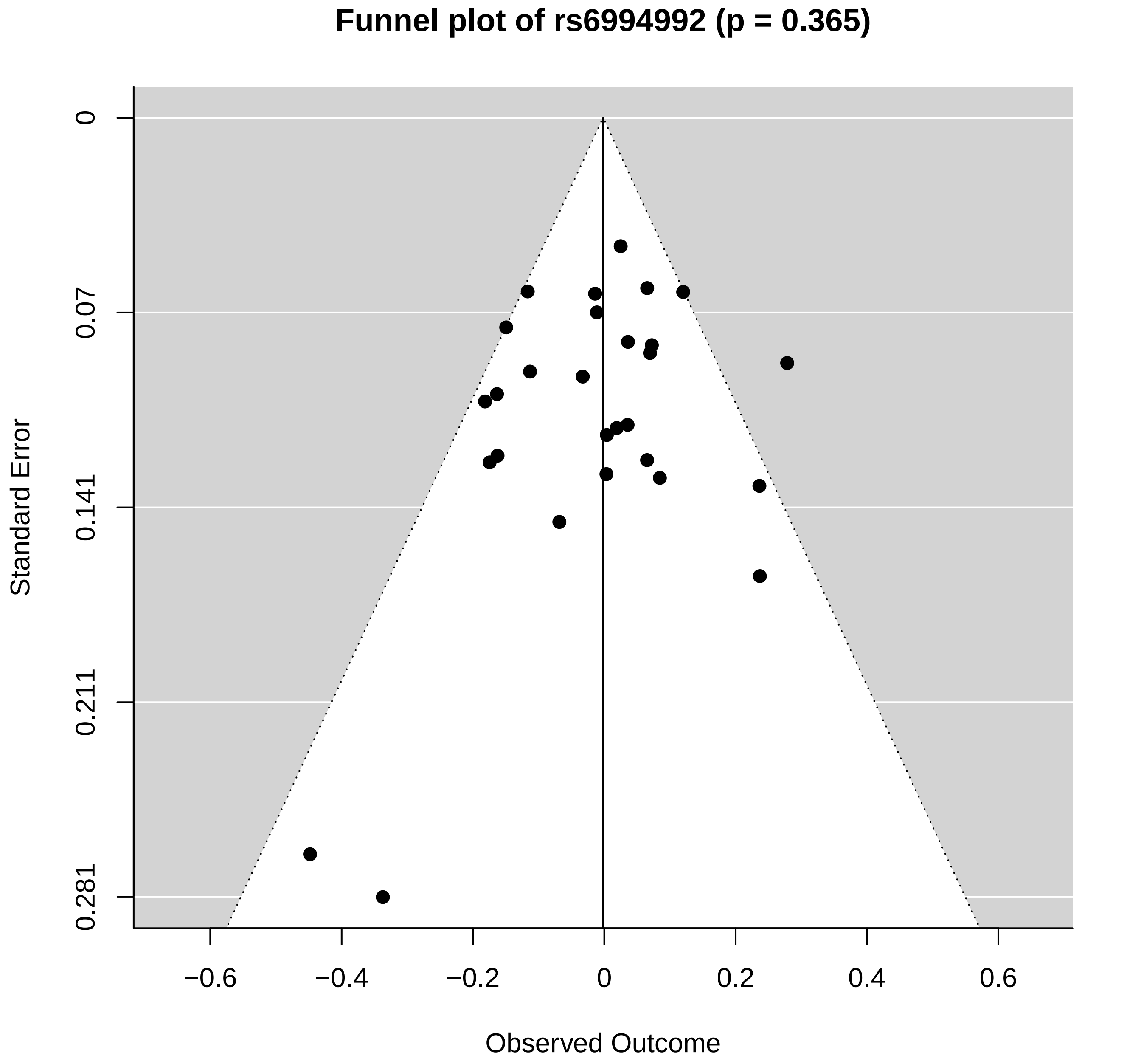
Funnel plot of rs6994992 (p = 0.365)
Standard Error
0
0.07
0.141
0.211
0.281
−0.6
−0.4
−0.2
0
0.2
0.4
0.6
Observed Outcome

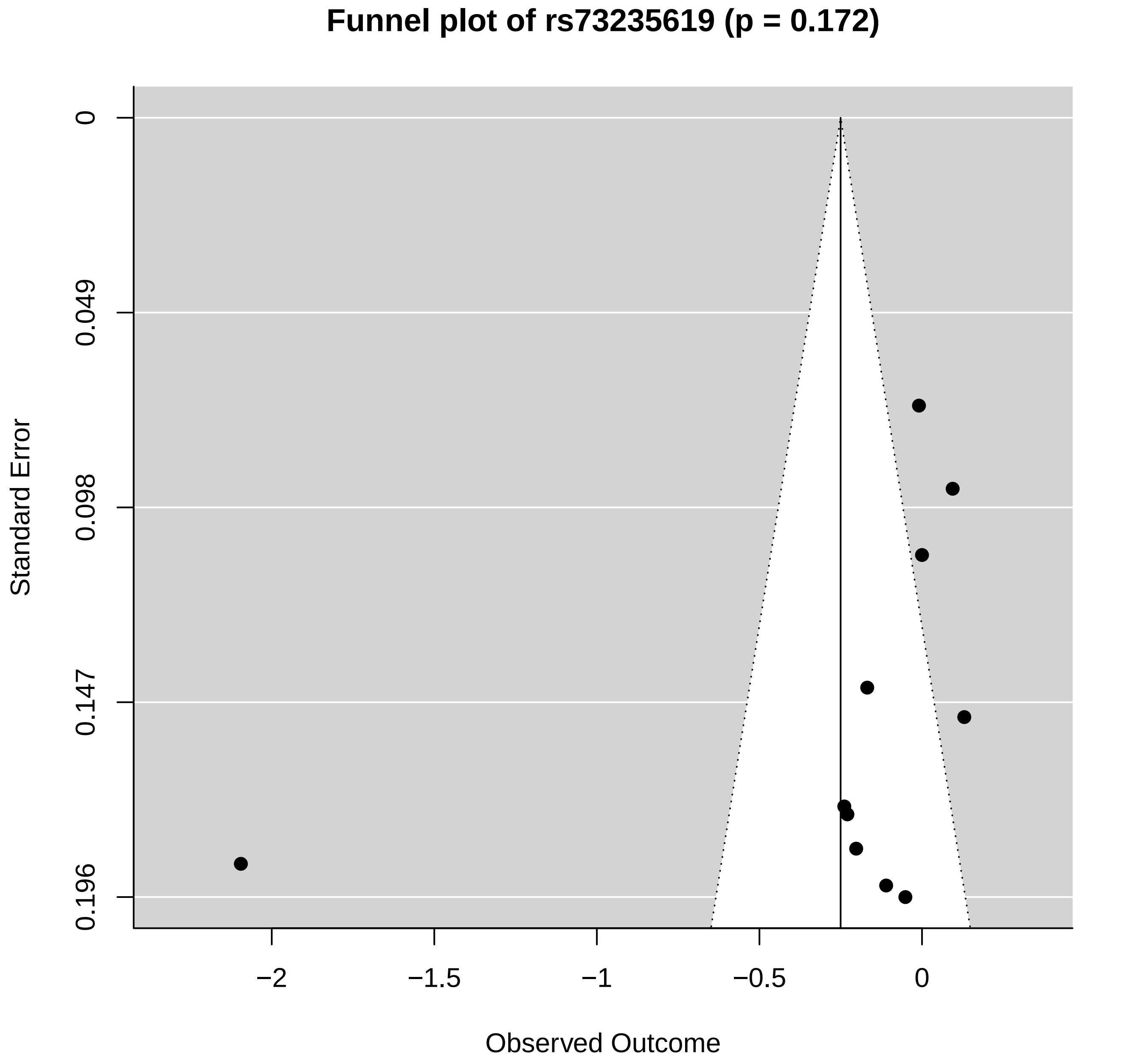

Funnel plot of rs73235619 (p = 0.172)
Standard Error
0
0.049
0.098
0.147
0.196
−2
−1.5
−1
−0.5
0
Observed Outcome

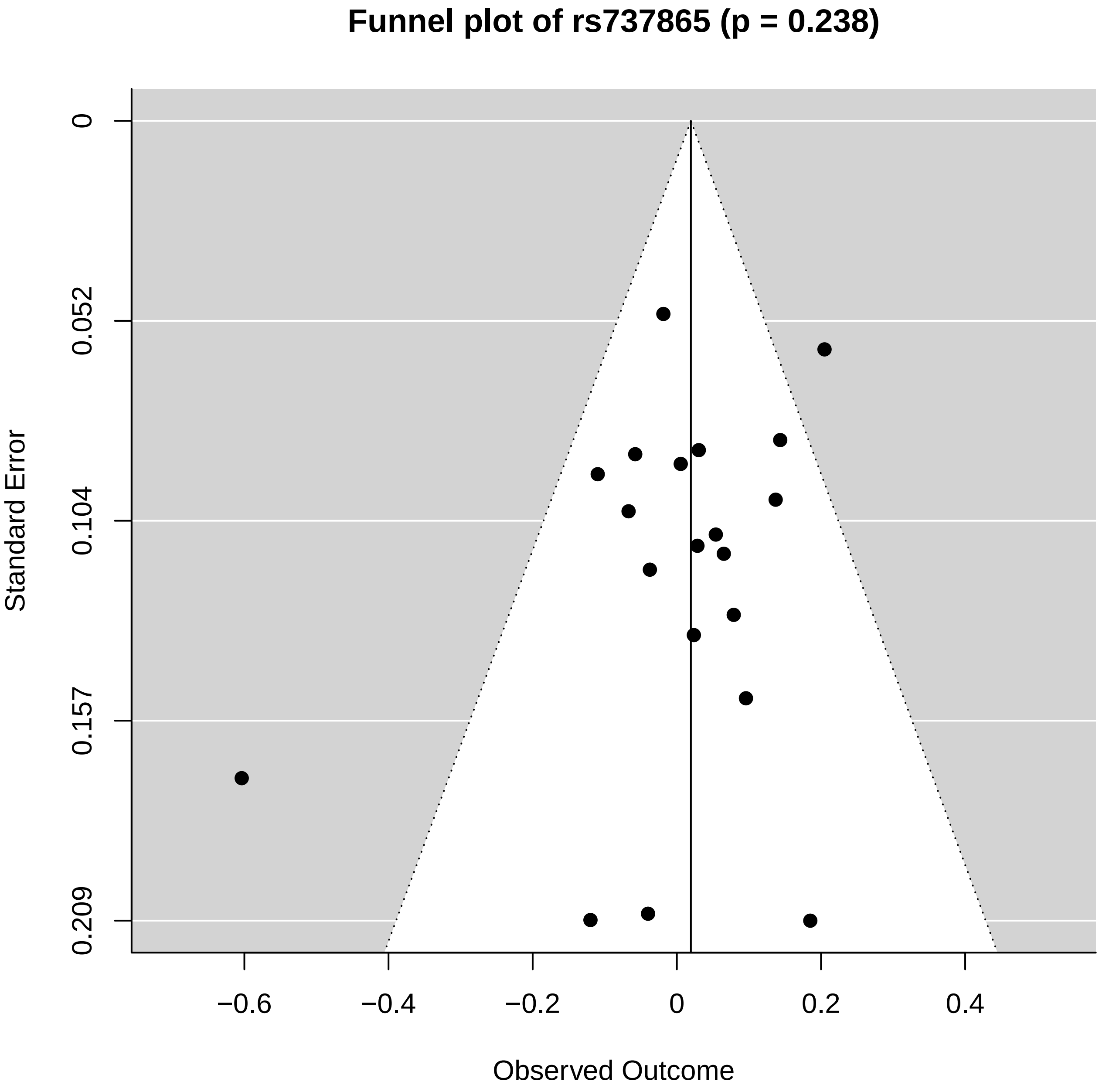
Funnel plot of rs737865 (p = 0.238)
Standard Error
0
0.052
0.104
0.157
0.209
−0.6
−0.4
−0.2
0
0.2
0.4
Observed Outcome

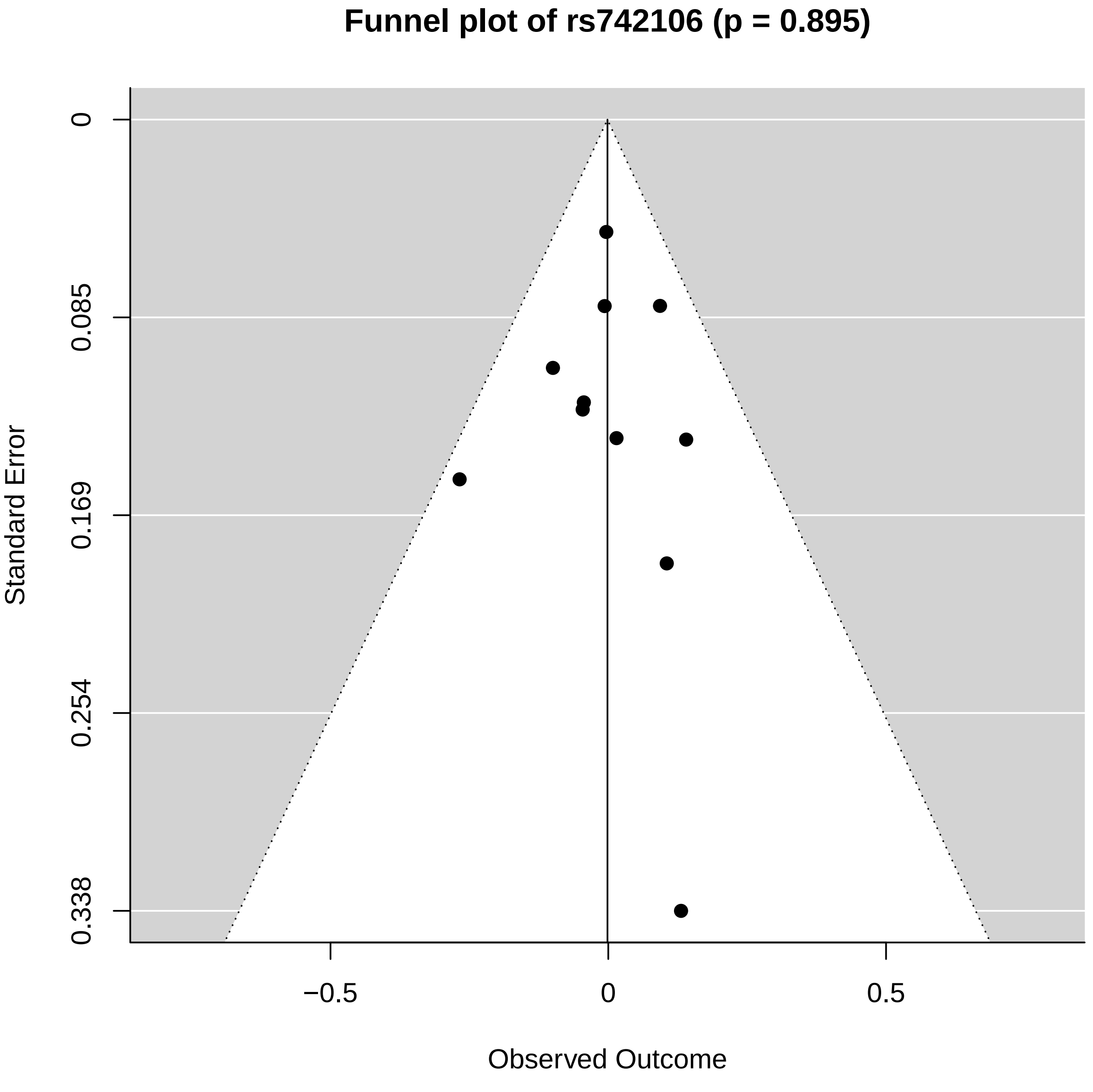

Funnel plot of rs742106 (p = 0.895)
0
0.085
0.169
0.254
0.338
Standard Error
−0.5
0
0.5
Observed Outcome

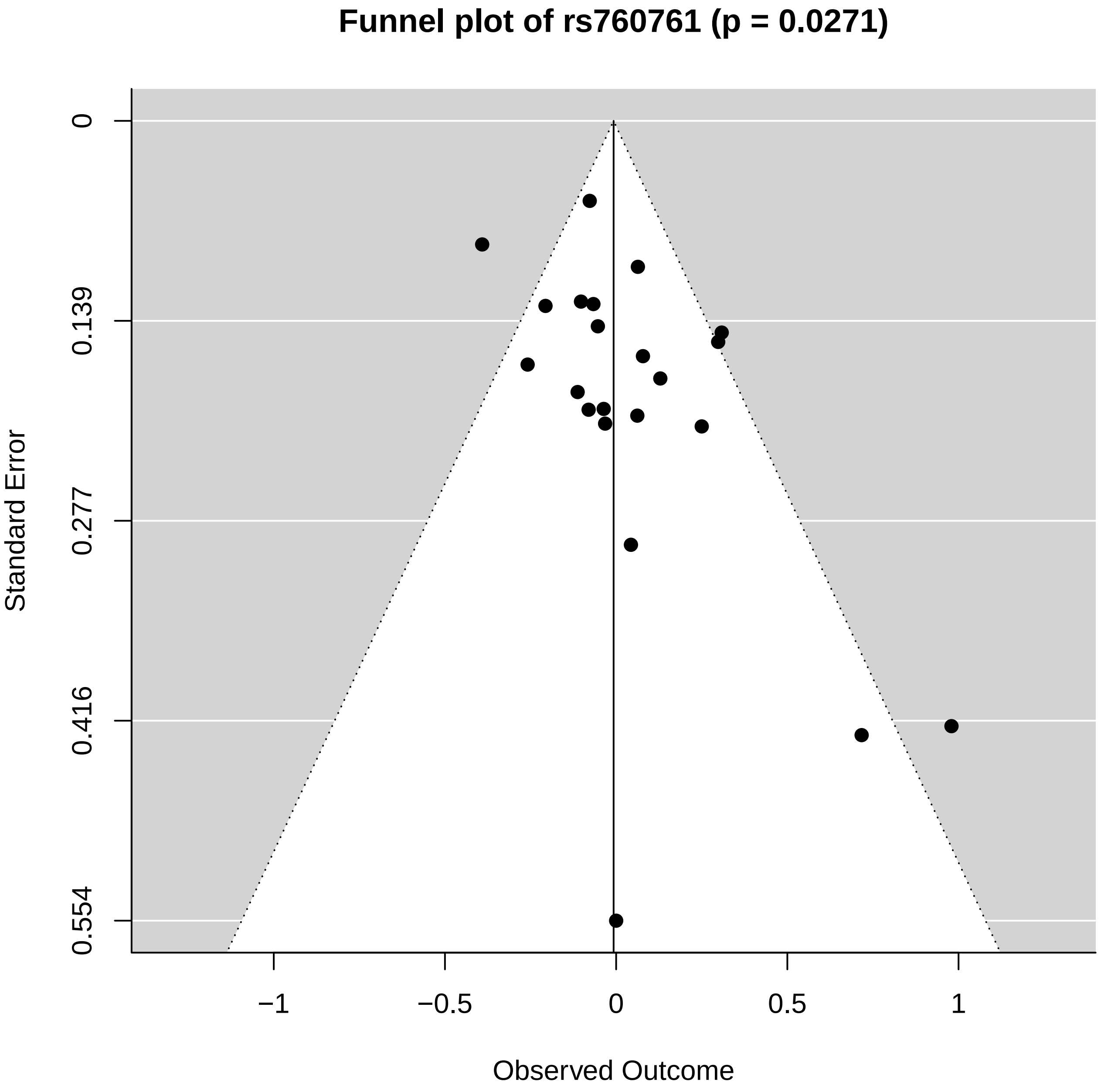
Funnel plot of rs760761 (p = 0.0271)
Standard Error
0
0.139
0.277
0.416
0.554
−1
−0.5
0
0.5
1
Observed Outcome

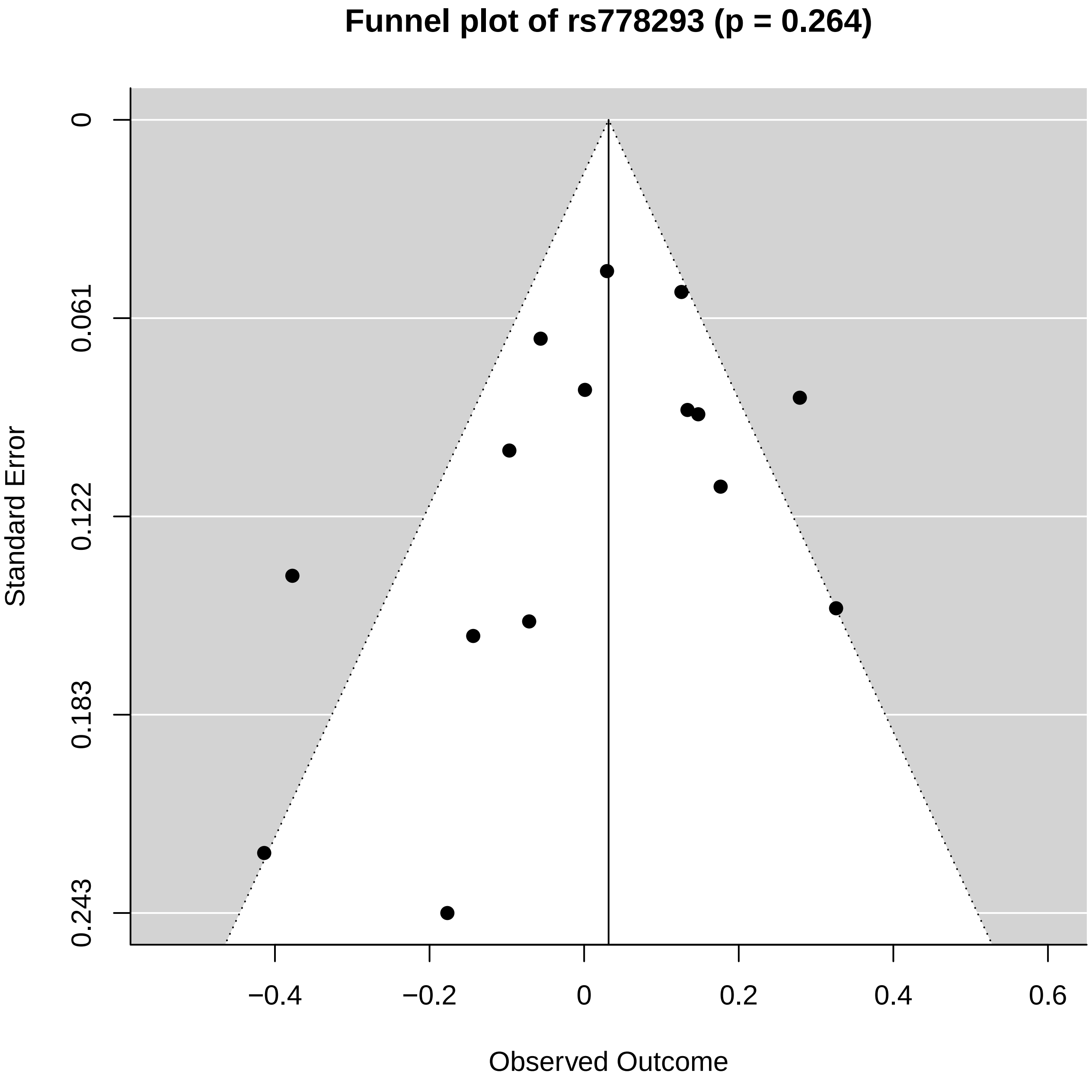
Funnel plot of rs778293 (p = 0.264)
Standard Error
0
0.061
0.122
0.183
0.243
−0.4
−0.2
0
0.2
0.4
0.6
Observed Outcome

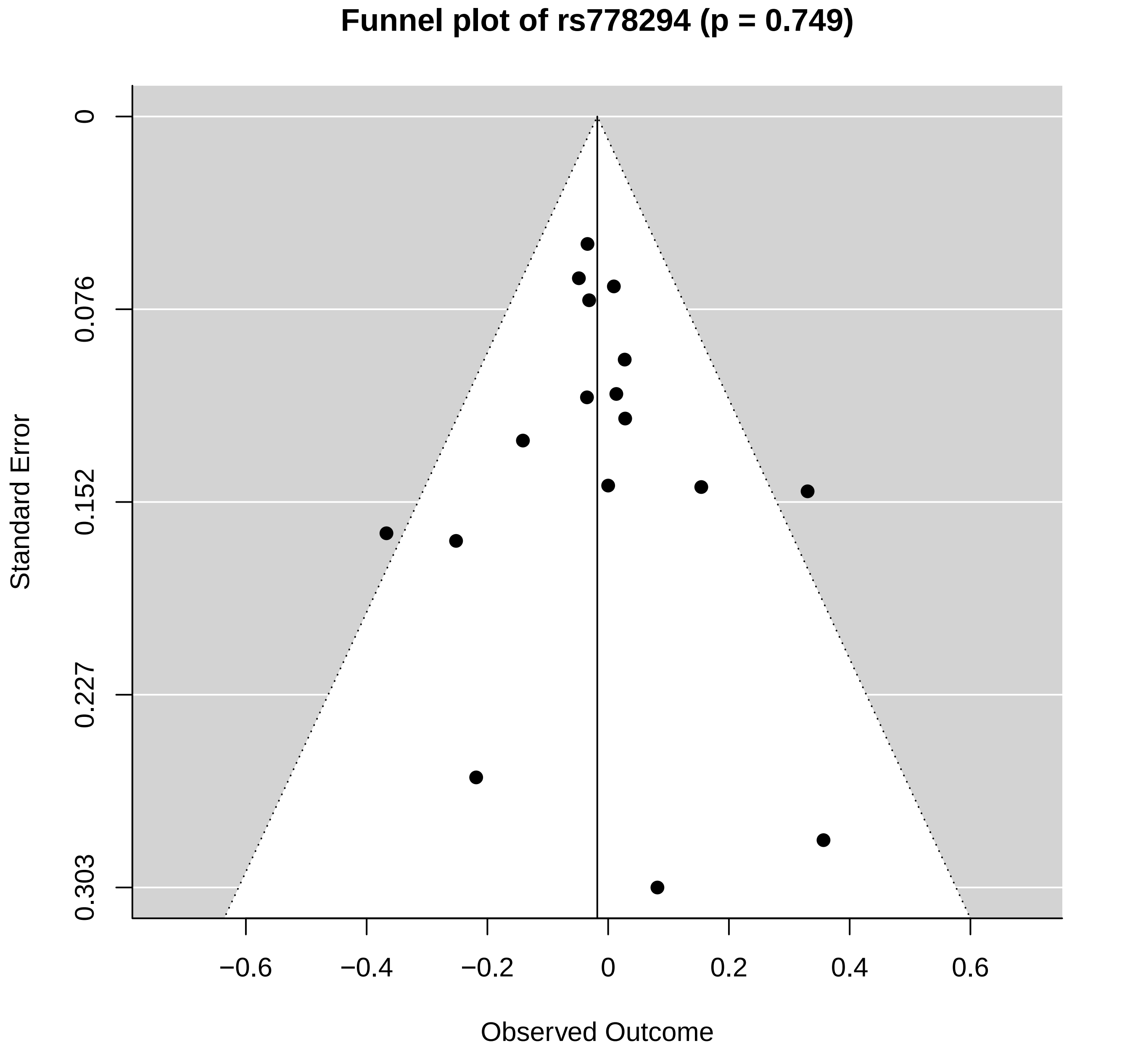

Funnel plot of rs778294 (p = 0.749)
Standard Error
0
0.076
0.152
0.227
0.303
−0.6
−0.4
−0.2
0
0.2
0.4
0.6
Observed Outcome

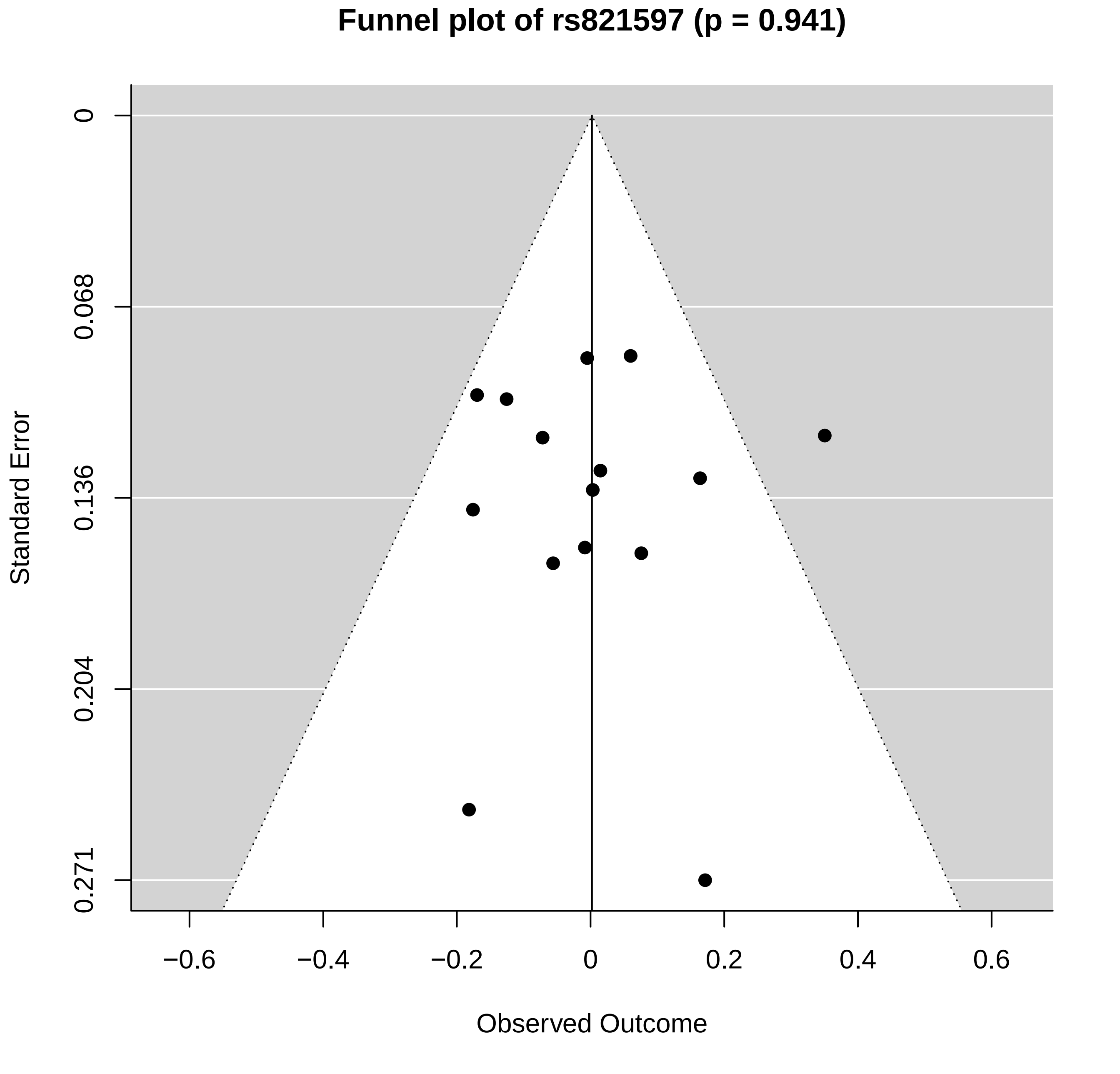
Funnel plot of rs821597 (p = 0.941)
Standard Error
0
0.068
0.136
0.204
0.271
−0.6
−0.4
−0.2
0
0.2
0.4
0.6
Observed Outcome

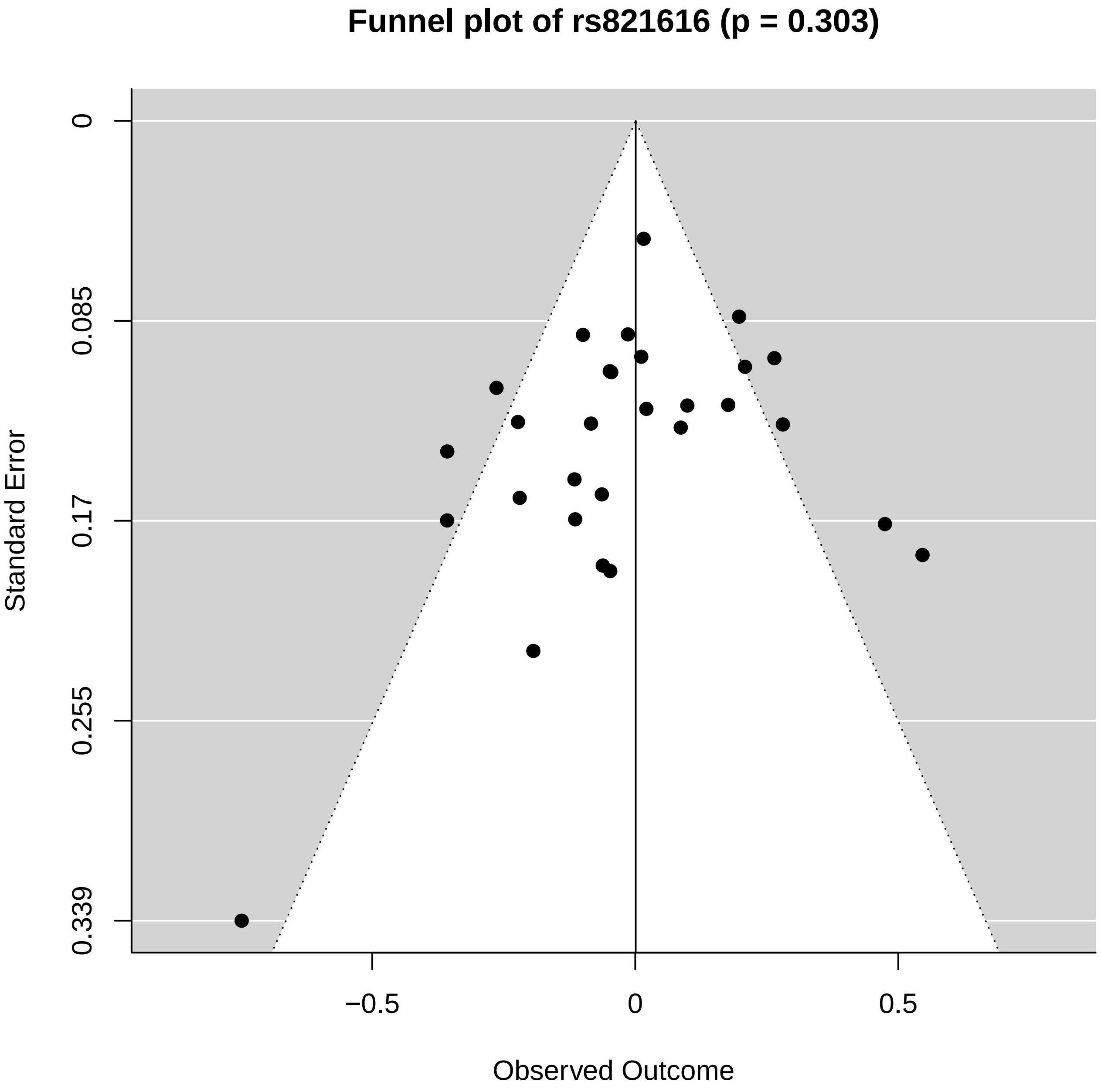
Funnel plot of rs821616 (p = 0.303)
0
0.085
0.17
0.255
0.339
Standard Error
−0.5
0
0.5
Observed Outcome

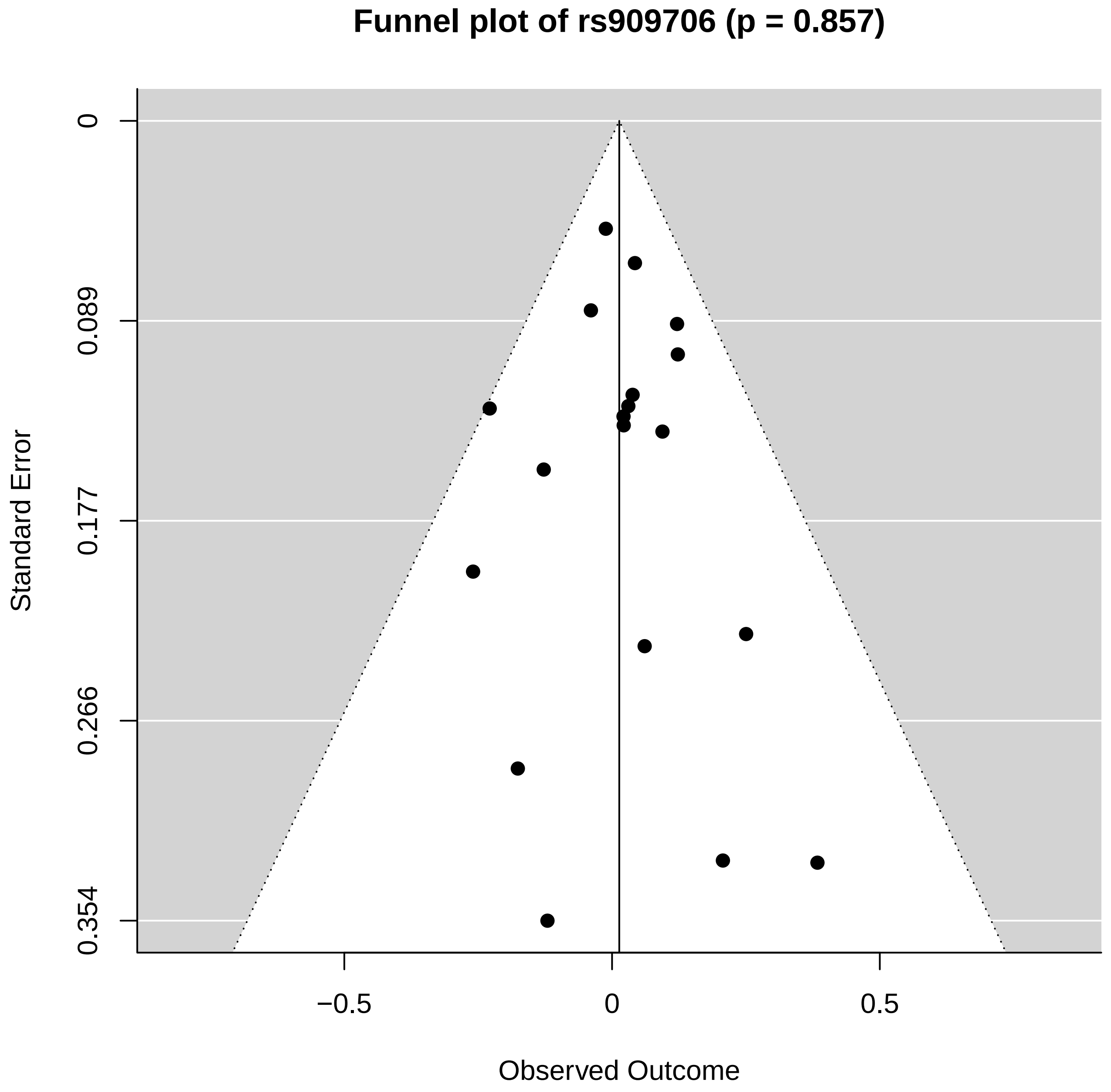
Funnel plot of rs909706 (p = 0.857)
Standard Error
0
0.089
0.177
0.266
0.354
−0.5
0
0.5
Observed Outcome

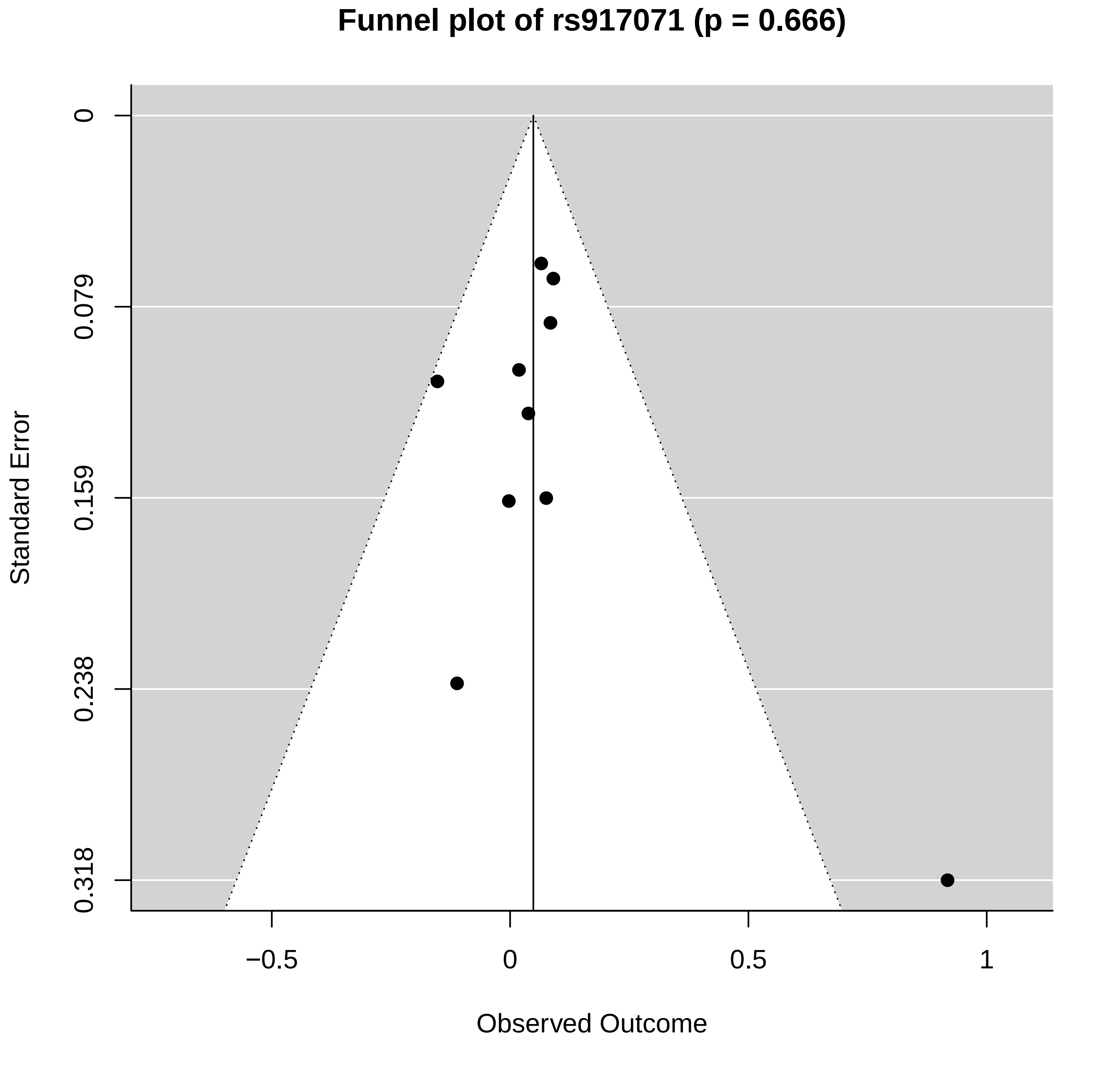
Funnel plot of rs917071 (p = 0.666)
Standard Error
0
0.079
0.159
0.238
0.318
−0.5
0
0.5
1
Observed Outcome

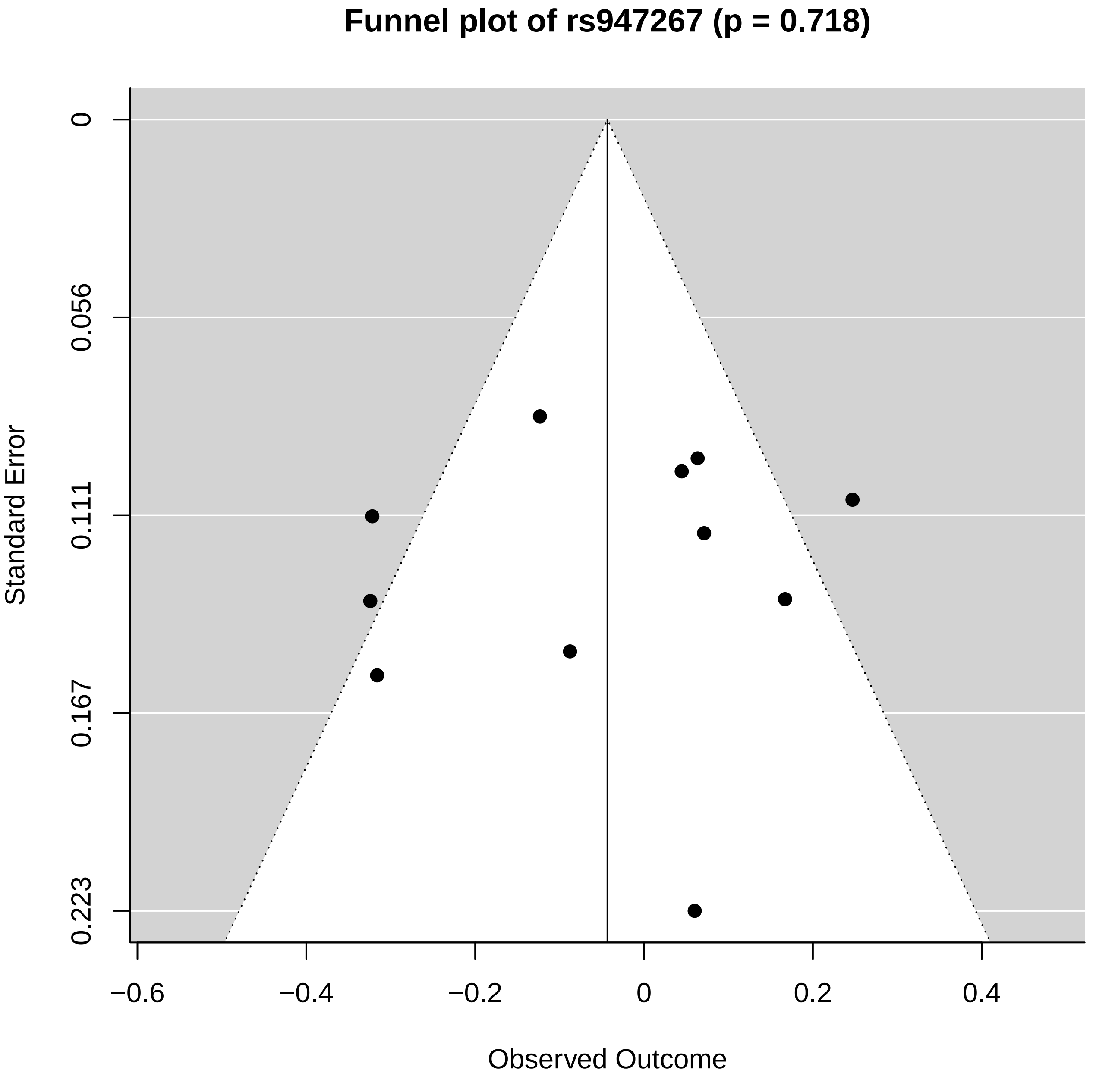

Funnel plot of rs947267 (p = 0.718)
Standard Error
0
0.056
0.111
0.167
0.223
−0.6
−0.4
−0.2
0
0.2
0.4
Observed Outcome

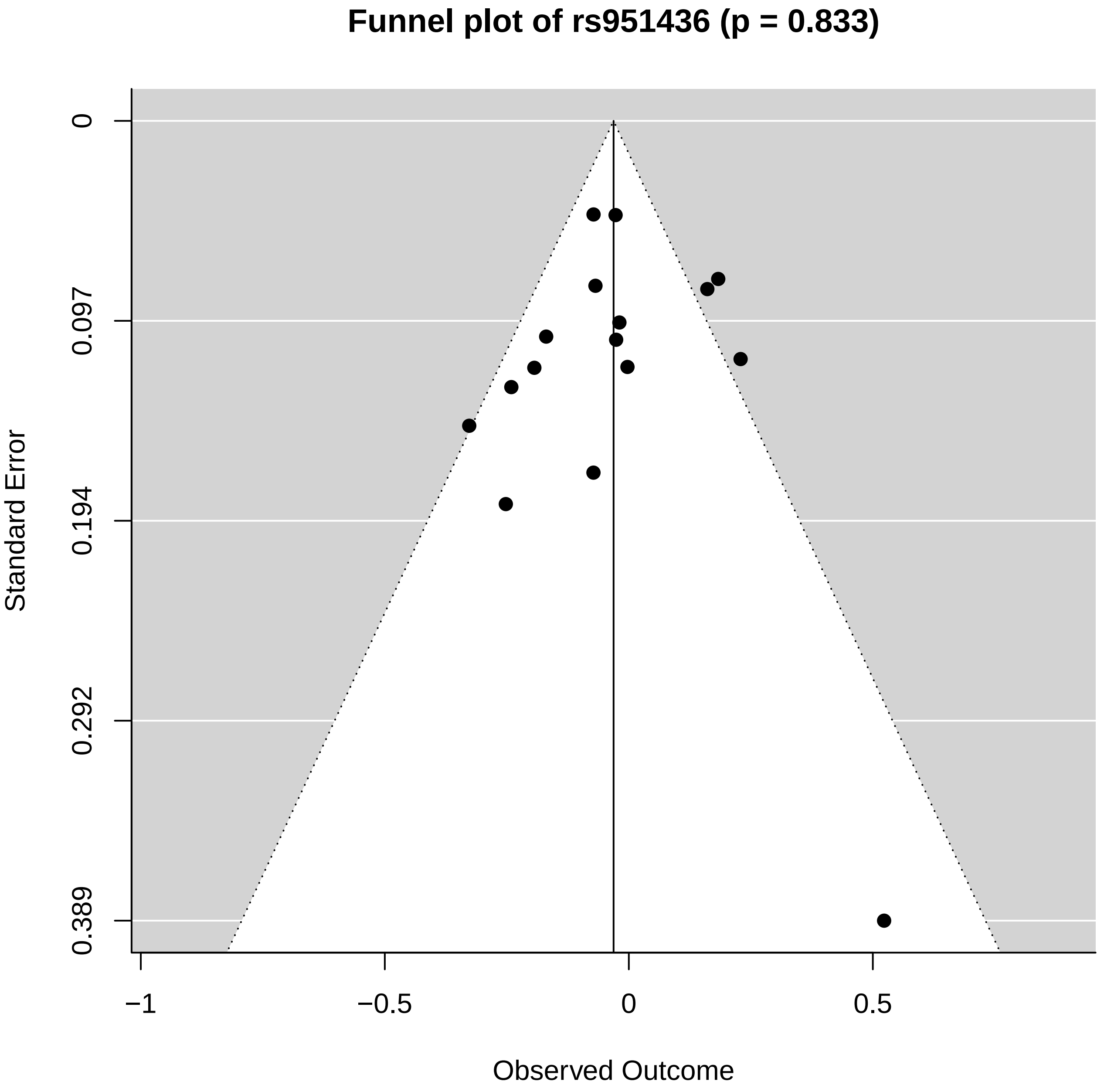

Funnel plot of rs951436 (p = 0.833)
Standard Error
0
0.097
0.194
0.292
0.389
−1
−0.5
0
0.5
Observed Outcome

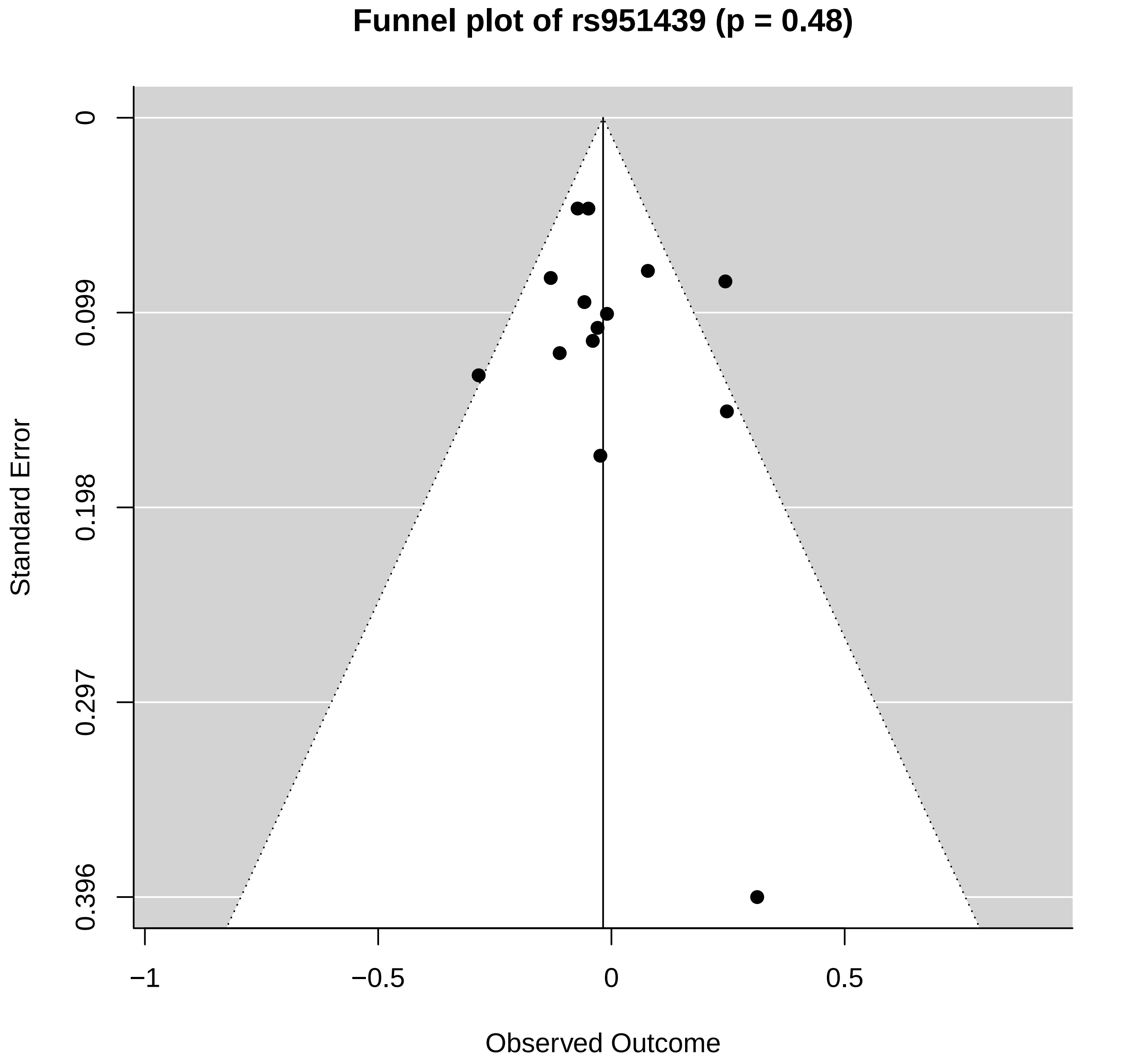
Funnel plot of rs951439 (p = 0.48)
Standard Error
0
0.099
0.198
0.297
0.396
−1
−0.5
0
0.5
Observed Outcome

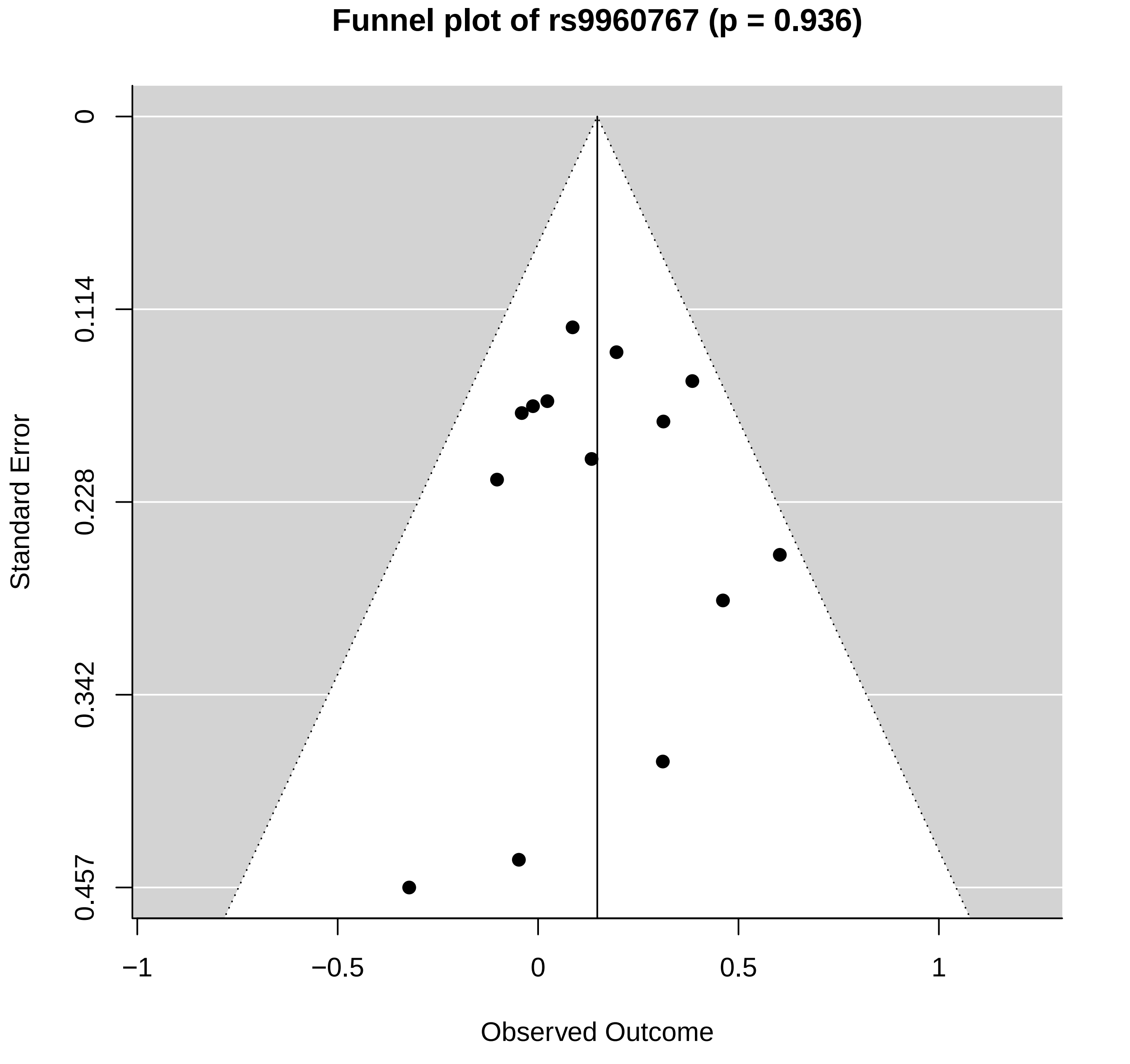
Funnel plot of rs9960767 (p = 0.936)
Standard Error
0
0.114
0.228
0.342
0.457
−1
−0.5
0
0.5
1
Observed Outcome

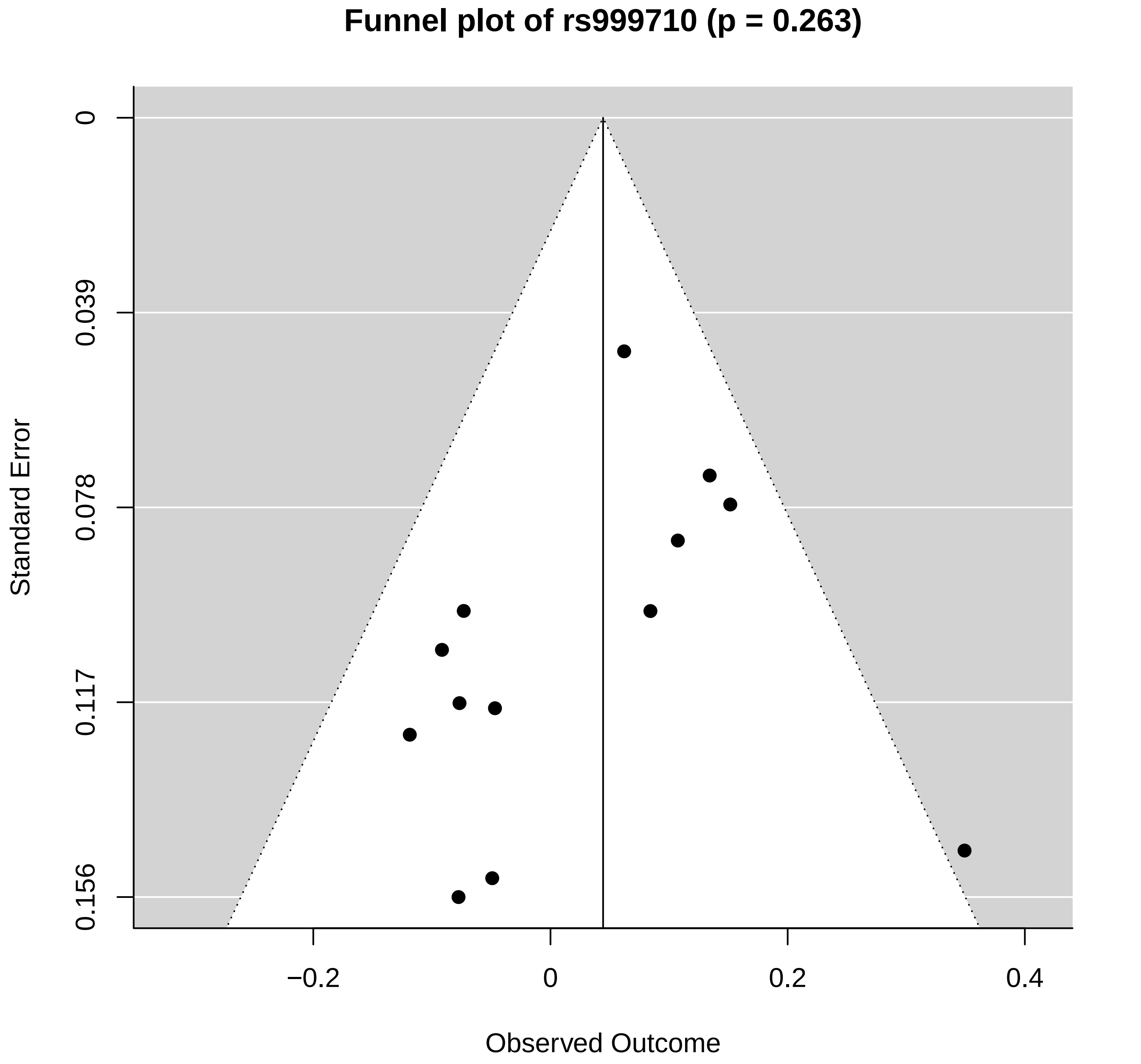
Funnel plot of rs999710 (p = 0.263)
Standard Error
0
0.039
0.078
0.117
0.156
−0.2
0
0.2
0.4
Observed Outcome
