# Supplementary material for: The schizophrenia genetics knowledgebase: a comprehensive update of findings from candidate gene studies
Source: Transl Psychiatry. 2019 Aug 27;9:205. doi: 10.1038/s41398-019-0532-4 (PMC6711957; doi:10.1038/s41398-019-0532-4)
Supplement: Supplementary file 6 — Supplementary Data S3 [file 41398_2019_532_MOESM6_ESM.pdf]

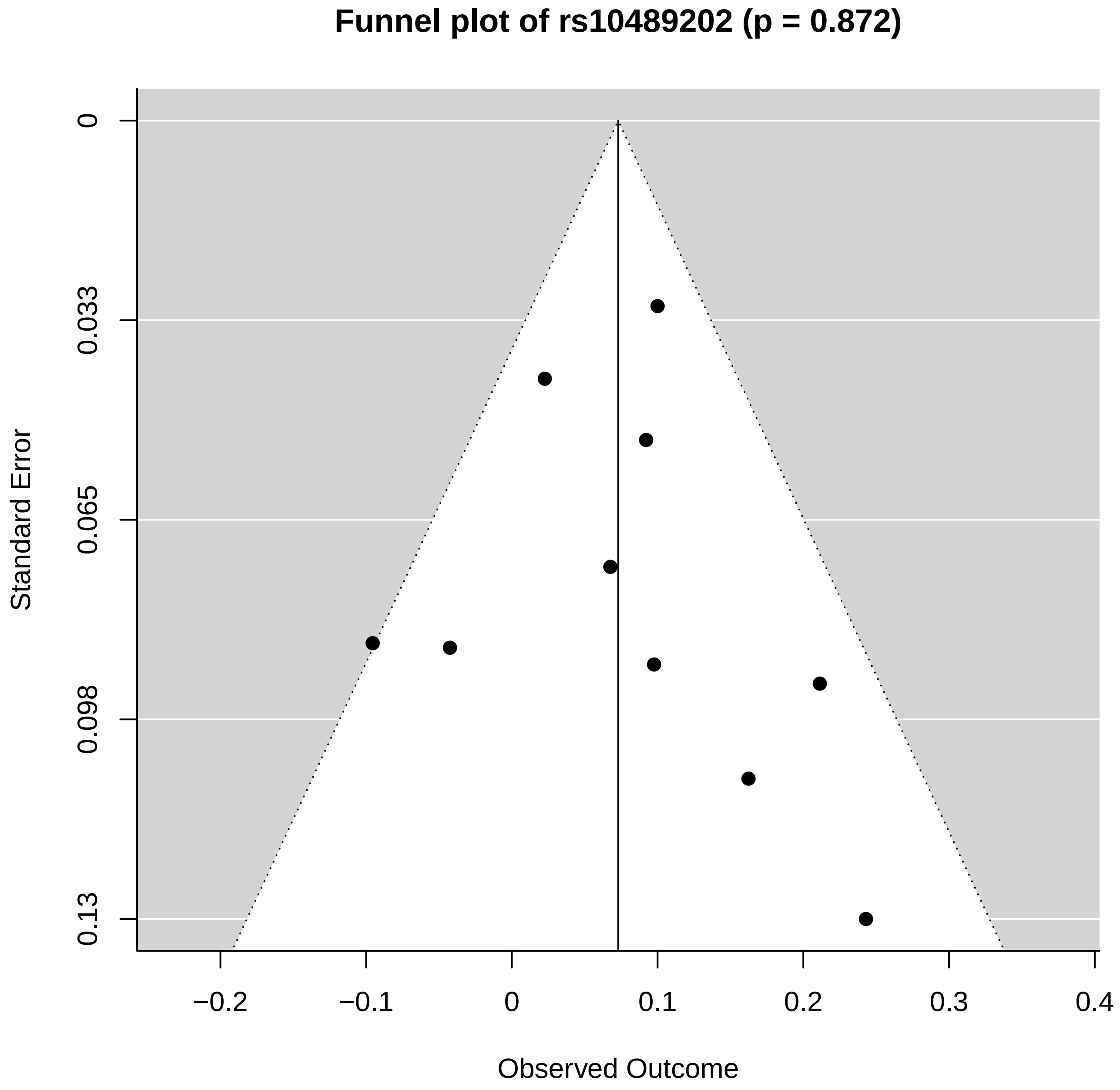

Funnel plot of rs10489202 (p = 0.872)
Standard Error
0
0.033
0.065
0.098
0.13
−0.2
−0.1
0
0.1
0.2
0.3
0.4
Observed Outcome

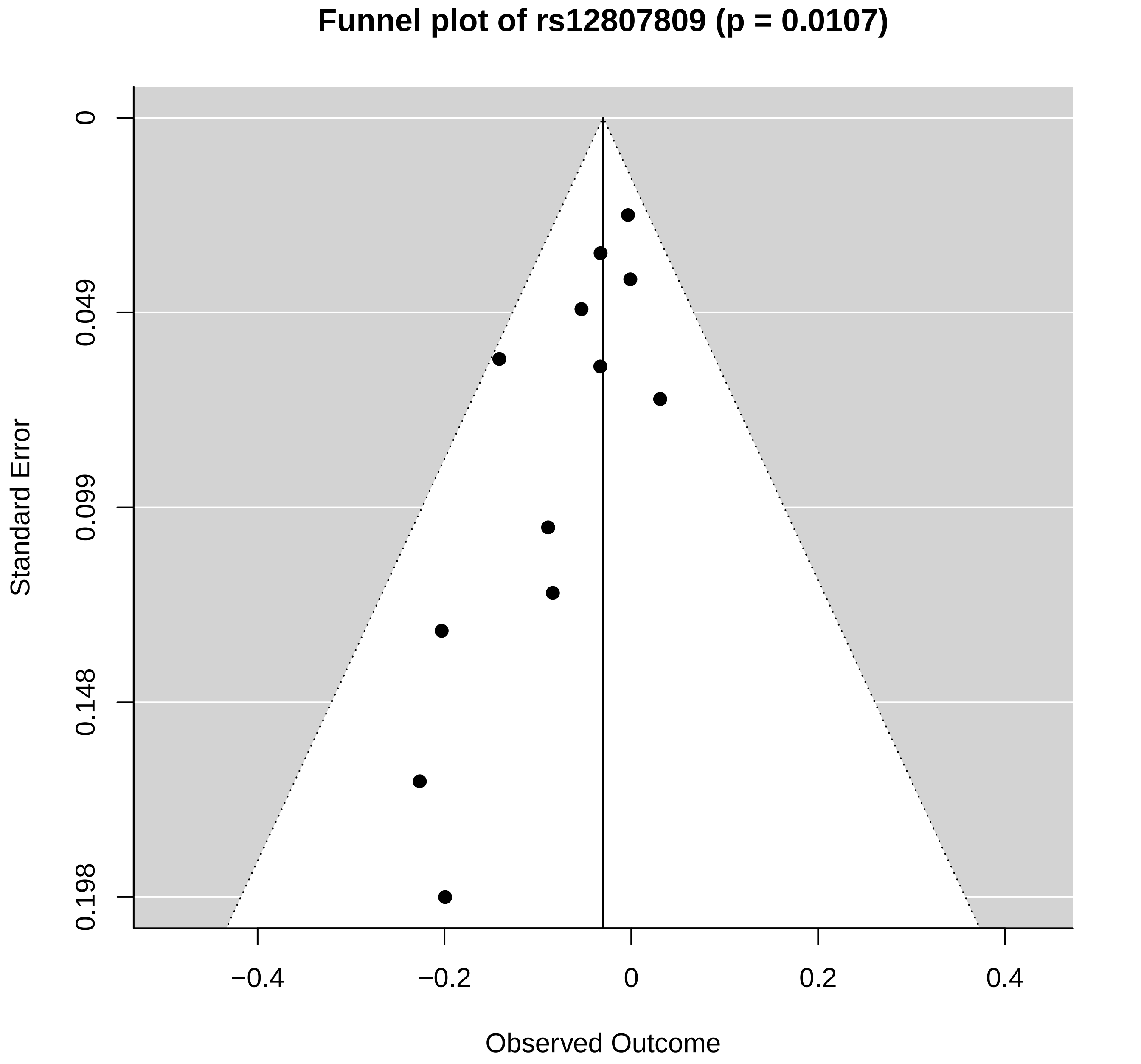
Funnel plot of rs12807809 (p = 0.0107)
Standard Error
0
0.049
0.099
0.148
0.198
−0.4
−0.2
0
0.2
0.4
Observed Outcome

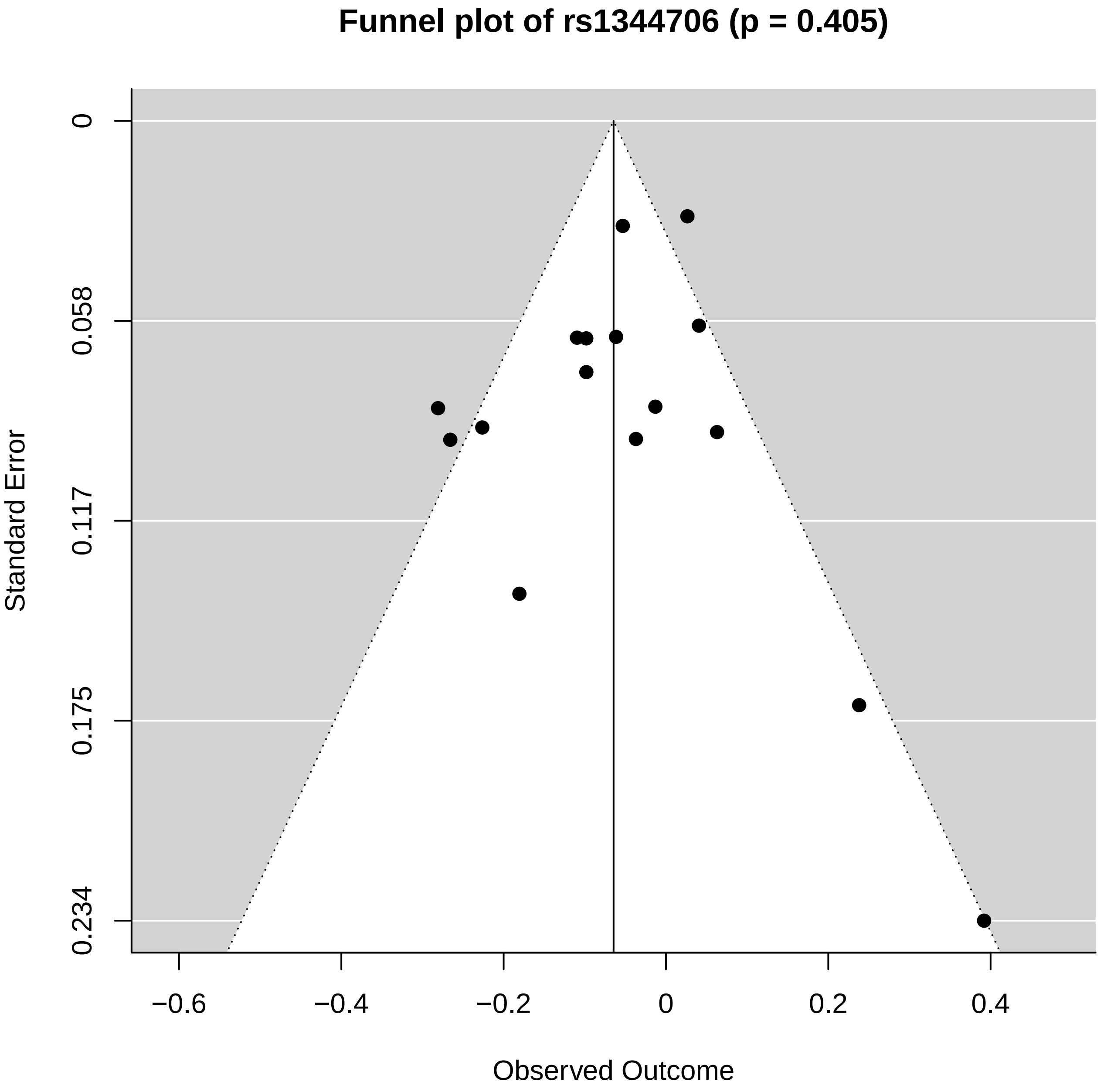

Funnel plot of rs1344706 (p = 0.405)
Standard Error
0
0.058
0.117
0.175
0.234
−0.6
−0.4
−0.2
0
0.2
0.4
Observed Outcome

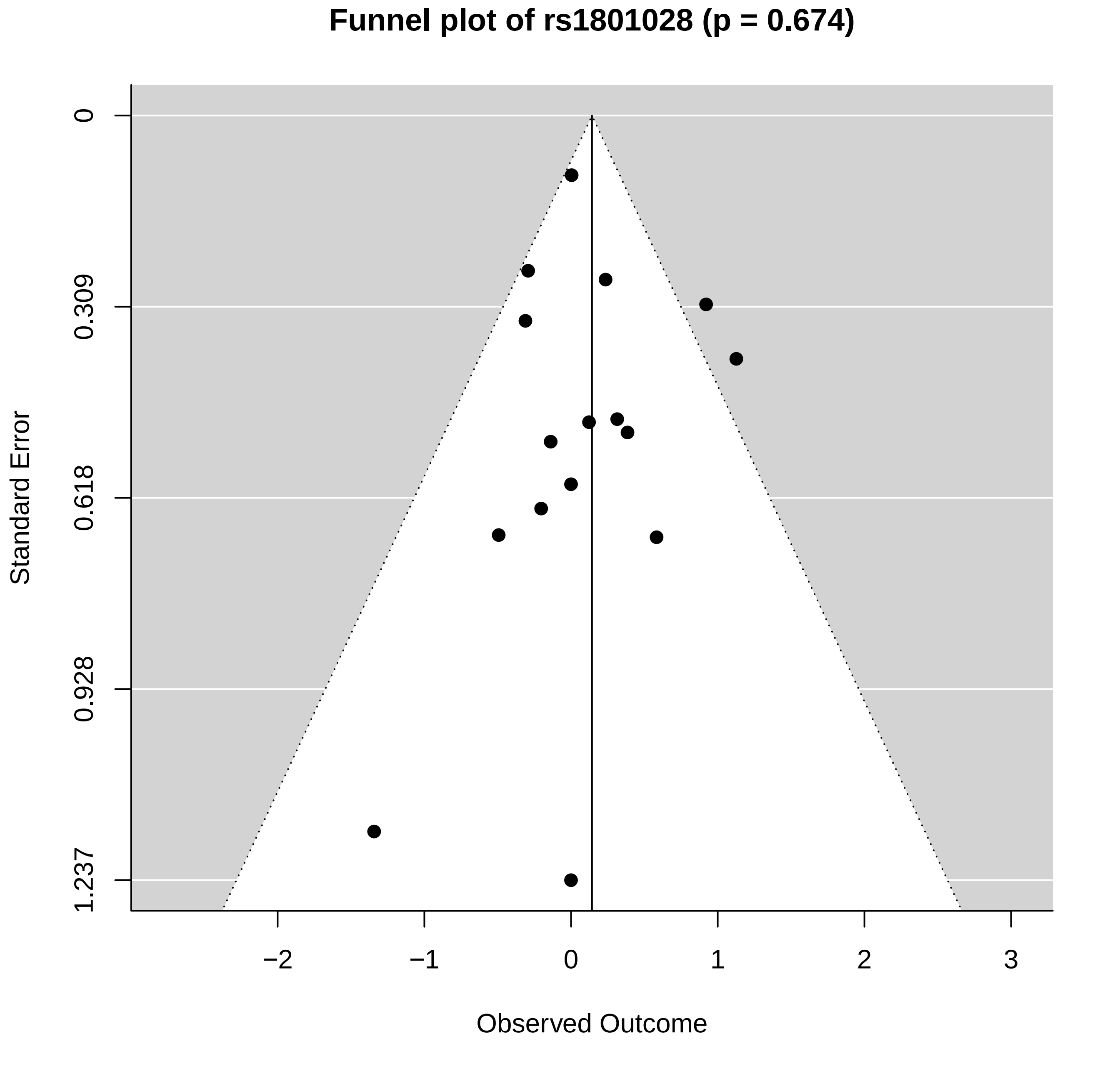
Funnel plot of rs1801028 (p = 0.674)
Standard Error
0
0.309
0.618
0.928
1.237
−2
−1
0
1
2
3
Observed Outcome

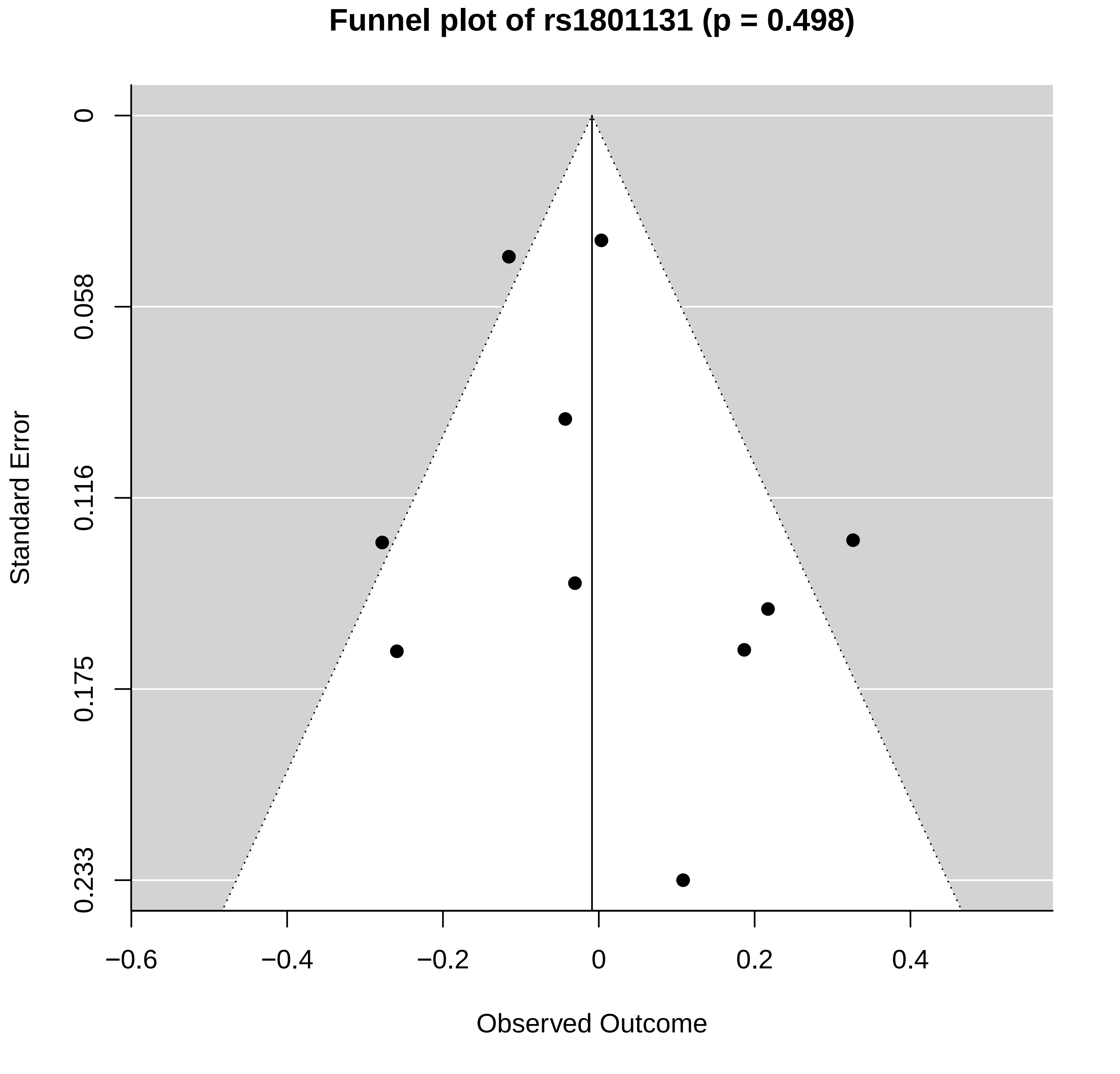

Funnel plot of rs1801131 (p = 0.498)
Standard Error
0
0.058
0.116
0.175
0.233
−0.6
−0.4
−0.2
0
0.2
0.4
Observed Outcome

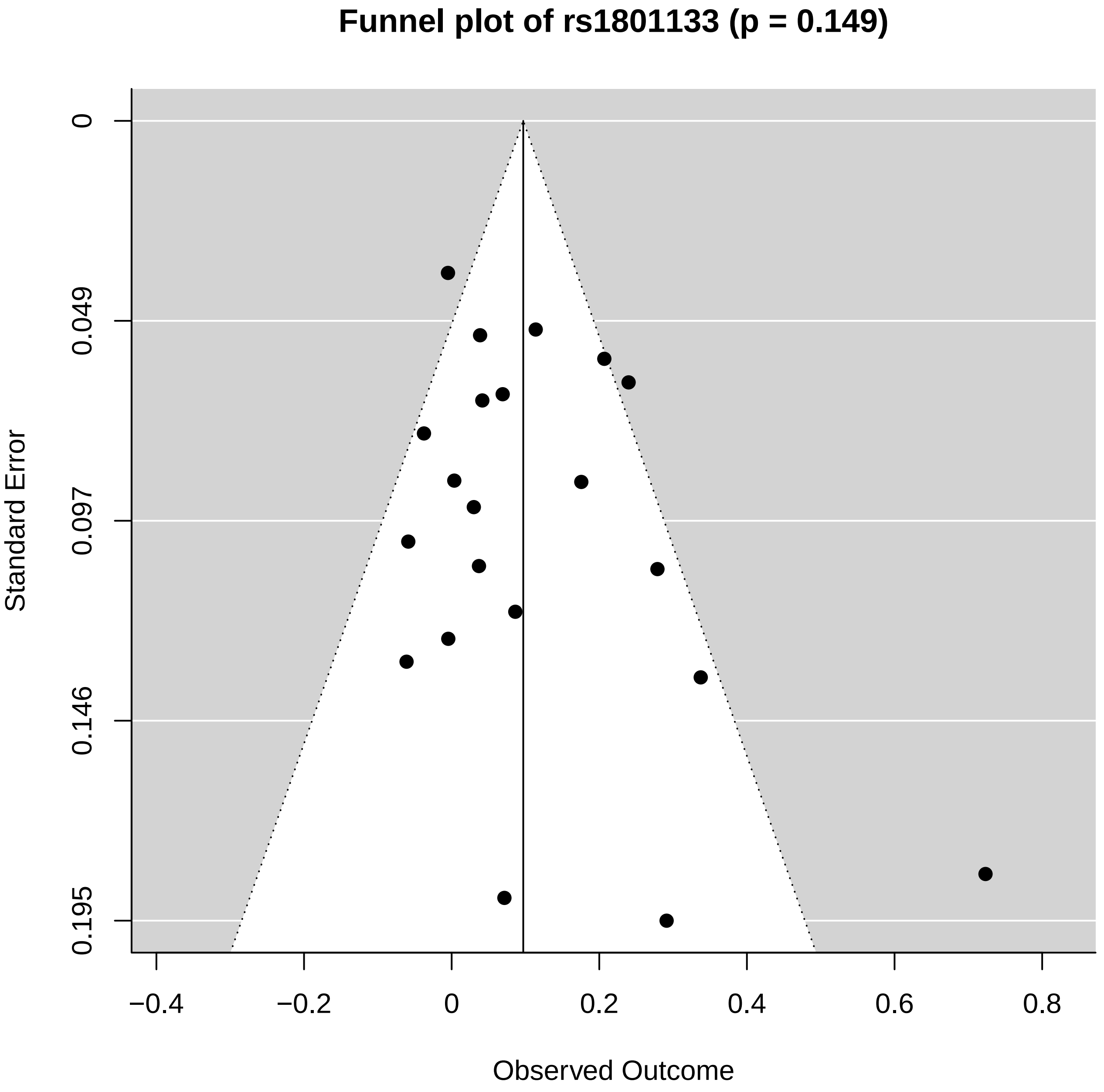

Funnel plot of rs1801133 (p = 0.149)
0
0.049
0.097
0.146
0.195
Standard Error
−0.4
−0.2
0
0.2
0.4
0.6
0.8
Observed Outcome

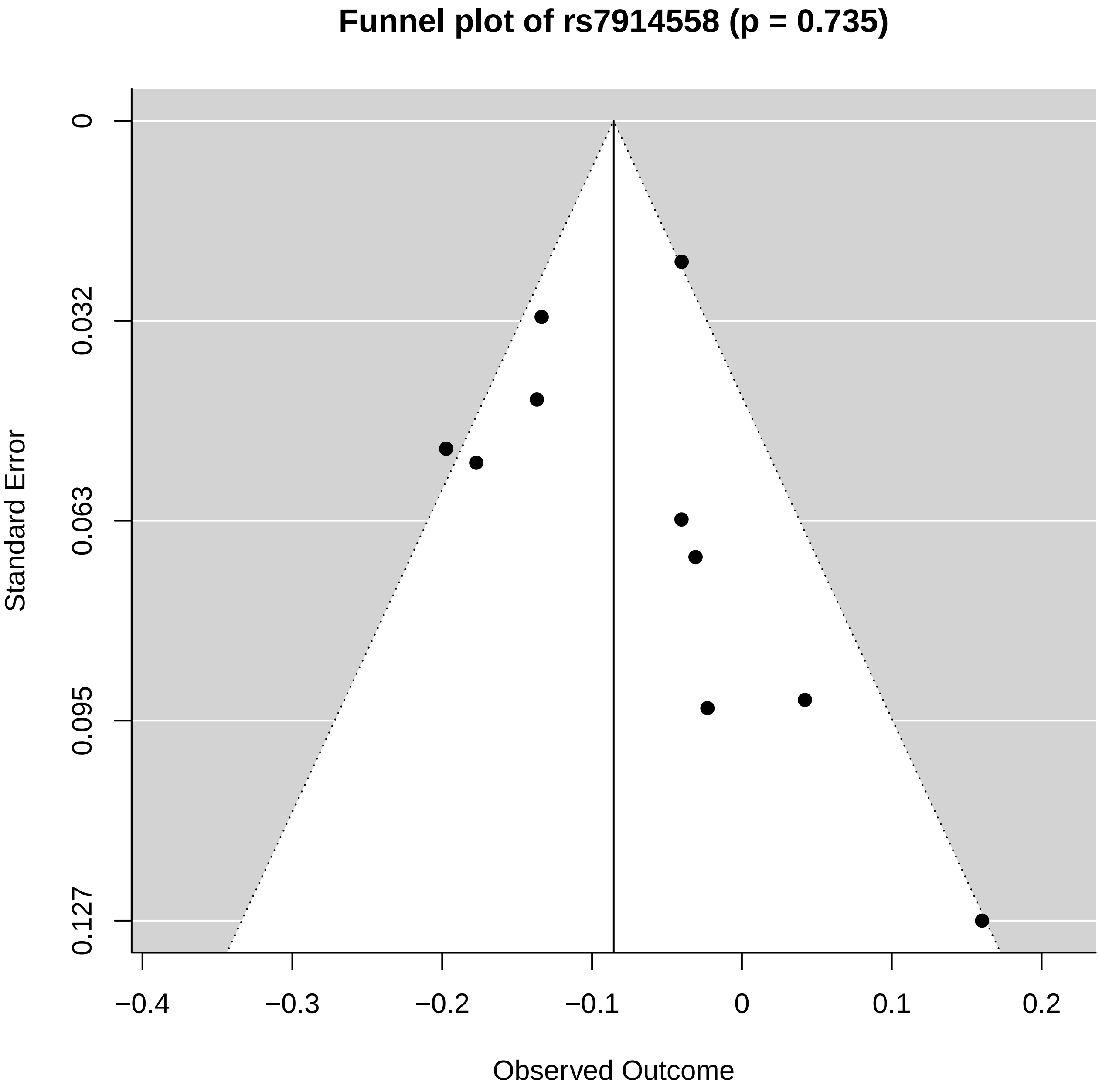
Funnel plot of rs7914558 (p = 0.735)
Standard Error
0
0.032
0.063
0.095
0.127
−0.4
−0.3
−0.2
−0.1
0
0.1
0.2
Observed Outcome
