# Supplementary material for: The schizophrenia genetics knowledgebase: a comprehensive update of findings from candidate gene studies
Source: Transl Psychiatry. 2019 Aug 27;9:205. doi: 10.1038/s41398-019-0532-4 (PMC6711957; doi:10.1038/s41398-019-0532-4)
Supplement: Supplementary file 7 — Supplementary Data S4 [file 41398_2019_532_MOESM7_ESM.pdf]

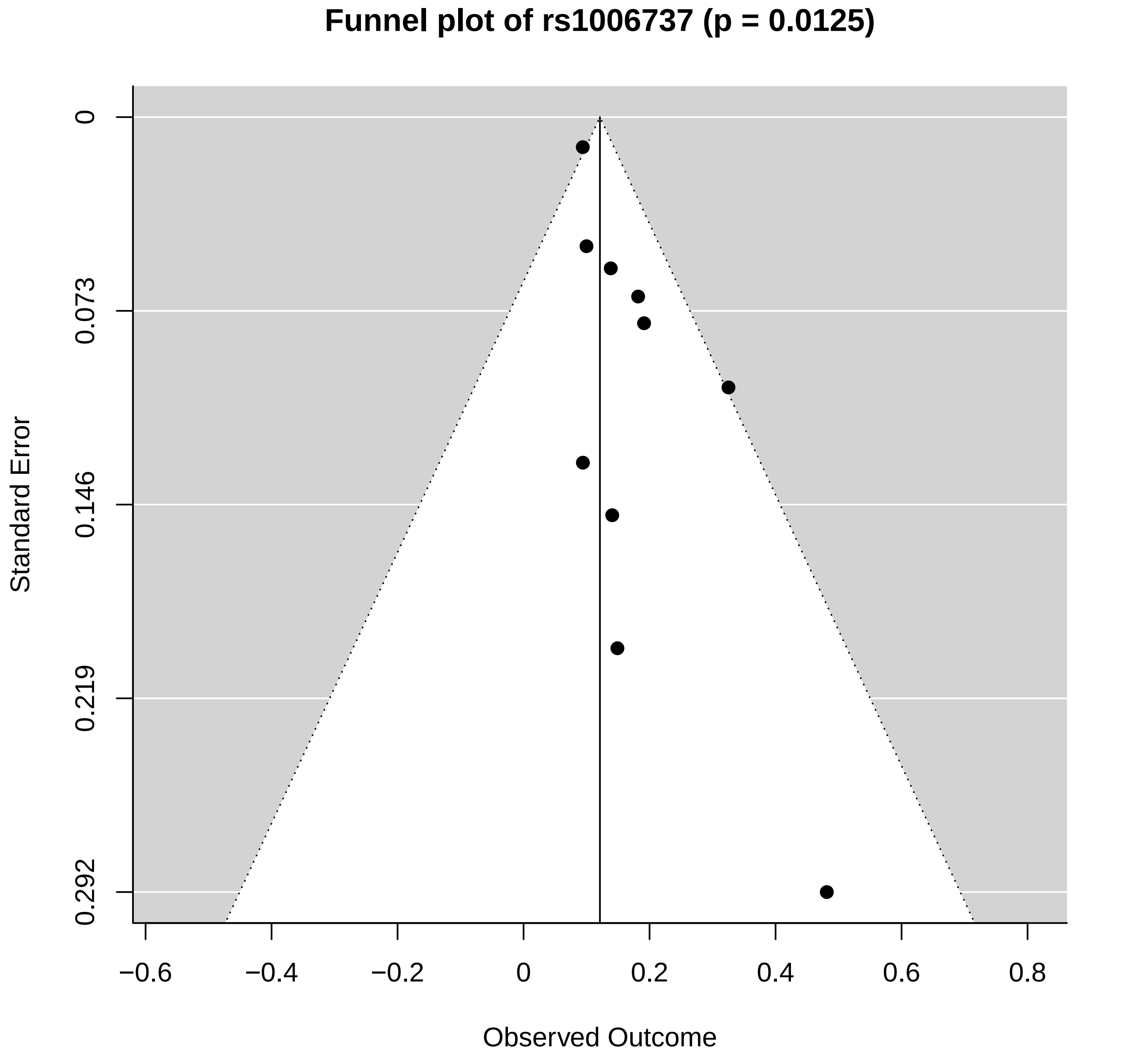
Funnel plot of rs1006737 (p = 0.0125)
Standard Error
0
0.073
0.146
0.219
0.292
−0.6
−0.4
−0.2
0
0.2
0.4
0.6
0.8
Observed Outcome

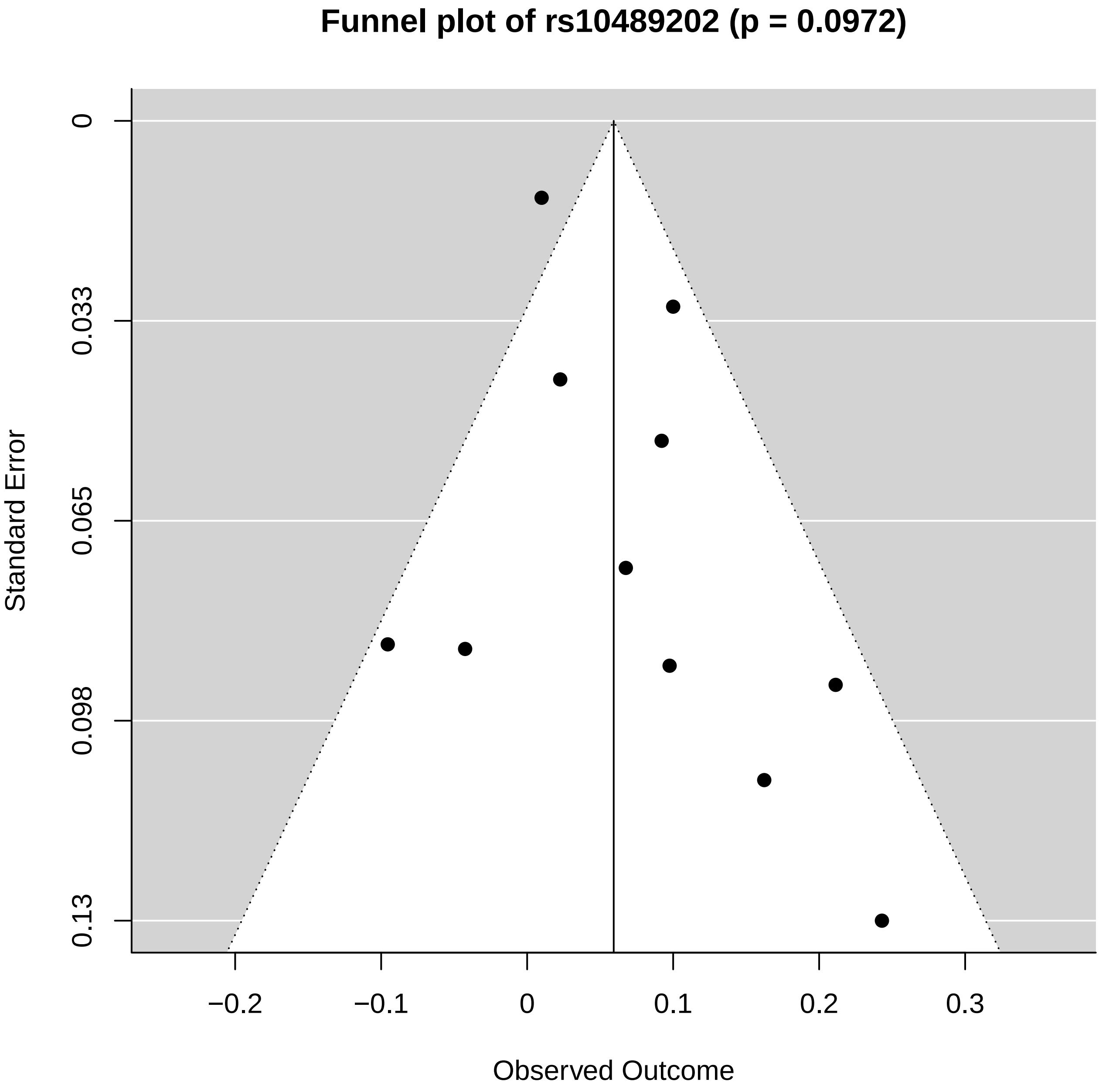

Funnel plot of rs10489202 (p = 0.0972)
Standard Error
0
0.033
0.065
0.098
0.13
−0.2
−0.1
0
0.1
0.2
0.3
Observed Outcome

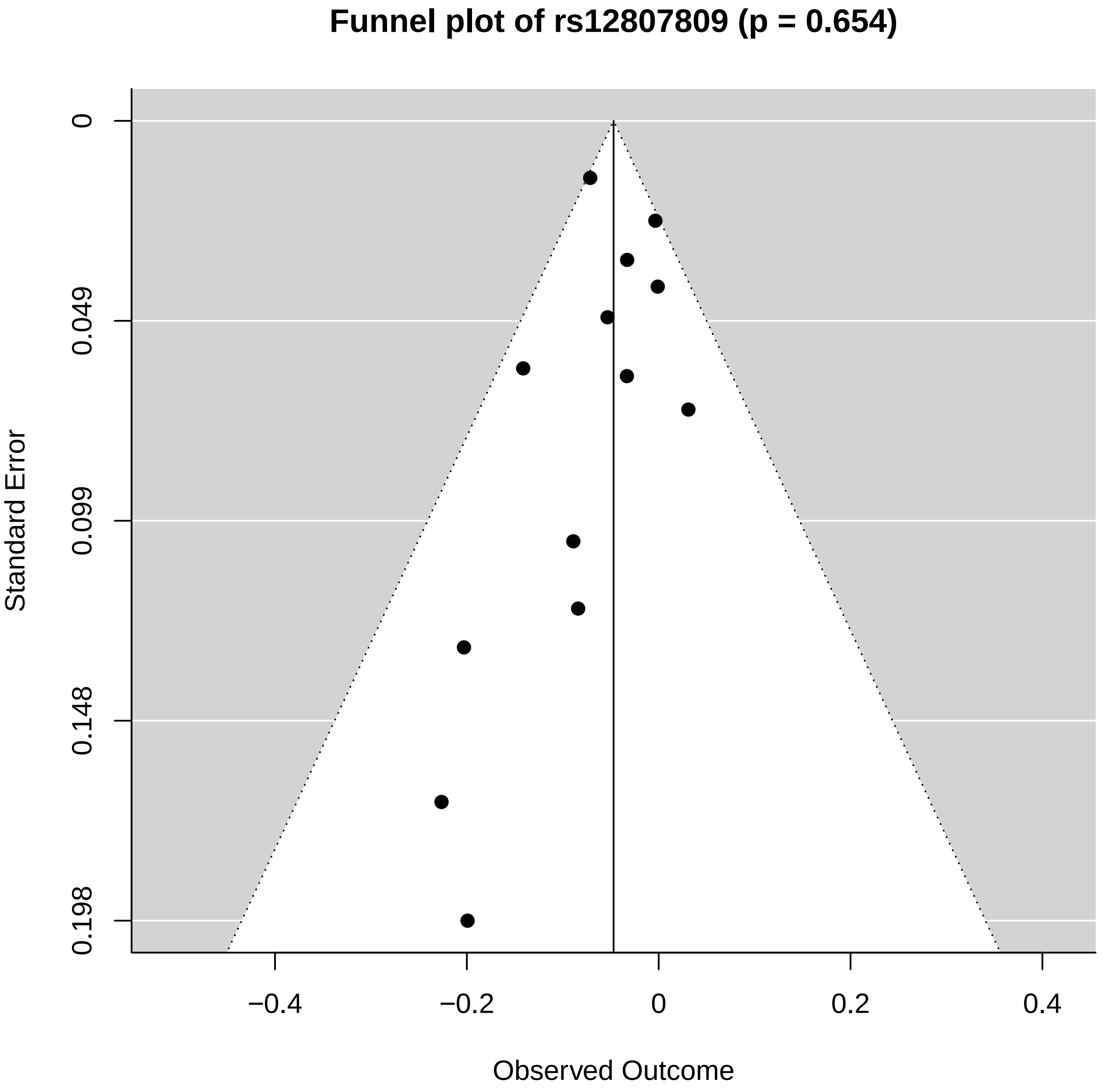

Funnel plot of rs12807809 (p = 0.654)
0
0.049
0.099
0.148
0.198
Standard Error
−0.4
−0.2
0
0.2
0.4
Observed Outcome

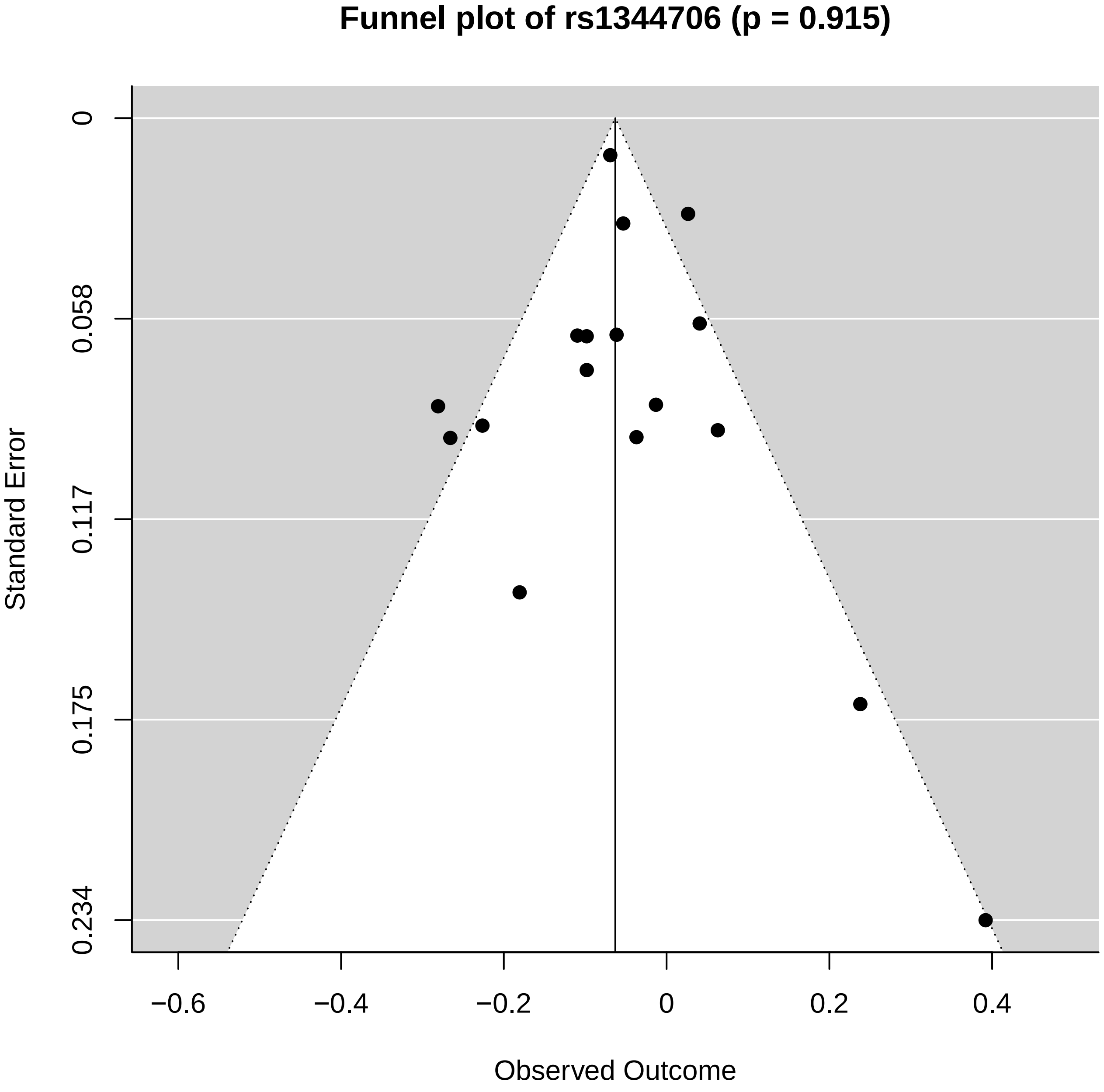

Funnel plot of rs1344706 (p = 0.915)
0
0.058
0.117
0.175
0.234
Standard Error
−0.6
−0.4
−0.2
0
0.2
0.4
Observed Outcome

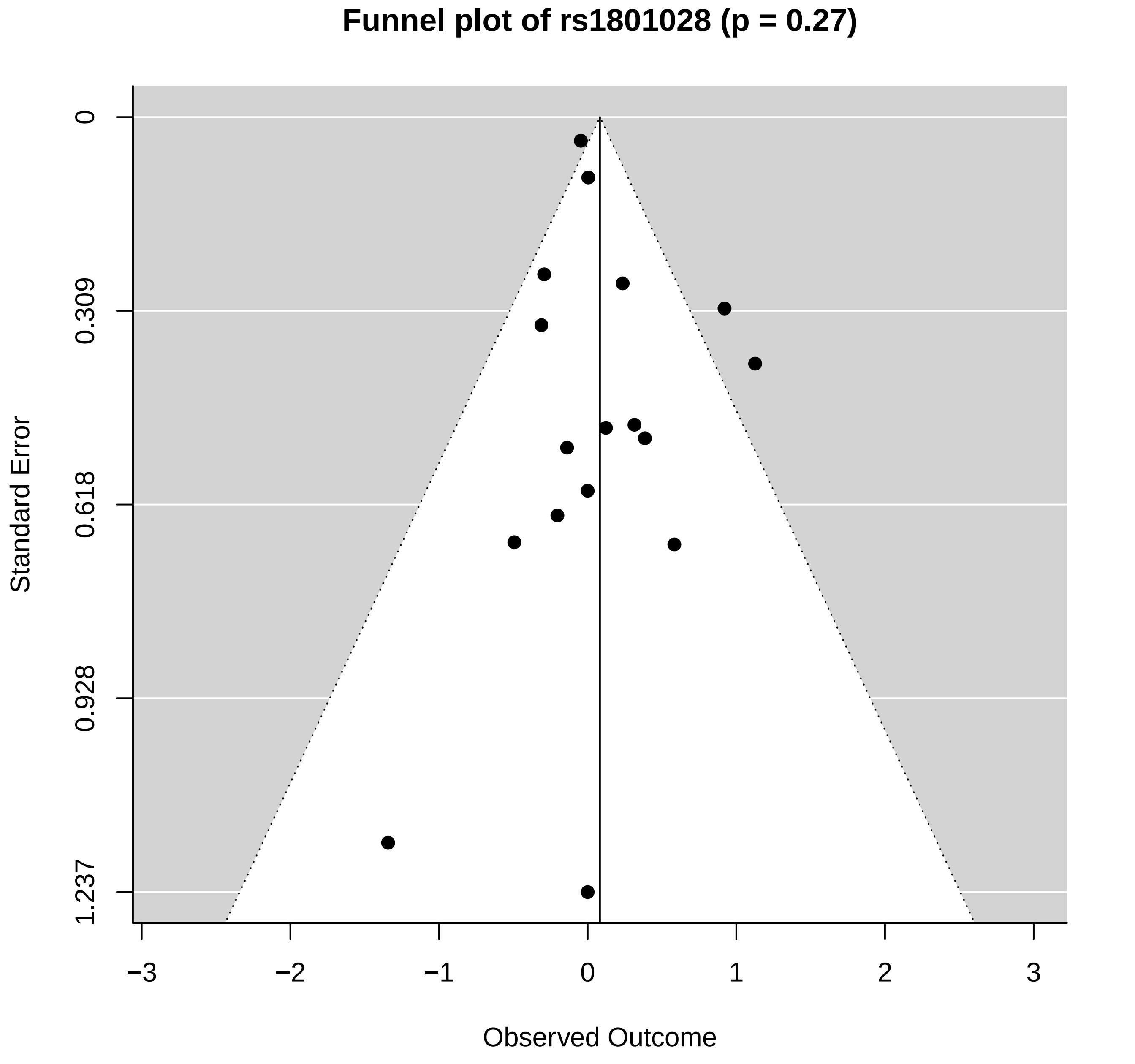
Funnel plot of rs1801028 (p = 0.27)
Standard Error
0
0.309
0.618
0.928
1.237
−3
−2
−1
0
1
2
3
Observed Outcome

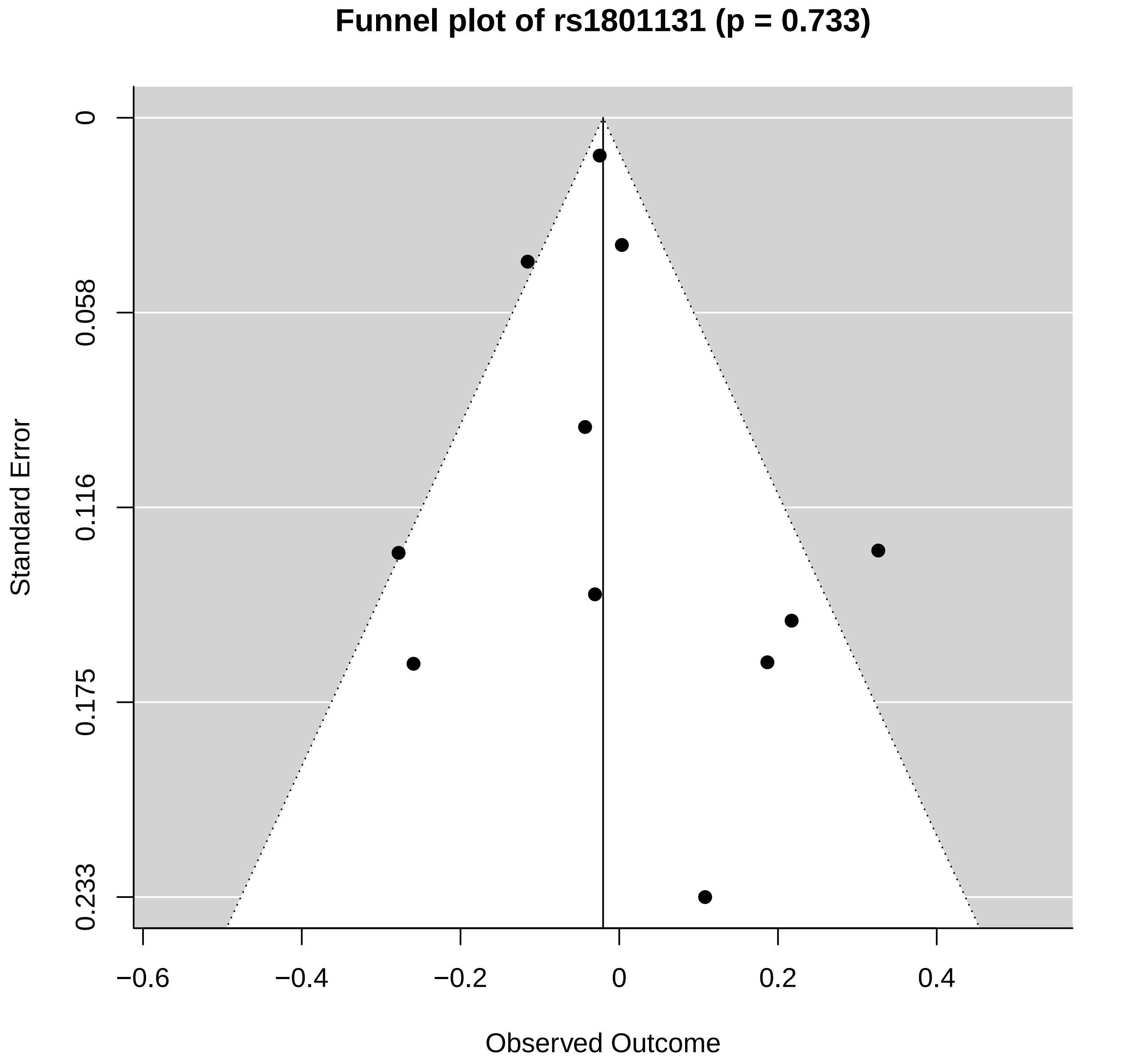

Funnel plot of rs1801131 (p = 0.733)
0
0.058
0.116
0.175
0.233
Standard Error
−0.6
−0.4
−0.2
0
0.2
0.4
Observed Outcome

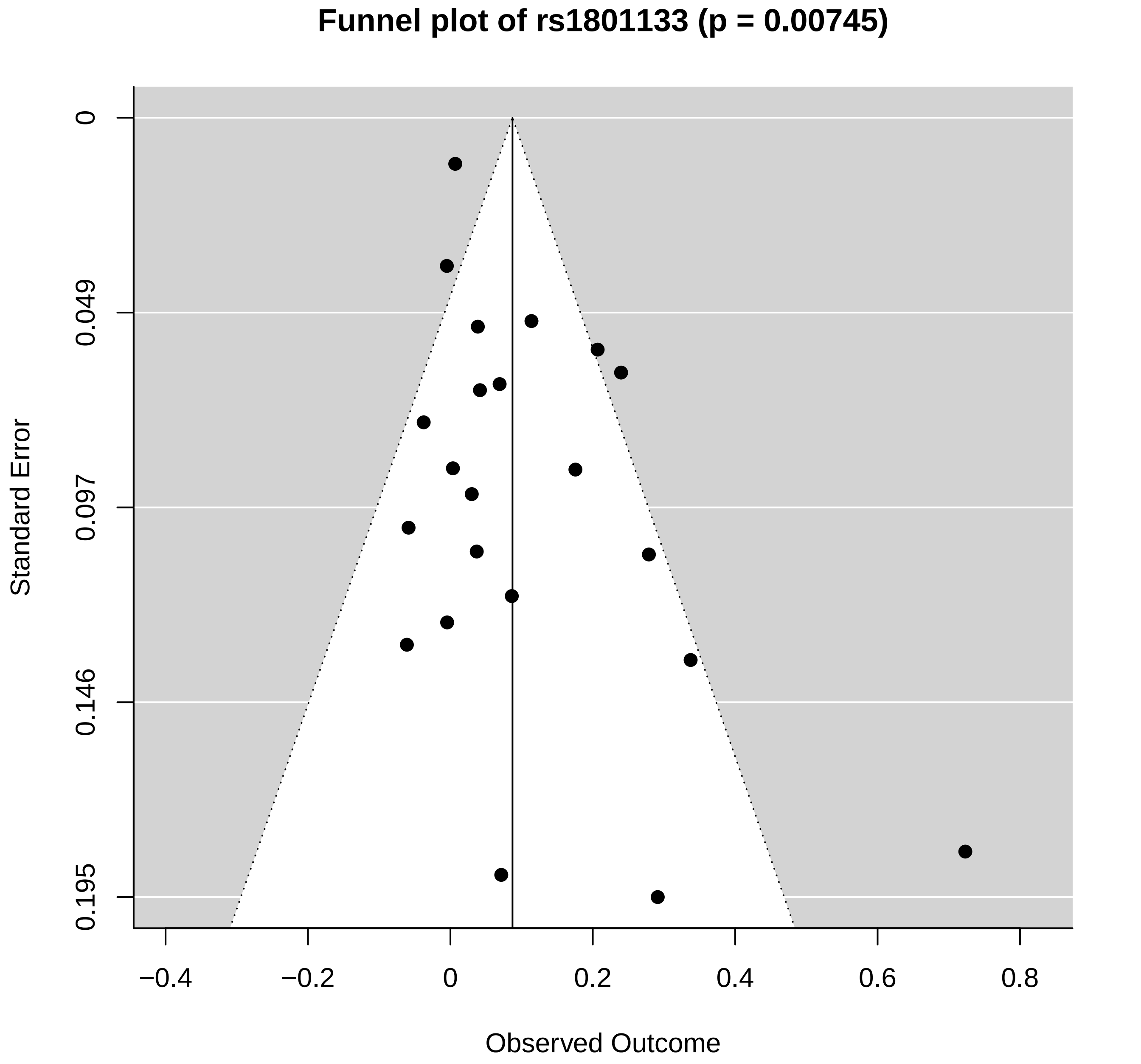

Funnel plot of rs1801133 (p = 0.00745)
0
0.049
0.097
0.146
0.195
Standard Error
−0.4
−0.2
0
0.2
0.4
0.6
0.8
Observed Outcome

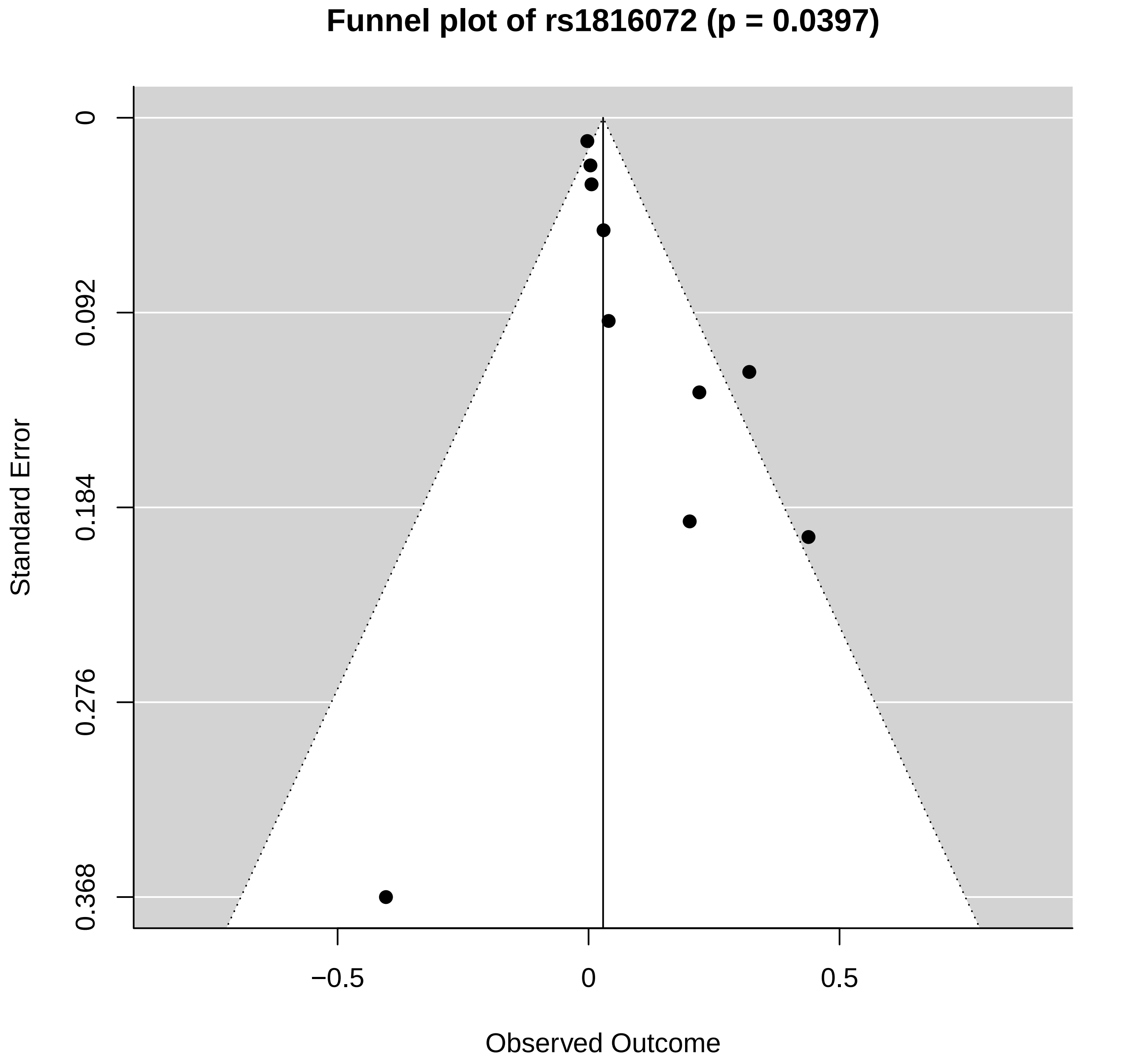

Funnel plot of rs1816072 (p = 0.0397)
Standard Error
0
0.092
0.184
0.276
0.368
−0.5
0
0.5
Observed Outcome

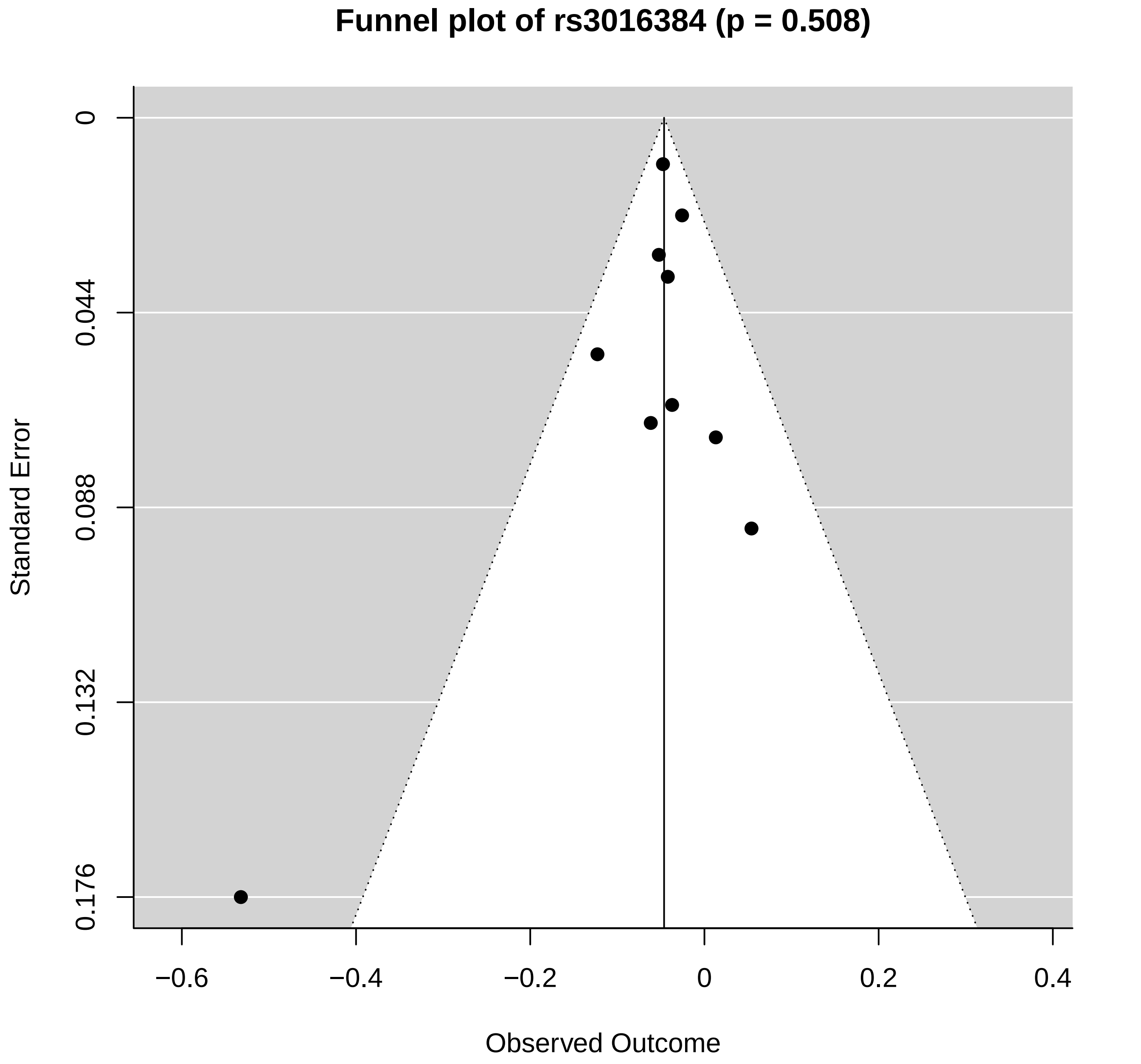

Funnel plot of rs3016384 (p = 0.508)
Standard Error
0
0.044
0.088
0.132
0.176
−0.6
−0.4
−0.2
0
0.2
0.4
Observed Outcome

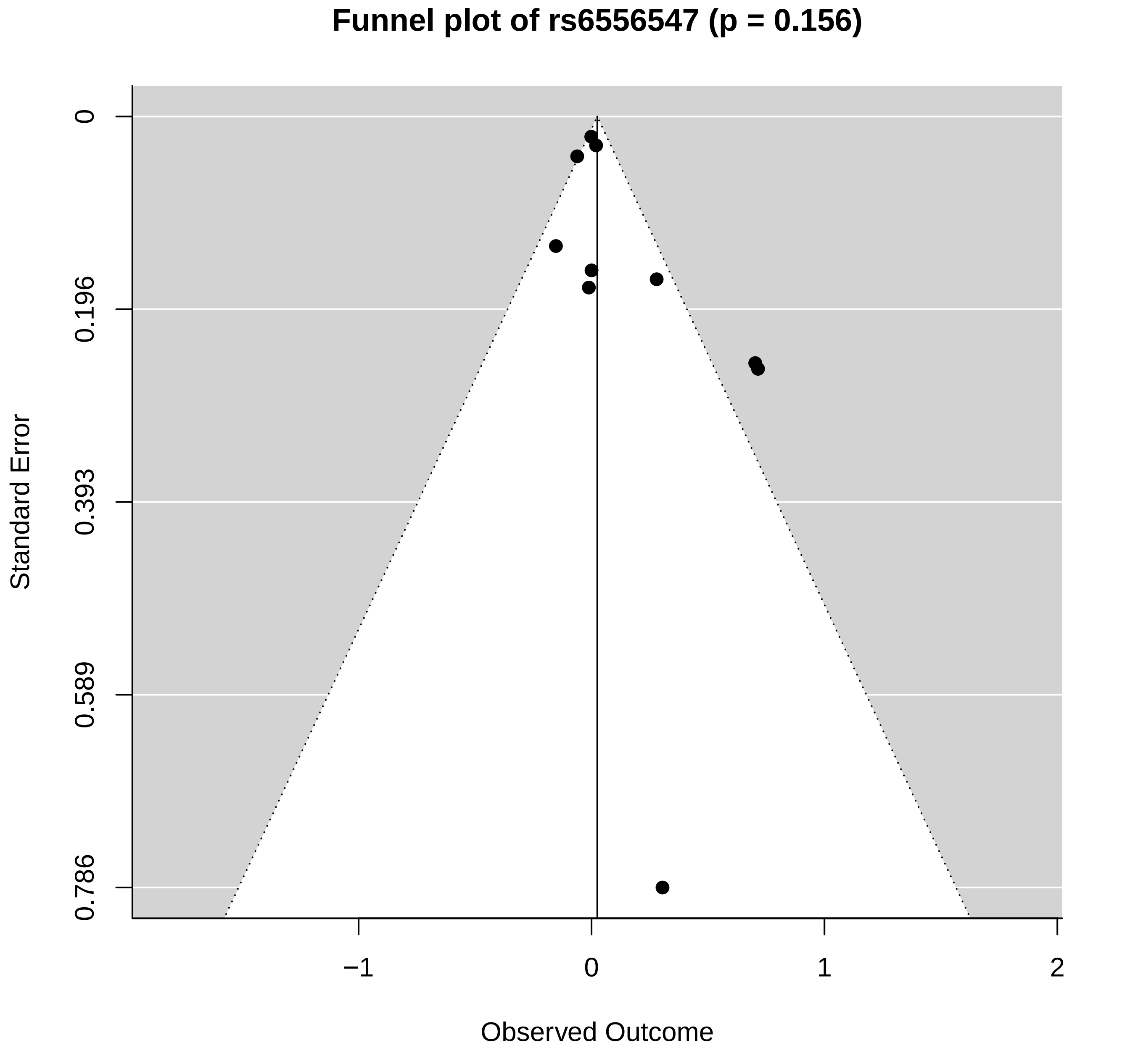
Funnel plot of rs6556547 (p = 0.156)
Standard Error
0
0.196
0.393
0.589
0.786
−1
0
1
2
Observed Outcome

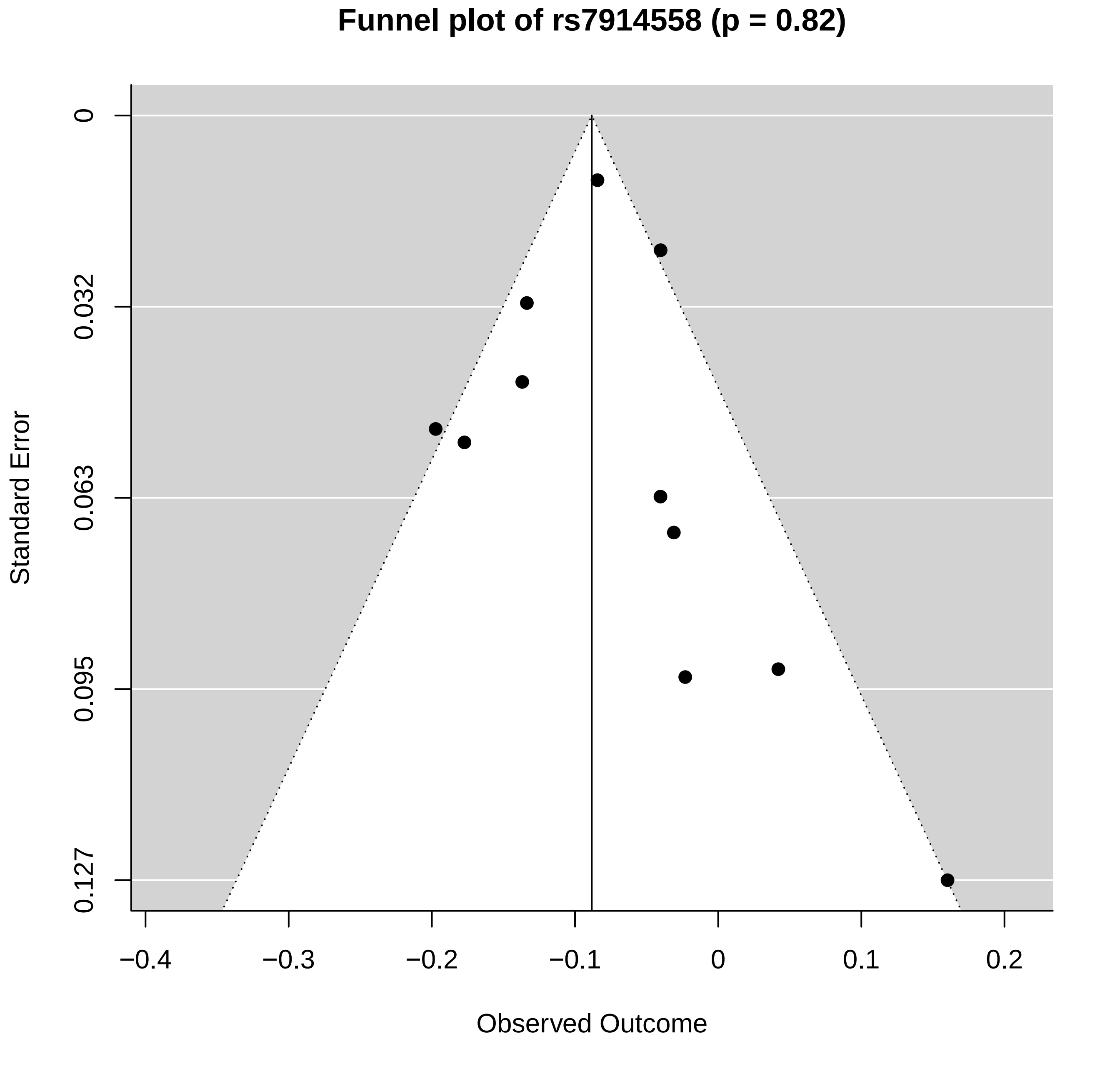

Funnel plot of rs7914558 (p = 0.82)
Standard Error
0
0.032
0.063
0.095
0.127
−0.4
−0.3
−0.2
−0.1
0
0.1
0.2
Observed Outcome
